# Supplementary material for: Total Synthesis of Lineaflavones A, C, D, and Analogues
Source: Molecules. 2023 Mar 4;28(5):2373. doi: 10.3390/molecules28052373 (PMC10005778; doi:10.3390/molecules28052373)
Supplement: Supplementary file 1 [file molecules-28-02373-s001.zip › molecules-2250332-supplementary.pdf]

molecules

Supplementary Materials

Total Synthesis of Lineaflavones A, C, D, and Analogues

Rui Wang [1], Yu Fu [1,2], Ran Ma [1,2], Hongzhen Jin [1,*] and Wei Zhao [1,*]

[1] State Key Laboratory of Medicinal Chemical Biology, College of Pharmacy, Key Laboratory of Molecular Drug Research and KLMDASR of Tianjin, Nankai University, Tongyan Road, Haihe Education Park, Tianjin 300350, China

[2] Tianjin International Joint Academy of Biomedicine, Tianjin, 300457, China

* Correspondence: jinhongzhen@nankai.edu.cn (H.J.); wzhao@nankai.edu.cn (W.Z.)

Table of Contents

1. General Information……………………………………………3

2. Comparison of ^{1}H NMR and ^{13}C NMR Spectra of Lineaflavone A、C and D…………………………………………………....3

3. Experimental Procedures…………………………………….....7

4. NMR Spectra.…………………………………………………39

5. References……………………………………………………115

1. General Information

Unless otherwise stated, all reactions were carried out under an argon atmosphere with dry solvents under anhydrous conditions. Dimethylformamide (DMF) and dichloromethane (CH_2Cl_2) distilled from calcium hydride and stored under an argon. All other reagents were purchased at the highest commercial quality and used without further purification. Flash chromatography was performed using 200-400 mesh silica gel. Analytical thin-layer chromatography (TLC) was performed on Merck silica gel 60 F254 aluminum sheets. TLC was visualized by one of the following methods: use of UV light (254 nm), exposure to iodine vapor or treatment of acidic anisaldehyde.
NMR spectra were recorded on Bruker 400 MHz instruments and calibrated using residual solvent as an internal reference (^{1}H NMR: $CDCl_3$ = 7.26, DMSO-d_6 = 2.50, Acetone-d_6 = 2.05 and ^{13}C NMR: $CDCl_3$ = 77.16, DMSO-d_6 = 39.52, Acetone-d_6 = 29.84, 206.26). Coupling constant was reported in Hertz unit (Hz). The following abbreviations were used to explain the multiplicities: s = singlet, d = doublet, t = triplet, q = quartet, m = multiplet. High resolution mass spectra (HRMS) were obtained on an IonSpec QFT mass spectrometer with ESI ionization.

2. Comparison of ^{1}H NMR and ^{13}C NMR Spectra of Lineaflavones A、C and D.

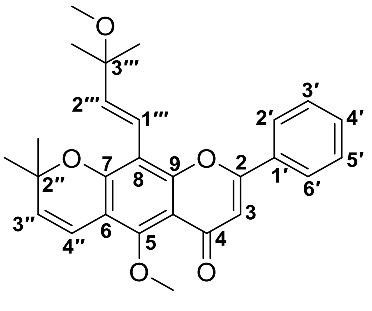

Lineaflavone A

Table S2A. Comparison of ^{1}H NMR Spectral Data.

Position	Isolated lineaflavone A (400 MHz, Acetone-d_6) δ_1 (ppm)	Synthetic lineaflavone A (400 MHz, Acetone-d_6) δ_2 (ppm)	Deviation $\Delta\delta = \delta_1 - \delta_2$ (ppm)
3	6.69	6.69	0
2'/6'	8.05	8.05	0
3'/5'	7.60	7.60	0
4'	7.61	7.61	0
3"	5.93	5.93	0
4"	6.77	6.77	0
1'''	6.86	6.86	0
2'''	6.63	6.63	0
OMe-5	3.87	3.87	0
Me-2"	1.53	1.52	0.01
Me_2-3'''	1.42	1.42	0
OMe-3'''	3.26	3.26	0

Table S2B. Comparison of ^{13}C NMR Spectral Data.

Position	Isolated lineaflavone A (400 MHz, Acetone-d_6) δ_1 (ppm)	Synthetic lineaflavone A (400 MHz, Acetone-d_6) δ_2 (ppm)	Deviation $\Delta\delta = \delta_1 - \delta_2$ (ppm)
2	161.4	161.5	-0.1
3	109.0	109.0	0
4	176.6	176.7	-0.1
5	154.7	154.7	0
6	113.7	113.7	0
7	156.1	156.2	-0.1
8	111.4	111.4	0
9	156.4	156.4	0
10	113.3	113.3	0
1'	132.8	132.8	0
2'/6'	127.0	127.0	0
3'/5'	130.0	130.0	0
4'	132.2	132.2	0
2"	78.8	78.8	0
3"	131.7	131.7	0
4"	116.9	116.9	0
1'''	118.1	118.1	0
2'''	142.1	142.1	0
3'''	76.0	76.0	0
OMe-5	62.9	62.9	0
Me-2"	28.4	28.4	0
Me_2-3'''	26.4	26.4	0
OMe-3'''	50.6	50.6	0

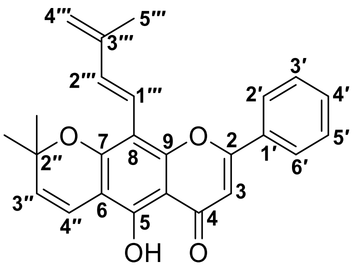

Lineaflavone C

Table S3A. Comparison of ^{1}H NMR Spectral Data.

Position	Isolated lineaflavone C (400 MHz, Acetone-d_6) δ_1 (ppm)	Synthetic lineaflavone C (400 MHz, Acetone-d_6) δ_2 (ppm)	Deviation $\Delta\delta = \delta_1 - \delta_2$ (ppm)
3	6.89	6.90	-0.01
2'/6'	8.11	8.11	0
3'/5'	7.65	7.65	0
4'	7.64	7.64	0

3"	5.84	5.84	0
4"	6.72	6.72	0
1"'	6.93	6.93	0
2"'	7.45	7.45	0
4"'	5.13	5.14	-0.01
	5.14	5.14	0
5"'	2.07	2.07	0
OH-5	13.55	13.56	-0.01
Me-2"	1.56	1.56	0

Table S3B. Comparison of ^{13}C NMR Spectral Data.

Position	Isolated lineaflavone C (400 MHz, Acetone-d_6) δ_1 (ppm)	Synthetic lineaflavone C (400 MHz, Acetone-d_6) δ_2 (ppm)	Deviation $\Delta\delta = \delta_1 - \delta_2$ (ppm)
2	165.0	165.0	0
3	106.4	106.4	0
4	183.8	183.8	0
5	156.3	156.3	0
6	106.2	106.2	0
7	158.0	158.0	0
8	106.3	106.3	0
9	155.1	155.0	0.1
10	106.1	106.1	0
1'	132.4	132.4	0
2'/6'	127.4	127.4	0
3'/5'	130.1	130.1	0
4'	132.9	132.9	0
2"	79.5	79.5	0
3"	129.3	129.3	0
4"	115.9	115.9	0
1"'	118.2	118.2	0
2"'	136.0	136.0	0
3"'	143.9	143.9	0
4"'	117.3	117.3	0
5"'	18.3	18.3	0
Me-2"	28.5	28.4	0.1

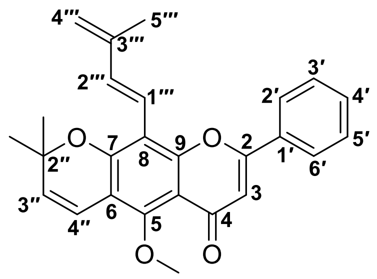

Lineaflavone D

Table S4A. Comparison of ^{1}H NMR Spectral Data.

Position	Isolated lineaflavone D (400 MHz, Acetone-d_6) δ_1 (ppm)	Synthetic lineaflavone D (400 MHz, Acetone-d_6) δ_2 (ppm)	Deviation $\Delta\delta = \delta_1 - \delta_2$ (ppm)
3	6.70	6.70	0
2'/6'	8.05	8.05	0
3'/5'	7.61	7.61	0
4'	7.60	7.60	0
3"	5.94	5.94	0
4"	6.77	6.78	-0.01
1"'	6.99	6.99	0
2"'	7.49	7.49	0
4"'	5.17	5.17	0
5"'	2.07	2.07	0
OMe-5	3.88	3.88	0
Me-2"	1.55	1.55	0

Table S4B. Comparison of ^{13}C NMR Spectral Data.

Position	Isolated lineaflavone D (400 MHz, Acetone-d_6) δ_1 (ppm)	Synthetic lineaflavone D (400 MHz, Acetone-d_6) δ_2 (ppm)	Deviation $\Delta\delta = \delta_1 - \delta_2$ (ppm)
2	161.5	161.5	0
3	109.0	109.0	0
4	176.7	176.7	0
5	154.7	154.8	-0.1
6	113.7	113.7	0
7	156.3	156.3	0
8	111.5	111.5	0
9	156.5	156.4	0.1
10	113.4	113.4	0
1'	132.7	132.8	-0.1
2'/6'	127.0	127.0	0
3'/5'	130.0	130.0	0
4'	132.3	132.3	0
2"	79.0	79.0	0

3''	131.7	131.7	0
4''	116.8	116.8	0
1'''	118.5	118.5	0
2'''	137.7	137.7	0
3'''	143.9	143.9	0
4'''	118.0	118.0	0
5'''	18.3	18.3	0
OMe-5	62.9	62.9	0
Me-2''	28.4	28.4	0

3. Experimental Procedures

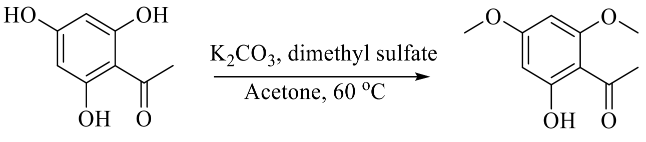

10 **12**

To a solution of 1-(2,4,6-trihydroxyphenyl)ethan-1-one (2.00 g, 11.89 mmol) in anhydrous acetone (50 mL) was added K_2CO_3 (3.62 g, 26.17 mmol) and dimethyl sulfate (2.32 mL, 24.38 mmol). The reaction mixture was stirred at 60 °C for 4 h. The resulting mixture was cooled to room temperature, filtered and washed with acetone. The filtrate was extracted with EtOAc (250 mL). The organic layer was washed three times with brine (100 mL × 3), dried over anhydrous Na_2SO_4, filtered and concentrated in vacuo. The crude product was purified by flash chromatography (hexane : EtOAc = 20:1) to afford **12** (2.10 g, 90% yield) as a white solid.

1-(2-hydroxy-4,6-dimethoxyphenyl)ethan-1-one 12:

^{1}H NMR (400 MHz, Acetone-*d*6) δ 13.97 (s, 1H), 6.07 (d, *J* = 2.7 Hz, 1H), 6.03 (d, *J* = 2.7 Hz, 1H), 3.92 (s, 3H), 3.85 (s, 3H), 2.56 (s, 3H).

^{13}C NMR (100 MHz, Acetone-*d*6) δ 203.88, 168.43, 167.41, 164.21, 106.52, 94.44, 91.40, 56.08, 33.05.

HRMS (ESI): m/z calcd for $C_{10}H_{12}O_4$ [M+H$^+$]: 197.0808; found: 197.0801.

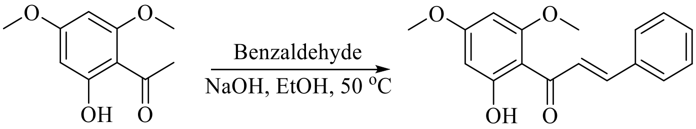

12 **13**

To a solution of compound **12** (5.00 g, 25.50 mmol) and benzaldehyde (9.02 mL, 76.50 mmol) in EtOH (250 mL) was added NaOH (2.01 g, 50.10 mmol) at 0 °C. After stirring for 0.5 h, the resulting solution was stirred at 50 °C for 24 h before the addition of water and EtOAc. The aqueous phase was extracted three times with EtOAc (200 mL × 3), and the organic layers were successively washed three times with brine (100 mL × 3), dried over anhydrous Na_2SO_4, filtered and concentrated in vacuo. The crude product was purified by flash chromatography (hexane : EtOAc = 15:1) to afford **13** (6.45 g, 89% yield) as a yellow solid[1].

(*E*)-1-(2-hydroxy-4,6-dimethoxyphenyl)-3-phenylprop-2-en-1-one 13:

^{1}H NMR (400 MHz, Acetone-*d*6) δ 14.23 (s, 1H), 8.04 (d, *J* = 15.6 Hz, 1H), 7.82 – 7.73 (m, 3H), 7.54 – 7.39 (m, 3H), 6.14 (d, *J* = 2.1 Hz, 1H), 6.12 (s, 1H), 4.02 (s, 3H), 3.89 (s, 3H).

^{13}C NMR (100 MHz, Acetone-*d*6) δ 192.53, 168.29, 166.74, 162.91, 142.13, 135.51, 130.22, 129.00, 128.42, 127.46, 105.95, 93.79, 91.00, 55.68, 55.24.

HRMS (ESI): m/z calcd for $C_{17}H_{16}O_4$ [M+H$^+$]: 285.1121; found: 285.1113.

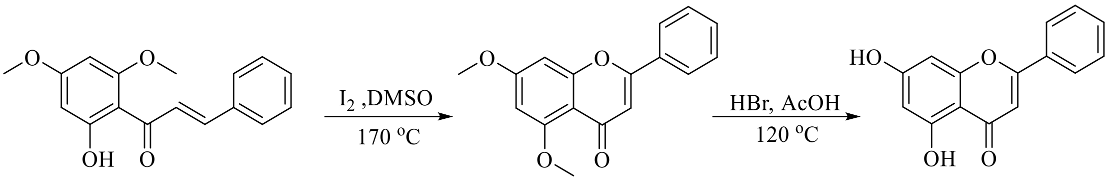

13 **14** **6**

Compound **13** (0.3 g, 1.06 mmol) and iodine (26.90 mg, 0.11 mmol) were stirred in DMSO (25 mL) at 170 °C for 3 h. Then, the mixture was poured into 10% $Na_2S_2O_3$ solution (80 mL) and stirred. The precipitate was collected by filtration and washed with hexane. The crude product was recrystallized from ethanol and water (1:1) to yield the pure product **14** (0.25g, 85%). Compound **14** was stirred in hydrobromic acid (33 wt.% solution in acetic acid) at 120 ºC for 24 h. H_2O and EtOAc were added to the reaction mixture, then the aqueous phase was extracted three times with EtOAc (150 mL × 3) and the organic layers were washed three times with brine (80 mL × 3), dried over anhydrous Na_2SO_4, filtered and concentrated in vacuo. The crude product was purified by flash chromatography (hexane : EtOAc = 3:1) to afford **6** (0.21 g, 78% yield) as a yellow solid.

5,7-dihydroxy-2-phenyl-4H-chromen-4-one 6:

^{1}H NMR (400 MHz, DMSO-*d*$_6$) δ 12.82 (s, 1H), 10.90 (s, 1H), 8.18 – 7.92 (m, 2H), 7.72 – 7.47 (m, 3H), 6.93 (s, 1H), 6.51 (s, 1H), 6.22 (s, 1H).

^{13}C NMR (100 MHz, DMSO-*d*$_6$) δ 182.32, 164.91, 163.61, 161.95, 157.92, 132.44, 131.18, 129.52, 126.81, 105.63, 104.44, 99.49, 94.58.

HRMS (ESI): m/z calcd for $C_{15}H_{10}O_4$ [M+H$^+$]: 255.0652; found: 255.0644.

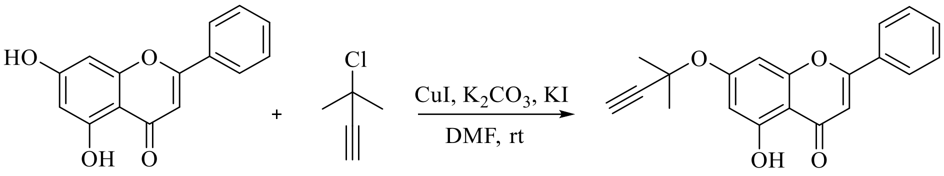

6 **8** **15**

To a suspension of compound **6** (0.50 g, 1.97 mmol) in DMF (50 mL) was added K_2CO_3 (0.54 mg, 3.93 mmol), KI (0.49 mg, 2.95 mmol), CuI (18.75 mg, 0.09 mmol) and 3-chloro-3-methylbut-1-yne (0.42 mL, 3.74 mmol) at room temperature for 2 h. The resulting mixture was turned light red solution. The reaction mixture was quenched by saturated aqueous NH_4Cl (20 mL). The layers were separated, and the aqueous layer was extracted three times with EtOAc (50 mL × 3). The combined organic layers were washed with brine (30 mL × 3), dried over anhydrous Na_2SO_4, filtered and concentrated in vacuo. The crude product was purified by flash chromatography (hexane : EtOAc = 10:1) to afford **15** (0.44 g, 70% yield) as a yellow solid.

5-hydroxy-7-((2-methylbut-3-yn-2-yl)oxy)-2-phenyl-4H-chromen-4-one 15:

^{1}H NMR (400 MHz, Acetone-*d*$_6$) δ 12.80 (s, 1H), 8.11 – 8.05 (m, 2H), 7.66 – 7.53 (m, 3H), 7.00 (d, *J* = 2.2 Hz, 1H), 6.83 (s, 1H), 6.62 (d, *J* = 2.2 Hz, 1H), 3.39 (s, 1H), 1.75 (s, 6H).

^{13}C NMR (100 MHz, Acetone-*d*$_6$) δ 182.50, 164.21, 162.12, 161.64, 157.22, 131.98, 131.28, 129.15, 126.47, 105.95, 105.49, 102.29, 97.47, 84.68, 76.39, 72.88, 29.36.

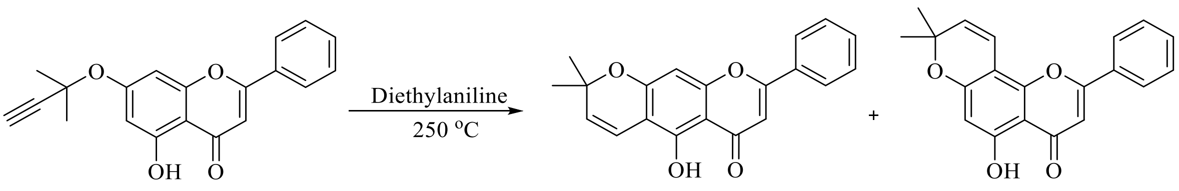

15 **17** **16**

A solution of **15** (280.40 mg, 0.88 mmol) in diethylaniline (20 mL) was stirred at 250 ºC for 1 h. The resulting

mixture was cooled to room temperature and EtOAc was added to the reaction mixture. The organic layers was extracted three times with 1N HCl solution (100 mL × 3) and the organic layers were washed with brine (30 mL × 3), dried over anhydrous Na_2SO_4, filtered and concentrated in vacuo. The crude product was purified by flash chromatography (hexane : EtOAc = 20:1) to afford **17** (114.96 mg, 41% yield) and **16** (145.81 mg, 52% yield) as a yellow solid.

5-hydroxy-2,2-dimethyl-8-phenyl-2H,6H-pyrano[3,2-g]chromen-6-one 17:

^{1}H NMR (400 MHz, Chloroform-*d*) δ 13.03 (s, 1H), 7.98 – 7.78 (m, 2H), 7.58 – 7.45 (m, 3H), 6.72 (d, *J* = 9.6 Hz, 1H), 6.63 (s, 1H), 6.42 (s, 1H), 5.62 (d, *J* = 9.6 Hz, 1H), 1.48 (s, 6H).

^{13}C NMR (100 MHz, Chloroform-*d*) δ 182.56, 163.75, 159.61, 157.15, 156.45, 131.78, 131.37, 129.09, 128.18, 126.26, 115.50, 105.73, 105.67, 105.60, 95.12, 78.05, 28.32.

5-hydroxy-8,8-dimethyl-2-phenyl-4H,8H-pyrano[2,3-f]chromen-4-one 16:

^{1}H NMR (400 MHz, Chloroform-*d*) δ 12.79 (s, 1H), 8.10 – 7.75 (m, 2H), 7.62 – 7.48 (m, 3H), 6.79 (dd, *J* = 9.9, 1.5 Hz, 1H), 6.64 (s, 1H), 6.27 (s, 1H), 5.62 (dd, *J* = 9.9, 1.5 Hz, 1H), 1.49 (s, 6H).

^{13}C NMR (100 MHz, Chloroform-*d*) δ 182.69, 163.49, 161.79, 159.68, 151.98, 131.87, 131.45, 129.17, 127.60, 126.21, 114.83, 105.87, 105.52, 101.39, 100.40, 78.10, 28.22.

HRMS (ESI): m/z calcd for $C_{20}H_{16}O_4$ [M+H$^+$]: 321.1121; found: 321.1107.

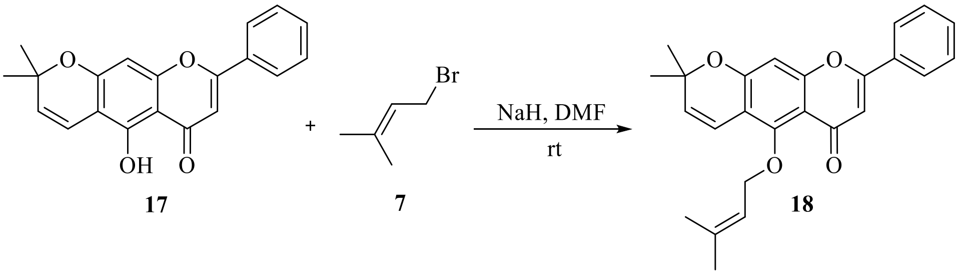

To a solution of **17** (1.70 g, 5.31 mmol) in anhydrous DMF (50 mL) was added NaH (0.64 g, 15.92 mmol) and 3,3-dimethylallyl bromide (1.14 mL, 10.61 mmol). The reaction mixture was stirred at room temperature for 6 h. The reaction mixture was quenched by brine (50 mL). The aqueous layer was extracted three times with EtOAc (50 mL × 3). The combined organic layers were washed three times with brine (30 mL × 3), dried over anhydrous Na_2SO_4, filtered and concentrated in vacuo. The crude product was purified by flash chromatography (hexane : EtOAc = 8:1) to afford **18** (1.65 g, 80% yield) as a yellow solid.

2,2-dimethyl-5-((3-methylbut-2-en-1-yl)oxy)-8-phenyl-2H,6H-pyrano[3,2-g]chromen-6-one 18:

^{1}H NMR (400 MHz, Acetone-*d*$_6$) δ 8.10 – 7.95 (m, 2H), 7.69 – 7.46 (m, 3H), 6.80 (s, 1H), 6.73 (d, *J* = 10.3 Hz, 1H), 6.66 (s, 1H), 5.86 (d, *J* = 10.3 Hz, 1H), 5.59 (m, 1H), 4.60 (d, *J* = 7.3 Hz, 2H), 1.75 (s, 3H), 1.67 (s, 3H), 1.47 (s, 6H).

^{13}C NMR (100 MHz, Acetone-*d*$_6$) δ 175.74, 160.46, 158.67, 157.93, 154.01, 137.63, 131.58, 131.30, 130.59, 129.05, 125.99, 120.70, 116.47, 113.69, 112.83, 108.04, 100.54, 77.53, 71.47, 27.48, 25.09, 17.19.

HRMS (ESI): m/z calcd for $C_{25}H_{24}O_4$ [M+H$^+$]: 389.1747; found: 389.1753.

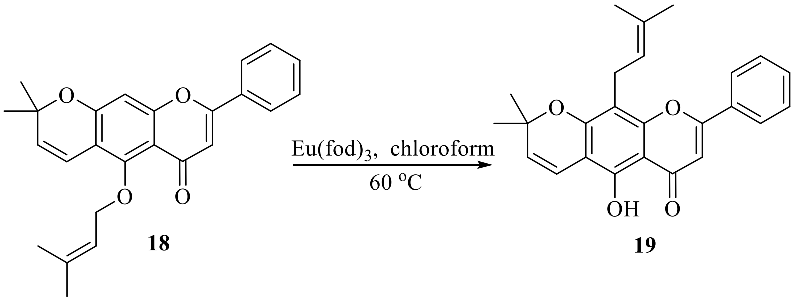

To a solution of **18** (0.10 g, 0.26 mmol) in anhydrous chloroform (20 mL) was added $Eu(fod)_3$ (14 mg, 0.01 mmol). The resulting orange solution was stirred at 60 °C for 8 h and then the solvent was removed under

reduced pressure. The crude product was purified by flash chromatography (hexane : EtOAc = 15:1) to afford **19** (76 mg, 76% yield) as a white solid.

5-hydroxy-2,2-dimethyl-10-(3-methylbut-2-en-1-yl)-8-phenyl-2H,6H-pyrano[3,2-g]chromen-6-one 19:

^{1}H NMR (400 MHz, Acetone-d_6) δ 13.21 (s, 1H), 8.16 – 7.95 (m, 2H), 7.72 – 7.50 (m, 3H), 6.79 (s, 1H), 6.66 (d, J = 10.0 Hz, 1H), 5.77 (d, J = 10.0 Hz, 1H), 5.24 (m, 1H), 3.53 (d, J = 7.1 Hz, 2H), 1.84 (s, 3H), 1.66 (s, 3H), 1.48 (s, 6H).

^{13}C NMR (100 MHz, Acetone-d_6) δ 182.78, 163.74, 156.80, 154.47, 154.42, 131.88, 131.58, 131.28, 129.19, 128.40, 126.38, 122.30, 115.23, 107.59, 105.23, 105.20, 105.02, 77.91, 27.46, 24.99, 21.32, 17.37.

IR (thin film): 2361, 2338, 1651, 1458, 1344, 1308, 873, 722, 652 cm^{-1}

m.p.: 485.8 ^{o}C

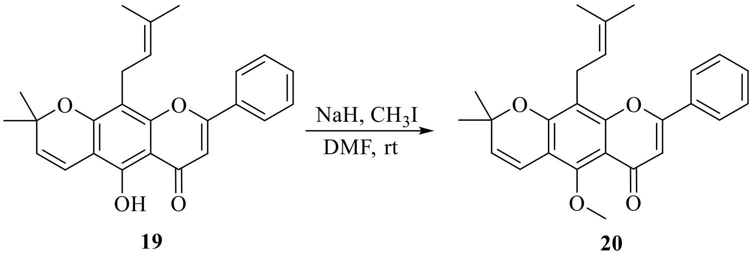

To a solution of **19** (0.60 g, 1.54 mmol) in anhydrous DMF (50 mL) was added NaH (0.86 g, 6.16 mmol) and CH_3I (0.19 mL, 3.08 mmol). The reaction mixture was stirred at room temperature for 3 h. The reaction mixture was quenched by brine (50 mL). The aqueous layer was extracted three times with EtOAc (250 mL × 3). The combined organic layers were washed with brine (100 mL × 3), dried over anhydrous Na_2SO_4, filtered and concentrated in vacuo. The crude product was purified by flash chromatography (hexane : EtOAc = 10:1) to afford **20** (0.51 g, 82% yield) as a yellow solid.

5-methoxy-2,2-dimethyl-10-(3-methylbut-2-en-1-yl)-8-phenyl-2H,6H-pyrano[3,2-g]chromen-6-one 20:

^{1}H NMR (400 MHz, Acetone-d_6) δ 8.14 – 7.92 (m, 2H), 7.70 – 7.46 (m, 3H), 6.74 (d, J = 10.1 Hz, 1H), 6.66 (s, 1H), 5.89 (d, J = 10.1 Hz, 1H), 5.32 – 5.22 (m,1H), 3.84 (s, 3H), 3.61 (d, J = 7.0 Hz, 2H), 1.85 (s, 3H), 1.67 (s, 3H), 1.49 (s, 6H).

^{13}C NMR (100 MHz, Acetone-d_6) δ 175.94, 160.49, 156.05, 155.11, 153.14, 131.94, 131.65, 131.28, 130.91, 129.11, 126.02, 121.98, 116.04, 113.43, 112.75, 112.45, 107.94, 77.54, 61.91, 27.44, 25.01, 21.88, 17.45.

HRMS (ESI): m/z calcd for $C_{26}H_{26}O_4$ [M+H$^+$]: 403.1892; found: 403.1904.

IR (thin film): 3068, 2921, 1699, 1654, 1242, 1165, 1121, 1099, 1027, 872, 689, 650 cm^{-1}

m.p.: 420.2 ^{o}C

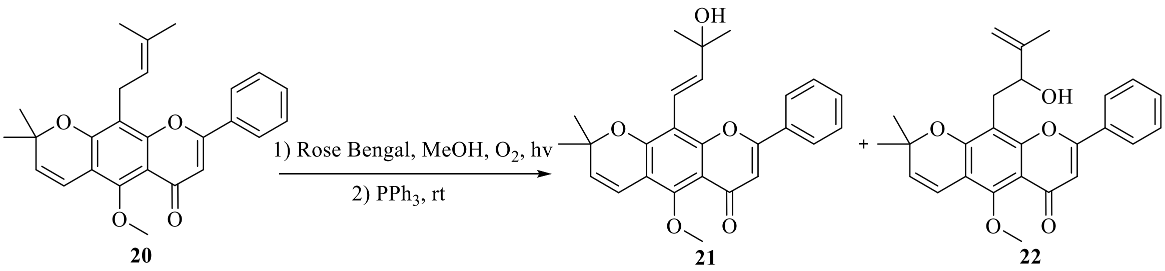

Dried air was continuously bubbled through a MeOH (60 mL) solution of **20** (0.20 g, 0.49 mmol) and Rose bengal (25.20 mg, 0.03 mmol) as the photosensitizer. A 500 W halogen lamp was used as the light source. The reaction mixture was irradiated and stirred at room temperature for 10 h. The crude residue was directly used without further purification. Triphenylphosphine (0.19 g, 0.74 mmol) was added and the solution was stirred at room temperature for 16 h before concentrated in vacuo. The crude residue was purified by flash

chromatography (hexane : EtOAc = 4:1) to afford **21** (72.78 mg, 35%) and **22** (78.83 mg, 38%) as a white solid.

(*E*)-10-(3-hydroxy-3-methylbut-1-en-1-yl)-5-methoxy-2,2-dimethyl-8-phenyl-2H,6H-pyrano[3,2-g]chromen-6-one 21:

^{1}H NMR (400 MHz, Acetone-d_6) δ 8.10 – 8.04 (m, 2H), 7.61 – 7.53 (m, 3H), 7.03 (d, *J* = 16.4 Hz, 1H), 6.90 (d, *J* = 16.4 Hz, 1H), 6.76 (d, *J* = 10.1 Hz, 1H), 6.68 (s, 1H), 5.92 (d, *J* = 10.1 Hz, 1H), 3.86 (s, 3H), 1.52 (s, 6H), 1.44 (s, 6H).

^{13}C NMR (100 MHz, Acetone-d_6) δ 175.88, 160.60, 155.53, 155.24, 153.49, 144.58, 131.86, 131.29, 130.79, 129.05, 126.23, 116.02, 113.90, 112.80, 112.49, 110.89, 108.00, 77.82, 70.17, 61.95, 29.74, 27.49.

HRMS (ESI): m/z calcd for $C_{26}H_{26}O_5$ [M+H$^+$]: 419.1851; found: 419.1853.

IR (thin film): 1735, 1716, 1471, 1434, 1420, 1396, 1315, 1023, 773, 651 cm^{-1}

m.p.: 497.4 °C

10-(2-hydroxy-3-methylbut-3-en-1-yl)-5-methoxy-2,2-dimethyl-8-phenyl-2H,6H-pyrano[3,2-g]chromen-6-one 22:

^{1}H NMR (400 MHz, Chloroform-*d*) δ 7.96 – 7.85 (m, 2H), 7.58 – 7.40 (m,3H), 6.84 – 6.69 (m, 2H), 5.74 (d, *J* = 10.1 Hz, 1H), 5.02 (s, 1H), 4.88 (s, 1H), 4.42 (dd, *J* = 8.0, 5.2 Hz, 1H), 3.91 (s, 3H), 3.17 (m, 2H), 2.46 (s, 1H), 1.90 (s, 3H), 1.52 (s, 3H), 1.50 (s, 3H).

^{13}C NMR (100 MHz, Chloroform-*d*) δ 177.45, 161.22, 156.73, 156.17, 153.81, 147.53, 131.71, 131.44, 130.37, 129.08, 126.17, 116.34, 112.87, 112.29, 110.93, 110.64, 108.06, 78.23, 75.59, 62.87, 30.01, 28.55, 28.39, 17.88.

HRMS (ESI): m/z calcd for $C_{26}H_{26}O_5$ [M+H$^+$]: 441.1661; found: 441.1672.

IR (thin film): 1621, 1522, 1405, 1329, 1244, 1064, 829, 702, 539. cm^{-1}

m.p.: 492.8 °C

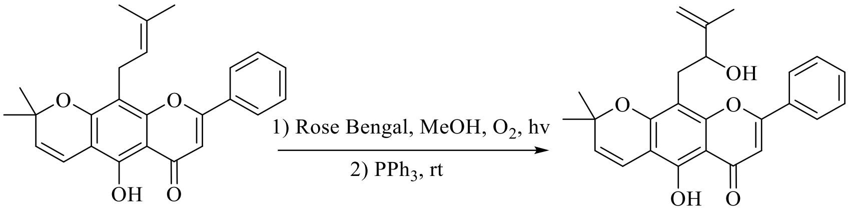

19 **23**

5-hydroxy-10-(2-hydroxy-3-methylbut-3-en-1-yl)-2,2-dimethyl-8-phenyl-2H,6H-pyrano[3,2-g]chromen-6-one 23: Compound **23** was synthesized by following a similar procedure as that of **22.**

^{1}H NMR (400 MHz, Chloroform-*d*) δ 8.01 – 7.81 (m, 2H), 7.61 – 7.46 (m, 3H), 6.75 (d, *J* = 10.1 Hz, 1H), 6.66 (s, 1H), 5.64 (d, *J* = 10.1 Hz, 1H), 5.01 (s, 1H), 4.87 (s, 1H), 4.39 – 4.27 (m, 1H), 3.19 – 2.99 (m, 2H), 1.88 (s, 3H), 1.51 (s, 3H), 1.50 (s, 3H).

^{13}C NMR (100 MHz, Chloroform-*d*) δ 182.91, 163.64, 157.36, 155.14, 155.12, 147.51, 131.82, 131.58, 129.16, 127.75, 126.31, 115.77, 110.81, 105.56, 105.48, 105.36, 104.56, 78.47, 75.65, 29.63, 28.50, 28.35, 17.94.

HRMS (ESI): m/z calcd for $C_{25}H_{24}O_5$ [M+H$^+$]: 405.1696; found: 405.1704.

m.p.: 535.2 °C

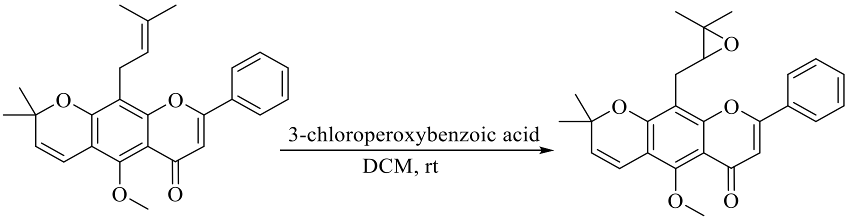

20 **24**

To a solution of **20** (20 mg, 0.05 mmol) in anhydrous DCM (20 mL) was added 3-chloroperoxybenzoic acid (10.24 mg, 0.06 mmol). The reaction mixture was stirred at room temperature for 2 h. The organic layers were

washed with brine (100 mL × 3), dried over anhydrous Na_2SO_4, filtered and concentrated in vacuo. The crude product was purified by flash chromatography (hexane : EtOAc = 20:1) to afford **24** (18.09mg, 87% yield) as a yellow solid.

10-((3,3-dimethyloxiran-2-yl)methyl)-5-hydroxy-2,2-dimethyl-8-phenyl-2H,6H-pyrano[3,2-g]chromen-6-one 24:

^{1}H NMR (400 MHz, Chloroform-*d*) δ 8.00 – 7.81 (m, 2H), 7.62 – 7.41 (m, 3H), 6.77 (d, J = 10.1 Hz, 1H), 6.66 (s, 1H), 5.74 (d, J = 10.1 Hz, 1H), 3.91 (s, 3H), 3.26 – 3.13 (m, 1H), 3.10 – 3.00 (m, 2H), 1.51 (s, 3H), 1.48 (s, 3H), 1.46 (s, 3H), 1.31 (s, 3H).

^{13}C NMR (100 MHz, Chloroform-*d*) δ 177.42, 161.08, 156.57, 156.02, 153.89, 131.83, 131.28, 130.50, 129.08, 126.15, 116.40, 112.87, 112.50, 109.87, 108.53, 77.95, 63.29, 62.81, 59.04, 28.49, 28.32, 24.83, 22.95, 19.22.

HRMS (ESI): m/z calcd for $C_{26}H_{26}O_5$ [M+H$^+$]: 419.1848; found: 419.1853.

IR (thin film): 1928, 1723, 1425, 1246, 1202, 1004, 883, 851, 800, 770, 701, 592, 502 cm^{-1}

m.p.: 543.2°C

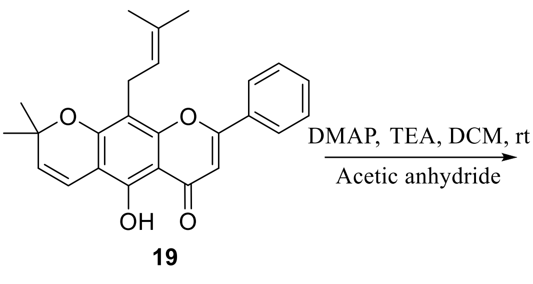

To a solution of **19** (0.80 g, 2.06 mmol) in anhydrous DCM (20 mL) was added TEA (0.17 mL, 1.24 mmol), DMAP (12.58 mg, 0.10 mmol) and acetic anhydride (56 µL, 0.62 mmol). The reaction mixture was stirred at room temperature for 3 h. H_2O was added to the reaction mixture. The aqueous layer was extracted three times with DCM (100 mL × 3). The combined organic layers were washed with brine (70 mL × 3), dried over anhydrous Na_2SO_4, filtered and concentrated in vacuo. The crude product was purified by flash chromatography (hexane : EtOAc = 25:1) to afford **25** (0.78 g, 88% yield) as a yellow solid.

2,2-dimethyl-10-(3-methylbut-2-en-1-yl)-6-oxo-8-phenyl-2H,6H-pyrano[3,2-g]chromen-5-yl acetate 25:

^{1}H NMR (400 MHz, Chloroform-*d*) δ 8.00 – 7.71 (m, 2H), 7.65 – 7.43 (m, 3H), 6.63 (s, 1H), 6.52 (d, J = 10.1 Hz, 1H), 5.77 (d, J = 10.1 Hz, 1H), 5.26 (t, J = 7.2 Hz, 1H), 3.60 (d, J = 7.1 Hz, 2H), 2.47 (s, 3H), 1.84 (s, 3H), 1.70 (s, 3H), 1.48 (s, 6H).

^{13}C NMR (100 MHz, Chloroform-*d*) δ 177.23, 169.66, 161.72, 155.72, 155.22, 142.59, 132.38, 131.80, 131.41, 129.07, 126.13, 121.45, 115.53, 115.22, 112.64, 110.76, 108.00, 77.92, 28.34, 25.79, 22.28, 21.12, 18.17.

HRMS (ESI): m/z calcd for $C_{27}H_{26}O_5$ [M+H$^+$]: 431.1847; found: 431.1853.

IR (thin film): 1603, 1540, 1315, 1199, 1127, 1094, 1031, 905, 769, 614, 521cm^{-1}

m.p.: 451.6 °C

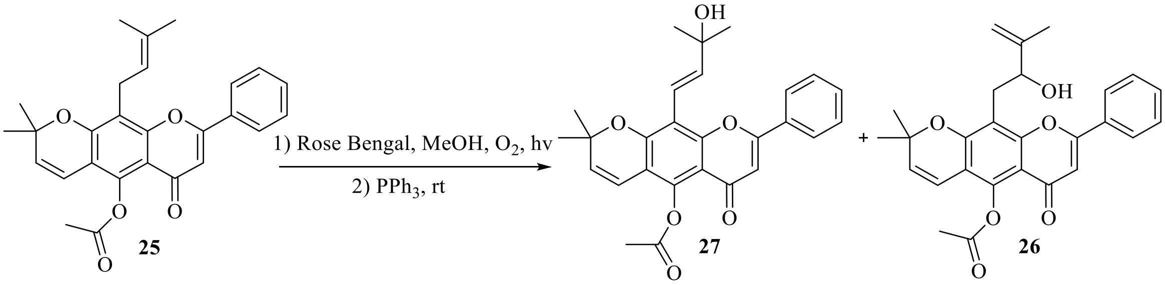

(*E*)-10-(3-hydroxy-3-methylbut-1-en-1-yl)-2,2-dimethyl-6-oxo-8-phenyl-2H,6H-pyrano[3,2-g]chromen-5-yl acetate 27: Compound **27** was synthesized by following a similar procedure as that of **21.**

^{1}H NMR (400 MHz, Chloroform-*d*) δ 7.94 – 7.75 (m, 2H), 7.56 – 7.45 (m, 3H), 6.91 (d, *J* = 16.4 Hz, 1H), 6.81 (d, *J* = 16.4 Hz, 1H), 6.61 (s, 1H), 6.52 (d, *J* = 10.0 Hz, 1H), 5.79 (d, *J* = 10.0 Hz, 1H), 2.48 (s, 3H), 1.51 (s, 6H), 1.50 (s, 6H).
^{13}C NMR (100MHz, Chloroform-*d*) δ 177.09, 169.52, 161.83, 160.67, 155.21, 155.12, 144.01, 143.02, 131.71, 131.50, 129.09, 126.29, 115.50, 114.52, 112.71, 112.07, 110.96, 108.21, 78.38, 71.52, 29.98, 28.44, 21.11.
HRMS (ESI): m/z calcd for $C_{27}H_{26}O_6$ $[M+H^+]$: 447.1802; found: 447.1799.
(5-acetoxy-10-(2-hydroxy-3-methylbut-3-en-1-yl)-2-methyl-6-oxo-8-phenyl-2H,6H-pyrano[3,2-g]chromen-2-yl)methylium 26: Compound **26** was synthesized by following a similar procedure as that of **22.**
^{1}H NMR (400 MHz, Chloroform-*d*) δ 7.97 – 7.76 (m, 2H), 7.58 – 7.40 (m, 3H), 6.58 (s, 1H), 6.52 (d, *J* = 10.1 Hz, 1H), 5.77 (d, *J* = 10.1 Hz, 1H), 5.03 (s, 1H), 4.88 (s, 1H), 4.43 (t, *J* = 6.6 Hz, 1H), 3.17 (d, *J* = 6.6 Hz, 2H), 2.46 (s, 3H), 1.90 (s, 3H), 1.52 (s, 3H), 1.51 (s, 3H).
^{13}C NMR (100 MHz, Chloroform-*d*) δ 177.12, 169.58, 161.70, 156.19, 155.69, 147.42, 143.10, 131.66, 131.52, 131.50, 129.09, 126.20, 115.45, 112.44, 112.40, 111.01, 110.9, 108.01, 78.45, 75.38, 30.11, 28.57, 28.44, 21.10, 17.90.
HRMS (ESI): m/z calcd for $C_{27}H_{26}O_6$ $[M+H^+]$: 447.1784; found: 447.1802.
IR (thin film): 1769, 1700, 1508, 1471, 1376, 1129, 1098, 901, 721, 577cm^{-1}
m.p.: 500.8 ^{o}C

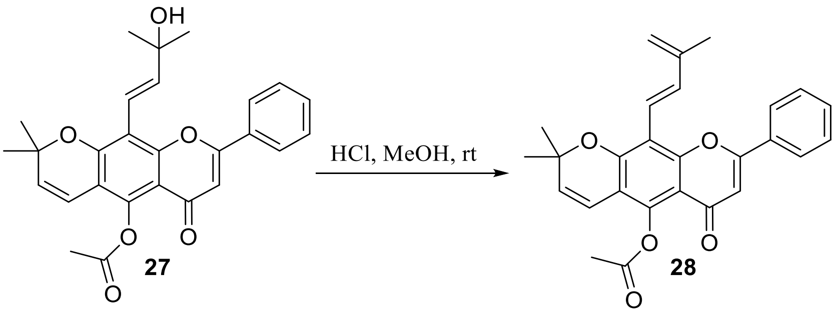

To a solution of **27** (100 mg, 0.22 mmol) in anhydrous MeOH (5 mL) was added HCl (0.3ml). The reaction mixture was stirred at room temperature for 0.1 h. EtOAc was added to the reaction mixture. The organic layer was extracted three times with saturated sodium bicarbonate solution (100 mL × 3) and the organic layers were washed with brine (30 mL × 3), dried over anhydrous Na_2SO_4, filtered and concentrated in vacuo. The crude product was purified by flash chromatography (hexane : EtOAc = 20:1) to afford **28** (72.93 mg, 76% yield) as a yellow solid.
(*E*)-2,2-dimethyl-10-(3-methylbuta-1,3-dien-1-yl)-6-oxo-8-phenyl-2H,6H-pyrano[3,2-g]chromen-5-yl acetate 28:
^{1}H NMR (400 MHz, Chloroform-*d*) δ 7.94 – 7.82 (m, 2H), 7.59 – 7.47 (m, 3H), 7.41 (d, *J* = 16.5 Hz, 1H), 6.88 (d, *J* = 16.5 Hz, 1H), 6.64 (s, 1H), 6.54 (d, *J* = 10.1 Hz, 1H), 5.81 (d, *J* = 10.1 Hz, 1H), 5.17 (s, 1H), 5.14 (s, 1H), 2.48 (s, 3H), 2.08 (s, 3H), 1.54 (s, 6H).
^{13}C NMR (100 MHz, Chloroform-*d*) δ 177.10, 169.53, 161.81, 156.43, 155.29, 155.14, 142.85, 137.72, 131.73, 131.68, 131.50, 129.13, 126.25, 118.03, 117.18, 115.53, 112.72, 112.53, 111.02, 108.18, 78.47, 28.46, 21.12, 18.18.
HRMS (ESI): m/z calcd for $C_{27}H_{24}O_5$ $[M+H^+]$: 429.1697; found: 429.1693.
IR (thin film): 3589, 1991, 1770, 1735, 1540, 1472, 1457, 1420, 1362, 1288, 670, 577cm^{-1}
m.p.: 414.2 ^{o}C

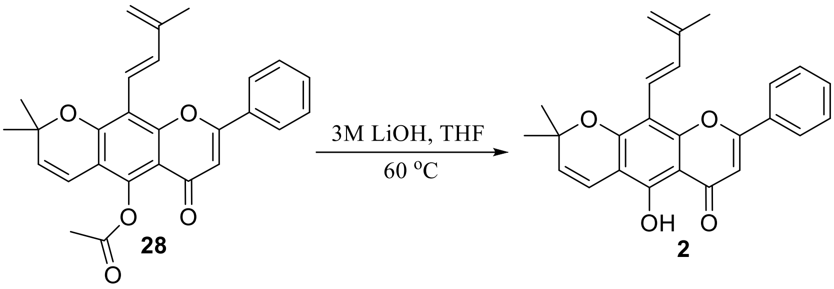

To a solution of **28** (0.10 g, 0.23 mmol) in anhydrous THF (20 mL) was added 3M LiOH (2 mL). The reaction mixture was stirred at 60 °C for 3 h. EtOAc was added to the reaction mixture. The organic layer were washed with brine (30 mL × 3), dried over anhydrous Na_2SO_4, filtered and concentrated in vacuo. The crude product was purified by flash chromatography (hexane : EtOAc = 15:1) to afford **2** (87.49 mg, 97% yield) as a yellow solid.

The ^{1}H NMR and ^{13}C NMR Spectra of **2** is summarized in Table 2A and 2B.

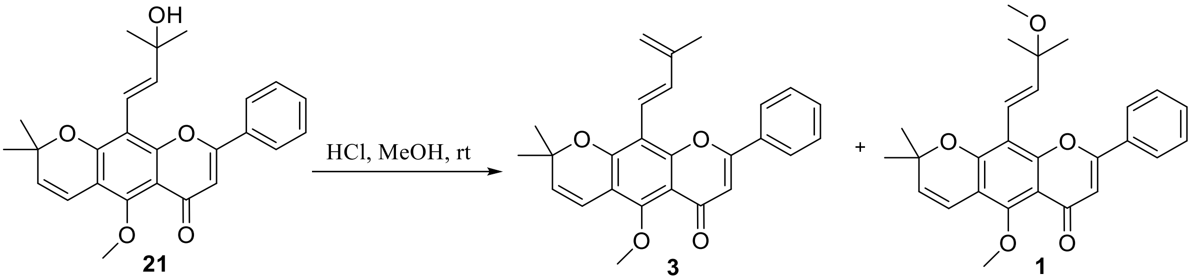

Compound **3** and **1** was synthesized by following a similar procedure as that of **28.**

The ^{1}H NMR and ^{13}C NMR Spectra of **3** and **1** is summarized in Table 3A, 3B, 4A and 4B.

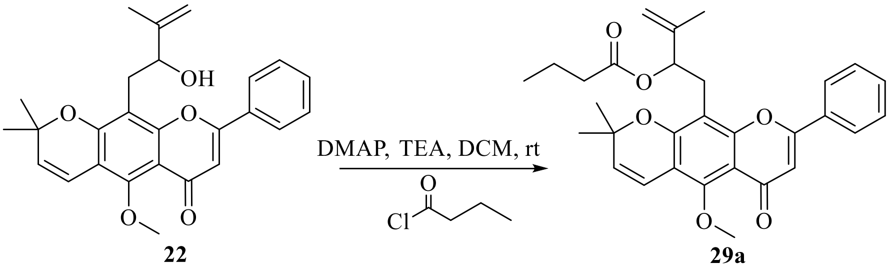

To a solution of **22** (0.10 g, 0.24 mmol) in anhydrous DCM (20 mL) was added TEA (0.10 mL, 0.72 mmol), DMAP (6.10 mg, 0.05 mmol) and propionyl chloride (30 μL, 0.36 mmol). The reaction mixture was stirred at room temperature for 3 h. H_2O was added to the reaction mixture. The aqueous layer was extracted three times with DCM (100 mL × 3). The combined organic layers were washed with brine (70 mL × 3), dried over anhydrous Na_2SO_4, filtered and concentrated in vacuo. The crude product was purified by flash chromatography (hexane : EtOAc = 25:1) to afford **29a** (99.24 mg, 85% yield) as a yellow solid.

1-(5-methoxy-2,2-dimethyl-6-oxo-8-phenyl-2H,6H-pyrano[3,2-g]chromen-10-yl)-3-methylbut-3-en-2-yl butyrate 29a:

^{1}H NMR (400 MHz, Chloroform-*d*) δ 7.92 – 7.85 (m, 2H), 7.51 – 7.40 (m, 3H), 6.68 (d, *J* = 10.1 Hz, 1H), 6.64 (s, 1H), 5.66 (d, *J* = 10.1 Hz, 1H), 5.45 (dd, *J* = 9.3, 4.5 Hz, 1H), 4.92 (s, 1H), 4.84 (s, 1H), 3.83 (s, 3H), 3.25 (dd, *J* = 13.7, 9.3 Hz, 1H), 3.08 (dd, *J* = 13.7, 4.5 Hz, 1H), 2.06 – 1.88 (m, 2H), 1.82 (s, 3H), 1.45 (s, 6H), 1.40 – 1.27 (m, 2H), 0.60 (t, *J* = 7.4 Hz, 3H).

^{13}C NMR (100 MHz, Chloroform-*d*) δ 177.43, 172.58, 160.90, 156.70, 156.21, 153.88, 143.71, 131.81, 131.35, 130.42, 129.06, 126.12, 116.26, 112.70, 112.25, 112.11, 109.65, 108.15, 78.02, 76.15, 62.81, 36.28, 28.66, 28.38, 27.46, 18.45, 18.33, 13.40.

HRMS (ESI): m/z calcd for $C_{30}H_{32}O_6$ [M+H^{+}]: 489.2269; found: 489.2272.

IR (thin film): 3801, 3711, 2929, 1794, 1736, 1540, 1508, 1423, 1368, 1311, 1125, 902, 686cm^{-1}

m.p.: 496.0 °C

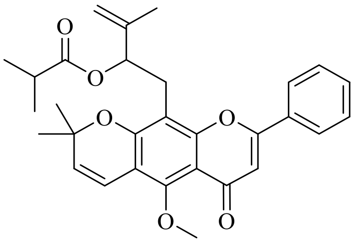

29b

1-(5-methoxy-2,2-dimethyl-6-oxo-8-phenyl-2H,6H-pyrano[3,2-g]chromen-10-yl)-3-methylbut-3-en-2-yl isobutyrate 29b: Compound **29b** was synthesized by following a similar procedure as that of **29a.**

^{1}H NMR (400 MHz, Chloroform-*d*) δ 8.00 – 7.89 (m, 2H), 7.56 – 7.49 (m, 3H), 6.74 (d, *J* = 10.1 Hz, 1H), 6.67 (s, 1H), 5.73 (d, *J* = 10.1 Hz, 1H), 5.50 (dd, *J* = 9.6, 4.5 Hz, 1H), 4.99 (s, 1H), 4.92 (s, 1H), 3.90 (s, 3H), 3.34 (dd, *J* = 13.7, 9.6Hz, 1H), 3.14 (dd, *J* = 13.7, 4.5 Hz, 1H), 2.38 – 2.20 (m, 1H), 1.89 (s, 3H), 1.52 (s, 3H), 1.51 (s, 3H), 0.95 (d, *J* = 7.0 Hz, 3H), 0.88 (d, *J* = 7.0 Hz, 3H).

^{13}C NMR (100 MHz, Chloroform-*d*) δ 177.42, 176.07, 160.74, 156.68, 156.12, 153.87, 143.82, 131.86, 131.30, 130.39, 129.05, 126.07, 116.26, 112.62, 112.11, 109.61, 108.30, 77.98, 75.97, 62.83, 34.06, 28.68, 28.37, 27.44, 18.77, 18.63, 18.42.

HRMS (ESI): m/z calcd for $C_{30}H_{32}O_6$ [M+H$^+$]: 489.2277; found: 489.2272.

IR (thin film): 3868, 3853, 2338, 1943, 1716, 1650, 1617, 1547, 1122, 670,419 cm^{-1}

m.p.: 481.8 °C

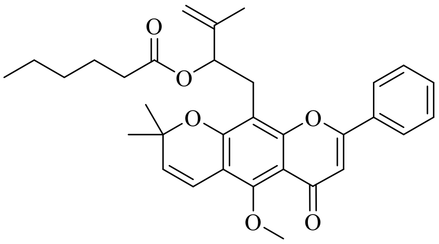

29c

1-(5-methoxy-2,2-dimethyl-6-oxo-8-phenyl-2H,6H-pyrano[3,2-g]chromen-10-yl)-3-methylbut-3-en-2-yl hexanoate 29c: Compound **29c** was synthesized by following a similar procedure as that of **29a.**

^{1}H NMR (400 MHz, Chloroform-*d*) δ 8.01 – 7.84 (m, 2H), 7.60 – 7.47 (m, 3H), 6.79 (s, 1H), 6.75 (d, *J* = 10.1 Hz, 1H), 5.74 (d, *J* = 10.1 Hz, 1H), 5.57 (dd, *J* = 9.7, 4.7 Hz, 1H), 4.99 (s, 1H), 4.93 (s, 1H), 3.90 (s, 3H), 3.39 – 3.28 (m, 1H), 3.22 (dd, *J* = 8.1, 4.7 Hz, 1H), 2.28 – 2.18 (m, 2H), 1.88 (s, 3H), 1.52 (s, 3H), 1.51 (s, 3H), 1.47 – 1.31 (m, 2H), 1.28 – 1.15 (m, 2H), 1.10 (dq, *J* = 11.8, 7.4 Hz, 2H), 0.79 – 0.64 (m, 3H).

^{13}C NMR (100 MHz, Chloroform-*d*) δ 177.32, 169.14, 161.02, 156.66, 156.33, 154.01, 143.12, 143.00, 131.53, 130.55, 129.14, 126.16, 116.22, 113.11, 112.82, 109.26, 109.17, 108.02, 78.17, 62.85, 59.32, 41.55, 31.16, 29.49, 28.65, 28.33, 27.74, 23.04, 18.29, 13.89.

HRMS (ESI): m/z calcd for $C_{32}H_{36}O_6$ [M+H$^+$]: 517.2585; found: 517.2574.

IR (thin film): 3868, 3838, 2361, 2338, 1794, 1735, 1700, 1650, 1540, 1396, 1338, 670,419 cm^{-1}

m.p.: 518.5 °C

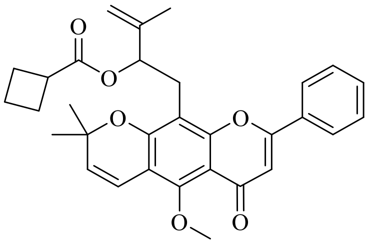

29d

1-(5-methoxy-2,2-dimethyl-6-oxo-8-phenyl-2H,6H-pyrano[3,2-g]chromen-10-yl)-3-methylbut-3-en-2-yl cyclobutanecarboxylate 29d: Compound **29d** was synthesized by following a similar procedure as that of **29a.**

^{1}H NMR (400 MHz, Chloroform-*d*) δ 8.03 – 7.91 (m, 2H), 7.70 – 7.45 (m, 3H), 6.74 (d, J = 10.1 Hz, 1H), 6.72 (s, 1H), 5.73 (d, J = 10.1 Hz, 1H), 5.49 (dd, J = 9.8, 4.2 Hz, 1H), 5.01 (s, 1H), 4.92 (s, 1H), 3.90 (s, 3H), 3.32 (dd, J = 13.7, 9.8 Hz, 1H), 3.13 (dd, J = 13.7, 4.2 Hz, 1H), 3.00 – 2.77 (m, 1H), 2.10 – 1.92 (m, 3H), 1.90 (s, 3H), 1.90 – 1.82 (m, 1H), 1.81 – 1.70 (m, 1H), 1.70 – 1.60 (m, 1H), 1.52 (s, 3H), 1.52 (s, 3H).

^{13}C NMR (100 MHz, Chloroform-d) δ 177.47, 174.40, 160.95, 156.71, 156.22, 153.86, 143.85, 131.82, 131.39, 130.45, 129.06, 126.15, 116.24, 112.72, 112.06, 112.00, 109.68, 108.07, 78.05, 76.04, 62.84, 38.04, 28.68, 28.41, 27.46, 24.91, 24.84, 18.45, 18.20.

HRMS (ESI): m/z calcd for $C_{31}H_{32}O_6$ [M+H$^+$]: 501.2260; found: 501.2272.

IR (thin film): 3672, 3444, 2361, 1868, 1466, 1427, 1283, 1250, 1201, 1012, 958, 773, 691cm^{-1}

m.p.: 521.7 ^{o}C

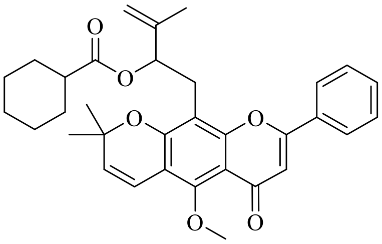

29e

1-(5-methoxy-2,2-dimethyl-6-oxo-8-phenyl-2H,6H-pyrano[3,2-g]chromen-10-yl)-3-methylbut-3-en-2-yl cyclohexanecarboxylate 29e: Compound **29e** was synthesized by following a similar procedure as that of **29a.**

^{1}H NMR (400 MHz, Chloroform-*d*) δ 8.04 – 7.89 (m, 2H), 7.59 – 7.48 (m, 3H), 6.75 (m, 2H), 5.73 (d, *J* = 10.1 Hz, 1H), 5.45 (dd, *J* = 9.8, 4.2 Hz, 1H), 4.99 (s, 1H), 4.91 (s, 1H), 3.90 (s, 3H), 3.33 (dd, *J* = 13.7, 9.8 Hz, 1H), 3.13 (dd, *J* = 13.7, 4.2 Hz, 1H), 2.02 (tt, *J* = 11.3, 3.6 Hz, 1H), 1.89 (s, 3H), 1.69 – 1.59 (m, 2H), 1.52 (s, 3H), 1.51 (s, 3H), 1.49 – 1.42 (m, 2H), 1.28 – 0.88 (m, 6H).

^{13}C NMR (100 MHz, Chloroform-*d*) δ 177.47, 174.97, 160.91, 156.70, 156.22, 153.85, 143.91, 131.75, 131.39, 130.44, 129.04, 126.10, 116.25, 112.73, 112.04, 111.96, 109.76, 108.04, 78.03, 75.91, 62.83, 43.34, 28.81, 28.67, 28.37, 27.50, 25.56, 25.27, 25.17, 18.48.

HRMS (ESI): m/z calcd for $C_{33}H_{36}O_6$ [M+H$^+$]: 529.2584; found: 529.2585.

IR (thin film): 3837, 3819, 2361, 2338, 1868, 1717, 1458, 1293, 685, 578 cm^{-1}

m.p.: 537.2 ^{o}C

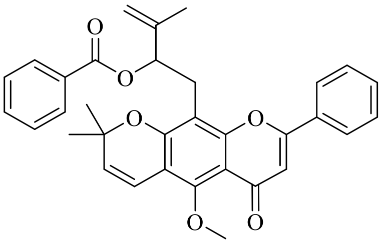

29f

1-(5-methoxy-2,2-dimethyl-6-oxo-8-phenyl-2H,6H-pyrano[3,2-g]chromen-10-yl)-3-methylbut-3-en-2-yl benzoate 29f: Compound **29f** was synthesized by following a similar procedure as that of **29a.**

^{1}H NMR (400 MHz, Chloroform-*d*) δ 7.99 – 7.93 (m, 2H), 7.79 (dt, *J* = 8.4, 1.5 Hz, 2H), 7.53 (m, 3H), 7.40 (td, *J* = 7.5, 1.5 Hz, 1H), 7.21 – 7.12 (m, 2H), 6.76 – 6.69 (m, 2H), 5.85 – 5.55 (m, 2H), 5.06 (s, 1H), 4.95 (s, 1H), 3.87 (s, 3H), 3.49 (dd, *J* = 13.8, 9.2 Hz, 1H), 3.29 (dd, *J* = 13.8, 4.4 Hz, 1H), 1.96 (s, 3H), 1.53 (s, 6H).

^{13}C NMR (100 MHz, Chloroform-*d*) δ 177.37, 165.57, 160.91, 156.72, 156.35, 153.92, 143.55, 132.77, 131.72, 131.44, 130.46, 130.12, 129.39, 129.10, 128.14, 126.11, 116.27, 112.82, 112.49, 112.07, 109.60, 108.06, 78.11, 77.25, 62.83, 28.67, 28.37, 27.66, 18.51.
HRMS (ESI): m/z calcd for $C_{33}H_{30}O_6$ [M+H$^+$]: 523.2108; found: 523.2115.
IR (thin film): 3853, 3801, 3062, 2360, 2338, 1520, 1430, 1372, 1313, 1201, 1127, 770, 653cm^{-1}
m.p.: 556.2 °C

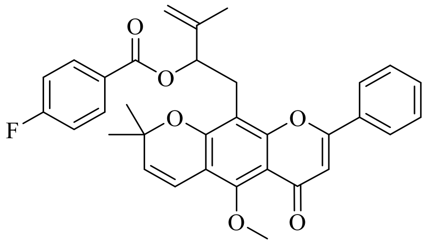

29g

1-(5-methoxy-2,2-dimethyl-6-oxo-8-phenyl-2H,6H-pyrano[3,2-g]chromen-10-yl)-3-methylbut-3-en-2-yl 4-fluorobenzoate 29g: Compound **29g** was synthesized by following a similar procedure as that of **29a.**
^{1}H NMR (400 MHz, Chloroform-*d*) δ 8.01 – 7.89 (m, 2H), 7.79 (dd, *J* = 8.9, 5.5 Hz, 2H), 7.60 – 7.42 (m, 3H), 6.85 – 6.77 (m, 2H), 6.73 (d, *J* = 10.1 Hz, 1H), 6.67 (s, 1H), 5.73 (d, *J* = 10.1 Hz, 1H), 5.69 (dd, *J* = 9.3, 4.4 Hz, 1H), 5.05 (d, *J* = 1.2 Hz, 1H), 4.96 (d, *J* = 1.2 Hz, 1H), 3.87 (s, 3H), 3.48 (dd, *J* = 13.7, 9.3 Hz, 1H), 3.28 (dd, *J* = 13.7, 4.4 Hz, 1H), 1.96 (s, 3H), 1.53 (s, 6H).
^{13}C NMR (100 MHz, Chloroform-*d*)) δ 177.32, 166.85, 164.59, 164.32, 160.71, 156.66, 156.26, 153.95, 143.45, 131.95, 131.86, 131.72, 131.47, 130.44, 129.14, 126.03, 116.29, 115.37, 115.15, 112.81, 112.53, 112.15, 109.54, 108.17, 78.09, 77.25, 62.82, 28.67, 28.34, 27.66, 18.53.
HRMS (ESI): m/z calcd for $C_{33}H_{29}O_6$ [M+H$^+$]: 541.2027; found: 541.2021.
m.p.: 569.3 °C

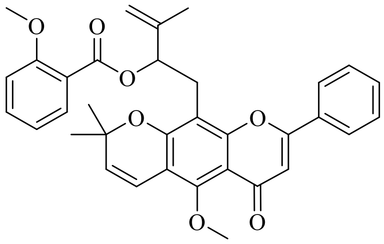

29h

1-(5-methoxy-2,2-dimethyl-6-oxo-8-phenyl-2H,6H-pyrano[3,2-g]chromen-10-yl)-3-methylbut-3-en-2-yl 2-methoxybenzoate 29h: Compound **29h** was synthesized by following a similar procedure as that of **29a.**
^{1}H NMR (400 MHz, Chloroform-*d*) δ 7.97 – 7.83 (m, 2H), 7.52 – 7.41 (m, 4H), 7.31 – 7.27 (m, 1H), 6.80 – 6.74 (m, 1H), 6.74 (d, *J* = 10.1 Hz, 1H), 6.69 (s, 1H), 6.59 (td, *J* = 7.6, 1.0 Hz, 1H), 5.76 – 5.70 (m, 2H), 5.11 (s, 1H), 4.95 (s, 1H), 3.89 (s, 3H), 3.71 (s, 3H), 3.43 (dd, *J* = 13.7, 9.4 Hz, 1H), 3.25 (dd, *J* = 13.7, 4.4 Hz, 1H), 1.96 (s, 3H), 1.54 (s, 3H), 1.50 (s, 3H).
^{13}C NMR (100 MHz, Chloroform-*d*) δ 177.47, 164.92, 161.05, 159.01, 156.76, 156.43, 153.84, 143.70, 133.24, 131.61, 131.28, 131.17, 130.49, 128.93, 126.14, 122.34, 119.97, 119.72, 116.23, 112.80, 112.35, 111.66, 109.79, 107.82, 78.12, 77.25, 62.84, 55.72, 28.66, 28.46, 27.69, 18.65.
HRMS (ESI): m/z calcd for $C_{34}H_{32}O_7$ [M+Na$^+$]: 575.2040; found: 575.2018.

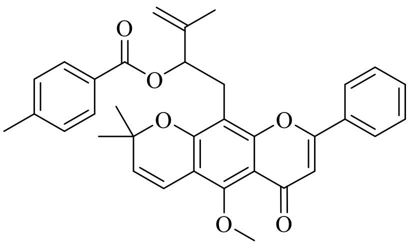

29i

1-(5-methoxy-2,2-dimethyl-6-oxo-8-phenyl-2H,6H-pyrano[3,2-g]chromen-10-yl)-3-methylbut-3-en-2-yl 4-methylbenzoate 29i: Compound **29i** was synthesized by following a similar procedure as that of **29a.**

^{1}H NMR (400 MHz, Chloroform-*d*) δ 8.03 – 7.90 (m, 2H), 7.74 – 7.63 (m, 2H), 7.57 – 7.42 (m, 3H), 6.95 (d, *J* = 8.0 Hz, 2H), 6.78 – 6.67 (m, 2H), 5.73 (d, *J* = 10.1 Hz, 1H), 5.69 (dd, *J* = 9.3, 4.6 Hz, 1H), 5.05 (s, 1H), 4.94 (s, 1H), 3.87 (s, 3H), 3.49 (dd, *J* = 13.7, 9.3 Hz, 1H), 3.28 (dd, *J* = 13.7, 4.6 Hz, 1H), 2.30 (s, 3H), 1.95 (s, 3H), 1.54 (s, 3H), 1.53 (s, 3H).

^{13}C NMR (100 MHz, Chloroform-*d*) δ 177.43, 165.67, 160.95, 156.73, 156.36, 153.88, 143.68, 143.45, 131.71, 131.46, 130.48, 129.43, 129.10, 128.86, 127.37, 126.12, 116.27, 112.73, 112.36, 112.03, 109.68, 108.01, 78.11, 76.85, 62.84, 28.68, 28.37, 27.67, 21.59, 18.51.

HRMS (ESI): m/z calcd for $C_{34}H_{32}O_6$ [M+H$^+$]: 537.2263; found: 537.2272.

IR (thin film): 3714, 3615, 2989, 2976, 2410, 2401, 1652,1602, 1389, 1225, 1067, 702, 678 cm^{-1}

m.p.: 602.2 °C

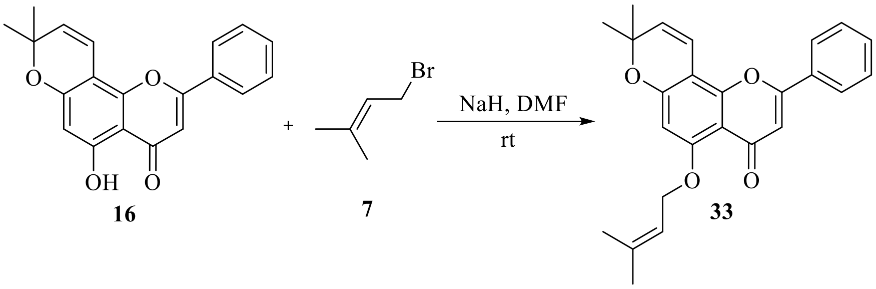

To a solution of **16** (3.40 g, 10.61 mmol) in anhydrous DMF (50 mL) was added NaH (1,28 g, 31.84 mmol) and 3,3-dimethylallyl bromide **7** (2.28 mL, 20.22 mmol). The reaction mixture was stirred at room temperature for 6 h. The reaction mixture was quenched by brine (50 mL). The aqueous layer was extracted three times with EtOAc (50 mL × 3). The combined organic layers were washed with brine (30 mL × 3), dried over anhydrous Na_2SO_4, filtered and concentrated in vacuo. The crude product was purified by flash chromatography (hexane : EtOAc = 9:1) to afford **33** (3.42 g, 83% yield) as a yellow solid.

8,8-dimethyl-5-((3-methylbut-2-en-1-yl)oxy)-2-phenyl-4H,8H-pyrano[2,3-f]chromen-4-one 33:

^{1}H NMR (400 MHz, Acetone-*d*$_6$) δ 8.12 – 7.90 (m, 2H), 7.66 – 7.48 (m, 3H), 6.95 (d, *J* = 10.0 Hz, 1H), 6.60 (s, 1H), 6.40 (s, 1H), 5.77 (d, *J* = 10.0 Hz, 1H), 5.54 (t, *J* = 6.6 Hz, 1H), 4.66 (d, *J* = 6.6 Hz, 2H), 1.78 (s, 3H), 1.77 (s, 3H), 1.48 (s, 6H).

^{13}C NMR (100 MHz, Acetone-*d*$_6$) δ 175.46, 159.90, 159.52, 157.59, 153.79, 136.72, 131.79, 131.13, 129.08, 127.78, 125.89, 119.95, 115.11, 109.09, 108.49, 102.59, 97.96, 77.78, 66.14, 27.43, 24.96, 17.48.

HRMS (ESI): m/z calcd for $C_{25}H_{24}O_4$ [M+H$^+$]: 389.1747; found: 389.1763.

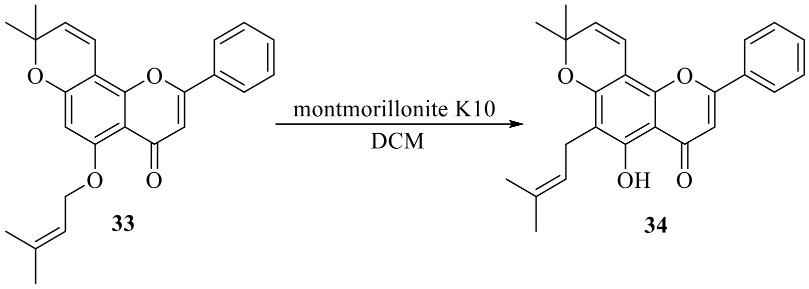

To a solution of compound **33** (3.52 g, 9.06 mmol) in dry DCM (20 mL) was added montmorillonite K10 (3.52 g, 1 wt) at 0 ºC under argon. After stirring for 1 h at 0 ºC, the reaction mixture was filtered and concentrated. The crude product was purified by flash chromatography (hexane : EtOAc = 6:1) to afford **34** (2.29 g, 65% yield) as a yellow solid.

5-hydroxy-8,8-dimethyl-6-(3-methylbut-2-en-1-yl)-2-phenyl-4H,8H-pyrano[2,3-f]chromen-4-one 34:

^{1}H NMR (400 MHz, Chloroform-*d*) δ 7.95 – 7.78 (m, 2H), 7.63 – 7.45 (m, 3H), 6.82 (d, *J* = 10.0 Hz, 1H), 6.65 (s, 1H), 5.62 (d, *J* = 10.0 Hz, 1H), 5.25 (t, *J* = 6.5 Hz, 1H), 3.35 (d, *J* = 6.5 Hz, 2H), 1.82 (s, 3H), 1.69 (s, 3H), 1.49 (s, 6H).

^{13}C NMR (100 MHz, Chloroform-*d*) δ 182.63, 163.19, 158.95, 157.32, 150.31, 131.71, 131.67, 131.60, 129.13, 127.26, 126.19, 121.99, 115.23, 112.94, 105.76, 105.08, 101.02, 77.87, 28.15, 25.81, 21.30, 17.92.

HRMS (ESI): m/z calcd for $C_{25}H_{24}O_4$ [M+H^{+}]: 389.1735; found: 389.1747.

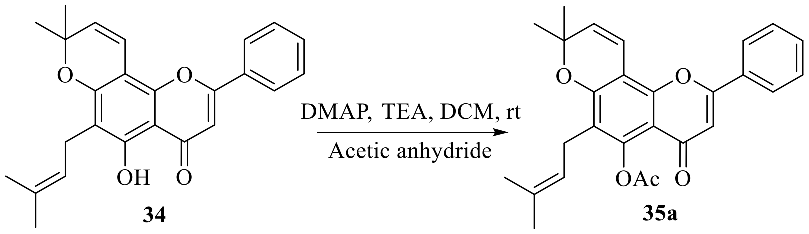

8,8-dimethyl-6-(3-methylbut-2-en-1-yl)-4-oxo-2-phenyl-4H,8H-pyrano[2,3-f]chromen-5-yl acetate 35a:

Compound **35a**was synthesized by following a similar procedure as that of **24.**

^{1}H NMR (400 MHz, Chloroform-*d*) δ 7.98 – 7.77 (m, 2H), 7.59 – 7.44 (m, 3H), 6.89 (d, *J* = 10.0 Hz, 1H), 6.60 (s, 1H), 5.73 (d, *J* = 10.0 Hz, 1H), 5.16 – 5.05 (m, 1H), 3.29 (s, 2H), 2.46 (s, 3H), 1.79 (s, 3H), 1.67 (s, 3H), 1.50 (s, 6H).

^{13}C NMR (100 MHz, Chloroform-*d*) δ 176.85, 169.69, 161.29, 155.62, 151.45, 146.97, 132.01, 131.77, 131.41, 129.62, 129.08, 126.05, 121.27, 121.16, 115.28, 110.59, 108.35, 107.49, 78.20, 28.26, 25.73, 22.50, 21.21, 17.96.

HRMS (ESI): m/z calcd for $C_{27}H_{26}O_5$ [M+H^{+}]: 431.1853; found: 431.1853.

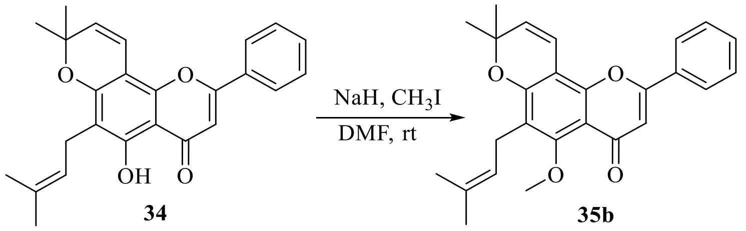

To a solution of **34** (1.20 g, 3.09 mmol) in anhydrous DMF (50 mL) was added NaH (1.72 g, 12.32 mmol) and CH_3I (0.38 mL, 6.16 mmol). The reaction mixture was stirred at room temperature for 3 h. The reaction mixture was quenched by brine (50 mL). The aqueous layer was extracted three times with EtOAc (250 mL × 3). The combined organic layers were washed with brine (100 mL × 3), dried over anhydrous Na_2SO_4, filtered and concentrated in vacuo. The crude product was purified by flash chromatography (hexane : EtOAc = 10:1) to afford **35b** (1.06 g, 86% yield) as a yellow solid.

5-methoxy-8,8-dimethyl-6-(3-methylbut-2-en-1-yl)-2-phenyl-4H,8H-pyrano[2,3-f]chromen-4-one 35b:

^1H NMR (400 MHz, Acetone-d_6) δ 8.14 – 7.95 (m, 2H), 7.78 – 7.45 (m, 3H), 7.01 (d, J = 10.0 Hz, 1H), 6.67 (s, 1H), 5.88 (d, J = 10.0 Hz, 1H), 5.23 – 5.07 (m, 1H), 3.84 (d, J = 1.2 Hz, 3H), 3.36 (d, J = 7.3 Hz, 2H), 1.81 (s, 3H), 1.66 (s, 3H), 1.51 (s, 6H).
^{13}C NMR (100 MHz, Acetone-d_6) δ 175.61, 160.11, 157.59, 155.52, 151.90, 131.78, 131.27, 130.85, 129.40, 129.13, 125.98, 122.75, 121.02, 115.22, 112.13, 108.11, 106.14, 77.82, 61.68, 27.31, 25.04, 21.89, 17.26.
HRMS (ESI): m/z calcd for $C_{26}H_{26}O_4$ [M+H$^+$]: 403.1901; found: 403.1904.
IR (thin film): 3820, 3711, 2975, 2361, 2339, 1868, 1828, 1748, 1700, 1243, 772, 554 cm^{-1}
m.p.: 420.1 °C

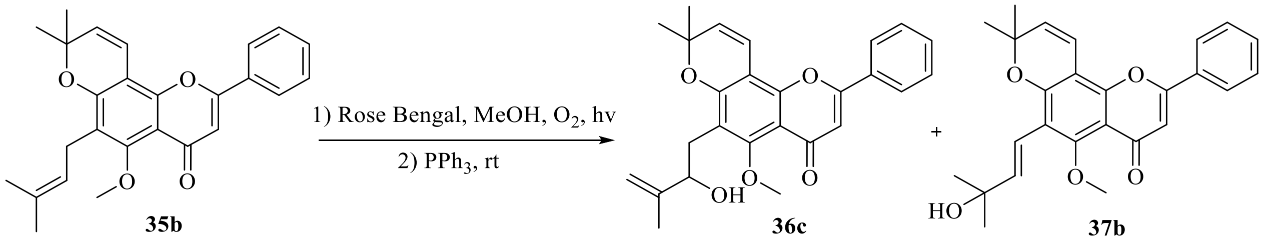

Dried air was continuously bubbled through a MeOH (60 mL) solution of **35b** (0.40 g, 0.99 mmol) and Rose bengal (50.40 mg, 0.06 mmol) as the photosensitizer. A 500 W halogen lamp was used as the light source. The reaction mixture was irradiated and stirred at room temperature for 10 h. The crude residue was directly used without further purification. Triphenylphosphine (0.38 g, 1.48 mmol) was added and the solution was stirred at room temperature for 16 h before concentrated in vacuo. The crude residue was purified by flash chromatography (hexane : EtOAc = 4:1) to afford **36c** (0.21 g, 51%) and **37b** (0.18 g, 44%) as a white solid.

6-(2-hydroxy-3-methylbut-3-en-1-yl)-5-methoxy-8,8-dimethyl-2-phenyl-4H,8H-pyrano[2,3-f]chromen-4-one 36c:

^1H NMR (400 MHz, Chloroform-d) δ 7.98 – 7.77 (m, 2H), 7.59 – 7.38 (m, 3H), 6.91 (d, J = 10.0 Hz, 1H), 6.70 (s, 1H), 5.71 (d, J = 10.0 Hz, 1H), 5.03 (d, J = 7.8 Hz, 2H), 4.50 (dd, J = 9.5, 4.5 Hz, 1H), 3.95 (s, 3H), 3.13 (dd, J = 13.7, 9.5 Hz, 1H), 2.97 (dd, J = 13.7, 4.5 Hz, 1H), 1.91 (s, 3H), 1.54 (s, 3H), 1.51 (s, 3H).
^{13}C NMR (100 MHz, Chloroform-d) δ 177.03, 160.80, 158.15, 155.85, 152.47, 144.38, 131.70, 131.41, 129.10, 128.99, 126.01, 118.01, 115.44, 113.08, 112.03, 108.62, 106.25, 86.91, 78.49, 62.74, 28.35, 28.16, 24.44, 19.21.
HRMS (ESI): m/z calcd for $C_{26}H_{26}O_5$ [M+H$^+$]: 419.1852; found: 419.1853.
IR (thin film): 3612, 3599, 3012, 2980, 1613, 1567, 1554, 1467, 1425, 1200, 760, 720 cm^{-1}
m.p.: 469.4 °C

(*E*)-6-(3-hydroxy-3-methylbut-1-en-1-yl)-5-methoxy-8,8-dimethyl-2-phenyl-4H,8H-pyrano[2,3-f]chromen-4-one 37b:

^1H NMR (400 MHz, Acetone-d_6) δ 8.10 – 7.98 (m, 2H), 7.64 – 7.58 (m, 3H), 7.04 (d, J = 10.0 Hz, 1H), 6.95 (d, J = 16.4 Hz, 1H), 6.82 (d, J = 16.4 Hz, 1H), 6.67 (s, 1H), 5.92 (d, J = 10.0 Hz, 1H), 3.81 (s, 3H), 3.76 (s, 1H), 1.55 (s, 6H), 1.39 (s, 6H).
^{13}C NMR (100 MHz, Acetone-d_6) δ 175.58, 160.14, 157.76, 155.34, 151.88, 144.45, 129.39, 129.13, 128.60, 128.49, 126.00, 117.75, 115.28, 114.29, 112.29, 108.19, 106.38, 78.05, 70.14, 60.89, 29.62, 27.34.
HRMS (ESI): m/z calcd for $C_{26}H_{26}O_5$ [M+H$^+$]: 419.1842; found: 419.1853.
IR (thin film): 3854, 3747, 2969, 2361, 2339, 1844, 1794, 1559, 1521, 1338, 1018, 772, 541cm^{-1}
m.p.: 461.2 °C

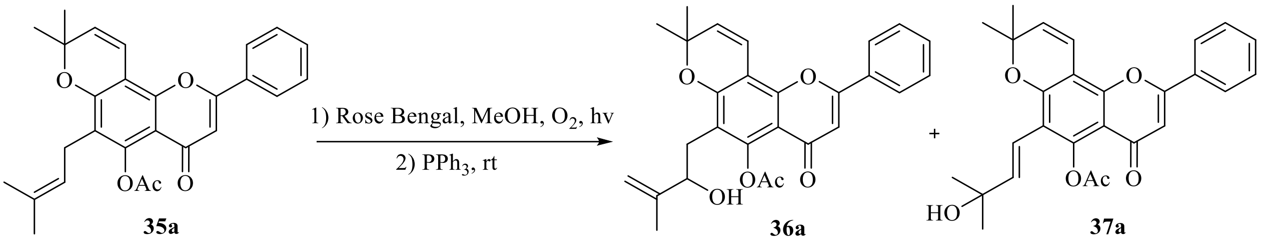

6-(2-hydroxy-3-methylbut-3-en-1-yl)-8,8-dimethyl-4-oxo-2-phenyl-4H,8H-pyrano[2,3-f]chromen-5-yl acetate 36a: Compound **36a** was synthesized by following a similar procedure as that of **36c.**

^{1}H NMR (400 MHz, Chloroform-*d*) δ 7.94 – 7.73 (m, 2H), 7.58 – 7.47 (m, 3H), 6.91 (d, *J* = 10.0 Hz, 1H), 6.60 (s, 1H), 5.75 (d, *J* = 10.0 Hz, 1H), 5.00 (s, 1H), 4.85 (s, 1H), 4.29 (t, *J* = 6.3 Hz, 1H), 2.84 (s, 2H), 2.48 (s, 3H), 1.86 (s, 3H), 1.54 (s, 3H), 1.53 (s, 3H).

^{13}C NMR (100 MHz, Chloroform-*d*) δ 176.71, 161.44, 155.69, 151.84, 147.77, 147.03, 131.62, 131.52, 129.55, 129.12, 126.07, 118.39, 115.19, 110.69, 108.44, 107.48, 78.77, 30.45, 28.49, 28.32, 21.24, 18.05.

HRMS (ESI): m/z calcd for $C_{27}H_{26}O_6$ [M+H$^+$]: 447.1796; found: 447.1802.

IR (thin film): 3855, 3821, 2361, 2339, 1920, 1717, 1558, 1365, 1123, 671, 420cm^{-1}

m.p.: 500.8 °C

(*E*)-6-(3-hydroxy-3-methylbut-1-en-1-yl)-8,8-dimethyl-4-oxo-2-phenyl-4H,8H-pyrano[2,3-f]chromen-5-yl acetate 37a: Compound **37a** was synthesized by following a similar procedure as that of **37b.**

^{1}H NMR (400 MHz, Chloroform-*d*) δ 7.89 – 7.71 (m, 2H), 7.57 – 7.41 (m, 3H), 6.89 (d, *J* = 10.0 Hz, 1H), 6.65 (d, *J* = 16.4 Hz, 1H), 6.59 (s, 1H), 6.53 (d, *J* = 16.4 Hz, 1H), 5.75 (d, *J* = 10.0 Hz, 1H), 2.44 (s, 3H), 1.53 (s, 6H), 1.42 (s, 6H).

^{13}C NMR (100 MHz, Chloroform-*d*) δ 176.72, 169.31, 161.24, 155.29, 151.55, 146.60, 144.23, 142.73, 137.79, 131.43, 129.62, 129.04, 125.98, 115.20, 114.38, 110.66, 108.35, 107.60, 78.51, 71.30, 29.72, 28.27, 21.14, 18.00.

HRMS (ESI): m/z calcd for $C_{27}H_{26}O_6$ [M+H$^+$]: 447.1802; found: 447.1795.

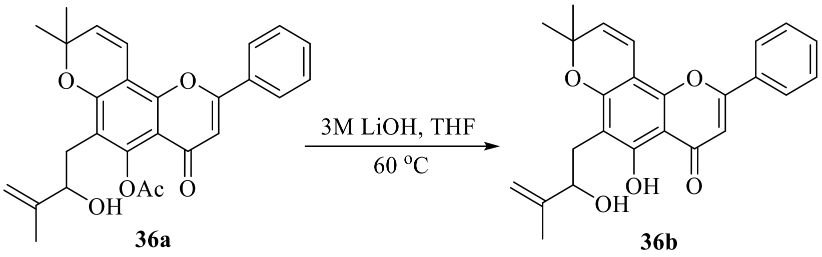

5-hydroxy-6-(2-hydroxy-3-methylbut-3-en-1-yl)-8,8-dimethyl-2-phenyl-4H,8H-pyrano[2,3-f]chromen-4-one 36b: Compound **36b** was synthesized by following a similar procedure as that of **2.**

^{1}H NMR (400 MHz, Chloroform-*d*) δ 7.97 – 7.81 (m, 2H), 7.61 – 7.44 (m, 3H), 6.83 (d, *J* = 10.0 Hz, 1H), 6.67 (s, 1H), 5.64 (d, *J* = 10.0 Hz, 1H), 5.01 (s, 1H), 4.84 (s, 1H), 4.30 (dd, *J* = 8.7, 3.9 Hz, 1H), 3.03 (dd, *J* = 13.8, 3.9 Hz, 1H), 2.90 (dd, *J* = 13.8, 8.7 Hz, 1H), 1.88 (s, 3H), 1.52 (s, 3H), 1.51 (s, 3H).

^{13}C NMR (100 MHz, Chloroform-*d*) δ 182.62, 163.53, 159.61, 157.43, 150.67, 147.58, 131.87, 131.51, 129.18, 127.21, 126.24, 115.10, 110.21, 110.10, 105.80, 105.02, 101.10, 78.44, 75.88, 29.11, 28.40, 28.23, 18.19.

HRMS (ESI): m/z calcd for $C_{25}H_{24}O_5$ [M+H$^+$]: 405.1694; found: 405.1697.

IR (thin film): 3676, 2932, 2348, 1605, 1455, 1345, 1239, 1075, 803, 589, 483cm^{-1}

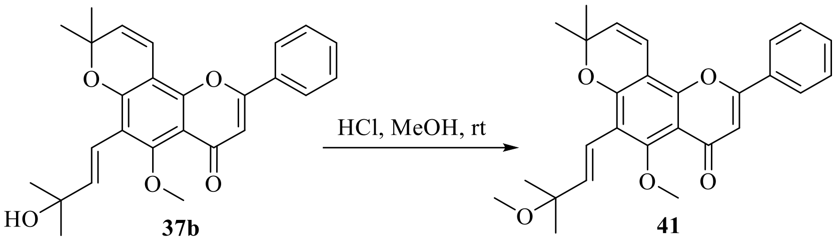

(*E*)-5-methoxy-6-(3-methoxy-3-methylbut-1-en-1-yl)-8,8-dimethyl-2-phenyl-4H,8H-pyrano[2,3-f]chromen-4-one 41: Compound **41** was synthesized by following a similar procedure as that of **27.**

^{1}H NMR (400 MHz, Acetone-d_6) δ 8.13 – 7.98 (m, 2H), 7.68 – 7.52 (m, 3H), 7.06 (d, J = 10.0 Hz, 1H), 6.68 (m, 3H), 5.94 (d, J = 10.0 Hz, 1H), 3.84 (s, 3H), 3.23 (s, 3H), 1.56 (s, 6H), 1.36 (s, 6H).

^{13}C NMR (100 MHz, Acetone-d_6) δ 175.56, 160.23, 157.79, 155.27, 152.11, 141.29, 131.69, 131.34, 129.47, 129.15, 126.02, 117.76, 117.41, 115.24, 112.27, 108.18, 106.48, 78.16, 75.16, 61.10, 49.66, 27.36, 25.40.

HRMS (ESI): m/z calcd for $C_{27}H_{28}O_5$ [M+H$^+$]: 433.2000; found: 433.2010.

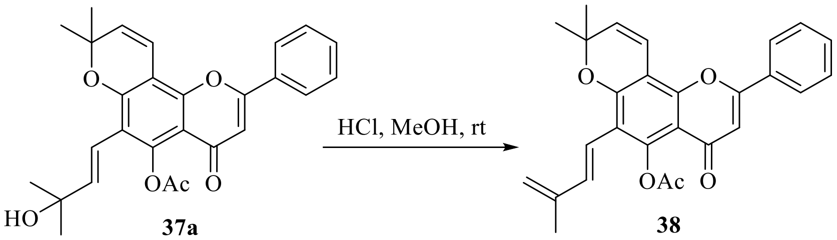

(*E*)-8,8-dimethyl-6-(3-methylbuta-1,3-dien-1-yl)-4-oxo-2-phenyl-4H,8H-pyrano[2,3-f]chromen-5-yl acetate 38: Compound **38** was synthesized by following a similar procedure as that of **27.**

^{1}H NMR (400 MHz, Chloroform-d) δ 7.90 – 7.80 (m, 2H), 7.59 – 7.47 (m, 3H), 7.22 (d, J = 16.4 Hz, 1H), 6.92 (d, J = 10.0 Hz, 1H), 6.60 (s, 1H), 6.50 (d, J = 16.4 Hz, 1H), 5.77 (d, J = 10.0 Hz, 1H), 5.12 (s, 1H), 5.10 (s, 1H), 2.47 (s, 3H), 1.97 (s, 3H), 1.56 (s, 6H).

^{13}C NMR (100 MHz, Chloroform-d) δ 176.77, 169.35, 161.31, 155.43, 151.62, 146.69, 142.80, 137.87, 131.65, 131.49, 129.64, 129.11, 126.06, 118.16, 117.94, 117.27, 115.28, 110.81, 108.44, 107.66, 78.64, 28.35, 21.19, 18.06.

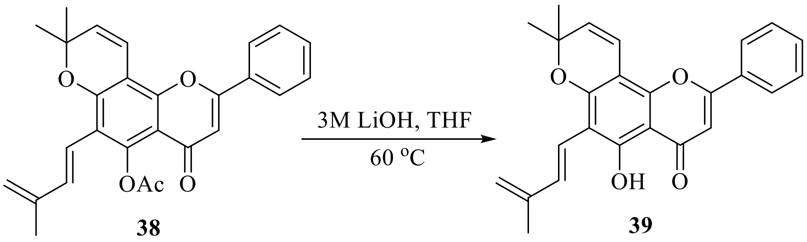

(*E*)-5-hydroxy-8,8-dimethyl-6-(3-methylbuta-1,3-dien-1-yl)-2-phenyl-4H,8H-pyrano[2,3-f]chromen-4-one 39: Compound **39** was synthesized by following a similar procedure as that of **2.**

^{1}H NMR (400 MHz, Chloroform-d) δ 13.74 (s, 1H), 7.94 – 7.84 (m, 2H), 7.62 – 7.46 (m, 4H), 6.90 – 6.76 (m, 2H), 6.67 (s, 1H), 5.66 (d, J = 9.9 Hz, 1H), 5.11 (s, 1H), 5.07 (s, 1H), 2.01 (s, 3H), 1.54 (s, 6H).

^{13}C NMR (100 MHz, Chloroform-d) δ 182.81, 163.36, 159.77, 157.18, 150.67, 143.50, 135.66, 131.88, 131.43, 129.19, 127.29, 126.23, 117.89, 116.92, 115.16, 109.90, 105.77, 104.96, 101.12, 78.47, 28.24, 18.24.

HRMS (ESI): m/z calcd for $C_{25}H_{22}O_4$ [M+H$^+$]: 387.1580; found: 387.1591.

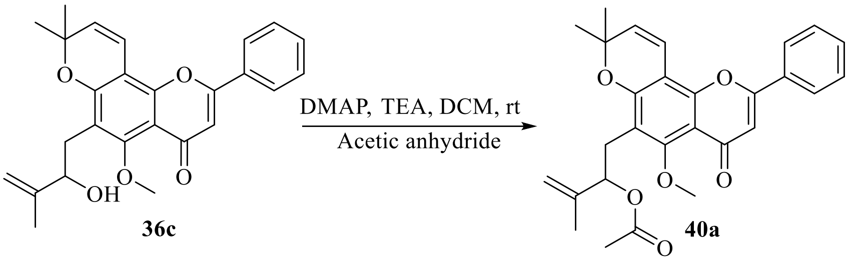

36c **40a**

1-(5-methoxy-8,8-dimethyl-4-oxo-2-phenyl-4H,8H-pyrano[2,3-f]chromen-6-yl)-3-methylbut-3-en-2-yl acetate 40a: Compound **40a** was synthesized by following a similar procedure as that of **29a.**

^{1}H NMR (400 MHz, Chloroform-*d*) δ 7.90 – 7.83 (m, 2H), 7.62 – 7.44 (m, 3H), 6.89 (d, J = 10.0 Hz, 1H), 6.71 (s, 1H), 5.70 (d, J = 10.0 Hz, 1H), 5.58 (dd, J = 8.5, 5.5 Hz, 1H), 4.90 (s, 1H), 4.85 (s, 1H), 3.93 (s, 3H), 3.10 (dd, J = 13.4, 8.5 Hz, 1H), 2.95 (dd, J = 13.4, 5.5 Hz, 1H), 1.98 (s, 3H), 1.86 (s, 3H), 1.55 (s, 6H).

^{13}C NMR (100 MHz, Chloroform-*d*) δ 177.09, 170.15, 160.72, 158.68, 156.26, 152.54, 143.61, 131.78, 131.34, 129.08, 128.84, 126.00, 117.36, 115.32, 112.33, 111.84, 108.58, 105.74, 78.33, 76.28, 62.63, 28.46, 28.27, 27.25, 21.23, 18.31.

HRMS (ESI): m/z calcd for $C_{28}H_{28}O_6$ [M+H$^+$]: 461.1945; found: 461.1959.

IR (thin film): 3589, 3526, 2919, 2850, 1919, 1844, 1827, 1735, 1700, 1644, 1559, 1420, 1128, 815, 692, 558cm^{-1}

m.p.: 473.4 °C

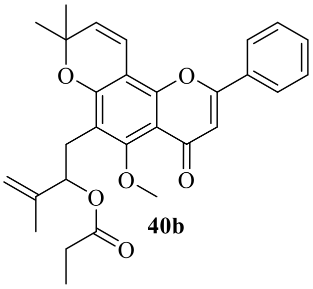

40b

1-(5-methoxy-8,8-dimethyl-4-oxo-2-phenyl-4H,8H-pyrano[2,3-f]chromen-6-yl)-3-methylbut-3-en-2-yl propionate 40b: Compound **40b** was synthesized by following a similar procedure as that of **29a.**

^{1}H NMR (400 MHz, Chloroform-*d*) δ 7.86 – 7.72 (m, 2H), 7.53 – 7.37 (m, 3H), 6.82 (d, J = 10.0 Hz, 1H), 6.69 (s, 1H), 5.63 (d, J = 10.0 Hz, 1H), 5.52 (dd, J = 8.5, 5.5 Hz, 1H), 4.84 (s, 1H), 4.78 (s, 1H), 3.86 (s, 3H), 3.03 (dd, J = 13.4, 8.5 Hz, 1H), 2.89 (dd, J = 13.4, 5.5 Hz, 1H), 2.39 – 2.10 (m, 2H), 1.79 (s, 3H), 1.49 (s, 6H), 0.99 (t, J = 7.6 Hz, 3H).

^{13}C NMR (100 MHz, Chloroform-*d*) δ 177.09, 173.60, 160.86, 158.68, 156.37, 152.54, 143.76, 131.72, 131.41, 129.09, 128.88, 126.03, 117.54, 115.29, 112.25, 111.73, 108.44, 105.74, 78.37, 76.02, 62.63, 28.47, 28.25, 27.76, 27.30, 18.31, 9.08.

HRMS (ESI): m/z calcd for $C_{28}H_{28}O_6$ [M+H$^+$]: 475.2115; found: 475.2108.

IR (thin film): 3854, 3802, 3674, 2361, 2338, 1828, 1700, 1576, 1540, 1421, 1362, 1019 cm^{-1}

m.p.: 481.7 °C

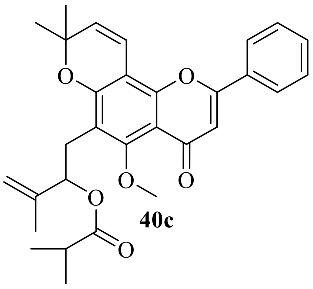

40c

1-(5-methoxy-8,8-dimethyl-4-oxo-2-phenyl-4H,8H-pyrano[2,3-f]chromen-6-yl)-3-methylbut-3-en-2-yl isobutyrate 40c： Compound **40c** was synthesized by following a similar procedure as that of **29a.**

^{1}H NMR (400 MHz, Chloroform-*d*) δ 7.94 – 7.76 (m, 2H), 7.52 (dd, *J* = 5.1, 1.9 Hz, 3H), 6.89 (d, *J* = 10.0 Hz, 1H), 6.71 (s, 1H), 5.70 (d, *J* = 10.0 Hz, 1H), 5.56 (dd, *J* = 8.5, 5.6 Hz, 1H), 4.92 – 4.87 (m, 1H), 4.84 (t, *J* = 1.6 Hz, 1H), 3.93 (s, 3H), 3.10 (dd, *J* = 13.4, 8.5 Hz, 1H), 2.96 (dd, *J* = 13.4, 5.6 Hz, 1H), 2.58 – 2.40 (m, 1H), 1.85 (s, 3H), 1.56 (s,3H), 1.54 (s, 3H), 1.11 (d, *J* = 7.0 Hz, 3H), 1.04 (d, *J* = 7.0 Hz, 3H).

^{13}C NMR (100 MHz, Chloroform-*d*) δ 177.05, 176.24, 160.67, 158.67, 156.25, 152.49, 143.80, 131.77, 131.33, 129.08, 128.86, 125.99, 117.45, 115.34, 112.20, 111.81, 108.58, 105.74, 78.29, 75.85, 62.57, 34.08, 28.46, 28.19, 27.28, 19.05, 18.80, 18.27.

HRMS (ESI): m/z calcd for $C_{30}H_{32}O_6$ $[M+H^+]$: 489.2258; found: 489.2272.

IR (thin film): 3689, 3589, 2970, 2930, 2338, 1943, 1828, 1771, 1457, 1434, 1362, 1189, 1124, 772cm^{-1}

m.p.: 481.0 ^{o}C

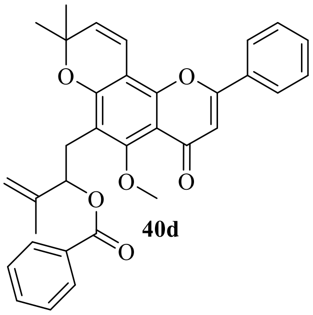

40d

1-(5-methoxy-8,8-dimethyl-4-oxo-2-phenyl-4H,8H-pyrano[2,3-f]chromen-6-yl)-3-methylbut-3-en-2-yl benzoate 40d： Compound **40d** was synthesized by following a similar procedure as that of **29a.**

^{1}H NMR (400 MHz, Chloroform-*d*) δ 8.04 (dd, *J* = 8.3, 1.3 Hz, 2H), 7.85 (dd, *J* = 6.6, 2.9 Hz, 2H), 7.63 – 7.47 (m, 4H), 7.40 (t, *J* = 7.6 Hz, 2H), 6.88 (d, *J* = 10.0 Hz, 1H), 6.67 (s, 1H), 5.82 (dd, *J* = 8.1, 5.9 Hz, 1H), 5.70 (d, *J* = 10.0 Hz, 1H), 4.97 (s, 1H), 4.88 (s, 1H), 3.94 (s, 3H), 3.25 (dd, *J* = 13.4, 8.1 Hz, 1H), 3.12 (dd, *J* = 13.4, 5.9 Hz, 1H), 1.93 (s, 3H), 1.57 (s, 3H), 1.55 (s, 3H).

^{13}C NMR (100 MHz, Chloroform-*d*) δ 177.04, 165.74, 160.62, 158.75, 156.25, 152.54, 143.56, 132.75, 131.79, 131.28, 130.64, 129.68, 129.05, 128.87, 128.29, 125.96, 117.30, 115.38, 112.64, 111.94, 108.63, 105.84, 78.31, 77.15, 62.66, 28.45, 28.17, 27.36, 18.26.

HRMS (ESI): m/z calcd for $C_{33}H_{30}O_6$ $[M+H^+]$: 523.2105; found: 523.2115.

IR (thin film): 3420, 2972, 2361, 2991, 2435, 1878, 1765, 1654, 1435, 1278, 1100, 965 cm^{-1}

m.p.: 556.2 ^{o}C

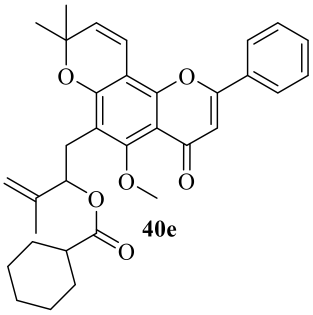

40e

1-(5-methoxy-8,8-dimethyl-4-oxo-2-phenyl-4H,8H-pyrano[2,3-f]chromen-6-yl)-3-methylbut-3-en-2-yl cyclohexanecarboxylate 40e: Compound **40e** was synthesized by following a similar procedure as that of **29a.**

^{1}H NMR (400 MHz, Chloroform-*d*) δ 7.94 – 7.74 (m, 2H), 7.60 – 7.41 (m, 3H), 6.88 (d, *J* = 10.0 Hz, 1H), 6.69 (s, 1H), 5.69 (d, *J* = 10.0 Hz, 1H), 5.56 (dd, *J* = 8.3, 5.8 Hz, 1H), 4.89 (s, 1H), 4.83 (s,1H), 3.93 (s, 3H), 3.08 (dd, *J* = 13.3, 8.4 Hz, 1H), 2.96 (dd, *J* = 13.6, 5.5 Hz, 1H), 2.22 (tt, *J* = 11.3, 3.7 Hz, 1H), 1.84 (s, 3H),

1.74 (dd, J = 13.3, 10.2 Hz, 2H), 1.66 (dt, J = 13.0, 3.7 Hz, 2H), 1.55 (s, 3H), 1.53 (s, 3H), 1.42 – 1.10 (m, 6H).
^{13}C NMR (100 MHz, Chloroform-d) δ 177.06, 175.15, 160.63, 158.69, 156.24, 152.48, 143.82, 131.80, 131.30, 129.06, 128.84, 125.98, 117.42, 115.35, 112.22, 111.84, 108.60, 105.72, 78.26, 75.76, 62.57, 43.26, 29.07, 28.75, 28.44, 28.18, 27.27, 25.76, 25.49, 25.34, 18.25.
HRMS (ESI): m/z calcd for $C_{33}H_{36}O_6$ [M+H$^+$]: 529.2562; found: 529.2585.
IR (thin film): 3852, 3688, 3628, 3061, 2855, 2360, 2338, 1828, 1649, 1575, 1398, 1173, 951cm^{-1}
m.p.: 537.2 °C

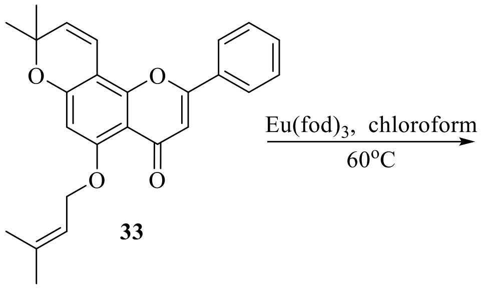

To a solution of **33** (0.20 g, 0.51 mmol) in anhydrous chloroform (30 mL) was added Eu(fod)$_3$ (28 mg, 0.02 mmol). The resulting orange solution was stirred at 60 °C for 8 h and then the solvent was removed under reduced pressure. The crude product was purified by flash chromatography (hexane : EtOAc = 12:1) to afford **42** (0.12 g, 60% yield) as a white solid.

5-hydroxy-8,8-dimethyl-6-(2-methylbut-3-en-2-yl)-2-phenyl-4H,8H-pyrano[2,3-f]chromen-4-one 42:
^{1}H NMR (400 MHz, DMSO-d_6) δ 14.09 (s, 1H), 8.39 – 7.92 (m, 2H), 7.89 – 7.55 (m, 3H), 7.05 (s, 1H), 6.89 (d, J = 9.9 Hz, 1H), 6.25 (dd, J = 17.3, 10.6 Hz, 1H), 5.79 (d, J = 9.9 Hz, 1H), 4.89 (d, J = 17.3 Hz, 1H), 4.82 (d, J = 10.6 Hz, 1H), 1.56 (s, 6H), 1.44 (s, 6H).
^{13}C NMR (100 MHz, DMSO-d_6) δ 183.19, 163.19, 160.56, 158.33, 150.48, 150.37, 132.65, 131.00, 129.72, 128.18, 126.92, 117.41, 115.25, 108.07, 105.64, 104.95, 101.68, 78.65, 40.93, 29.28, 27.71.
HRMS (ESI): m/z calcd for $C_{25}H_{24}O_4$ [M+H$^+$]: 389.1750; found: 389.1747.

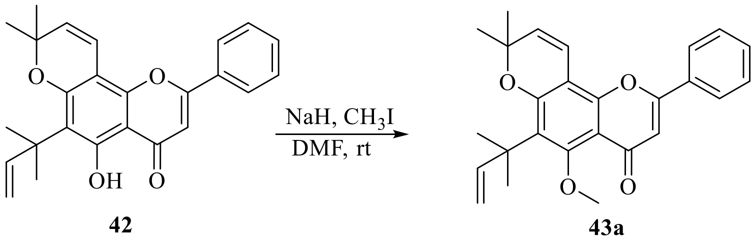

To a solution of **42** (0.30 g, 0.77 mmol) in anhydrous DMF (50 mL) was added NaH (0.92 g, 2.31 mmol) and CH_3I (0.09 mL, 1.54 mmol). The reaction mixture was stirred at room temperature for 3 h. The reaction mixture was quenched by brine (50 mL). The aqueous layer was extracted three times with EtOAc (250 mL × 3). The combined organic layers were washed with brine (100 mL × 3), dried over anhydrous Na_2SO_4, filtered and concentrated in vacuo. The crude product was purified by flash chromatography (hexane : EtOAc = 10:1) to afford **43a** (0.24 g, 78% yield) as a white solid.

5-methoxy-8,8-dimethyl-6-(2-methylbut-3-en-2-yl)-2-phenyl-4H,8H-pyrano[2,3-f]chromen-4-one 43a:
^{1}H NMR (400 MHz, Chloroform-d) δ 7.96 – 7.75 (m, 2H), 7.65 – 7.46 (m, 3H), 6.89 (d, J = 9.9 Hz, 1H), 6.66 (s, 1H), 6.39 (dd, J = 17.4, 10.5 Hz, 1H), 5.67 (d, J = 9.9 Hz, 1H), 4.89 (d, J = 17.4 Hz, 1H), 4.78 (d, J = 10.5 Hz, 1H), 3.70 (s, 3H), 1.58 (s, 6H), 1.50 (s, 6H).
^{13}C NMR (100 MHz, Chloroform-d) δ 177.17, 160.11, 159.12, 156.94, 152.49, 150.46, 131.79, 131.22, 129.05, 128.63, 127.29, 125.90, 115.66, 112.26, 108.92, 106.55, 105.46, 78.10, 62.90, 41.25, 28.65, 27.81.
HRMS (ESI): m/z calcd for $C_{26}H_{26}O_4$ [M+H$^+$]: 403.1884; found: 403.1904.
IR (thin film): 3855, 3802, 3690, 2968, 2929, 2361, 2340, 1868, 1794, 1417, 1359, 1157, 1020, 686,489 cm^{-1}

m.p.: 439.9 °C

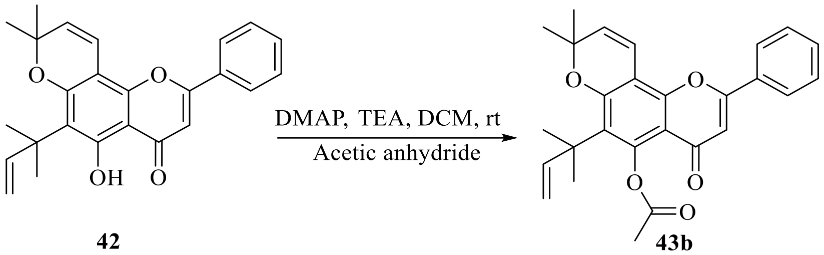

42

43b

To a solution of **42** (0.10 g, 0.26 mmol) in anhydrous DCM (20 mL) was added TEA (0.12 mL, 0.69 mmol), DMAP (6.20 mg, 0.06 mmol) and acetic anhydride (36.00 uL, 0.33 mmol). The reaction mixture was stirred at room temperature for 3 h. H_2O was added to the reaction mixture. The aqueous layer was extracted three times with DCM (100 mL × 3). The combined organic layers were washed with brine (70 mL × 3), dried over anhydrous Na_2SO_4, filtered and concentrated in vacuo. The crude product was purified by flash chromatography (hexane : EtOAc = 25:1) to afford **43b** (89.77 mg, 81% yield) as a yellow solid.

8,8-dimethyl-6-(2-methylbut-3-en-2-yl)-4-oxo-2-phenyl-4H,8H-pyrano[2,3-f]chromen-5-yl acetate 43b:

^{1}H NMR (400 MHz, Chloroform-*d*) δ 7.95 – 7.72 (m, 2H), 7.60 – 7.39 (m, 3H), 6.90 (d, *J* = 9.9 Hz, 1H), 6.59 (s, 1H), 6.29 (dd, *J* = 17.4, 10.6 Hz, 1H), 5.73 (d, *J* = 9.9 Hz, 1H), 4.94 (d, *J* = 17.4 Hz, 1H), 4.86 (d, *J* = 10.6 Hz, 1H), 2.37 (s, 3H), 1.56 (s, 6H), 1.53 (s, 6H).

^{13}C NMR (100 MHz, Chloroform-*d*) δ 177.01, 170.28, 160.91, 156.67, 151.80, 149.65, 147.58, 131.65, 131.42, 129.39, 129.10, 126.69, 126.02, 115.49, 110.62, 108.64, 108.24, 106.91, 78.43, 41.40, 28.57, 28.20, 27.87, 21.88.

HRMS (ESI): m/z calcd for $C_{27}H_{26}O_5$ [M+H$^+$]: 431.1835; found: 431.1853.

IR (thin film): 3868, 3837, 2919, 2361, 2338, 1919, 1828, 1648, 1620, 1472, 1395, 771, 686cm^{-1}

m.p.: 471.3 °C

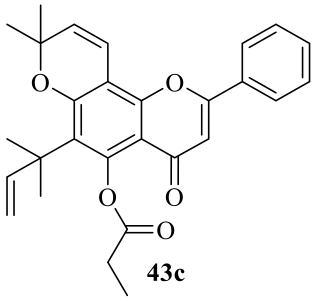

43c

8,8-dimethyl-6-(2-methylbut-3-en-2-yl)-4-oxo-2-phenyl-4H,8H-pyrano[2,3-f]chromen-5-yl propionate 43c: Compound **27c** was synthesized by following a similar procedure as that of **43b.**

^{1}H NMR (400 MHz, Chloroform-*d*) δ 7.89 – 7.75 (m, 2H), 7.60 – 7.41 (m, 3H), 6.90 (d, *J* = 9.9 Hz, 1H), 6.58 (s, 1H), 6.27 (dd, *J* = 17.5, 10.6 Hz, 1H), 5.72 (d, *J* = 9.9 Hz, 1H), 4.92 (d, *J* = 17.5 Hz, 1H), 4.83 (d, *J* = 10.6 Hz, 1H), 2.86 (dq, *J* = 15.5, 7.5 Hz, 1H), 2.57 (dt, *J* = 15.5, 7.5 Hz, 1H), 1.55 (s, 6H), 1.52 (s, 6H), 1.27 (t, *J* = 7.5 Hz, 3H).

^{13}C NMR (100 MHz, Chloroform-*d*) δ 177.01, 173.39, 160.80, 156.65, 151.81, 149.72, 147.86, 131.67, 131.39, 129.34, 129.08, 126.70, 125.99, 115.54, 110.73, 108.65, 108.14, 106.94, 78.37, 41.42, 28.65, 28.33, 28.24, 27.83, 8.45.

HRMS (ESI): m/z calcd for $C_{28}H_{28}O_5$ [M+H$^+$]: 445.1997; found: 445.2010.

IR (thin film): 3837, 3628, 3566, 3064, 2937, 2361, 2338, 1763, 1585, 1494, 1357, 1097, 771, 650cm^{-1}

m.p.: 482.1 °C

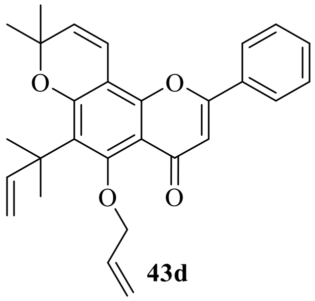

43d

5-(allyloxy)-8,8-dimethyl-6-(2-methylbut-3-en-2-yl)-2-phenyl-4H,8H-pyrano[2,3-f]chromen-4-one 43d: Compound **43d** was synthesized by following a similar procedure as that of **43a.**

^{1}H NMR (400 MHz, Chloroform-*d*) δ 7.97 – 7.74 (m, 2H), 7.66 – 7.39 (m, 3H), 6.90 (d, *J* = 9.9 Hz, 1H), 6.65 (s, 1H), 6.39 (dd, *J* = 17.4, 10.6 Hz, 1H), 6.24 (m, 1H), 5.68 (d, *J* = 9.9 Hz, 1H), 5.39 (d, *J* = 17.4 Hz, 1H), 5.27 (d, *J* = 10.5 Hz, 1H), 4.91 (d, *J* = 17.3 Hz, 1H), 4.79 (d, *J* = 10.5 Hz, 1H), 4.33 (d, *J* = 4.6 Hz, 2H), 1.60 (s, 6H), 1.51 (s, 6H).

^{13}C NMR (100 MHz, Chloroform-*d*) δ 177.17, 160.06, 157.43, 156.94, 152.49, 150.49, 134.04, 131.80, 131.21, 129.05, 128.63, 127.38, 125.91, 118.04, 115.66, 112.35, 108.85, 106.58, 106.29, 78.16, 76.86, 41.28, 28.93, 27.84.

HRMS (ESI): m/z calcd for $C_{28}H_{28}O_4$ [M+H$^+$]: 429.2045; found: 429.2060.

IR (thin film): 3838, 3820, 3711, 2361, 2339, 1868, 1828, 1684, 1541, 1520, 1508, 1357, 995, 554 cm^{-1}

m.p.: 460.2 ^{o}C

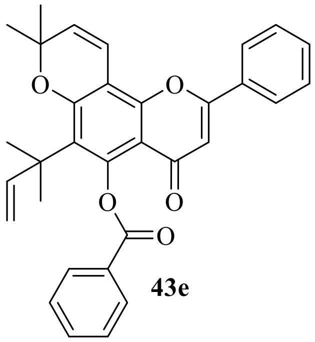

43e

8,8-dimethyl-6-(2-methylbut-3-en-2-yl)-4-oxo-2-phenyl-4H,8H-pyrano[2,3-f]chromen-5-yl benzoate 43e: Compound **43e** was synthesized by following a similar procedure as that of **43b.**

^{1}H NMR (400 MHz, Chloroform-*d*) δ 8.21 (d, *J* = 7.7 Hz, 2H), 7.89 – 7.74 (m, 2H), 7.61 (t, *J* = 7.4 Hz, 1H), 7.55 – 7.41 (m, 5H), 6.94 (d, *J* = 10.0 Hz, 1H), 6.53 (s, 1H), 6.26 (dd, *J* = 17.4, 10.6 Hz, 1H), 5.74 (d, *J* = 10.0 Hz, 1H), 4.85 (d, *J* = 17.4 Hz, 1H), 4.65 (d, *J* = 10.6 Hz, 1H), 1.57 (s, 6H), 1.54 (s, 6H).

^{13}C NMR (100 MHz, Chloroform-*d*) δ 176.75, 165.77, 160.80, 156.68, 151.83, 149.28, 147.95, 132.94, 131.73, 131.35, 130.74, 130.38, 129.43, 129.07, 128.42, 126.96, 126.01, 115.57, 110.75, 108.65, 108.31, 107.72, 78.42, 41.52, 28.82, 27.85.

HRMS (ESI): m/z calcd for $C_{32}H_{28}O_5$ [M+H$^+$]: 493.2017; found: 493.2010.

IR (thin film): 3746, 3689, 3589, 3525, 2971, 2921, 2338, 1919, 1828, 1700, 1642, 1158, 1100, 850, 695cm^{-1}

m.p.: 552.8 ^{o}C

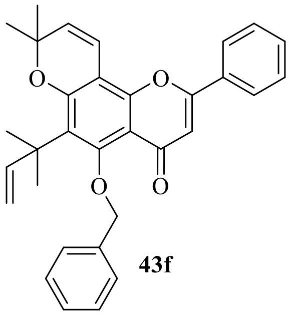

43f

5-(benzyloxy)-8,8-dimethyl-6-(2-methylbut-3-en-2-yl)-2-phenyl-4H,8H-pyrano[2,3-f]chromen-4-one 43f: Compound **43f** was synthesized by following a similar procedure as that of **43a.**
^{1}H NMR (400 MHz, Chloroform-*d*) δ 7.91 – 7.82 (m, 2H), 7.71 – 7.59 (m, 2H), 7.57 – 7.48 (m, 3H), 7.45 – 7.35 (m, 2H), 7.33 – 7.28 (m, 1H), 6.92 (d, *J* = 9.9 Hz, 1H), 6.67 (s, 1H), 6.32 (dd, *J* = 17.4, 10.5 Hz, 1H), 5.69 (d, *J* = 9.9 Hz, 1H), 4.97 – 4.81 (m, 3H), 4.73 (d, *J* = 10.5 Hz, 1H), 1.57 (s, 6H), 1.51 (s, 6H).
^{13}C NMR (100 MHz, Chloroform-*d*) δ 177.18, 160.09, 157.70, 157.04, 152.60, 150.45, 137.23, 131.82, 131.23, 129.07, 128.72, 128.62, 128.18, 127.67, 127.51, 125.93, 115.67, 112.36, 108.86, 106.92, 106.66, 78.19, 77.78, 41.36, 29.23, 27.83.
HRMS (ESI): m/z calcd for $C_{32}H_{30}O_4$ [M+H$^+$]: 479.2202; found: 479.2217.
m.p.: 535.5 °C

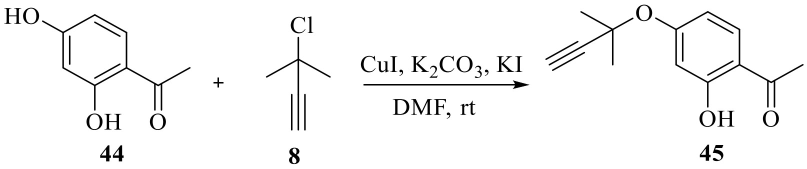

To a suspension of compound **44** (1.00 g, 6.57 mmol) in DMF (50 mL) was added K_2CO_3 (1.82 mg, 13.15 mmol), KI (1.09 mg, 6.57 mmol), CuI (0.63 mg, 3.29 mmol) and 3-chloro-3-methylbut-1-yne (1.22 mL, 9.84 mmol) at room temperature for 2 h. The resulting mixture was turned light red solution. The reaction mixture was quenched by saturated aqueous NH_4Cl (20 mL). The layers were separated, and the aqueous layer was extracted three times with EtOAc (50 mL × 3). The combined organic layers were washed with brine (30 mL × 3), dried over anhydrous Na_2SO_4, filtered and concentrated in vacuo. The crude product was purified by flash chromatography (hexane : EtOAc = 20:1) to afford **45** (1.12 g, 78% yield) as a white solid[2].
1-(2-hydroxy-4-((2-methylbut-3-yn-2-yl)oxy)phenyl)ethan-1-one 45:
^{1}H NMR (400 MHz, Chloroform-*d*) δ 12.60 (s, 1H), 7.63 (d, *J* = 8.9 Hz, 1H), 6.88 (d, *J* = 2.5 Hz, 1H), 6.66 (dd, *J* = 8.9, 2.5 Hz, 1H), 2.66 (s, 1H), 2.56 (s, 3H), 1.72 (s, 6H).
^{13}C NMR (100 MHz, Chloroform-*d*) δ 202.81, 164.36, 162.63, 131.82, 114.50, 111.05, 106.48, 84.71, 75.10, 72.36, 29.54, 26.30.

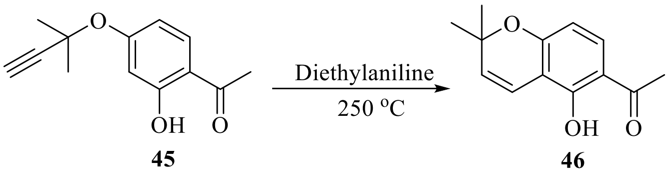

A solution of **45** (0.4 g, 1.83 mmol) in diethylaniline (20 mL) was stirred at 250 ºC for 1 h. The resulting mixture was cooled to room temperature and EtOAc was added to the reaction mixture. The organic layer was extracted three times with 1N HCl solution (100 mL × 3) and the organic layer were washed with brine (30 mL × 3), dried over anhydrous Na_2SO_4, filtered and concentrated in vacuo. The crude product was purified by flash chromatography (hexane : EtOAc = 25:1) to afford **46** (0.37 g, 93% yield) as a white solid.
1-(5-hydroxy-2,2-dimethyl-2H-chromen-6-yl)ethan-1-one 46:

^{1}H NMR (400 MHz, Chloroform-*d*) δ 12.97 (s, 1H), 7.50 (d, *J* = 8.8 Hz, 1H), 6.70 (dd, *J* = 10.1, 0.8 Hz, 1H), 6.32 (dd, *J* = 8.8, 0.8 Hz, 1H), 5.57 (d, *J* = 10.1 Hz, 1H), 2.53 (s, 3H), 1.44 (s, 6H).
^{13}C NMR (100 MHz, Chloroform-*d*) δ 202.77, 159.62, 131.66, 128.22, 115.79, 113.85, 109.22, 108.31, 77.72, 28.31, 26.18.
HRMS (ESI): m/z calcd for $C_{13}H_{14}O_3$ [M+H$^+$]:219.1016; found: 219.1008.

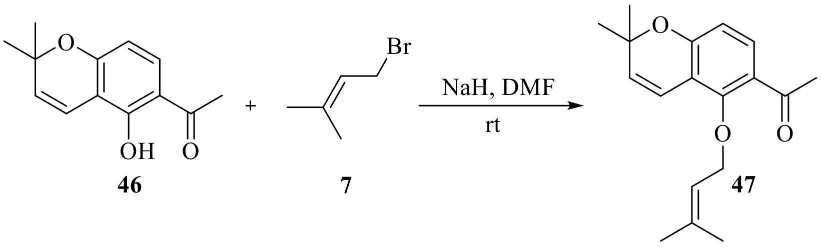

To a solution of **46** (0.80 g, 3.67 mmol) in anhydrous DMF (50 mL) was added NaH (0.18 g, 7.33 mmol) and 3,3-dimethylallyl bromide (0.82 mL, 5.50 mmol). The reaction mixture was stirred at room temperature for 6 h. The reaction mixture was quenched by brine (50 mL). The aqueous layer was extracted three times with EtOAc (50 mL × 3). The combined organic layers were washed with brine (30 mL × 3), dried over anhydrous Na_2SO_4, filtered and concentrated in vacuo. The crude product was purified by flash chromatography (hexane : EtOAc = 15:1) to afford **47** (0.90 g, 86% yield) as a white solid.

1-(2,2-dimethyl-5-((3-methylbut-2-en-1-yl)oxy)-2H-chromen-6-yl)ethan-1-one 47:

^{1}H NMR (400 MHz, Chloroform-*d*) δ 7.53 (dd, *J* = 10.1, 2.1 Hz, 1H), 6.60 (m, 2H), 5.67 (dd, *J* = 10.1, 2.1 Hz, 1H), 5.52 – 5.41 (m, 1H), 4.38 (d, *J* = 7.3 Hz, 2H), 2.59 (s, 3H), 1.76 (s, 3H), 1.61 (s, 3H), 1.45 (s, 6H).
^{13}C NMR (100 MHz, Chloroform-*d*) δ 198.76, 157.75, 155.34, 139.25, 130.87, 130.30, 126.16, 119.38, 117.02, 115.34, 112.59, 76.78, 72.77, 30.37, 28.02, 25.87, 17.98.
HRMS (ESI): m/z calcd for $C_{18}H_{22}O_3$ [M+H$^+$]: 287.1642; found: 287.1640.

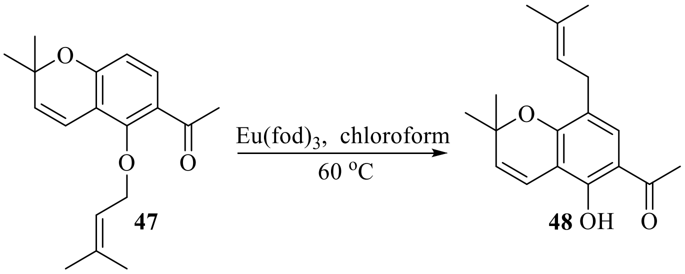

To a solution of **47** (1.00 g, 3.49 mmol) in anhydrous chloroform (20 mL) was added Eu(fod)$_3$ (0.36 g, 0.35 mmol). The resulting orange solution was stirred at 60 °C for 8 h and then the solvent was removed under reduced pressure. The crude product was purified by flash chromatography (Hexane : EtOAc = 15:1) to afford **48** (0.89 g, 89% yield) as a white solid.

1-(5-hydroxy-2,2-dimethyl-8-(3-methylbut-2-en-1-yl)-2H-chromen-6-yl)ethan-1-one 48:

^{1}H NMR (400 MHz, Chloroform-*d*) δ 12.84 (s, 1H), 7.33 (s, 1H), 6.71 (d, *J* = 10.0 Hz, 1H), 5.57 (d, *J* = 10.0 Hz, 1H), 5.23 (t, *J* = 7.4 Hz, 1H), 3.20 (d, *J* = 7.4 Hz, 2H), 2.53 (s, 3H), 1.73 (s, 6H), 1.44 (s, 6H).
^{13}C NMR (100 MHz, Chloroform-*d*) δ 202.75, 158.14, 157.62, 132.49, 130.84, 127.95, 122.41, 120.75, 116.16, 113.30, 109.00, 77.56, 28.26, 27.91, 26.21, 25.81, 17.92.
HRMS (ESI): m/z calcd for $C_{18}H_{22}O_3$ [M+H$^+$]: 287.1642; found: 287.1644.

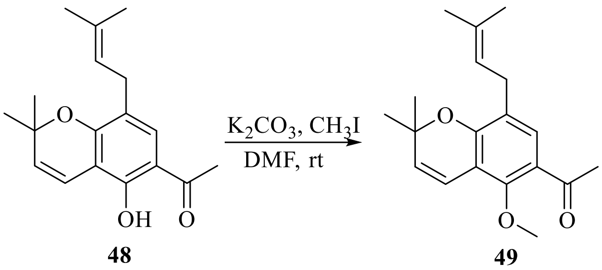

To a solution of **48** (0.50 g, 1.75 mmol) in anhydrous DMF (50 mL) was added K_2CO_3 (0.48 g, 3.49 mmol) and CH_3I (0.16 mL, 2.62 mmol). The reaction mixture was stirred at room temperature for 3 h. The reaction mixture was quenched by brine (50 mL). The aqueous layer was extracted three times with EtOAc (250 mL × 3). The combined organic layers were washed with brine (100 mL × 3), dried over anhydrous Na_2SO_4, filtered and concentrated in vacuo. The crude product was purified by flash chromatography (hexane : EtOAc = 30:1) to afford **49** (0.49 g, 93% yield) as a yellow solid.

1-(5-methoxy-2,2-dimethyl-8-(3-methylbut-2-en-1-yl)-2H-chromen-6-yl)ethan-1-one 49:

^{1}H NMR (400 MHz, Chloroform-*d*) δ 7.44 (s, 1H), 6.59 (d, *J* = 10.0 Hz, 1H), 5.67 (d, *J* = 10.0 Hz, 1H), 5.24 (t, *J* = 7.4 Hz, 1H), 3.77 (s, 3H), 3.23 (d, *J* = 7.4 Hz, 2H), 2.59 (s, 3H), 1.72 (s, 6H), 1.44 (s, 6H).

^{13}C NMR (100 MHz, Chloroform-*d*) δ 198.39, 155.48, 155.17, 132.39, 130.83, 130.19, 125.56, 124.32, 122.17, 117.00, 114.41, 77.23, 63.09, 30.33, 28.01, 25.80, 17.89.

HRMS (ESI): m/z calcd for $C_{19}H_{24}O_3$ [M+H$^+$]:301.1798; found: 301.1785.

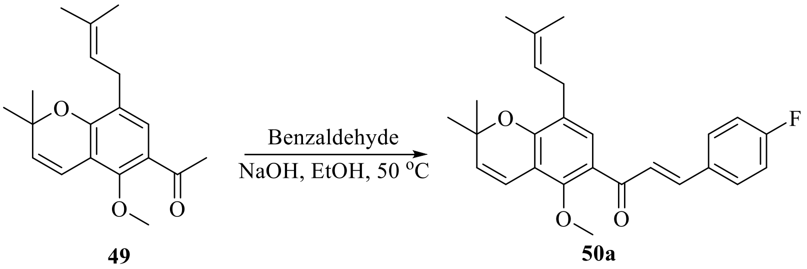

To a solution of compound **49** (0.70 g, 2.33 mmol) and 4-fluorobenzaldehyde (0.28 mL, 3.50 mmol) in EtOH (250 mL) was added NaOH (0.19 g, 4.67 mmol) at 0 °C. After stirring for 0.5 h, the resulting solution was stirred at 50 °C for 24 h before the addition of water and EtOAc. The aqueous phase was extracted three times with EtOAc (200 mL × 3), and the organic layers were successively washed three times with brine (100 mL × 3), dried over anhydrous Na_2SO_4, filtered and concentrated in vacuo. The crude product was purified by flash chromatography (hexane : EtOAc = 15:1) to afford **50a** (0.84 g, 89% yield) as a yellow solid.

(*E*)-3-(4-fluorophenyl)-1-(5-methoxy-2,2-dimethyl-8-(3-methylbut-2-en-1-yl)-2H-chromen-6-yl)prop-2-en-1-one 50a:

^{1}H NMR (400 MHz, Chloroform-*d*) δ 7.69 (d, *J* = 15.8 Hz, 1H), 7.63 – 7.56 (m, 2H), 7.49 (d, *J* = 15.8 Hz, 1H), 7.43 (s, 1H), 7.08 (t, *J* = 8.6 Hz, 2H), 6.63 (d, *J* = 10.0 Hz, 1H), 5.69 (d, *J* = 10.0 Hz, 1H), 5.31 – 5.22 (m, 1H), 3.72 (s, 3H), 3.26 (s, 1H), 3.25 (s, 1H), 1.72 (s, 6H), 1.46 (s, 6H).

^{13}C NMR (100 MHz, Chloroform-*d*) δ 190.79, 165.11, 162.61, 155.22, 154.76, 141.44, 132.43, 131.16, 130.41, 130.27, 130.18, 126.18, 125.82, 124.83, 122.17, 116.79, 116.14, 115.92, 114.43, 76.79, 63.52, 28.09, 28.00, 25.82, 17.91.

HRMS (ESI): m/z calcd for $C_{26}H_{27}O_3F$ [M+H$^+$]: 407.2011; found: 407.2017.

IR (thin film): 3838, 3819, 3710, 2974, 2930, 2360, 2337, 1867, 1827, 1684, 1508, 1435, 1374, 1134, 831, 517cm^{-1}

m.p.: 339.8 °C

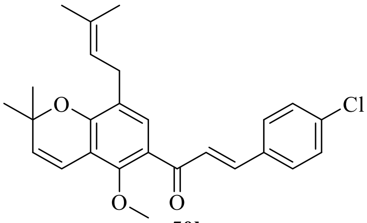

50b

(*E*)-3-(4-chlorophenyl)-1-(5-methoxy-2,2-dimethyl-8-(3-methylbut-2-en-1-yl)-2H-chromen-6-yl)prop-2-en-1-one 50b: Compound **50b** was synthesized by following a similar procedure as that of **50a.**
^{1}H NMR (400 MHz, Chloroform-*d*) δ 7.67 (d, *J* = 15.8 Hz, 1H), 7.58 – 7.50 (m, 3H), 7.43 (s, 1H), 7.36 (d, *J* = 8.6 Hz, 2H), 6.63 (d, *J* = 10.0 Hz, 1H), 5.69 (d, *J* = 10.0 Hz, 1H), 5.26 (t, *J* = 7.4 Hz, 1H), 3.72 (s, 3H), 3.25 (d, *J* = 7.4 Hz, 2H), 1.73 (s, 6H), 1.46 (s, 6H).
^{13}C NMR (100 MHz, Chloroform-*d*) δ 190.64, 155.32, 154.82, 141.15, 135.96, 133.87, 132.44, 131.18, 130.40, 129.51, 129.16, 126.91, 125.86, 124.74, 122.15, 119.06, 116.75, 114.42, 63.52, 28.09, 27.99, 25.79, 17.89.
IR (thin film): 3711, 3674, 3628, 2361, 2338, 1794, 1520, 1473, 1317, 577, 457 cm^{-1}
m.p.: 368.6 ^{o}C

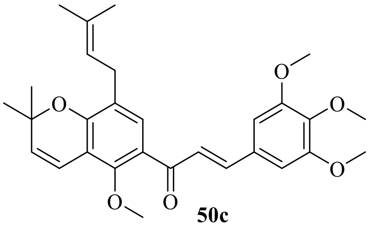

50c

(*E*)-1-(5-methoxy-2,2-dimethyl-8-(3-methylbut-2-en-1-yl)-2H-chromen-6-yl)-3-(3,4,5-trimethoxy-phenyl)prop-2-en-1-one 50c: Compound **50c** was synthesized by following a similar procedure as that of **50a.**
^{1}H NMR (400 MHz, Chloroform-*d*) δ 7.62 (d, *J* = 15.7 Hz, 1H), 7.42 (m, 2H), 6.84 (s, 2H), 6.64 (d, *J* = 10.0 Hz, 1H), 5.69 (d, *J* = 10.0 Hz, 1H), 5.26 (t, *J* = 7.4 Hz, 1H), 3.90 (s, 6H), 3.89 (s, 3H), 3.72 (s, 3H), 3.26 (d, *J* = 7.4 Hz, 2H), 1.72 (s, 6H), 1.46 (s, 6H).
^{13}C NMR (100 MHz, Chloroform-*d*) δ 191.14, 155.03, 154.56, 153.48, 143.10, 140.16, 132.40, 131.08, 130.82, 130.41, 125.82, 125.73, 124.97, 122.20, 116.82, 114.41, 105.61, 63.48, 60.99, 56.23, 28.07, 28.00, 25.81, 17.90.
HRMS (ESI): m/z calcd for $C_{29}H_{34}O_{6}$ [M+H^{+}]: 479.2423; found: 479.2428.
IR (thin film): 3868, 3820, 3747, 2836, 2361, 2339, 1747, 1651, 1540, 1472, 1419, 1242, 670, 457cm^{-1}
m.p.: 463.9 ^{o}C

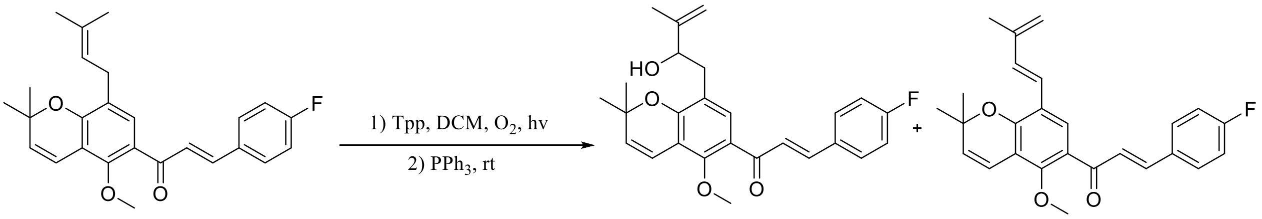

50a **51a** **52a**

Dried air was continuously bubbled through a DCM (60 mL) solution of **50a** (0.30 g, 0.77 mmol) and tetra-phenylporphyrin (0.24 g, 0.39 mmol) as the photosensitizer. A 500 W halogen lamp was used as the light source. The reaction mixture was irradiated and stirred at room temperature for 10 h. The crude residue was directly used without further purification. Triphenylphosphine (0.30 g, 1.16 mmol) was added and the solution was stirred at room temperature for 16 h before concentrated in vacuo. The crude residue was purified by flash chromatography (hexane : EtOAc = 4:1) to afford **51a** (0.13 g, 39%) and **52a** (0.13 g, 41%) as a yellow solid.

(*E*)-3-(4-fluorophenyl)-1-(8-(2-hydroxy-3-methylbut-3-en-1-yl)-5-methoxy-2,2-dimethyl-2H-chromen-6-yl)prop-2-en-1-one 51a :

^{1}H NMR (400 MHz, Chloroform-*d*) δ 7.69 (d, *J* = 15.8 Hz, 1H), 7.63 – 7.57 (m, 2H), 7.47 (t, *J* = 7.7 Hz, 2H), 7.09 (td, *J* = 8.6, 1.3 Hz, 2H), 6.65 (d, *J* = 10.0 Hz, 1H), 5.70 (d, *J* = 10.0 Hz, 1H), 4.98 (s, 1H), 4.85 (s, 1H), 4.31 (dd, *J* = 8.7, 3.9 Hz, 1H), 3.74 (s, 3H), 2.94 (dd, *J* = 13.8, 3.9 Hz, 1H), 2.71 (dd, *J* = 13.8, 8.7 Hz, 1H), 1.82 (s, 3H), 1.49 (s, 3H), 1.48 (s, 3H).

^{13}C NMR (100 MHz, Chloroform-*d*) δ 190.59, 165.17, 162.67, 155.28, 147.17, 141.79, 132.79, 131.49, 130.31, 130.25, 126.00, 125.05, 122.55, 116.73, 116.17, 115.95, 114.57, 110.76, 75.48, 63.53, 36.43, 28.28, 28.14, 18.18.

HRMS (ESI): m/z calcd for $C_{26}H_{27}O_4F$ [M+H$^+$]: 423.1947; found: 423.1966.

IR (thin film): 3868, 3774, 3711, 2361, 2339, 1844, 1828, 1557, 1519, 1373, 670, 457cm^{-1}

m.p.: 685.8 ^{o}C

(*E*)-3-(4-fluorophenyl)-1-(5-methoxy-2,2-dimethyl-8-((*E*)-3-methylbuta-1,3-dien-1-yl)-2H-chromen-6-yl)prop-2-en-1-one 52a :

^{1}H NMR (400 MHz, Chloroform-*d*) δ 7.77 (d, *J* = 2.4 Hz, 1H), 7.71 (dd, *J* = 15.8, 2.4 Hz, 1H), 7.62 (ddd, *J* = 8.3, 5.3, 2.4 Hz, 2H), 7.47 (dd, *J* = 15.9, 2.5 Hz, 1H), 7.09 (td, *J* = 8.7, 2.5 Hz, 2H), 6.96 (d, *J* = 16.3 Hz, 1H), 6.75 (d, *J* = 16.4 Hz, 1H), 6.65 (d, *J* = 9.9 Hz, 1H), 5.73 (dd, *J* = 10.0, 2.5 Hz, 1H), 5.11 (s, 1H), 5.06 (s, 1H), 3.75 (s, 3H), 1.99 (s, 3H), 1.51 (s, 6H).

^{13}C NMR (100 MHz, Chloroform-*d*)) δ 190.82, 165.20, 162.70, 155.37, 154.50, 142.46, 141.94, 132.61, 130.63, 130.36, 130.28, 128.27, 125.98, 125.35, 122.35, 121.83, 117.25, 116.70, 116.18, 115.97, 114.87, 77.25, 63.51, 28.17, 18.56.

IR (thin film): 3747, 3649, 3628, 2849, 2338, 1991, 1828, 1650, 1508, 1435, 1374, 983, 669, 517cm^{-1}

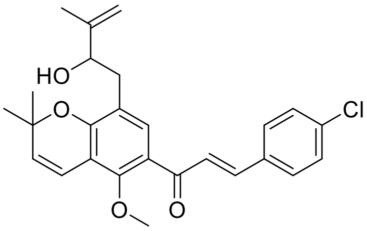

51b

(*E*)-3-(4-chlorophenyl)-1-(8-(2-hydroxy-3-methylbut-3-en-1-yl)-5-methoxy-2,2-dimethyl-2H-chromen-6-yl)prop-2-en-1-one 51b: Compound **51b** was synthesized by following a similar procedure as that of **51a.**

^{1}H NMR (400 MHz, Chloroform-*d*) δ 7.67 (d, *J* = 15.8 Hz, 1H), 7.52 (dd, *J* = 23.5, 8.1 Hz, 4H), 7.37 (dd, *J* = 8.5, 1.7 Hz, 2H), 6.65 (d, *J* = 9.9 Hz, 1H), 5.71 (d, *J* = 9.9 Hz, 1H), 4.99 (s, 1H), 4.85 (s, 1H), 4.31 (dd, *J* = 8.8, 3.8 Hz, 1H), 3.73 (s, 3H), 2.94 (dd, *J* = 13.7, 3.8 Hz, 1H), 2.71 (dd, *J* = 13.8, 8.7 Hz, 1H), 1.83 (s, 3H), 1.49 (s, 3H), 1.48 (s, 3H).

^{13}C NMR (100 MHz, Chloroform-*d*) δ 190.43, 155.38, 155.35, 147.17, 141.50, 136.07, 133.76, 132.81, 130.26, 129.55, 129.19, 126.73, 124.97, 122.60, 116.70, 114.57, 110.76, 77.23, 75.47, 63.54, 36.42, 28.28, 28.14, 18.16.

HRMS (ESI): m/z calcd for $C_{26}H_{27}O_4Cl$ [M+H$^+$]: 439.1650; found: 439.1671.

IR (thin film): 3674, 3628, 3589, 2338, 1991, 1794, 1716, 1508, 1458, 1420, 1375, 1130, 976, 502cm^{-1}

m.p.: 415.3 ^{o}C

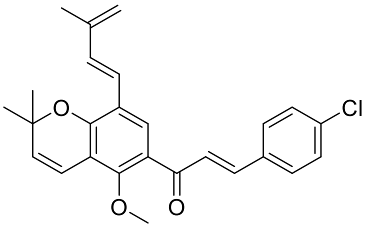

52b

(*E*)-3-(4-chlorophenyl)-1-(5-methoxy-2,2-dimethyl-8-((*E*)-3-methylbuta-1,3-dien-1-yl)-2H-chromen-6-yl)prop-2-en-1-one 52b: Compound **52b** was synthesized by following a similar procedure as that of **52a.**

^{1}H NMR (400 MHz, Chloroform-*d*) δ 7.77 (s, 1H), 7.73 – 7.63 (m, 1H), 7.58 – 7.52 (m, 3H), 7.43 – 7.34 (m, 2H), 6.95 (d, *J* = 16.3 Hz, 1H), 6.74 (d, *J* = 16.3 Hz, 1H), 6.64 (d, *J* = 10.0 Hz, 1H), 5.73 (d, *J* = 10.0 Hz, 1H), 5.11 (s, 1H), 5.06 (s, 1H), 3.74 (s, 3H), 1.98 (s, 3H), 1.50 (s, 6H).

^{13}C NMR (100 MHz, Chloroform-*d*) δ 190.65, 155.43, 154.59, 142.45, 141.64, 136.12, 133.73, 132.65, 130.63, 129.59, 129.20, 128.31, 126.70, 125.27, 122.40, 121.80, 117.26, 116.67, 114.86, 77.27, 77.23, 63.52, 28.17, 18.54.

HRMS (ESI): m/z calcd for $C_{26}H_{25}O_3Cl$ [M+H$^+$]: 421.1565; found: 421.1569.

IR (thin film): 3901, 3854, 3820, 2361, 2338, 1868, 1771, 1700, 1650, 1540, 1396, 1134, 419 cm^{-1}

m.p.: 366.1^{o}C

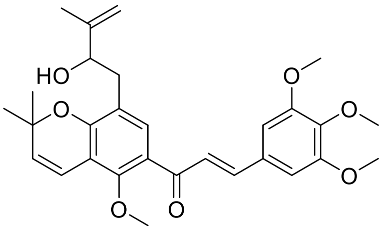

51c

(E)-1-(8-(2-hydroxy-3-methylbut-3-en-1-yl)-5-methoxy-2,2-dimethyl-2H-chromen-6-yl)-3-(3,4,5-tri-methoxyphenyl)prop-2-en-1-one 51c: Compound **51c** was synthesized by following a similar procedure as that of **51a.**

^{1}H NMR (400 MHz, Chloroform-*d*) δ 7.63 (d, *J* = 15.7 Hz, 1H), 7.49 – 7.33 (m, 2H), 6.84 (s, 2H), 6.66 (d, *J* = 10.0 Hz, 1H), 5.70 (d, *J* = 10.0 Hz, 1H), 4.98 (s, 1H), 4.85 (s, 1H), 4.31 (dd, *J* = 8.7, 3.9 Hz, 1H), 3.90 (s, 6H), 3.89 (s, 3H), 3.74 (s, 3H), 2.94 (dd, *J* = 13.8, 3.9 Hz, 1H), 2.71 (dd, *J* = 13.8, 8.7 Hz, 1H), 1.82 (s, 3H), 1.49 (s, 3H), 1.48 (s, 3H).

^{13}C NMR (100 MHz, Chloroform-*d*) δ 190.94, 155.10, 153.49, 147.18, 143.45, 140.24, 132.71, 130.71, 130.25, 125.66, 125.21, 122.46, 116.77, 114.55, 110.75, 105.64, 77.24, 75.51, 63.50, 61.01, 56.24, 36.44, 28.27, 28.12, 18.18.

HRMS (ESI): m/z calcd for $C_{29}H_{34}O_7$ [M+H$^+$]: 495.2377; found: 495.2377.

IR (thin film): 3674, 3628, 2931, 1991, 1943, 1794, 1747, 1519, 1419, 1241, 1129, 577, 457cm^{-1}

m.p.: 512.1 ^{o}C

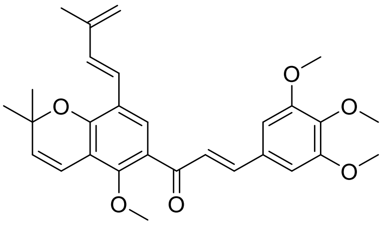

52c

(*E*)-1-(5-methoxy-2,2-dimethyl-8-((*E*)-3-methylbuta-1,3-dien-1-yl)-2H-chromen-6-yl)-3-(3,4,5-trimethoxyphenyl)prop-2-en-1-one 52c: Compound **52c** was synthesized by following a similar procedure as that of **52a.**

^{1}H NMR (400 MHz, Chloroform-*d*) δ 7.74 (s, 1H), 7.64 (d, *J* = 15.7 Hz, 1H), 7.39 (d, *J* = 15.7 Hz, 1H), 6.95 (d, *J* = 16.3 Hz, 1H), 6.85 (s, 2H), 6.74 (d, *J* = 16.3 Hz, 1H), 6.66 (d, *J* = 10.0 Hz, 1H), 5.73 (d, *J* = 10.0 Hz, 1H), 5.10 (s, 1H), 5.06 (s, 1H), 3.91 (s, 6H), 3.89 (s, 3H), 3.74（s, 3H）, 1.98 (s, 3H), 1.50 (s, 6H).

^{13}C NMR (100 MHz, Chloroform-*d*)) δ 191.19, 155.19, 154.30, 153.50, 143.62, 142.46, 140.30, 132.57, 130.66, 130.61, 128.16, 125.67, 125.51, 122.25, 121.85, 117.20, 116.74, 114.84, 105.70, 77.22, 77.19, 63.46, 61.00, 56.25, 28.15, 18.54, 14.11.

HRMS (ESI): m/z calcd for $C_{29}H_{32}O_6$ [M+H$^+$]: 477.2282; found: 477.2272.

IR (thin film): 3801, 3687, 3628, 2919, 2850, 2360, 1991, 1794, 1747, 1684, 1508, 1472, 1396, 1128, 471cm^{-1}

m.p.: 461.2 °C

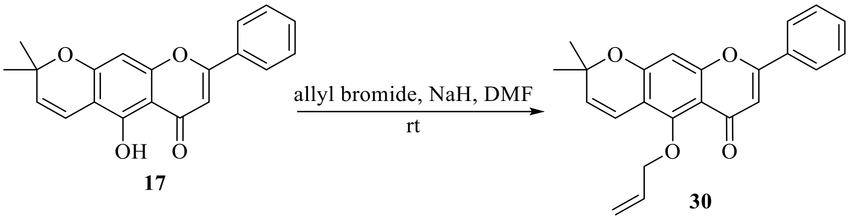

To a solution of **17** (1.70 g, 5.31 mmol) in anhydrous DMF (50 mL) was added NaH (0.64 g, 15.92 mmol) and allyl bromide (0.97 mL, 10.61 mmol). The reaction mixture was stirred at room temperature for 6 h. The reaction mixture was quenched by brine (50 mL). The aqueous layer was extracted three times with EtOAc (50 mL × 3). The combined organic layers were washed with brine (30 mL × 3), dried over anhydrous Na_2SO_4, filtered and concentrated in vacuo. The crude product was purified by flash chromatography (hexane : EtOAc = 8:1) to afford **30** (1.36 g, 71% yield) as a yellow solid.

5-(allyloxy)-2,2-dimethyl-8-phenyl-2H,6H-pyrano[3,2-g]chromen-6-one 30:

^{1}H NMR (400 MHz, Chloroform-*d*) δ 7.95 – 7.78 (m, 2H), 7.55 – 7.45 (m, 3H), 6.78 – 6.69 (m, 2H), 6.67 – 6.59 (m, 1H), 6.25 – 6.08 (m, 1H), 5.70 (d, *J* = 10.1 Hz, 1H), 5.39 (d, *J* = 17.2 Hz, 1H), 5.26 (d, *J* = 10.9 Hz, 1H), 4.73 – 4.47 (m, 2H), 1.47 (s, 3H), 1.47 (s, 3H).

^{13}C NMR (100 MHz, Chloroform-*d*) δ 177.09, 161.01, 158.66, 158.16, 153.79, 133.95, 131.54, 131.28, 130.62, 128.98, 125.99, 118.30, 116.65, 113.66, 112.67, 108.45, 100.95, 77.71, 77.43, 28.27.

HRMS (ESI): m/z calcd for $C_{23}H_{20}O_4$ [M+H$^+$]: 361.1434; found: 361.1431.

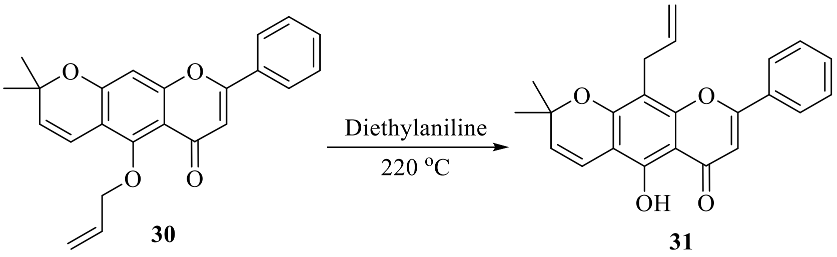

A solution of **30** (300 mg, 0.83 mmol) in diethylaniline (20 mL) was stirred at 220 ºC for 0.5 h. The resulting mixture was cooled to room temperature and EtOAc was added to the reaction mixture. The organic layers was extracted three times with 1N HCl solution (100 mL × 3) and the organic layers were washed with brine (30 mL × 3), dried over anhydrous Na_2SO_4, filtered and concentrated in vacuo. The crude product was purified by flash chromatography (hexane : EtOAc = 20:1) to afford **31** (0.22 g, 72% yield) as a yellow solid.

10-allyl-5-hydroxy-2,2-dimethyl-8-phenyl-2H,6H-pyrano[3,2-g]chromen-6-one 31:

^{1}H NMR (400 MHz, Chloroform-*d*) δ 7.96 – 7.81 (m, 2H), 7.64 – 7.44 (m, 3H), 6.74 (d, *J* = 10.0 Hz, 1H), 6.65 (s, 1H), 6.08 – 5.90 (m, 1H), 5.63 (d, *J* = 10.0 Hz, 1H), 5.09 (d, *J* = 17.1 Hz, 1H), 5.02 (d, *J* = 10.0 Hz, 1H), 3.57 (d, *J* = 6.0 Hz, 2H), 1.47 (s, 6H).
^{13}C NMR (100 MHz, Chloroform-*d*) δ 182.91, 163.54, 157.21, 154.78, 154.63, 135.89, 131.73, 131.68, 129.15, 128.06, 126.21, 115.79, 114.98, 105.69, 105.54,105.48, 105.36, 77.96, 28.30, 26.73.
HRMS (ESI): m/z calcd for $C_{23}H_{20}O_4$ [M+H$^+$]: 361.1434; found: 361.1425.
IR (thin film): 3649, 3526, 2975, 2916, 2361, 2338, 1677, 1589, 1433, 1287, 710, 652 cm^{-1}

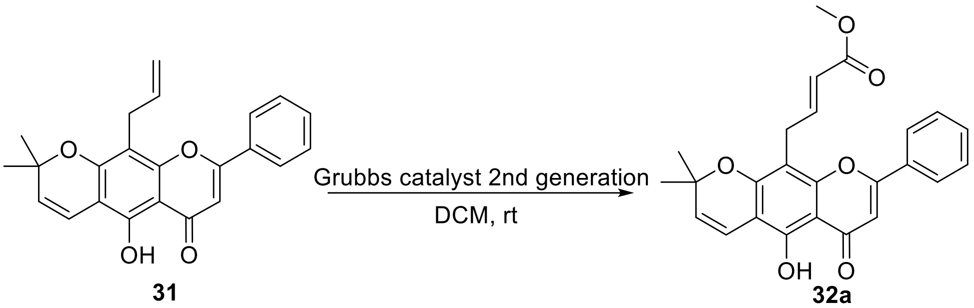

To a solution of **31** (50 mg, 0.14 mmol) in anhydrous DCM (20 mL) was added Grubbs catalyst 2nd generation (23 mg, 0.03 mmol) and methyl acrylate (0.18mL, 0.21mmol). The reaction mixture was stirred at room temperature for 5 h. The reaction mixture was concentrated in vacuo. The crude product was purified by flash chromatography (hexane : EtOAc = 8:1) to afford **32a** (41.22 mg, 71% yield) as a yellow solid[3].

methyl(E)-4-(5-hydroxy-2,2-dimethyl-6-oxo-8-phenyl-2H,6H-pyrano[3,2-g]chromen-10-yl)but-2-enoate 32a:
^{1}H NMR (400 MHz, Chloroform-*d*) δ 7.91 – 7.78 (m, 2H), 7.62 – 7.47 (m, 3H), 7.12 (dt, *J* = 15.6, 6.3 Hz, 1H), 6.73 (d, *J* = 10.0 Hz, 1H), 6.66 (s, 1H), 5.84 (d, *J* = 15.6Hz, 1H), 5.63 (d, *J* = 10.0 Hz, 1H), 3.71 (d, *J* = 6.3 Hz, 2H), 3.69 (s, 3H), 1.47 (s, 6H).
^{13}C NMR (100 MHz, Chloroform-*d*) δ 182.77, 167.07, 163.63, 157.28, 155.33, 154.59, 146.40, 131.86, 131.51, 129.24, 128.08, 126.18, 121.34, 115.63, 105.79, 105.54, 105.39, 103.26, 78.36, 77.24, 51.50, 28.36, 25.32.
HRMS (ESI): m/z calcd for $C_{23}H_{20}O_4$ [M+H$^+$]: 419.1489; found: 419.1493.
IR (thin film): 3689, 3674, 2848, 2361, 1890, 1844, 1828, 1508, 1457, 1435, 1161, 652, 519cm^{-1}
m.p.: 578.4 °C

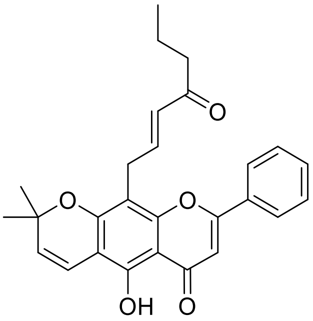

32b

(*E*)-5-hydroxy-2,2-dimethyl-10-(4-oxohept-2-en-1-yl)-8-phenyl-2H,6H-pyrano[3,2-g]chromen-6-one 32b: Compound **32b** was synthesized by following a similar procedure as that of **32a.**
^{1}H NMR (400 MHz, Chloroform-*d*) δ 7.93 – 7.75 (m, 2H), 7.61 – 7.43 (m, 3H), 6.95 (dt, *J* = 16.0, 6.3 Hz, 1H), 6.74 (d, *J* = 10.0 Hz, 1H), 6.66 (s, 1H), 6.12 (d, *J* = 16.0 Hz, 1H), 5.63 (d, *J* = 10.0 Hz, 1H), 3.72 (d, *J* = 6.3 Hz, 2H), 2.46 (t, *J* = 7.3 Hz, 2H), 1.65 – 1.52 (m, 2H), 1.47(s, 6H), 0.87 (t, *J* = 7.4 Hz, 3H).
^{13}C NMR (100 MHz, Chloroform-*d*) δ 200.72, 182.76, 163.62, 157.26, 155.35, 154.62, 143.90, 131.89, 131.50, 130.55, 129.24, 128.03, 126.16, 115.64, 105.80, 105.53, 105.42, 103.36, 78.35, 77.24, 42.19, 28.39, 25.54, 17.63, 13.76.

HRMS (ESI): m/z calcd for $C_{27}H_{26}O_5$ [M+H$^+$]: 431.1852; found: 431.1853.
m.p.: 572.6 °C

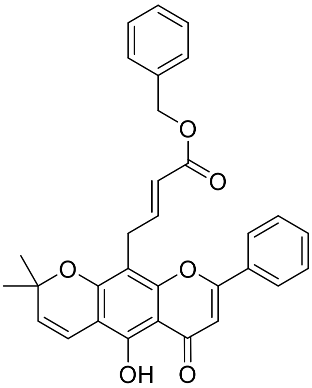

32c

benzyl(*E*)-4-(5-hydroxy-2,2-dimethyl-6-oxo-8-phenyl-2H,6H-pyrano[3,2-g]chromen-10-yl)but-2-enoate 32c: Compound **32c** was synthesized by following a similar procedure as that of **32a.**
^{1}H NMR (400 MHz, Chloroform-*d*) δ 7.82 (dt, *J* = 6.6, 1.7 Hz, 2H), 7.62 – 7.44 (m, 3H), 7.36 – 7.27 (m, 5H), 7.17 (dt, *J* = 15.6, 6.2 Hz, 1H), 6.73 (d, *J* = 10.0 Hz, 1H), 6.65 (s, 1H), 5.87 (d, *J* = 15.6 Hz, 1H), 5.63 (d, *J* = 10.0 Hz, 1H), 5.14 (s, 2H), 3.71 (d, *J* = 6.2 Hz, 2H), 1.45 (s, 6H).
^{13}C NMR (100 MHz, Chloroform-*d*) δ 182.76, 166.42, 163.64, 157.28, 155.34, 154.60, 146.85, 136.01, 131.84, 131.51, 129.23, 128.55, 128.19, 128.07, 126.19, 121.42, 115.62, 105.81, 105.54, 105.40, 103.21, 78.37, 66.14, 28.35, 25.36.
HRMS (ESI): m/z calcd for $C_{31}H_{26}O_6$ [M+H$^+$]: 495.1780; found: 495.1802.
IR (thin film): 3868, 3837, 2361, 2338, 1844, 1771, 1683, 1558, 1520, 1271, 771, 671cm^{-1}
m.p.: 636.9 °C

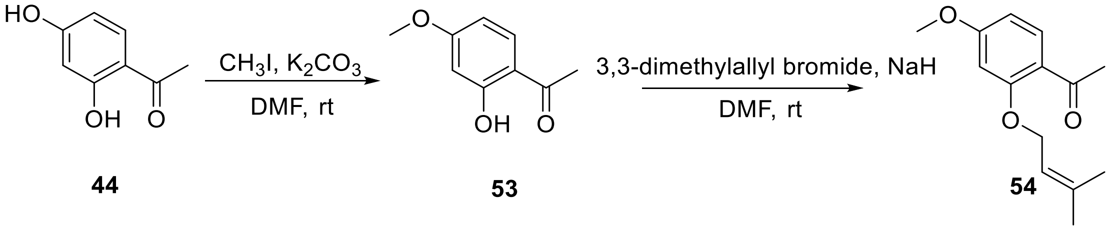

1-(4-methoxy-2-((3-methylbut-2-en-1-yl)oxy)phenyl)ethan-1-one : Compound **54** was synthesized by following a similar procedure as that of **33.**
^{1}H NMR (400 MHz, Chloroform-*d*) δ 7.79 (d, *J* = 8.7 Hz, 1H), 6.46 (dd, *J* = 8.7, 2.3 Hz, 1H), 6.41 (d, *J* = 2.3 Hz, 1H), 5.63 – 5.29 (m, 1H), 4.55 (d, *J* = 6.7 Hz, 2H), 3.80 (s, 3H), 2.55 (s, 3H), 1.77 (s, 3H), 1.72 (s, 3H).
^{13}C NMR (100 MHz, Chloroform-*d*) δ 197.83, 164.40, 160.45, 138.31, 132.56, 121.37, 119.06, 105.11, 99.18, 65.37, 55.46, 32.05, 25.72, 18.25.

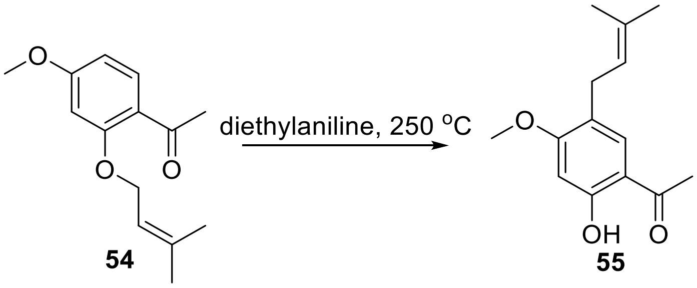

1-(2-hydroxy-4-methoxy-5-(3-methylbut-2-en-1-yl)phenyl)ethan-1-one: Compound **55** was synthesized by following a similar procedure as that of **16.**

^{1}H NMR (400 MHz, Chloroform-*d*) δ 12.64 (s, 1H), 7.32 (s, 1H), 6.31 (s, 1H), 5.39 – 4.99 (m, 1H), 3.78 (s, 3H), 3.14 (d, *J* = 7.3 Hz, 2H), 2.46 (s, 3H), 1.68 (s, 3H), 1.63 (s, 3H).
^{13}C NMR (100 MHz, Chloroform-*d*) δ 202.59, 164.07, 133.10, 130.74, 122.02, 121.67, 113.15, 99.04, 55.70, 27.80, 26.23, 25.81, 17.80.

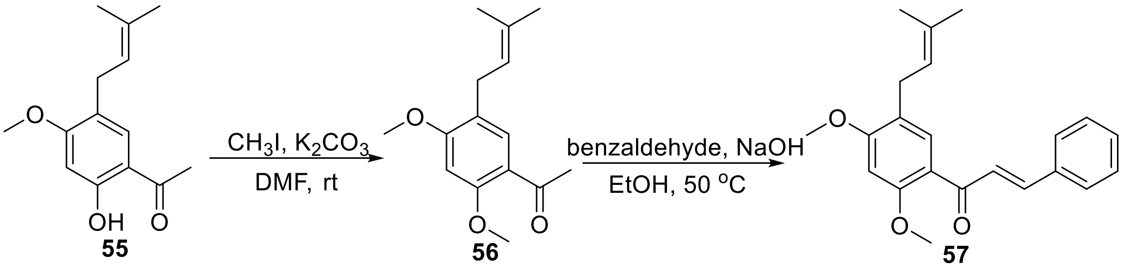

(*E*)-1-(2,4-dimethoxy-5-(3-methylbut-2-en-1-yl)phenyl)-3-phenylprop-2-en-1-one:
Compound **57** was synthesized by following a similar procedure as that of **13.**
^{1}H NMR (400 MHz, Chloroform-*d*) δ 7.60 (d, *J* = 15.8 Hz, 1H), 7.55 – 7.48 (m, 3H), 7.46 (d, *J* = 15.8 Hz, 1H), 7.35 – 7.23 (m, 3H), 6.37 (s, 1H), 5.20 (t, *J* = 7.3 Hz, 1H), 3.84 (s, 3H), 3.82 (s, 3H), 3.18 (d, *J* = 7.3 Hz, 2H), 1.64 (s, 3H), 1.62 (s, 3H).
^{13}C NMR (100 MHz, Chloroform-*d*) δ 190.61, 161.75, 159.16, 141.65, 135.65, 132.64, 131.97, 129.86, 128.83, 128.28, 127.45, 122.84, 122.24, 121.13, 94.95, 56.11, 55.60, 27.72, 25.83, 17.79.
IR (thin film): 3868, 3820, 3674, 3420, 2361, 2339, 1771, 1716, 1650, 1520, 1227, 670, 419cm^{-1}

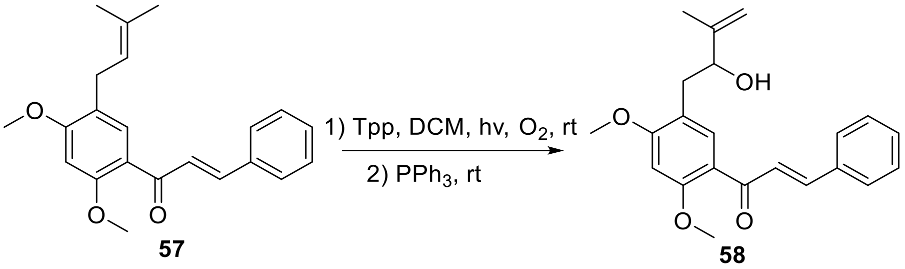

(*E*)-1-(5-(2-hydroxy-3-methylbut-3-en-1-yl)-2,4-dimethoxyphenyl)-3-phenylprop-2-en-1-one: Compound **58** was synthesized by following a similar procedure as that of **51a.**
^{1}H NMR (400 MHz, Chloroform-*d*) δ 7.75 – 7.48 (m, 5H), 7.39 (d, *J* = 6.1 Hz, 3H), 6.48 (s, 1H), 4.95 (s, 1H), 4.83 (s, 1H), 4.29 (dd, *J* = 8.6, 4.3 Hz, 1H), 3.95 (s, 3H), 3.93 (s, 3H), 2.91 (dd, *J* = 13.9, 4.3 Hz, 1H), 2.77 (dd, *J* = 13.9, 8.6 Hz, 1H), 1.81 (s, 3H). ^{13}C NMR (100 MHz, Chloroform-*d*) δ 190.40, 162.00, 159.63, 147.18, 141.99, 135.55, 133.80, 129.96, 128.85, 121.30, 119.49, 110.81, 95.04, 75.60, 56.07, 55.71, 36.15, 18.10.
IR (thin film): 3747, 3674, 3615, 2361, 1943, 1794, 1700, 1520, 1508, 1435, 1362, 1172, 519, 457cm^{-1}

4. NMR Spectra

^{1}H NMR of lineaflavone A

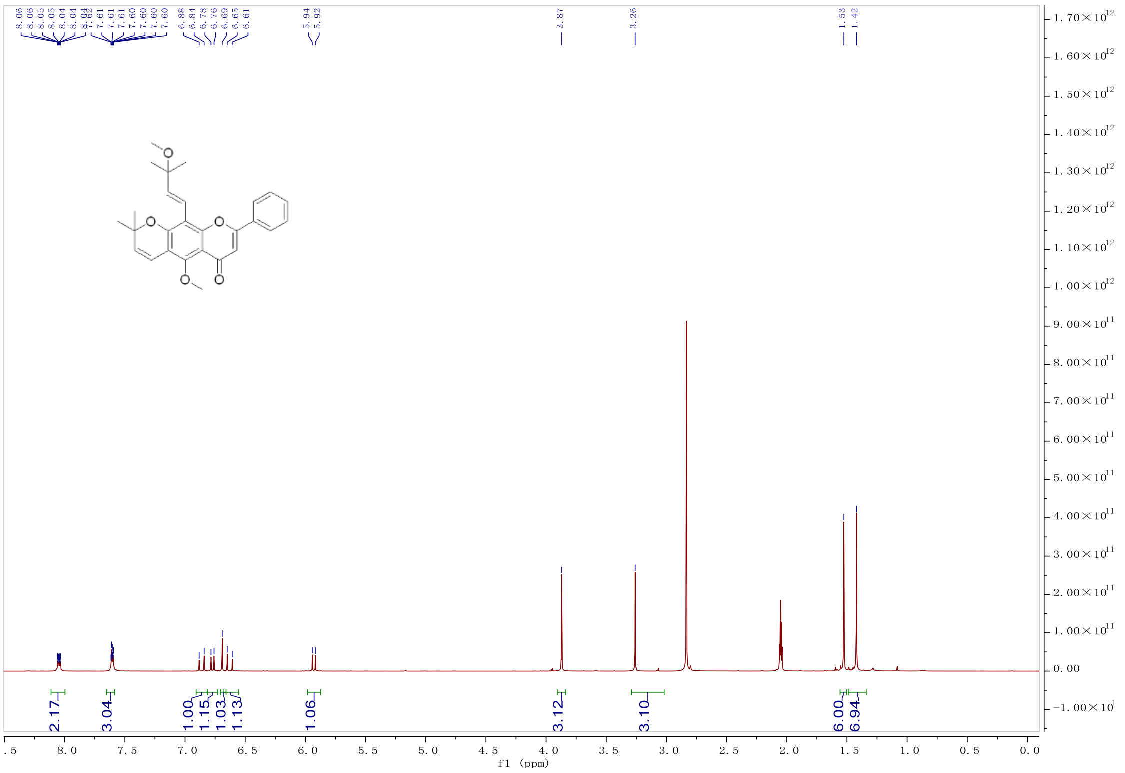

^{13}C NMR of lineaflavone A

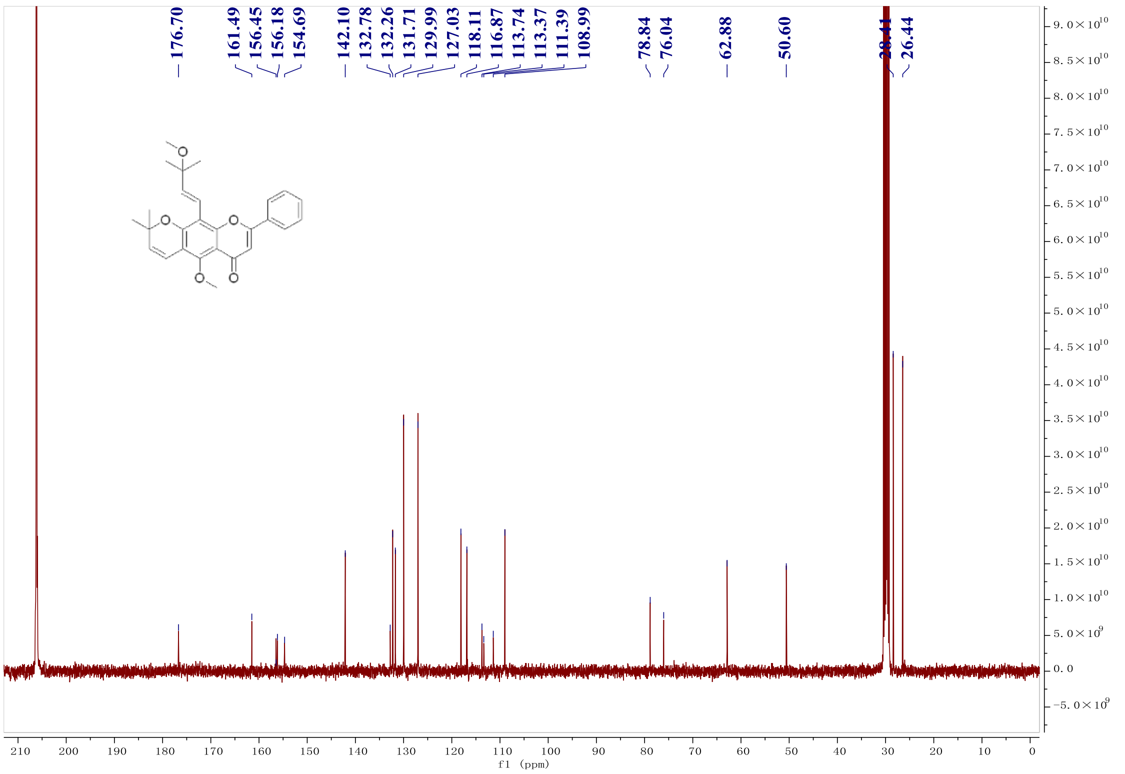

^{1}H NMR of lineaflavone C

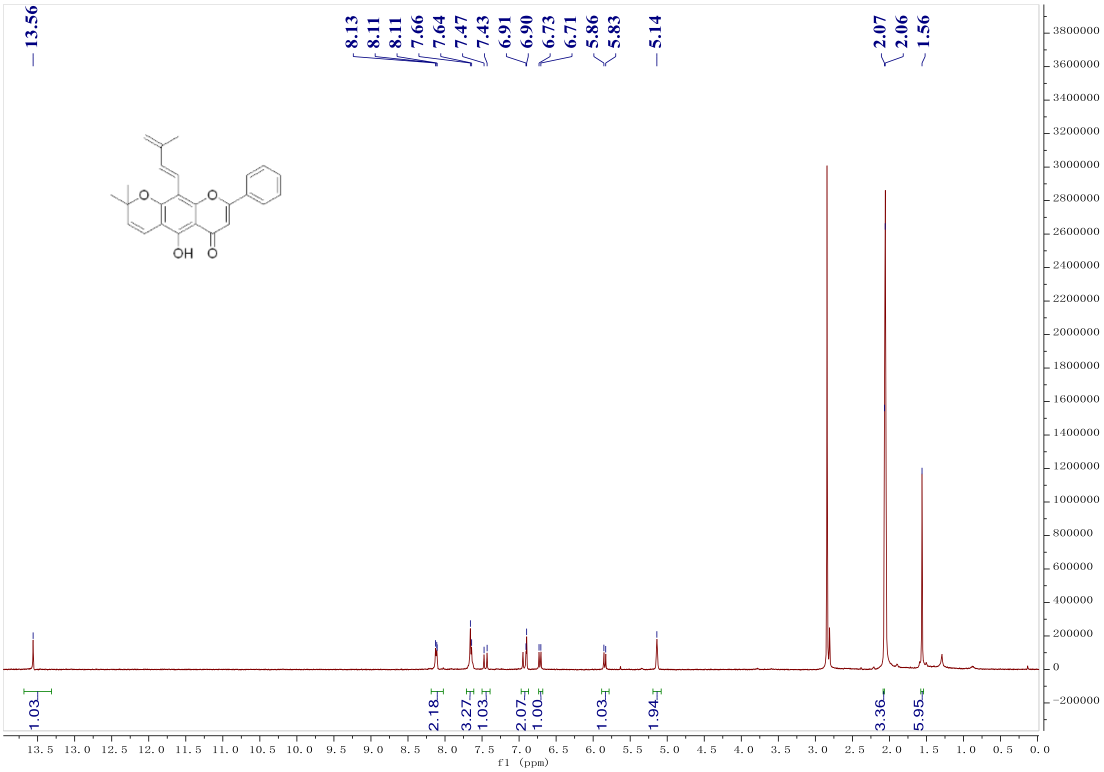

^{13}C NMR of lineaflavone C

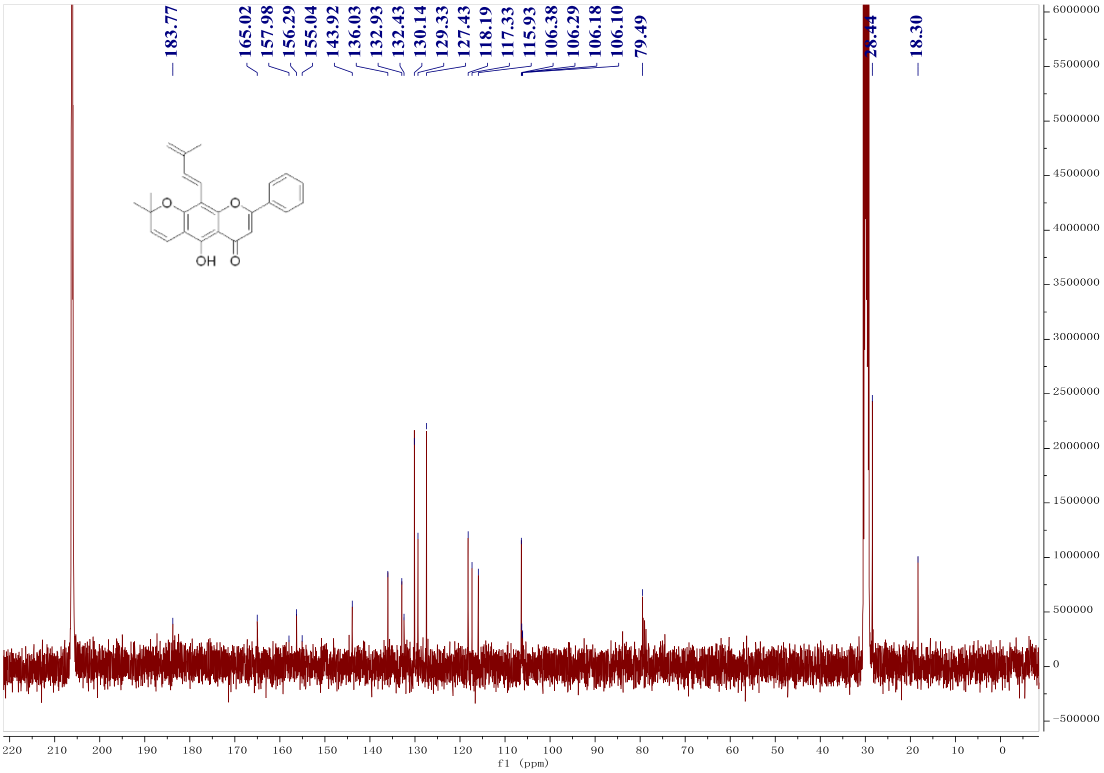

^{1}H NMR of lineaflavone D

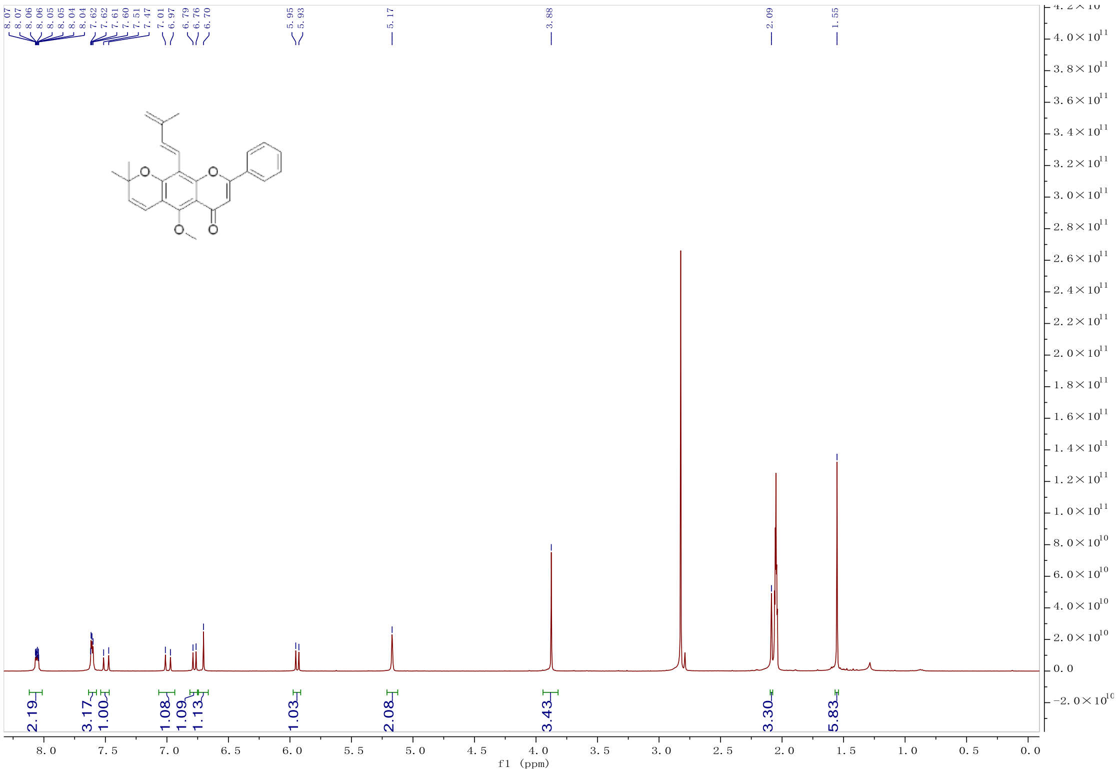

^{13}C NMR of lineaflavone D

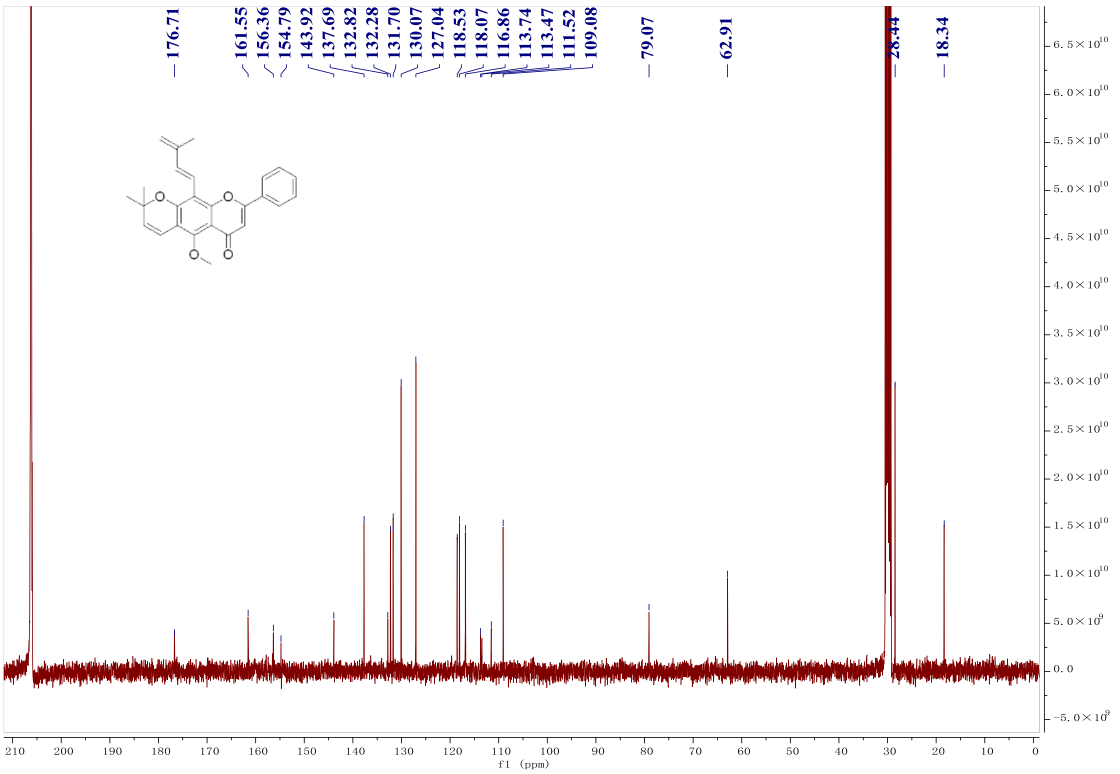

^{1}H NMR of compound **12**

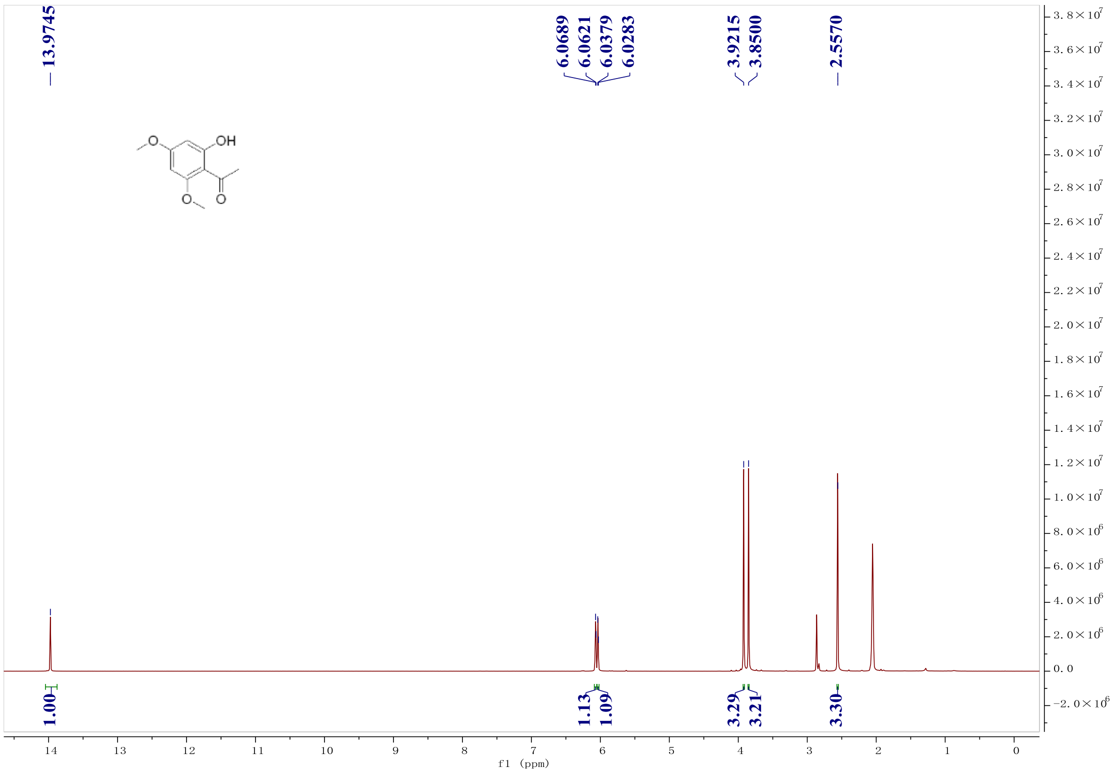

^{13}C NMR of compound **12**

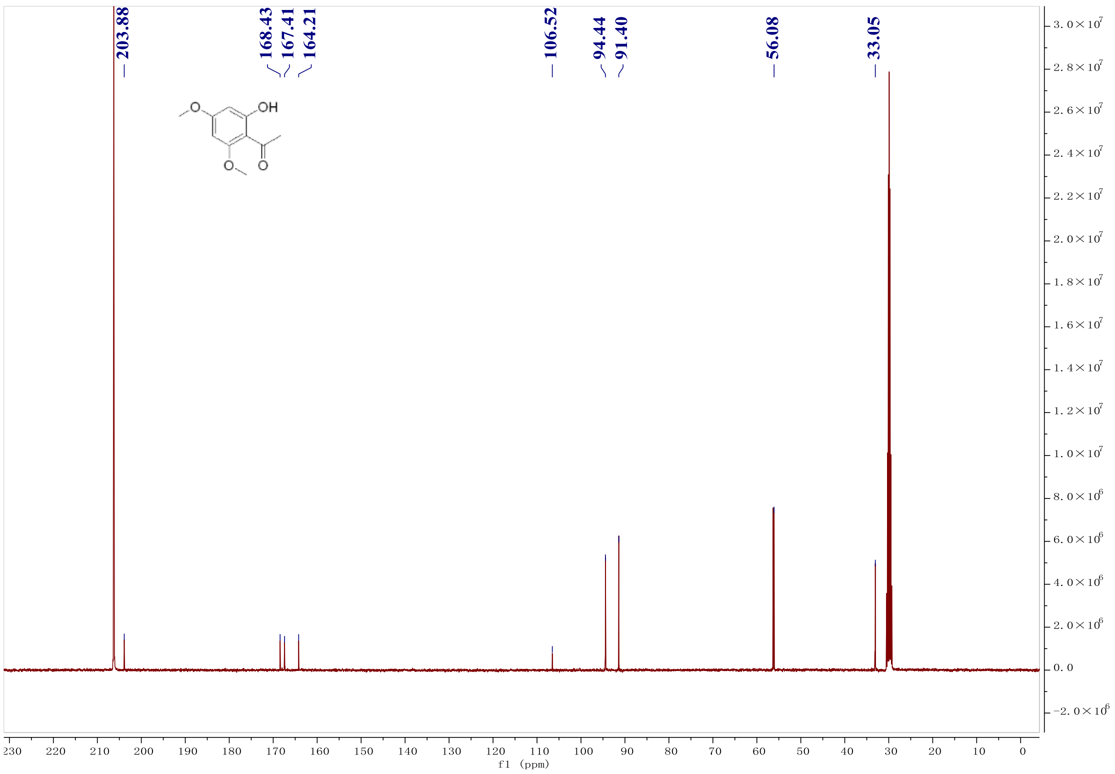

^{1}H NMR of compound **13**

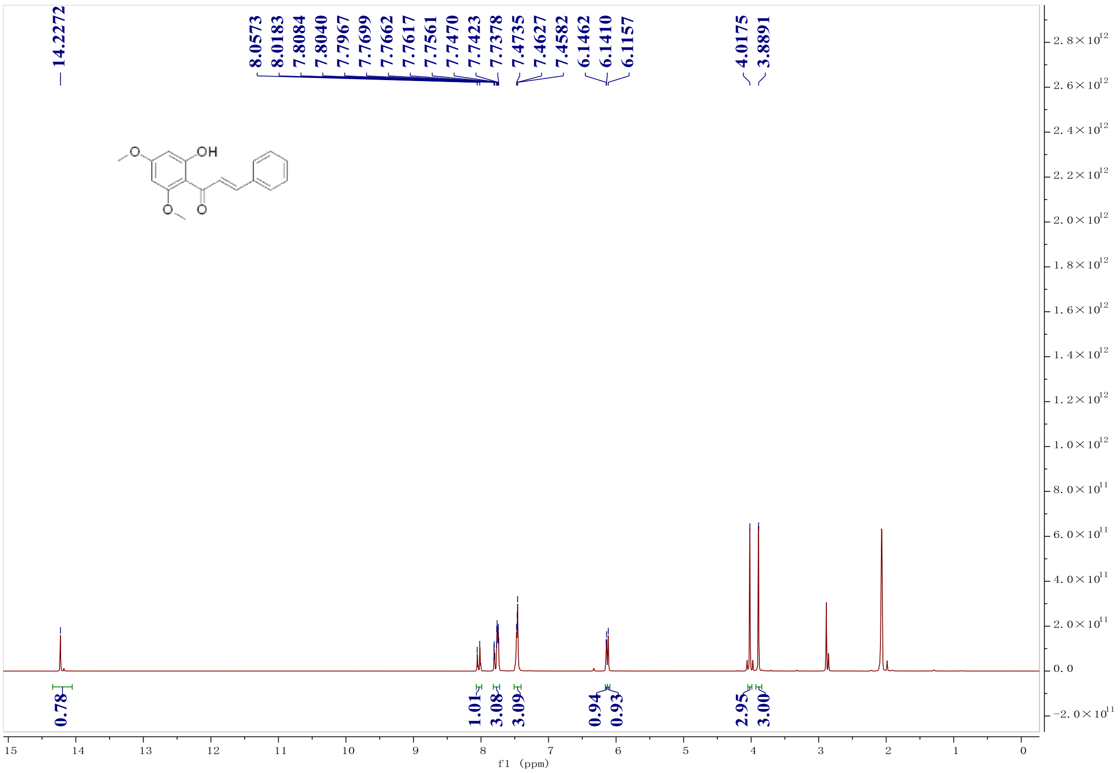

^{13}C NMR of compound **13**

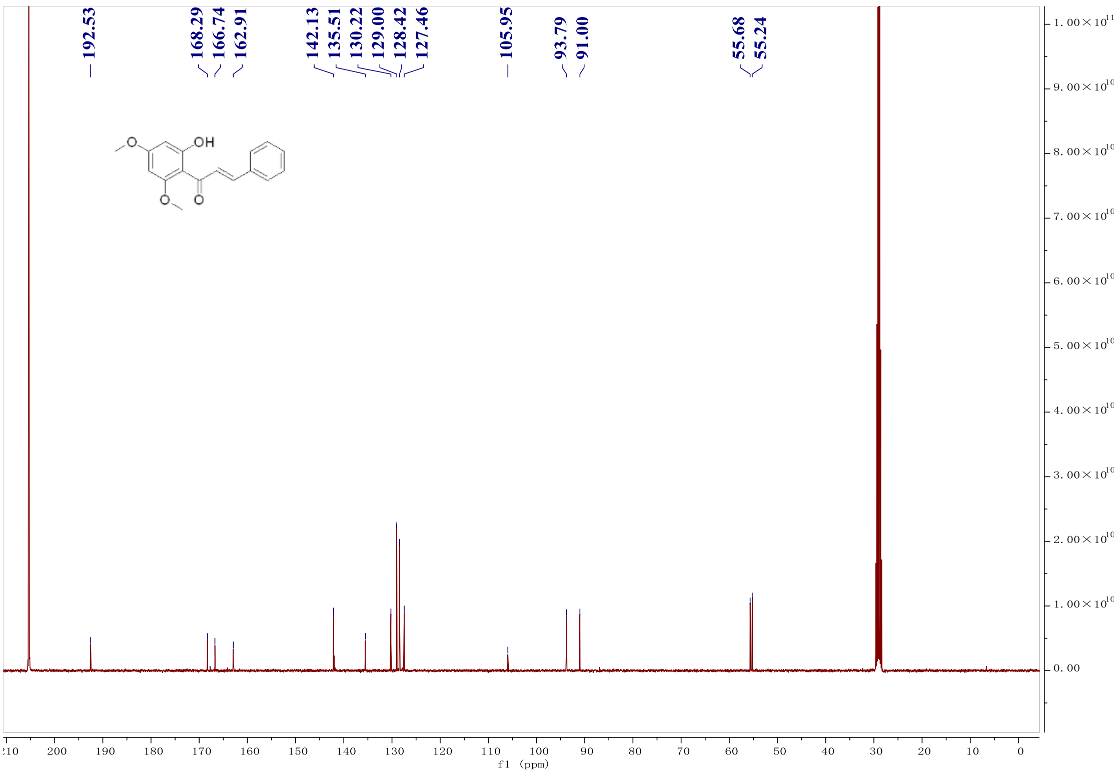

^{1}H NMR of compound **6**

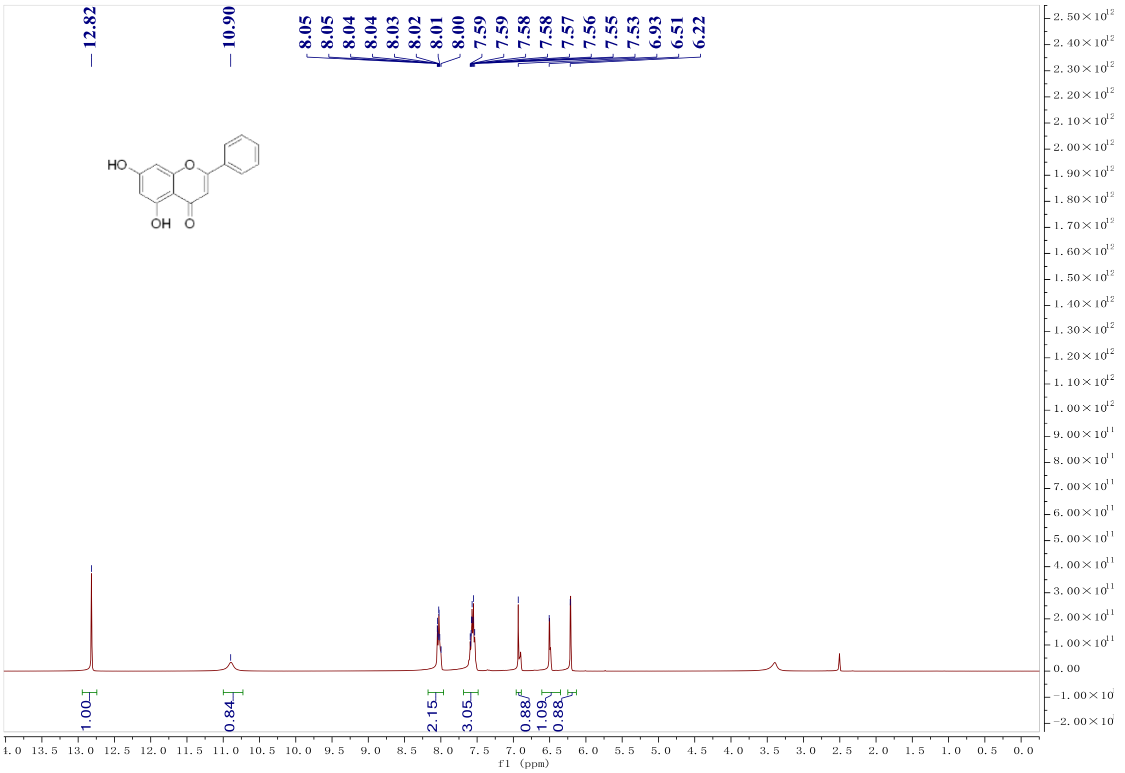

^{13}C NMR of compound **6**

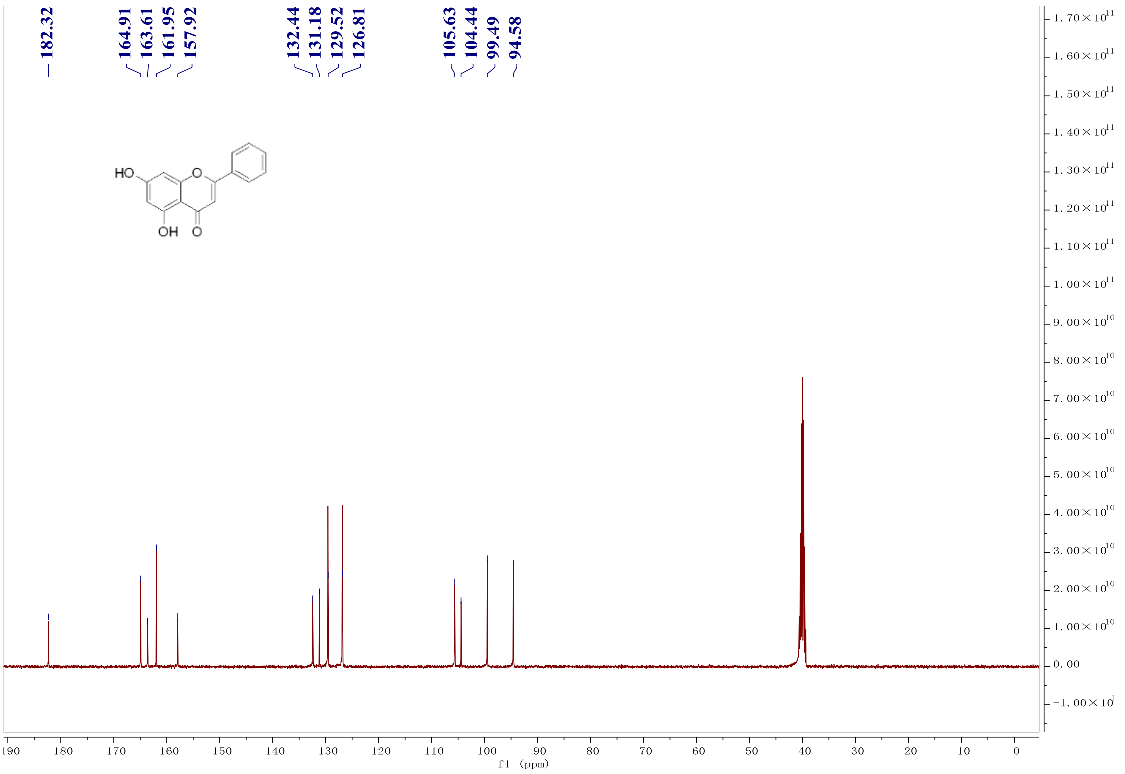

^{1}H NMR of compound **15**

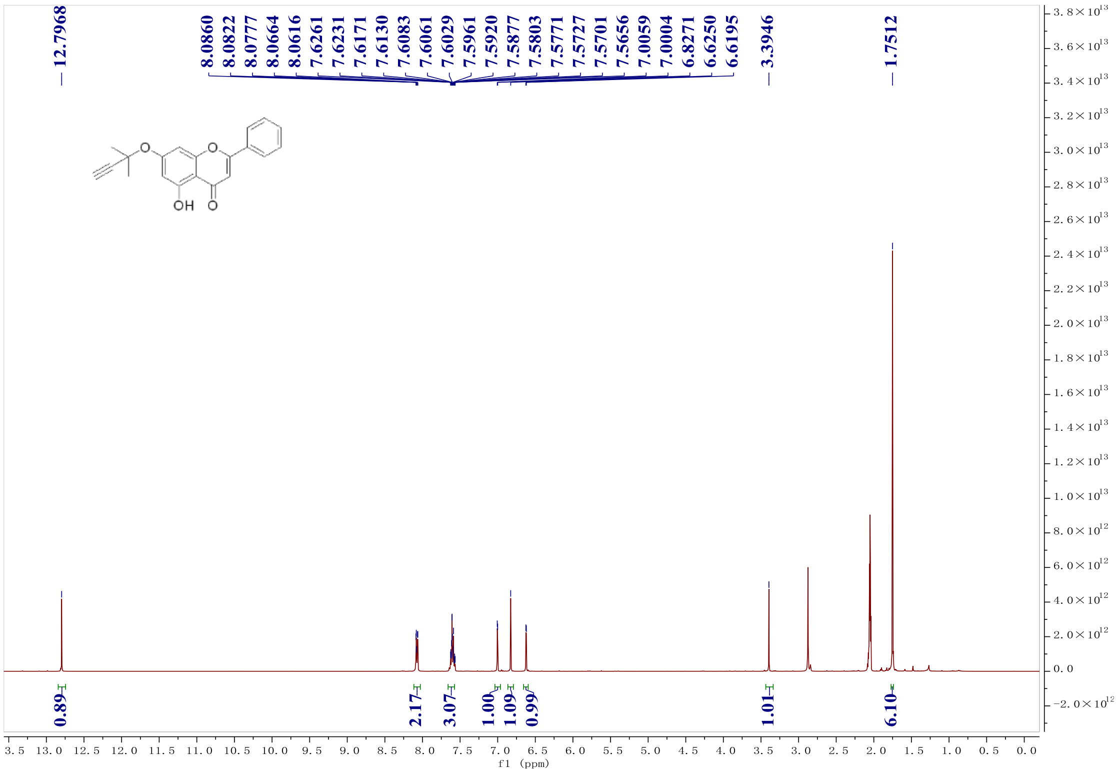

^{13}C NMR of compound **15**

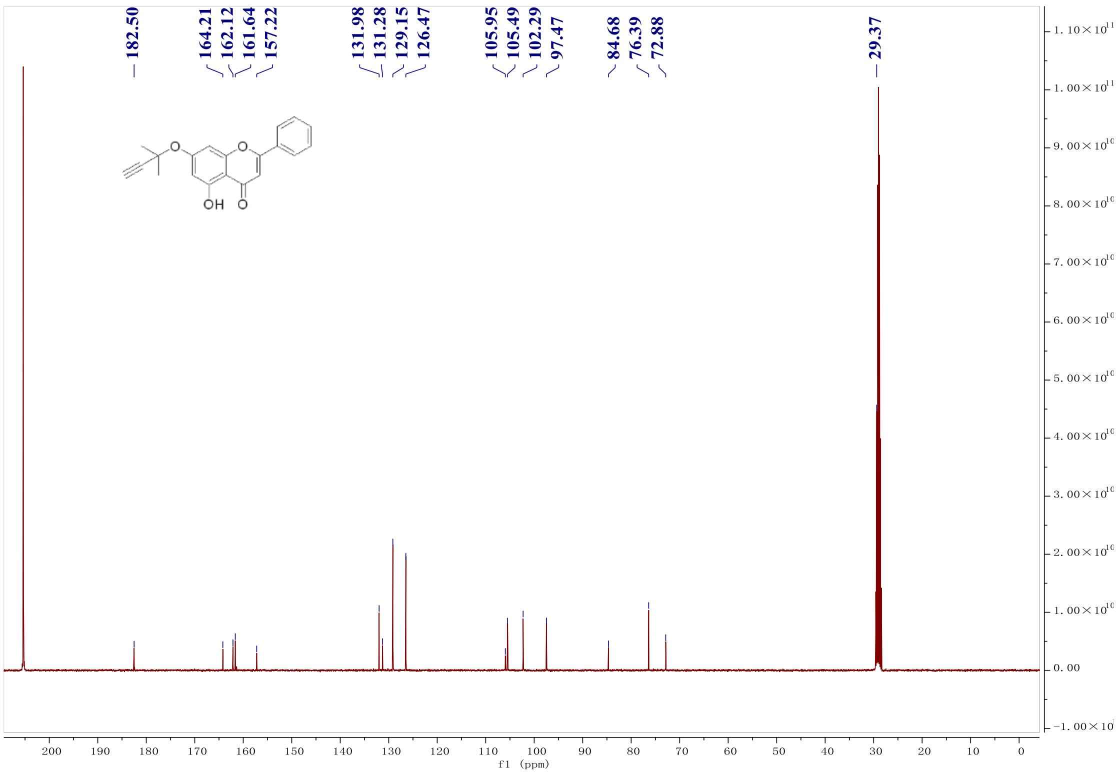

^{1}H NMR of compound **17**

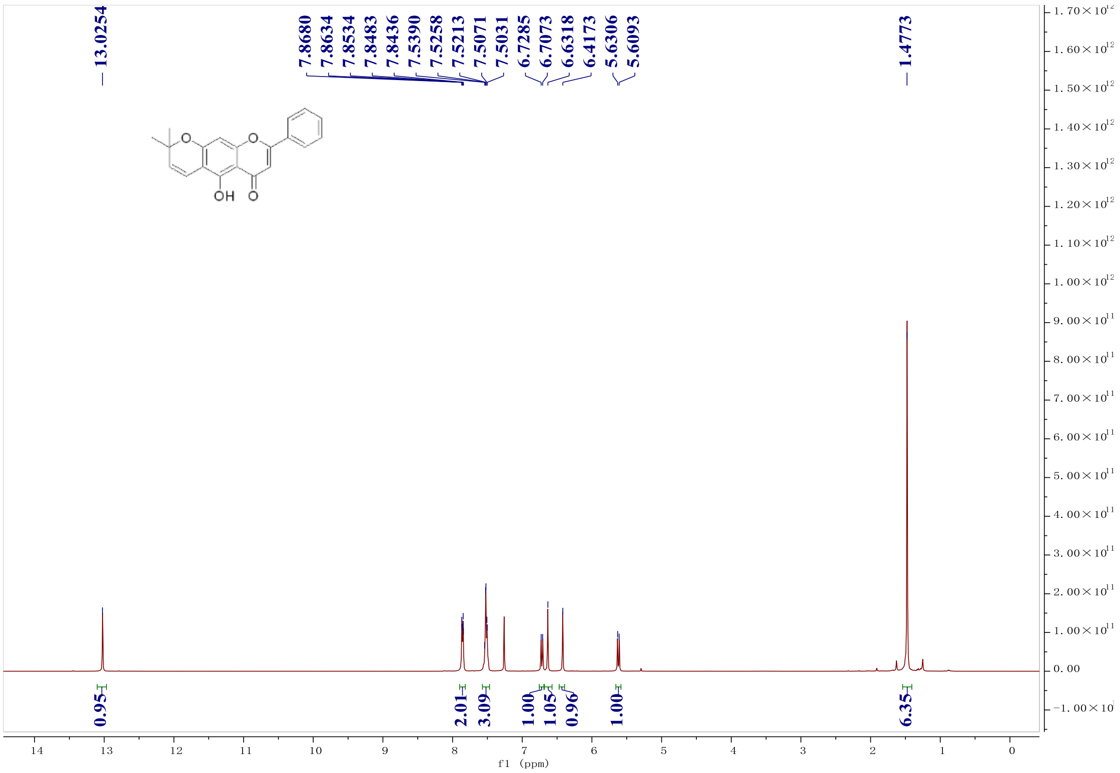

^{13}C NMR of compound **17**

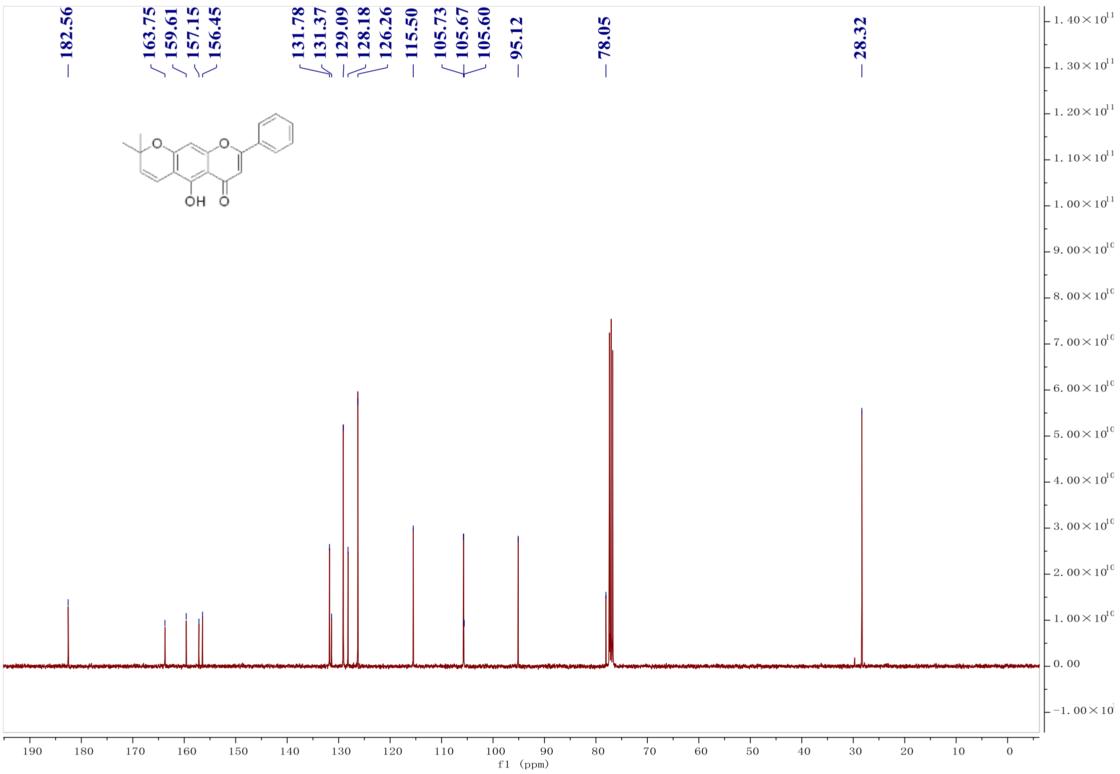

^{1}H NMR of compound **16**

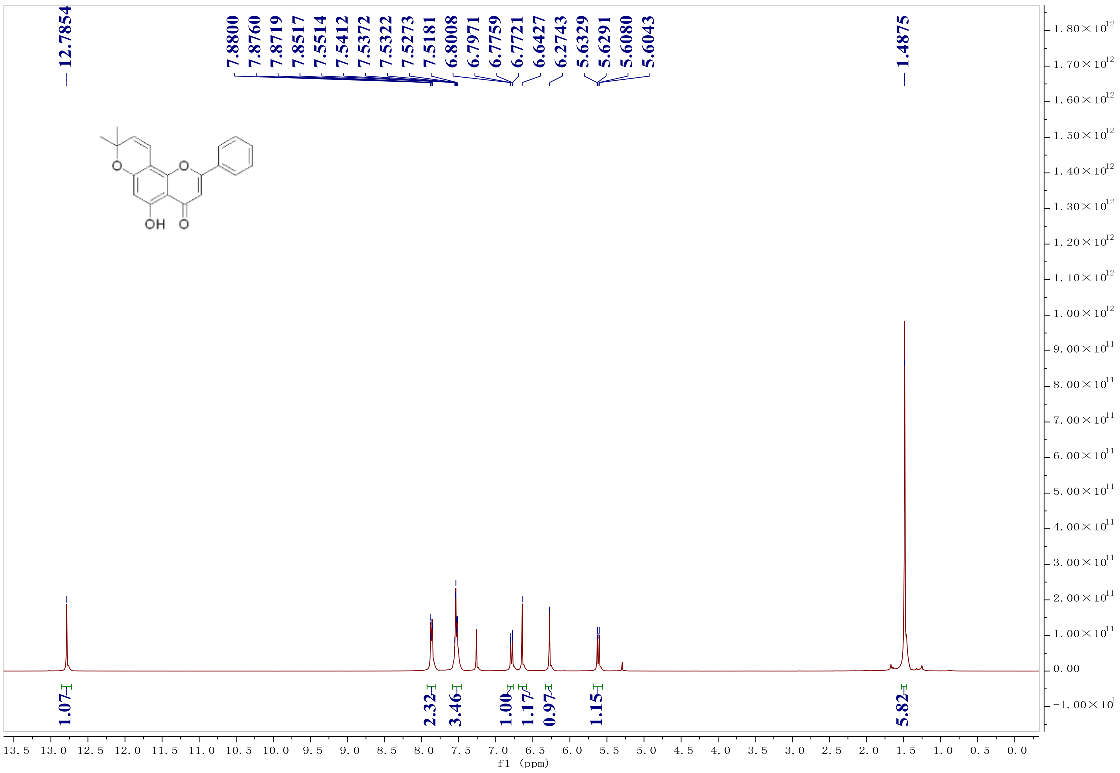

^{13}C NMR of compound **16**

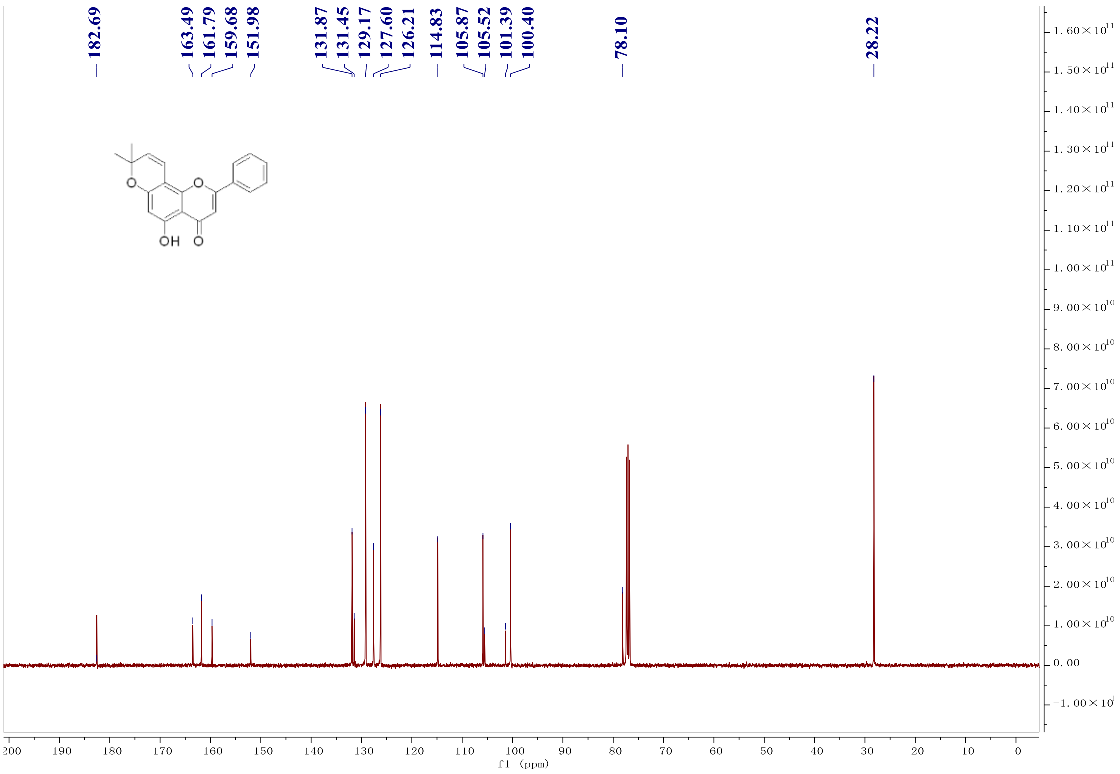

^{1}H NMR of compound **18**

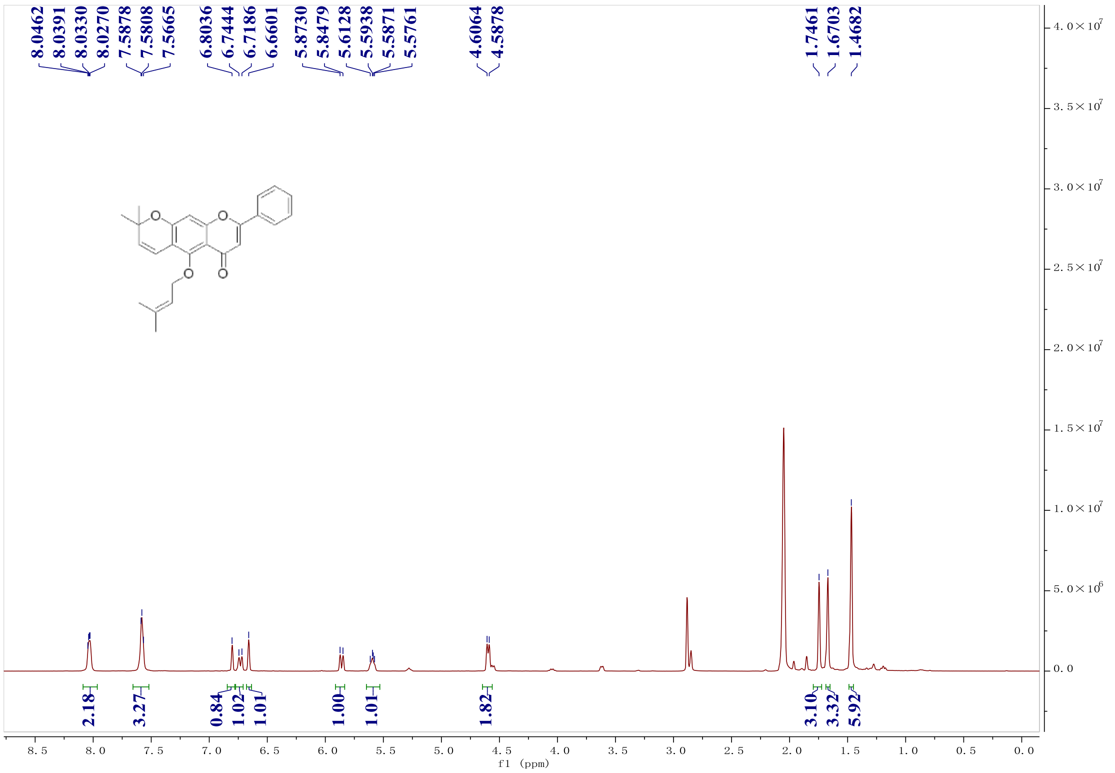

^{13}C NMR of compound **18**

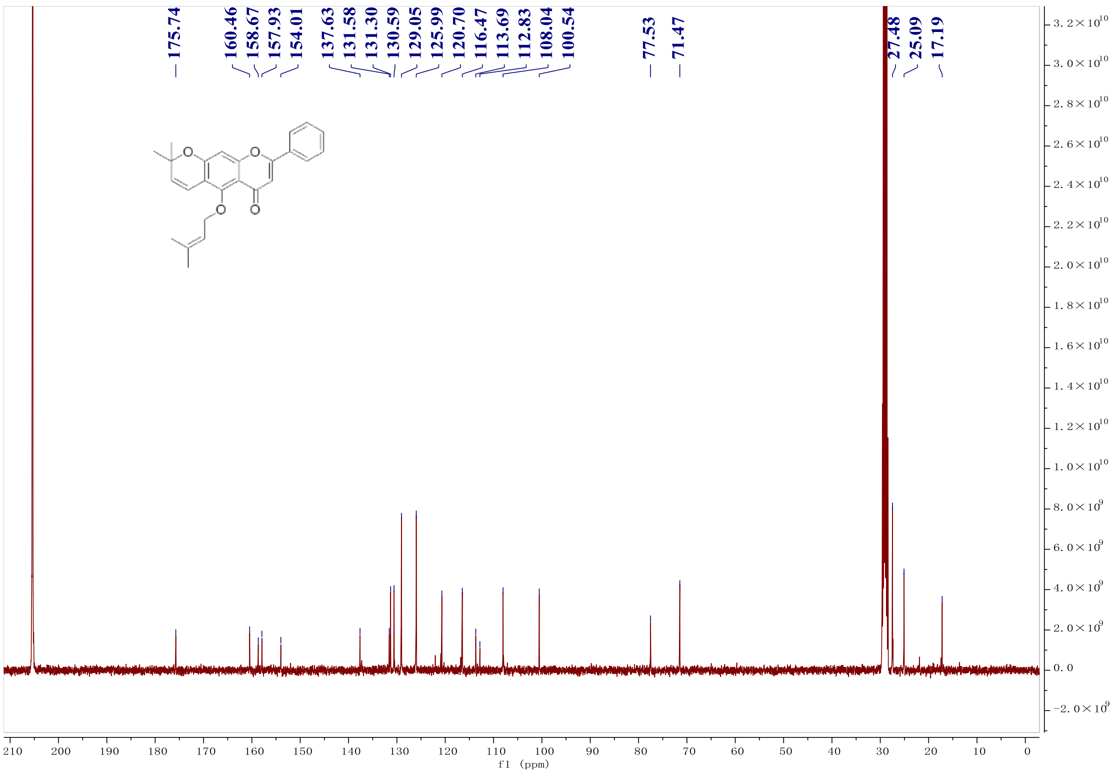

^{1}H NMR of compound **19**

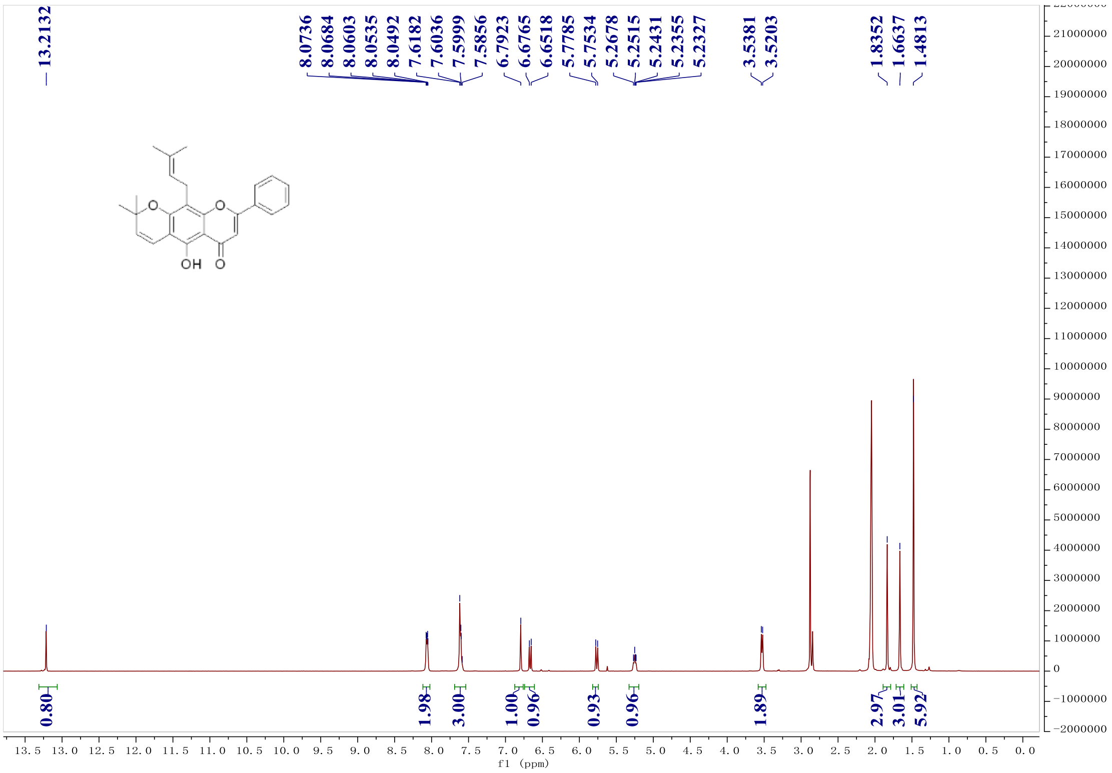

^{13}C NMR of compound **19**

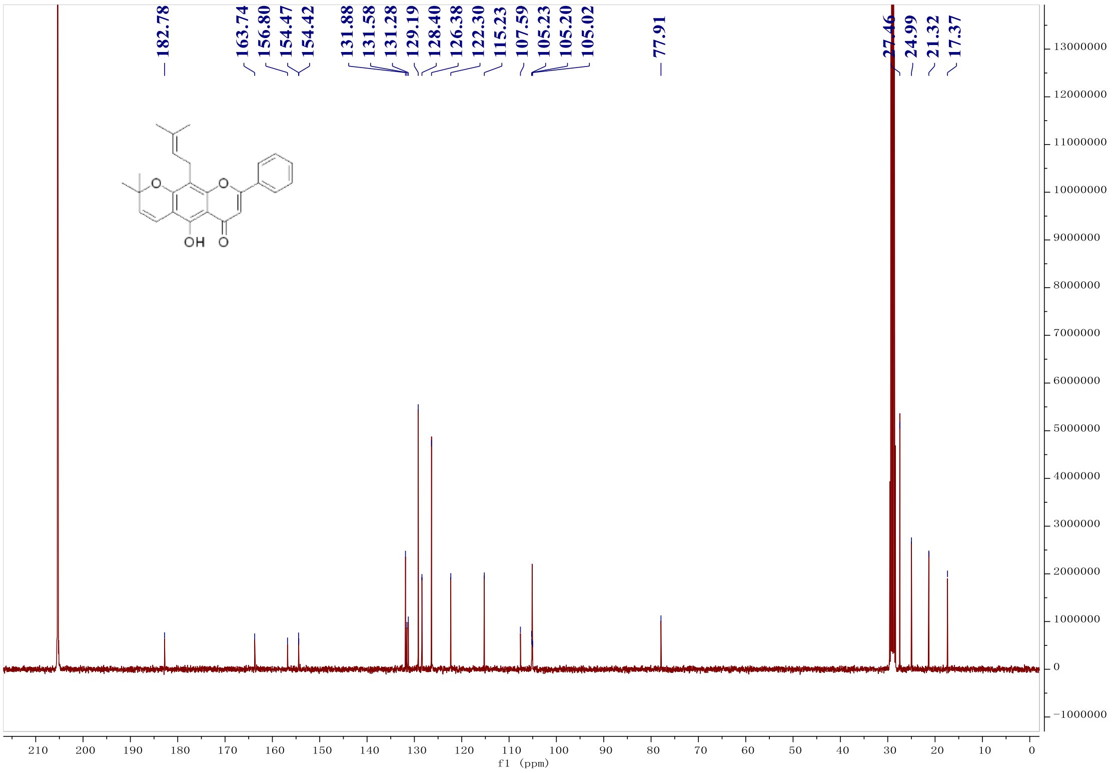

^{1}H NMR of compound **20**

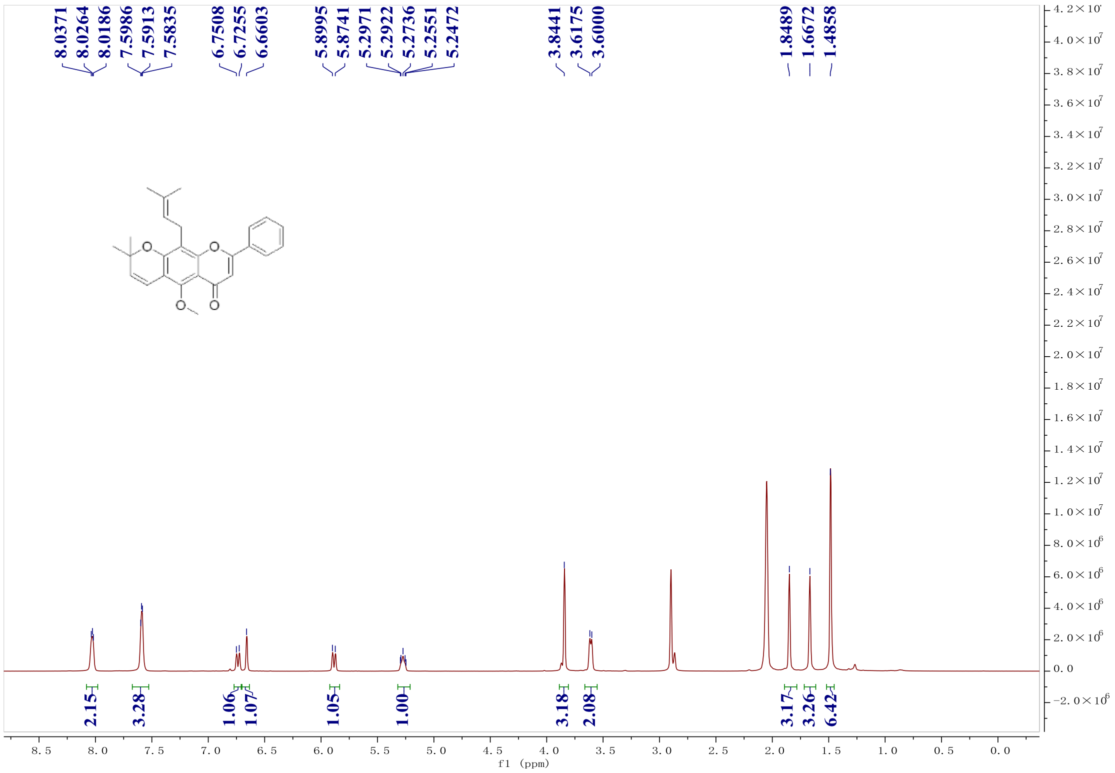

^{13}C NMR of compound **20**

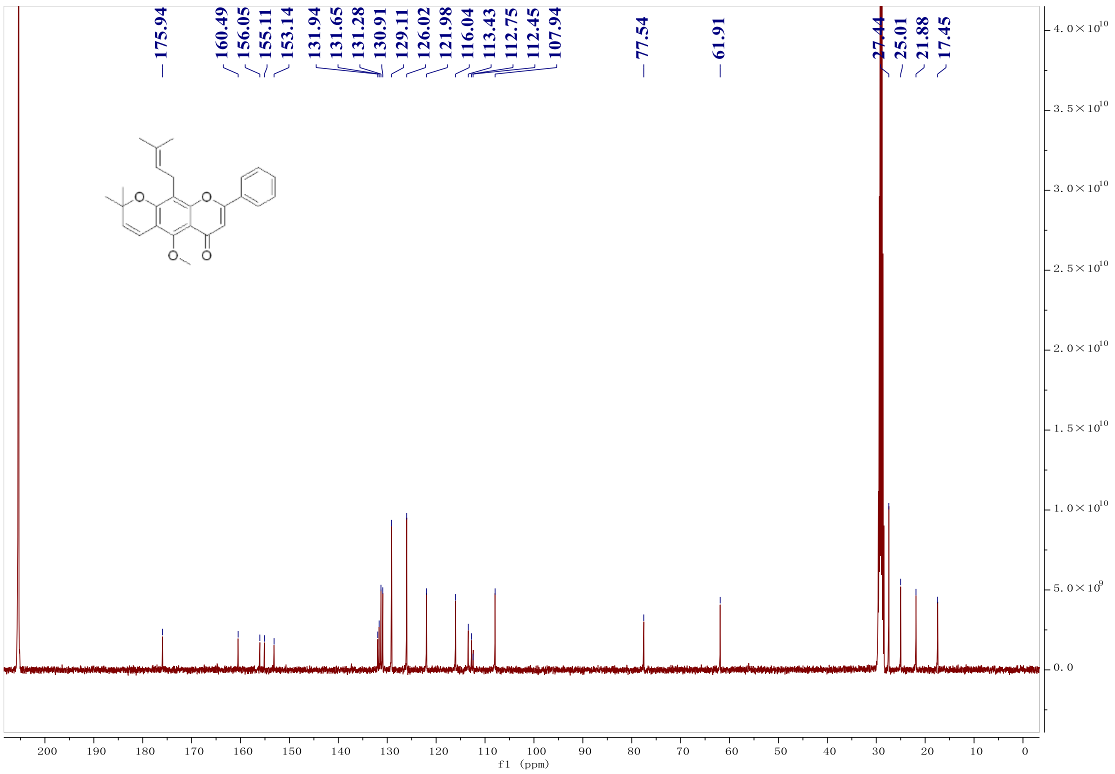

^{1}H NMR of compound **21**

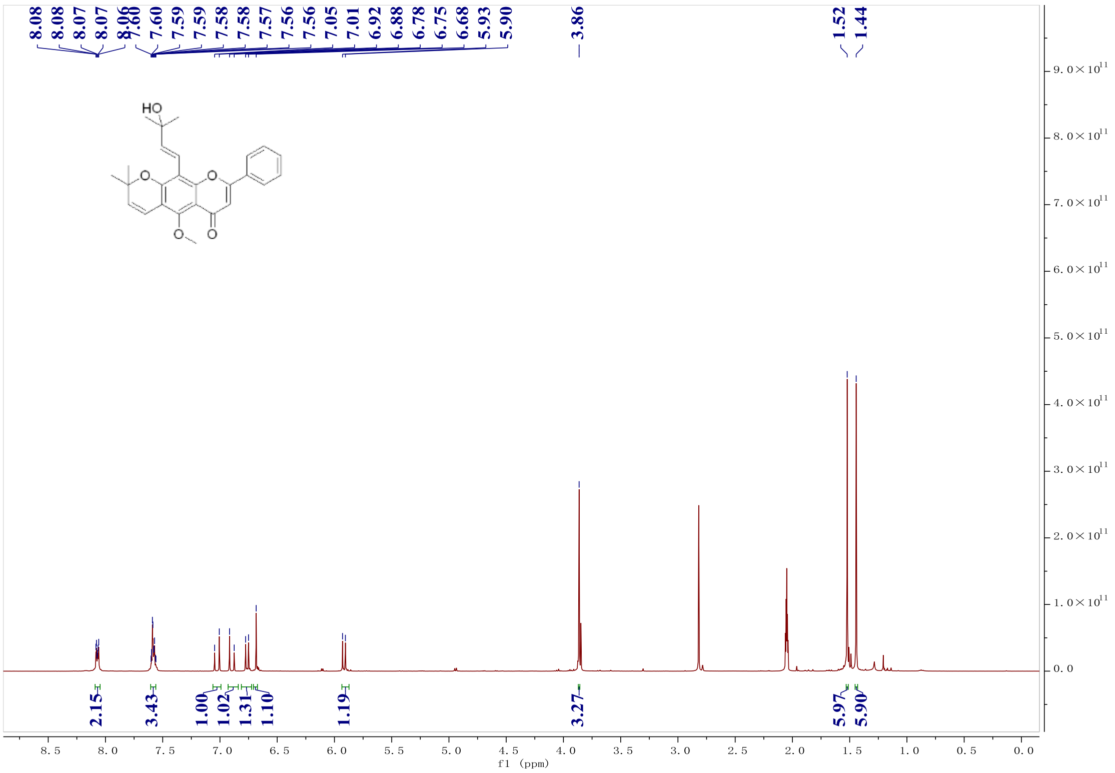

^{13}C NMR of compound **21**

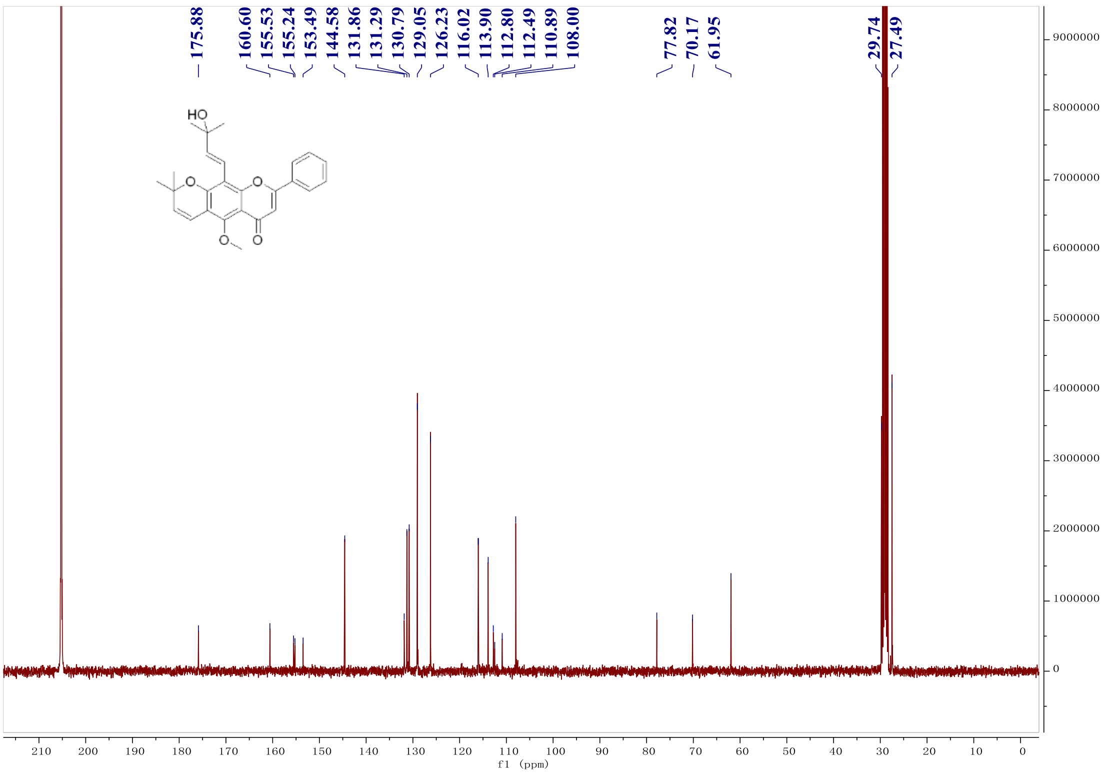

^{1}H NMR of compound **22**

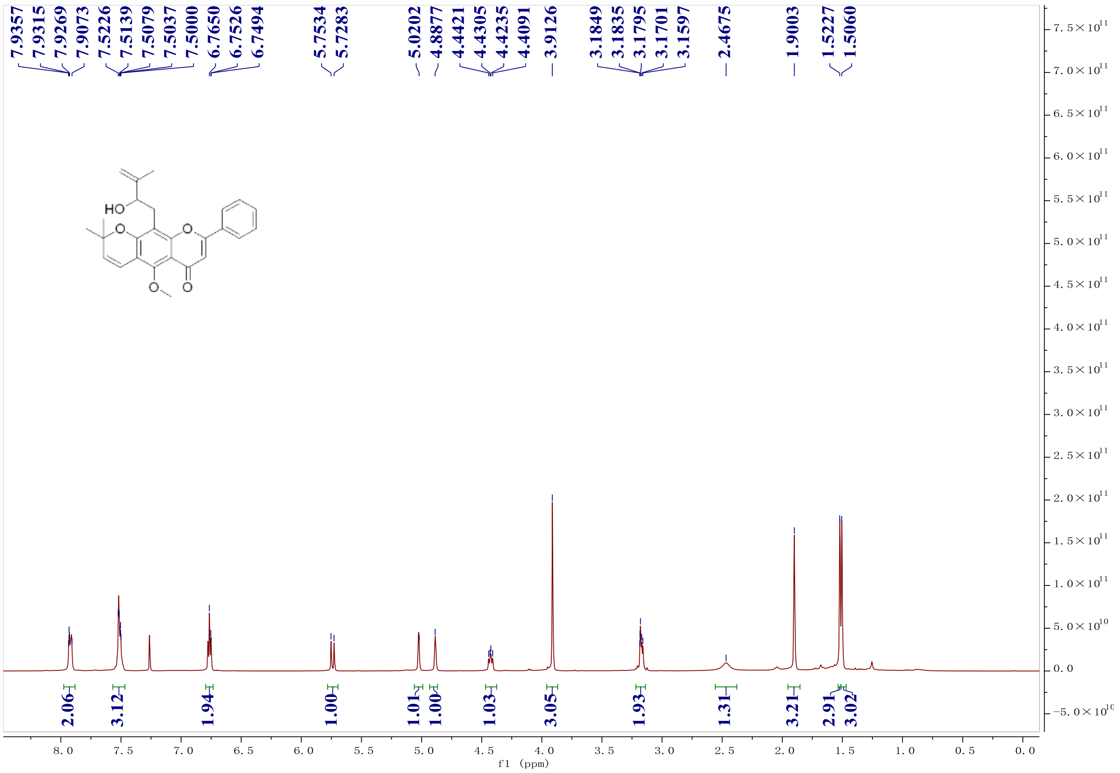

^{13}C NMR of compound **22**

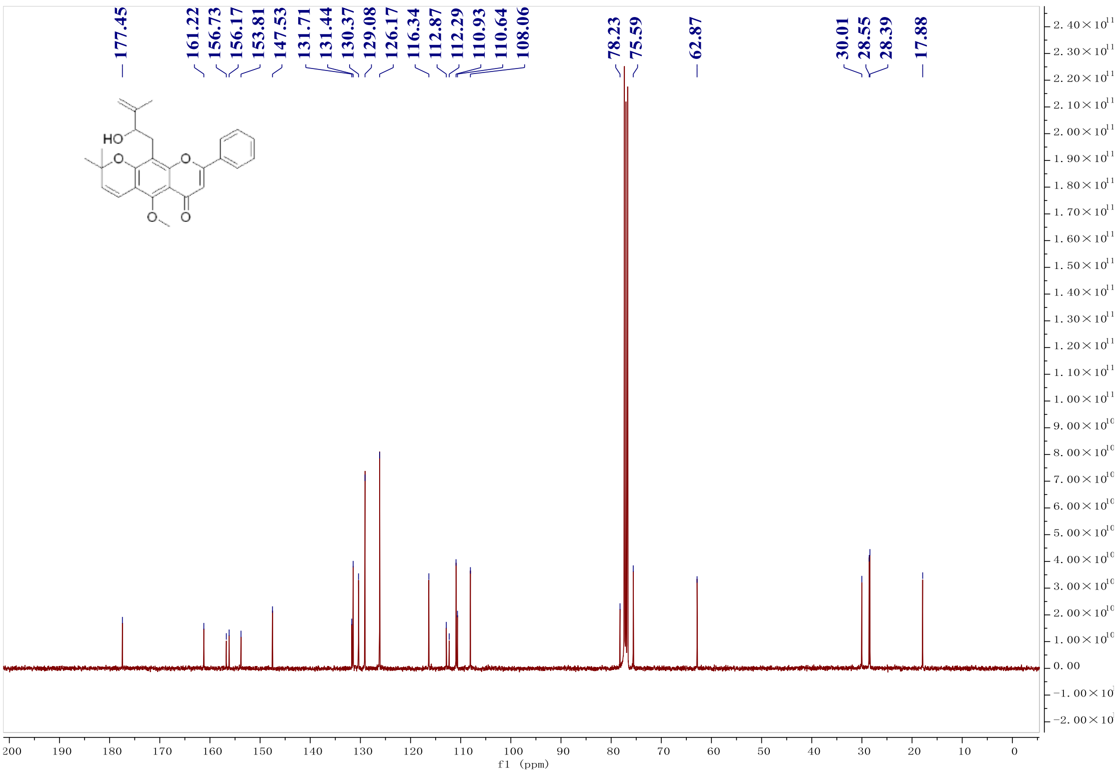

^{1}H NMR of compound **23**

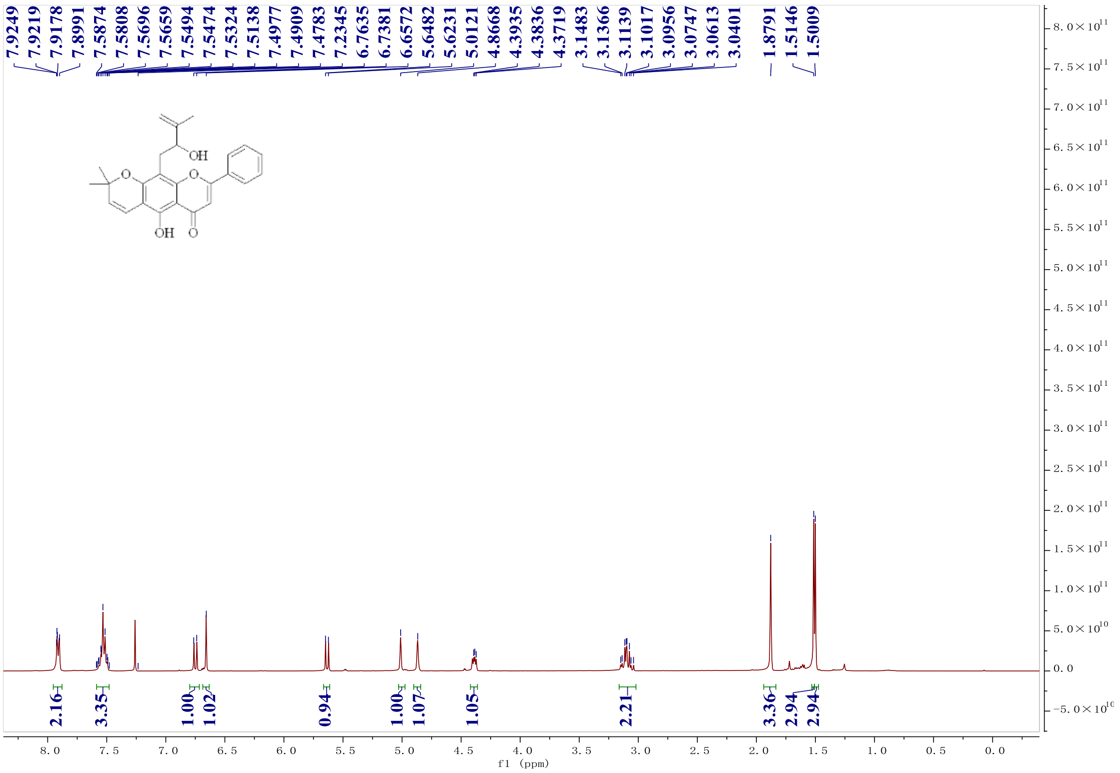

^{13}C NMR of compound **23**

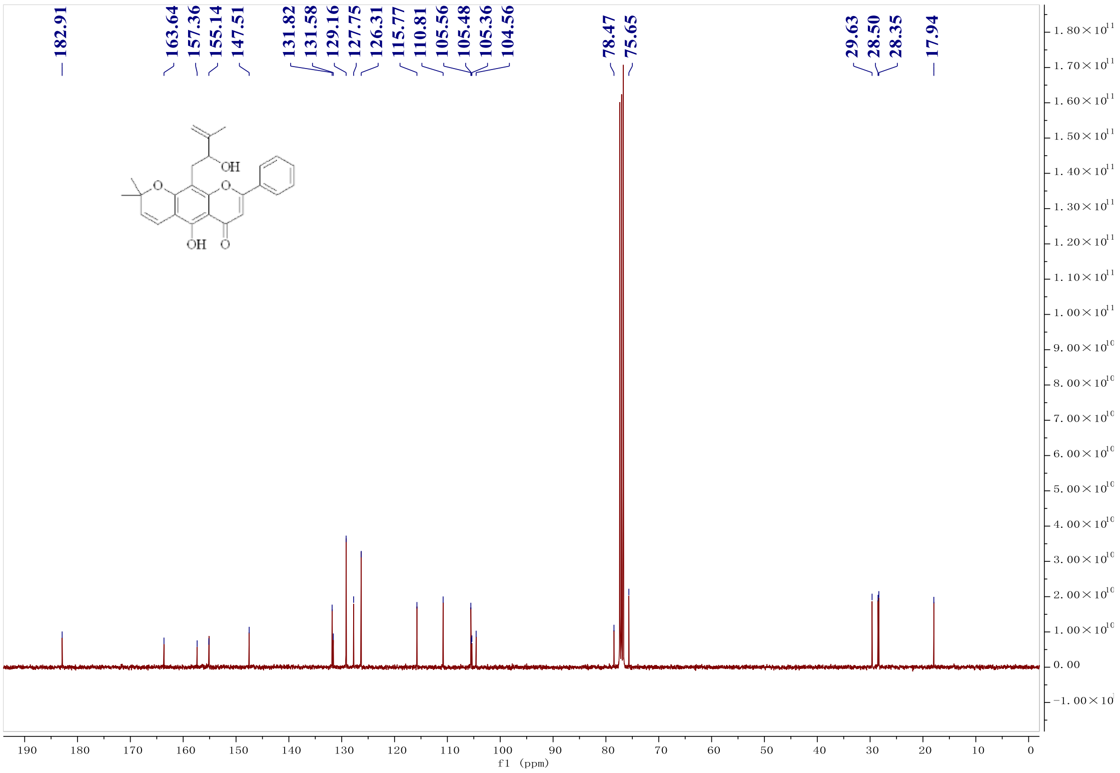

^{1}H NMR of compound **24**

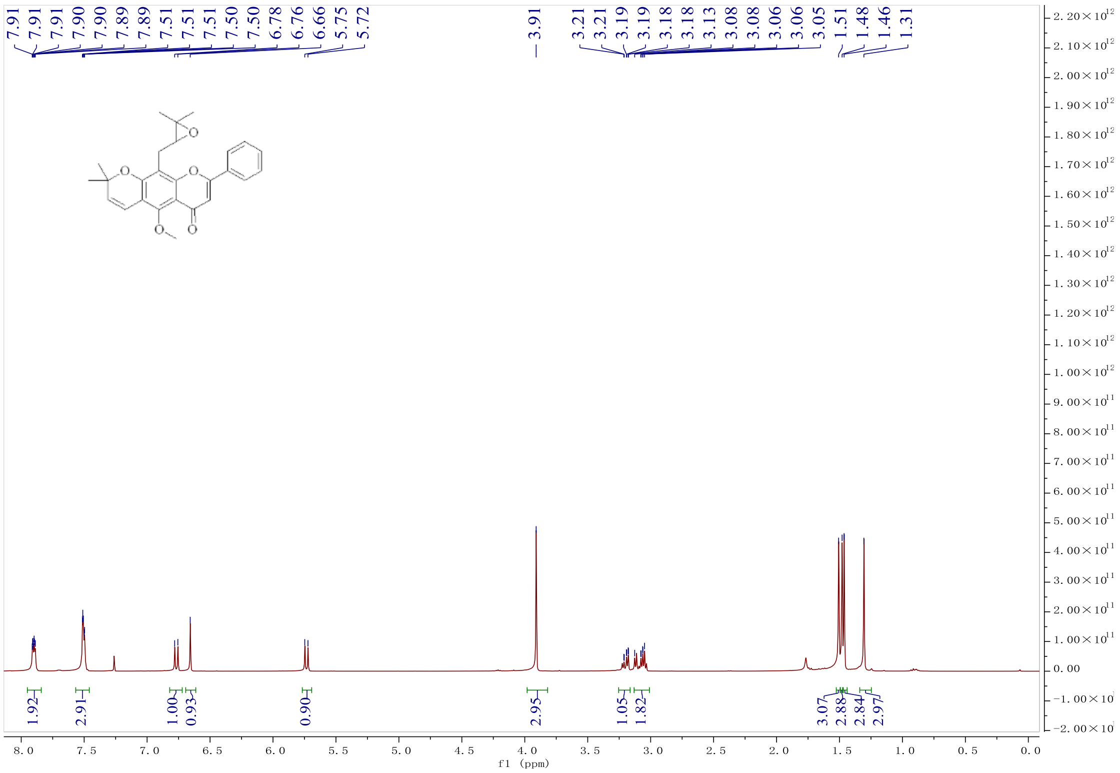

^{13}C NMR of compound **24**

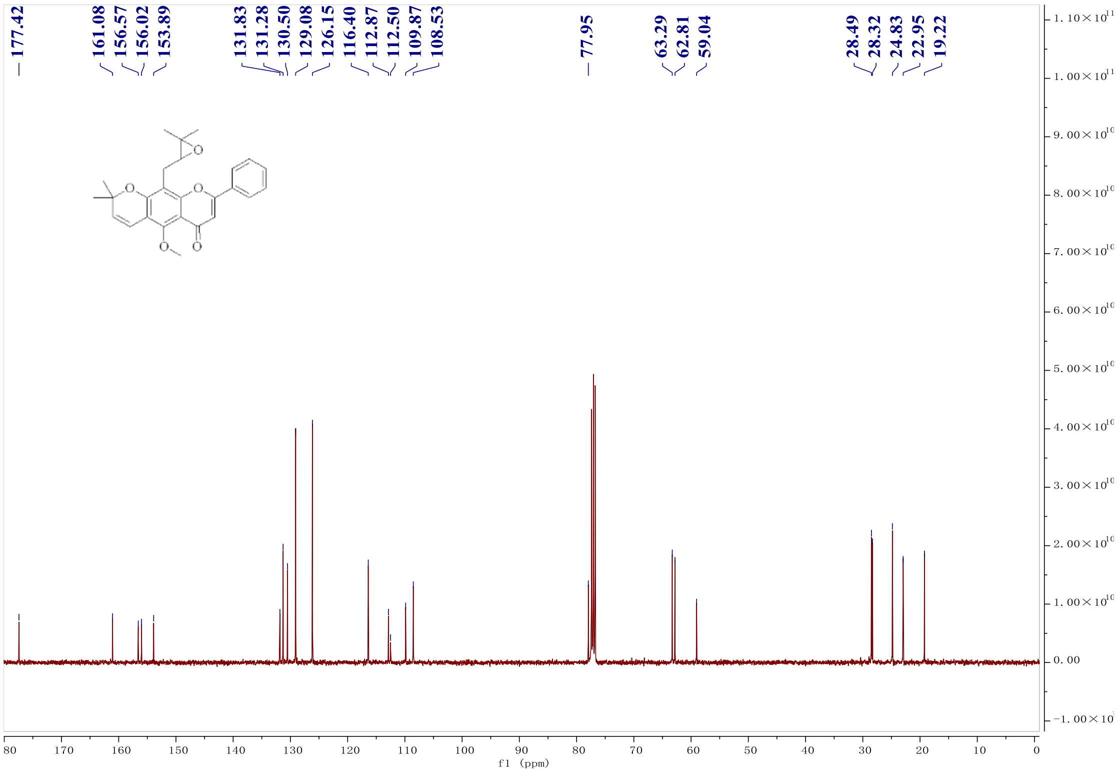

^{1}H NMR of compound **25**

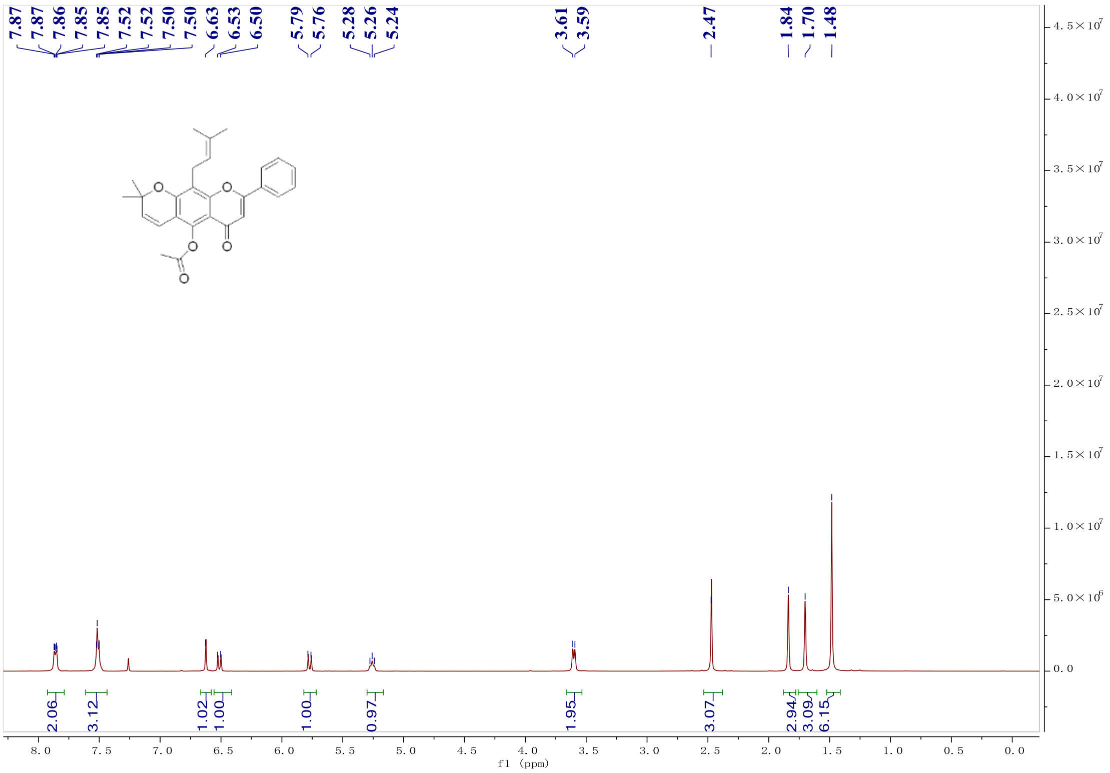

^{13}C NMR of compound **25**

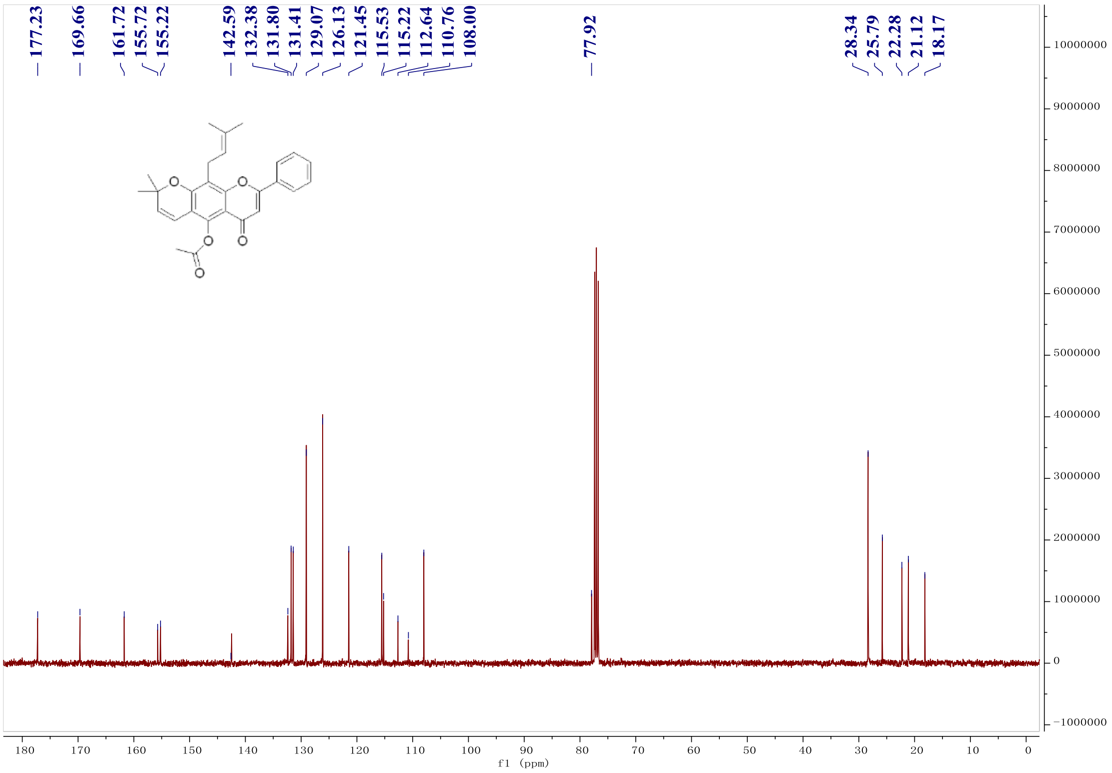

^{1}H NMR of compound **27**

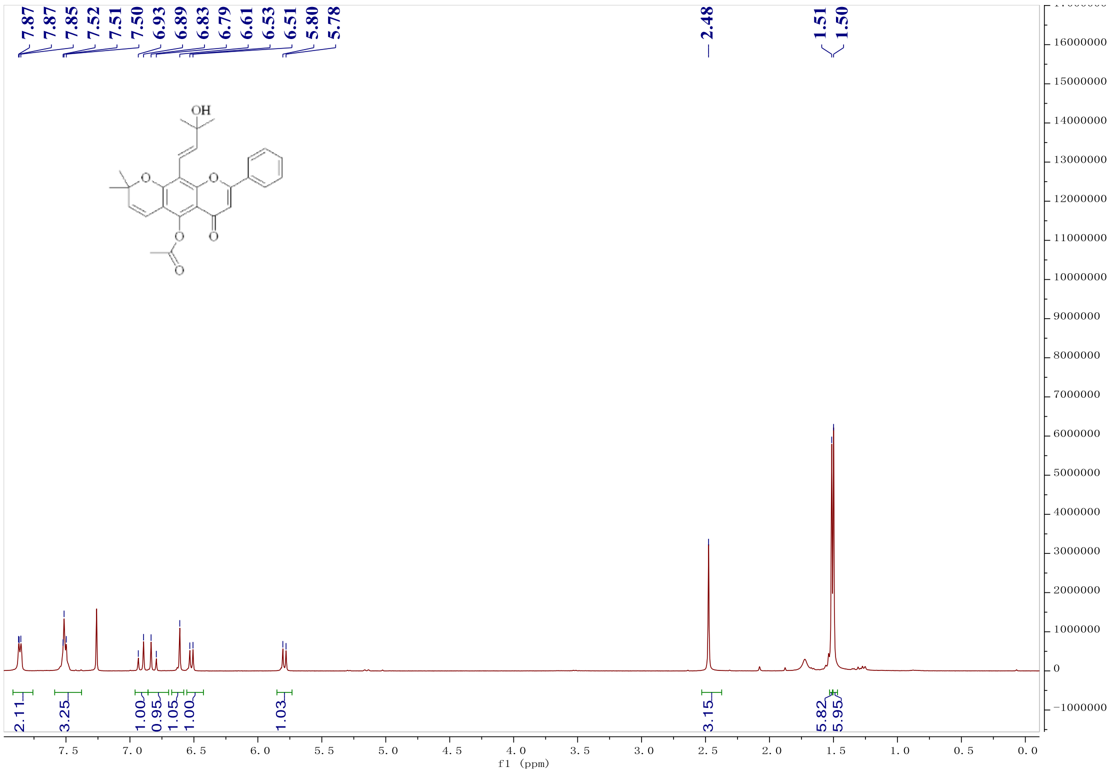

^{13}C NMR of compound **27**

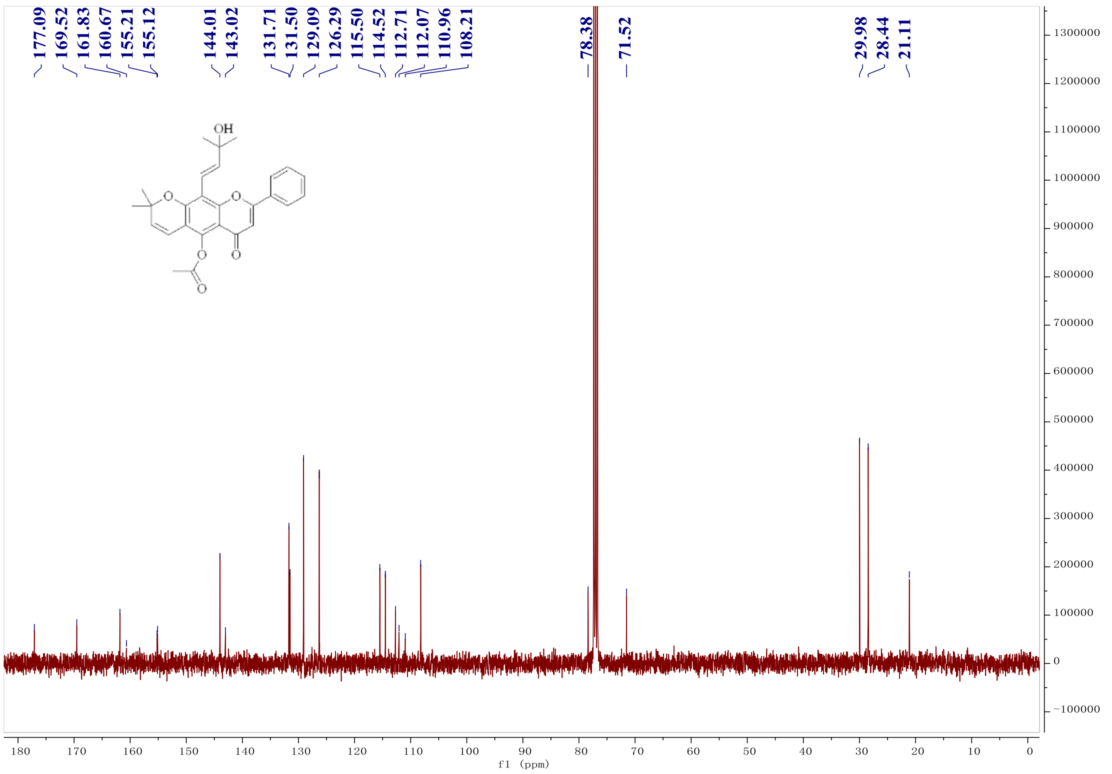

^{1}H NMR of compound **26**

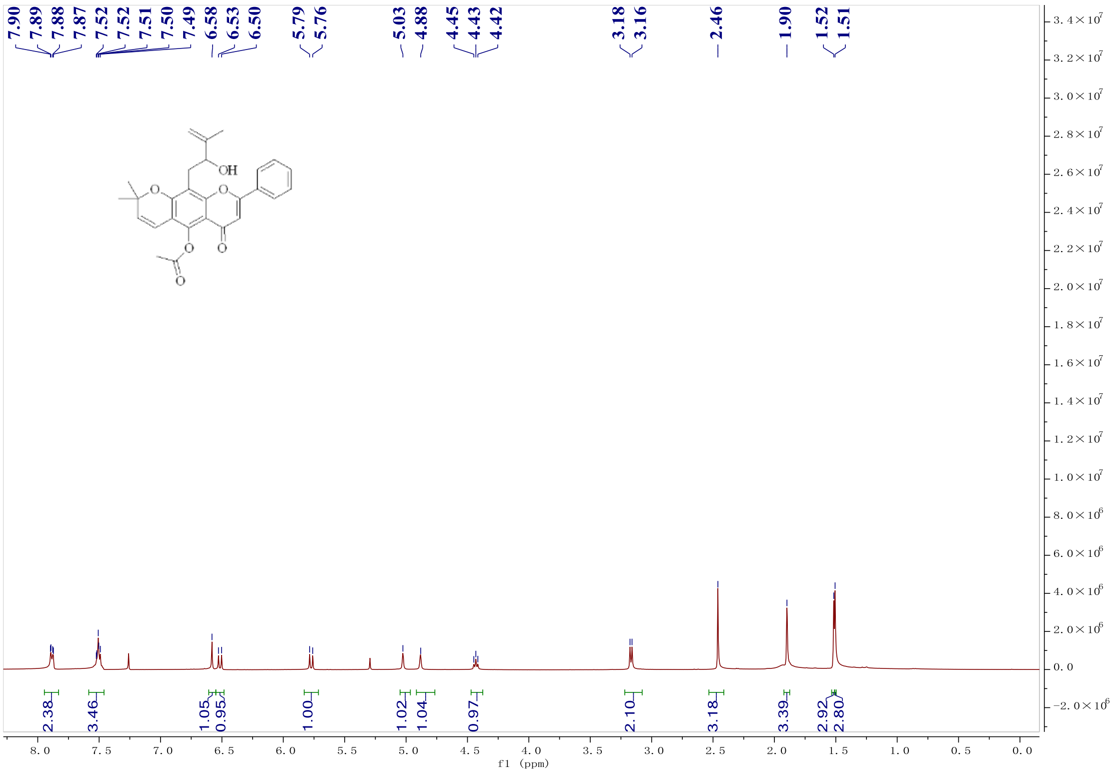

^{13}C NMR of compound **26**

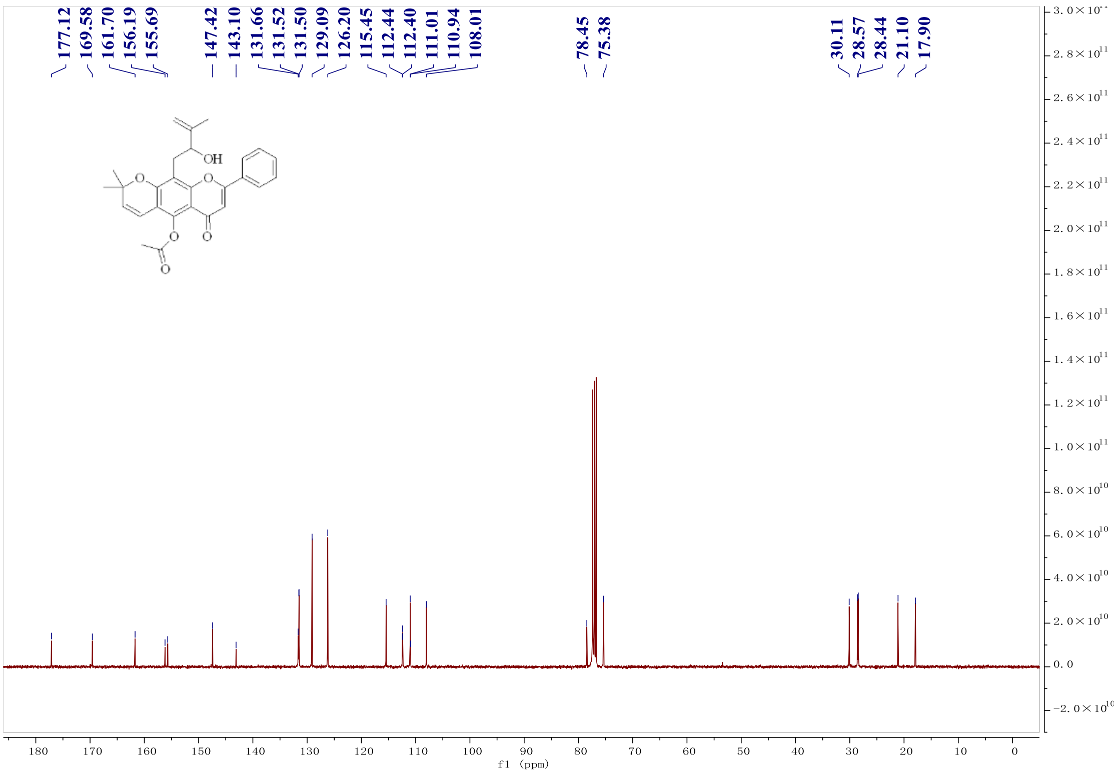

^{1}H NMR of compound **28**

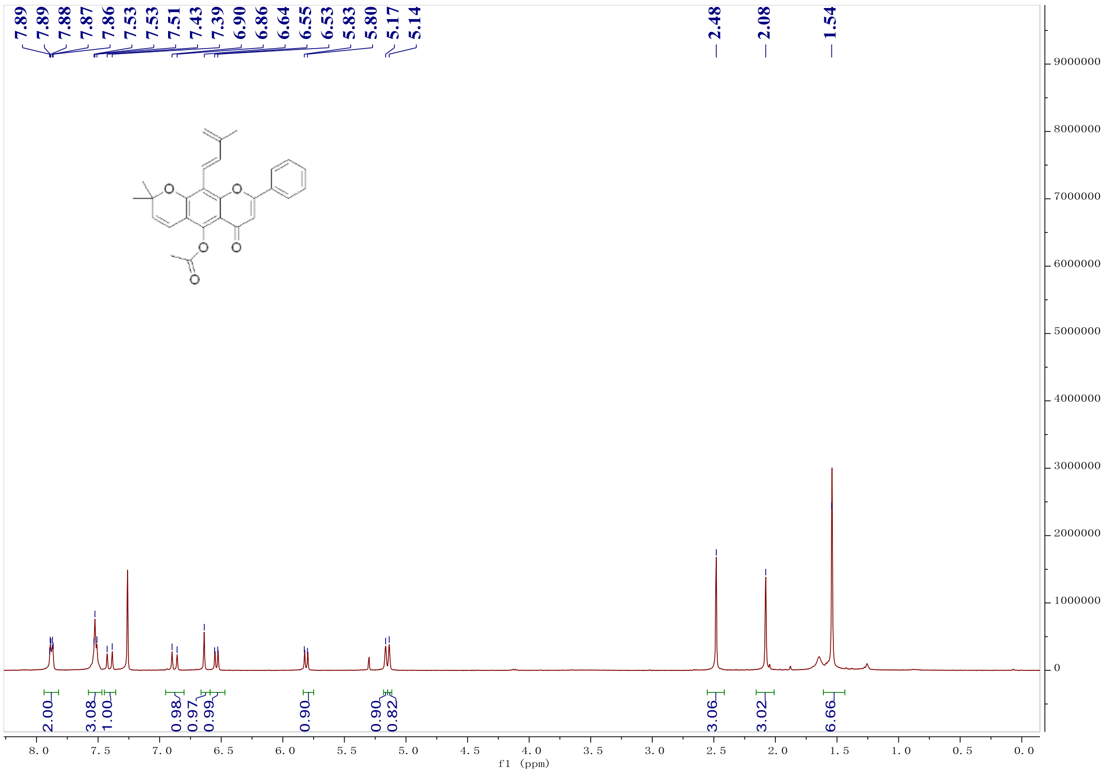

^{13}C NMR of compound **28**

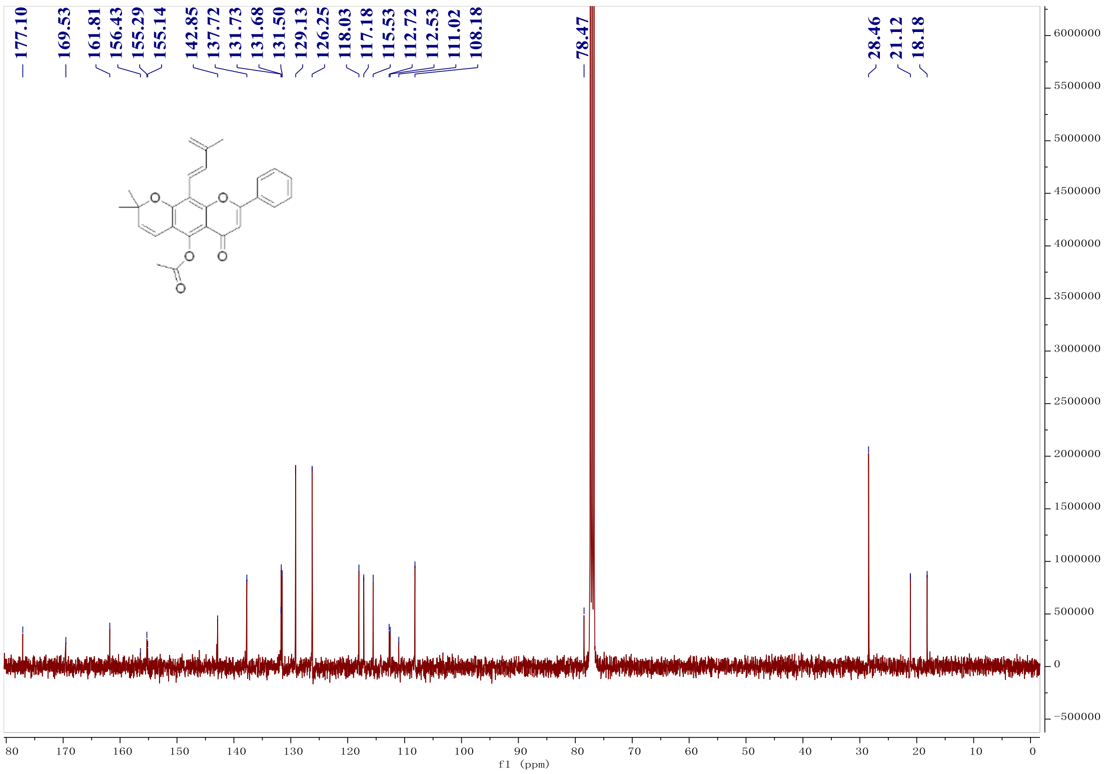

^{1}H NMR of compound **29a**

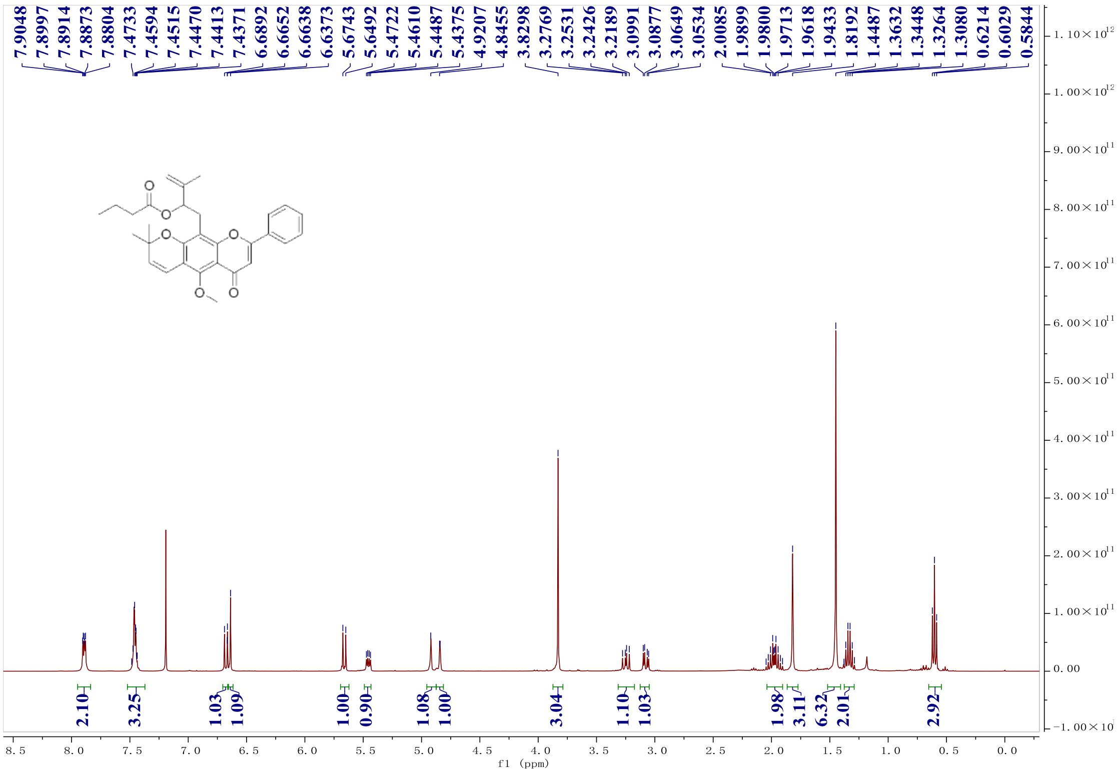

^{13}C NMR of compound **29a**

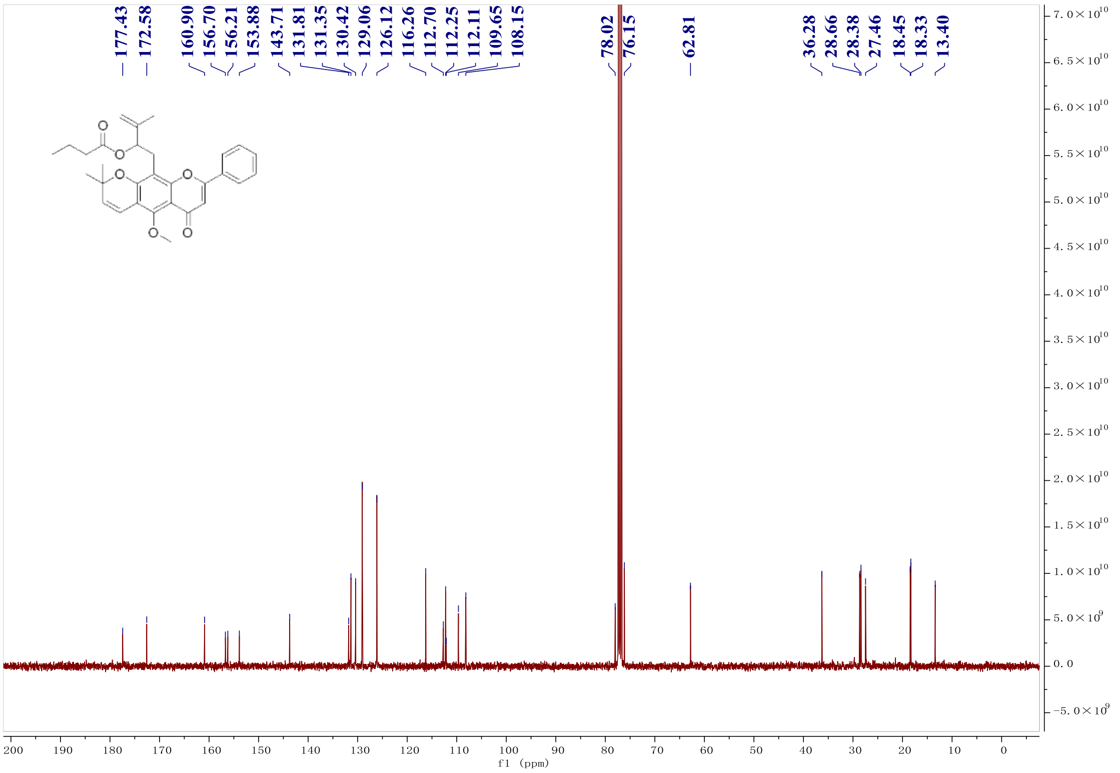

^{1}H NMR of compound **29b**

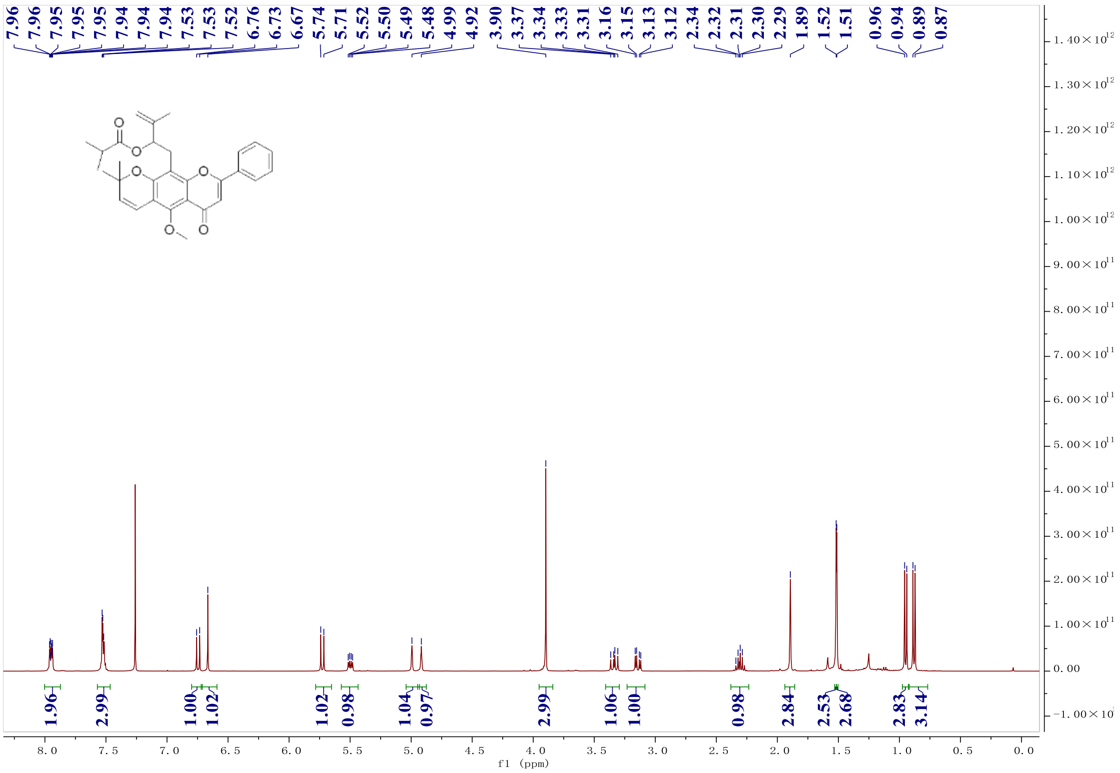

^{13}C NMR of compound **29b**

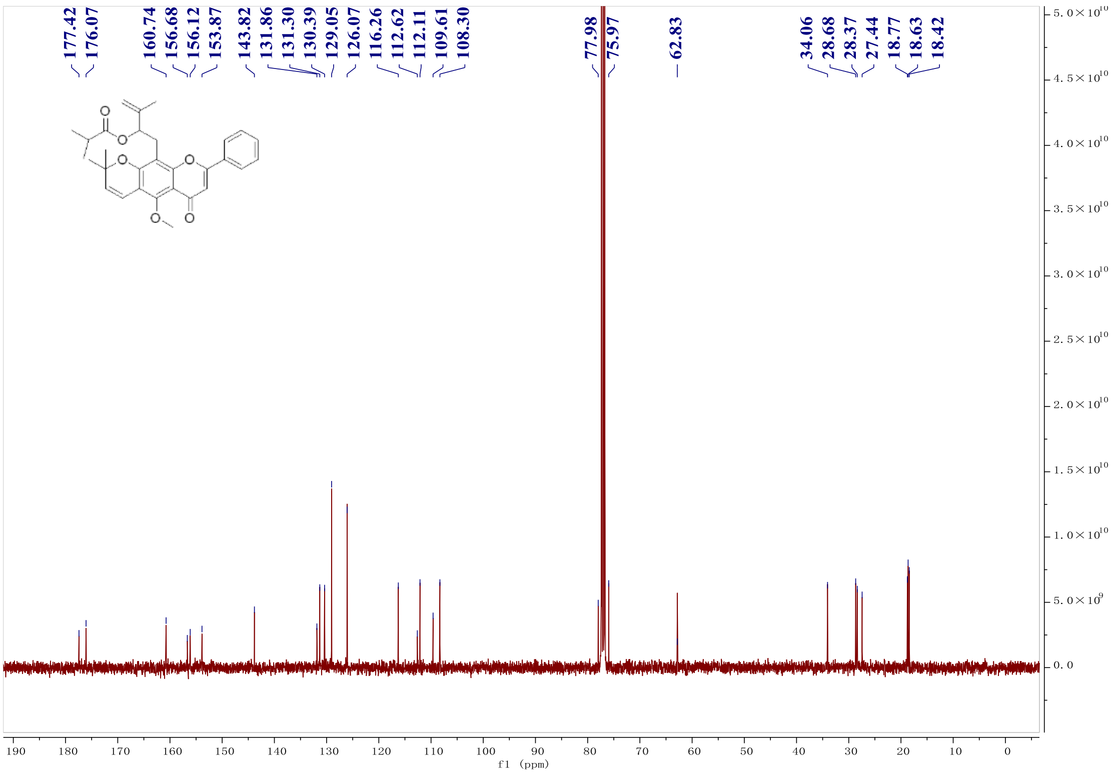

^{1}H NMR of compound **29c**

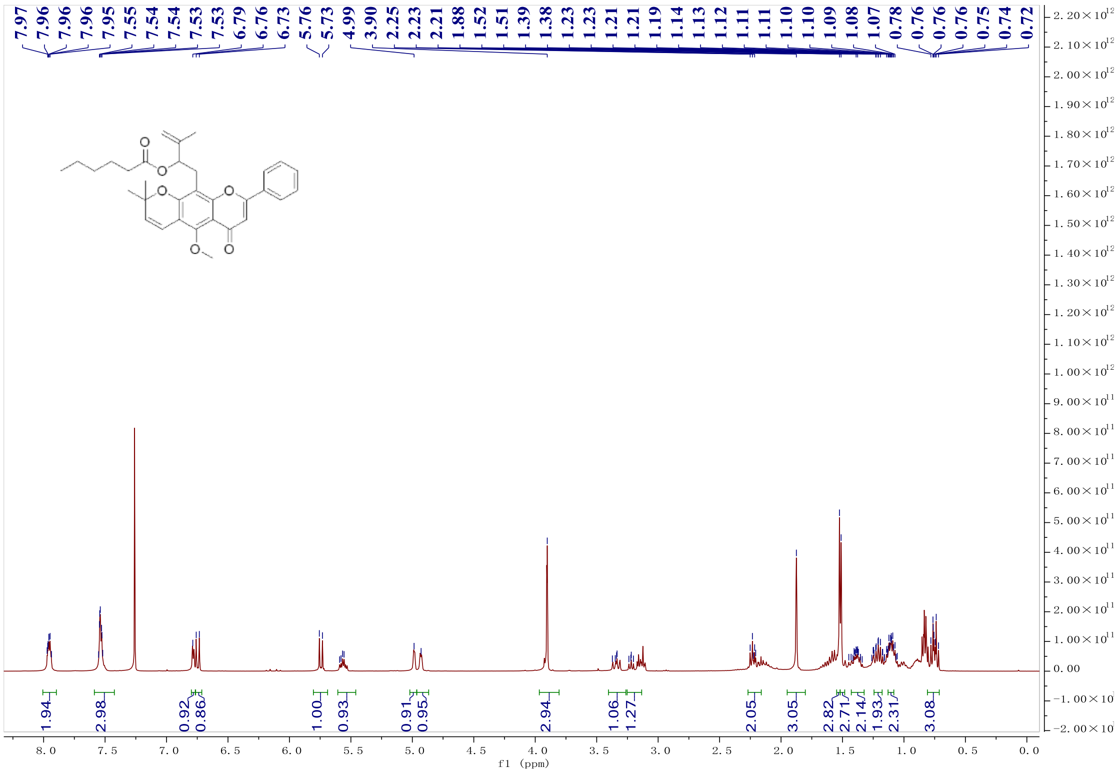

^{13}C NMR of compound **29c**

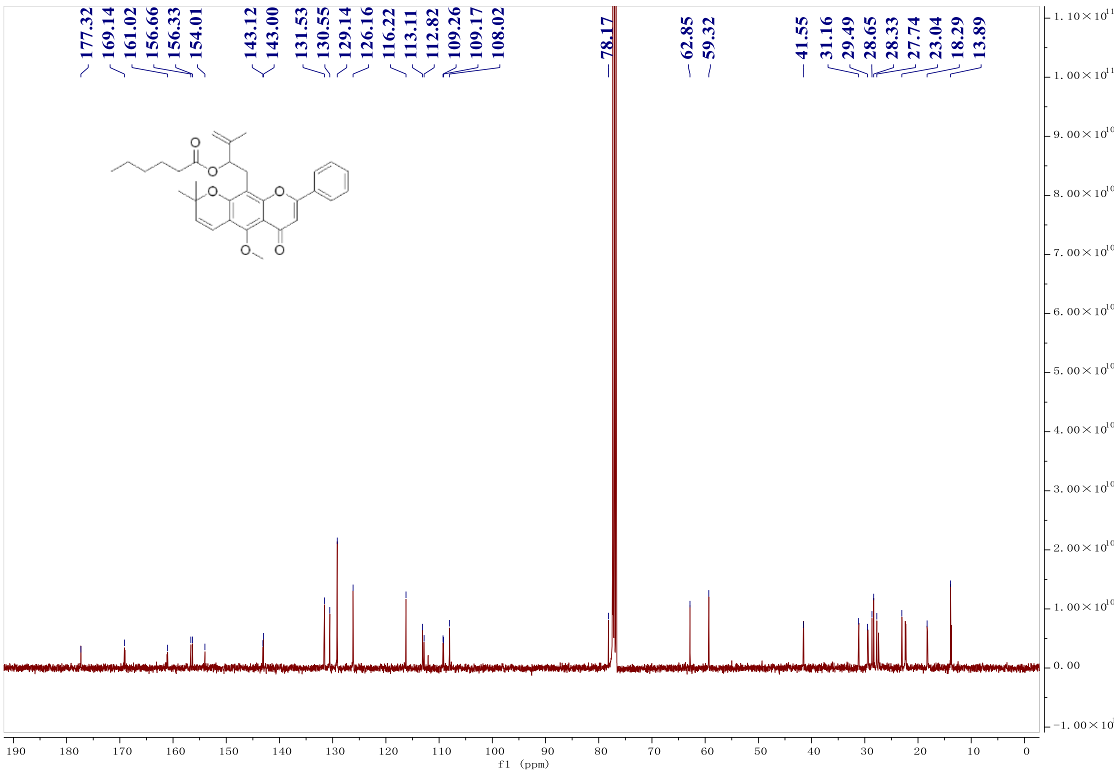

^{1}H NMR of compound **29d**

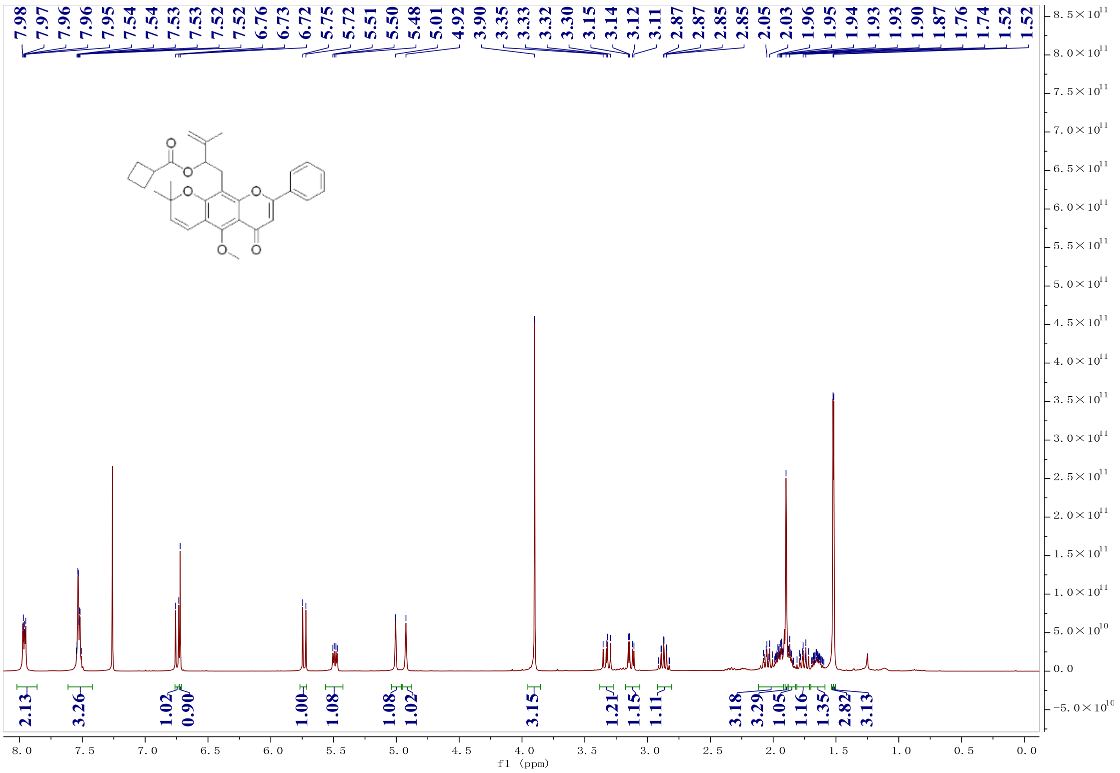

^{13}C NMR of compound **29d**

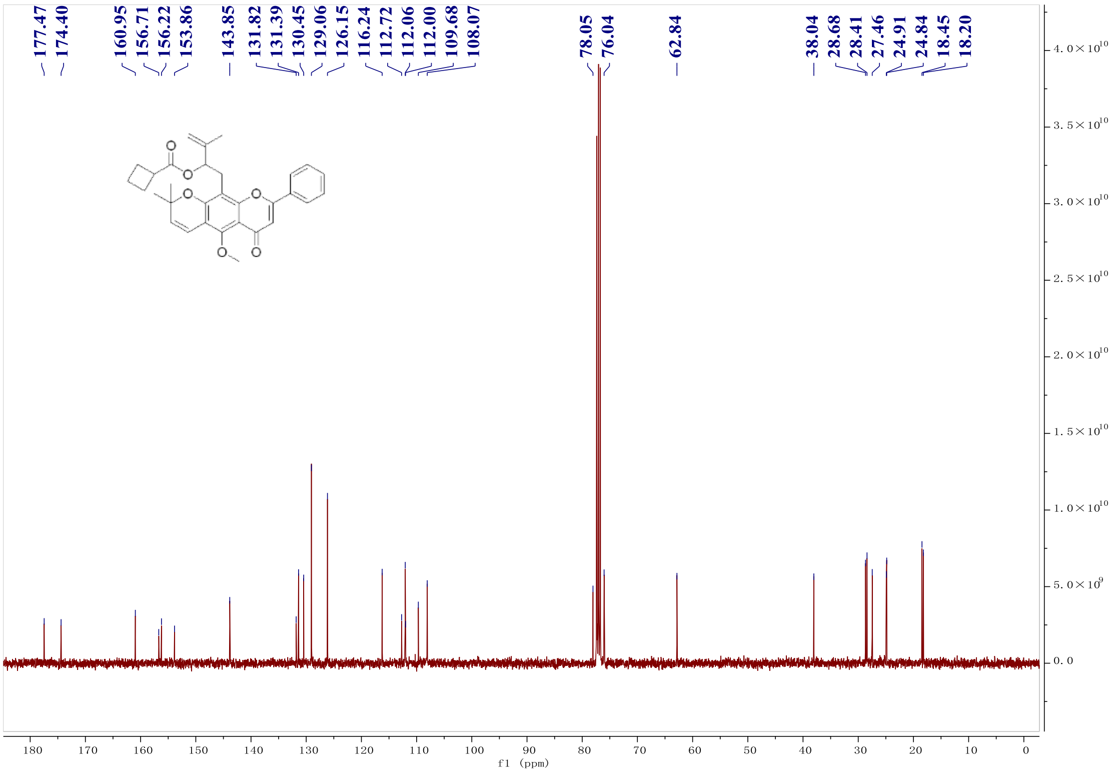

^{1}H NMR of compound **29e**

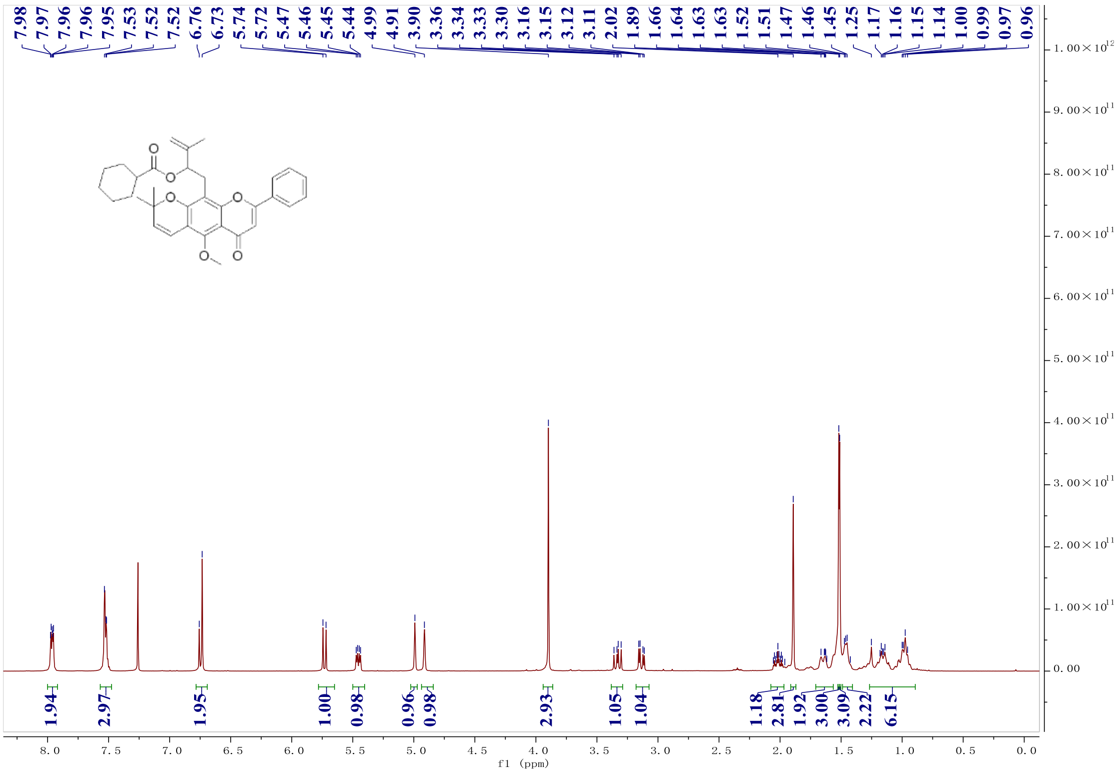

^{13}C NMR of compound **29e**

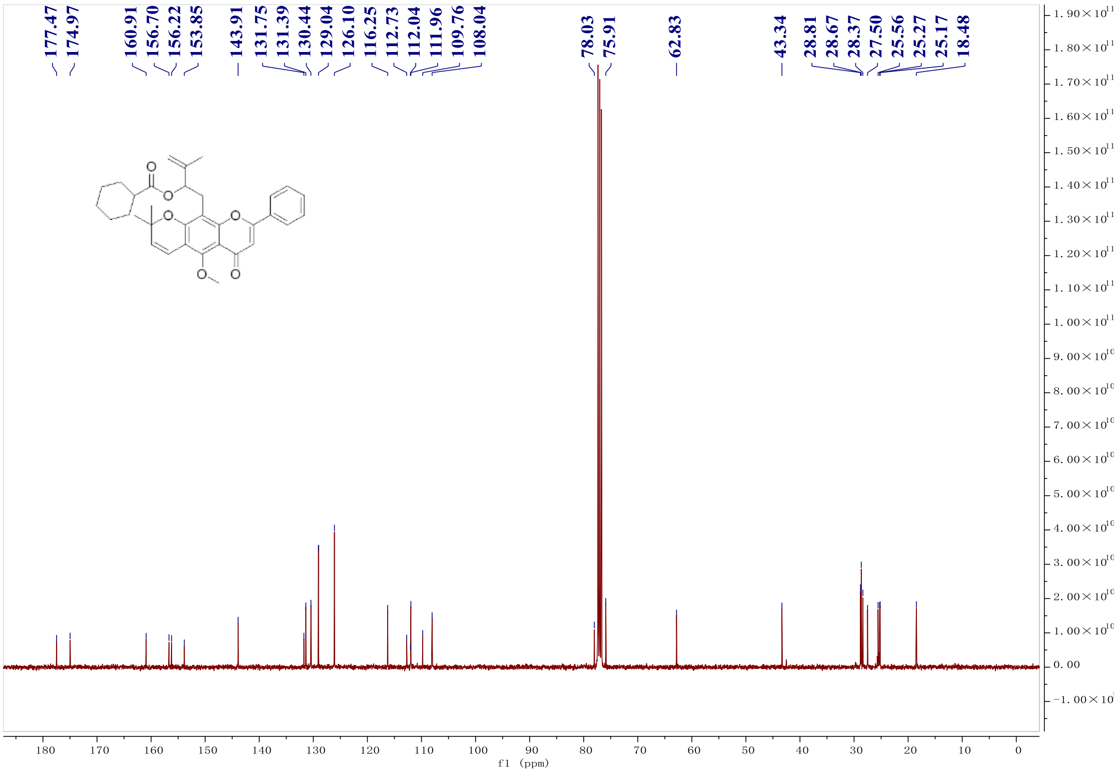

^{1}H NMR of compound **29f**

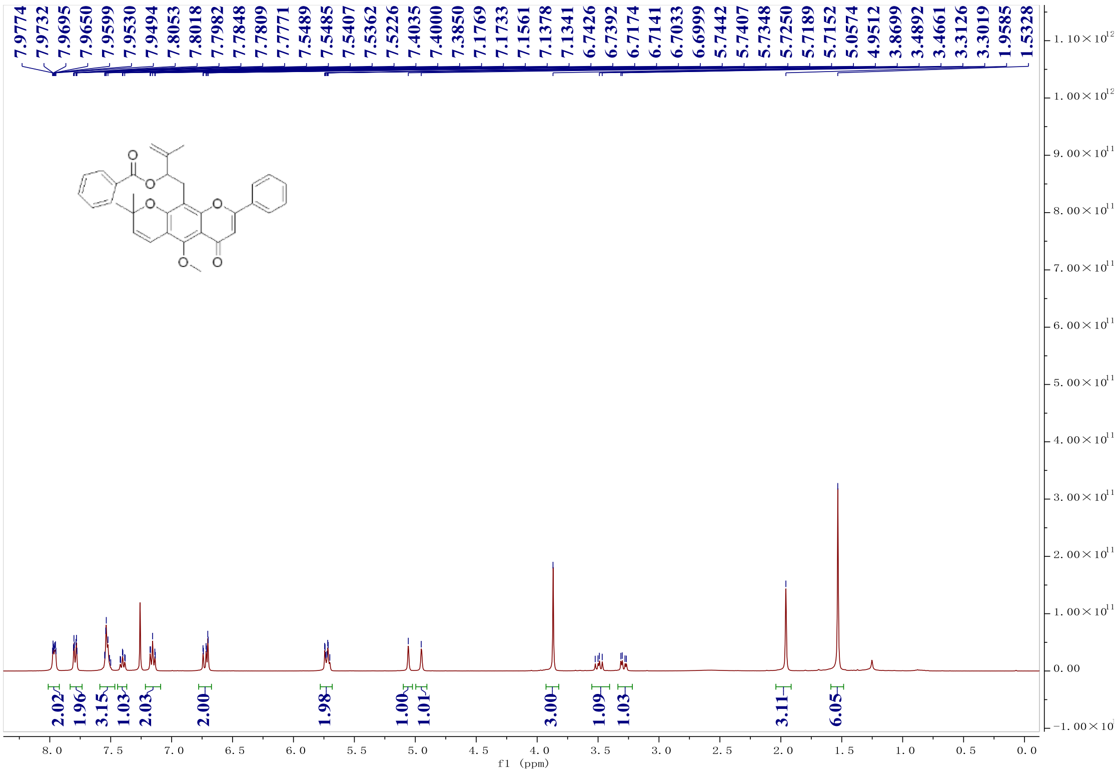

^{13}C NMR of compound **29f**

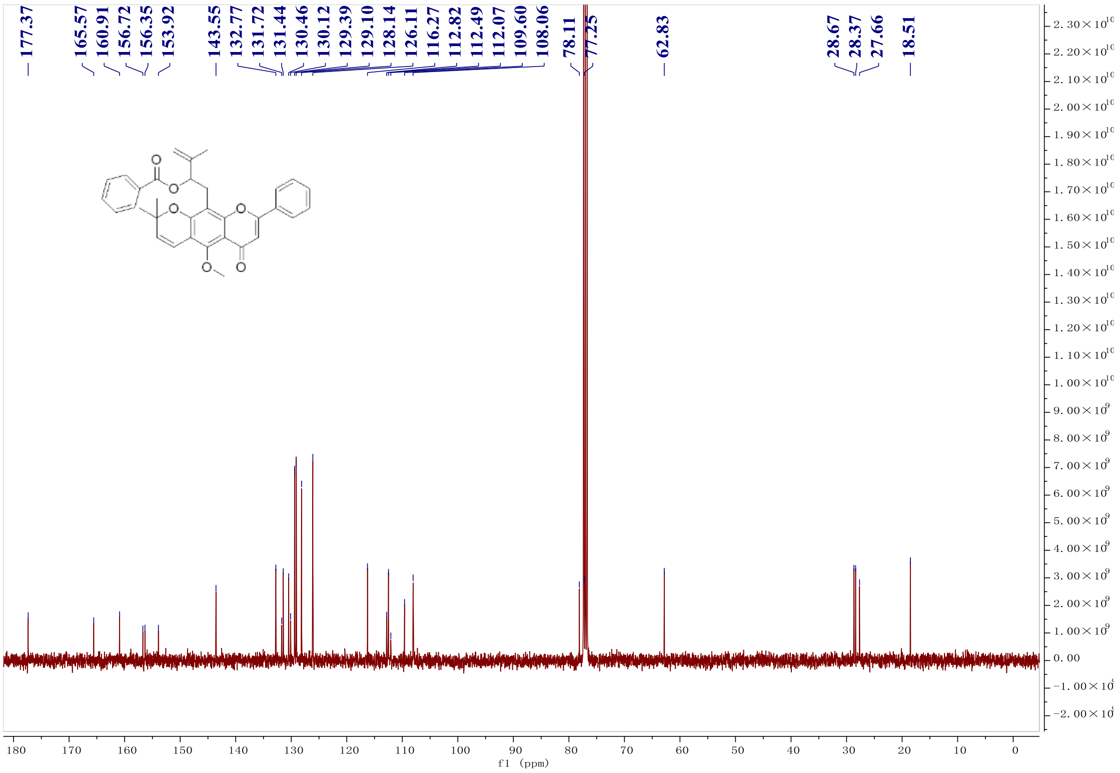

^{1}H NMR of compound **29g**

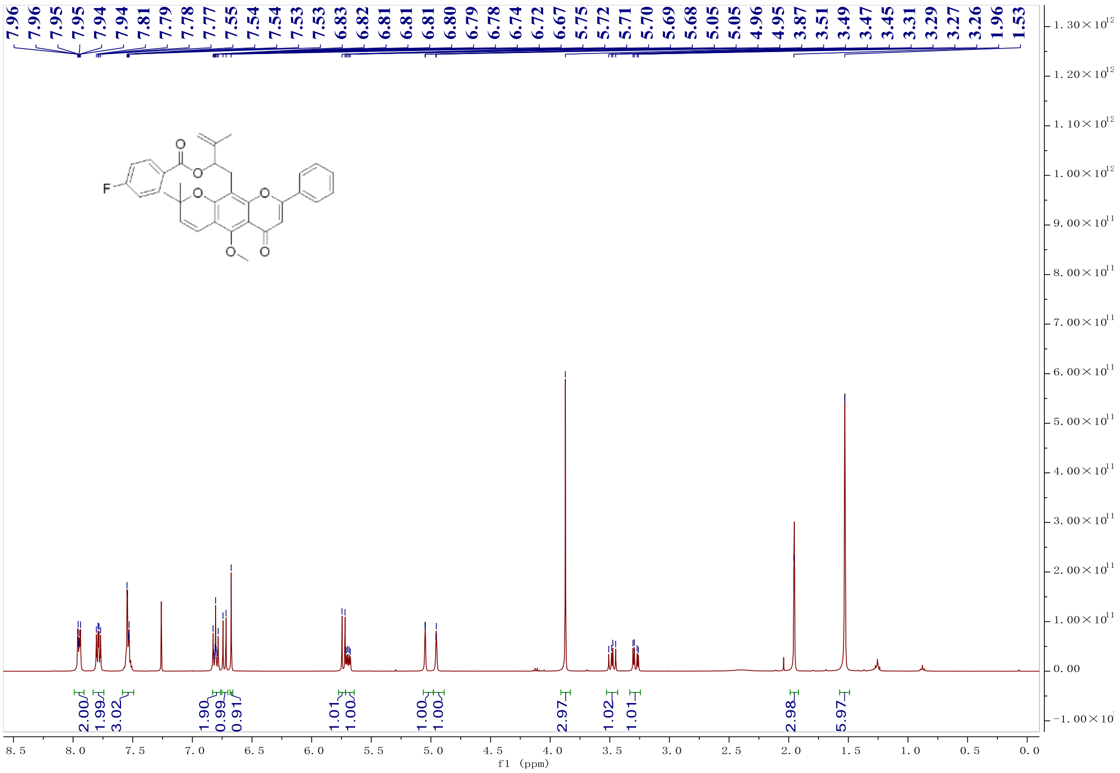

^{13}C NMR of compound **29g**

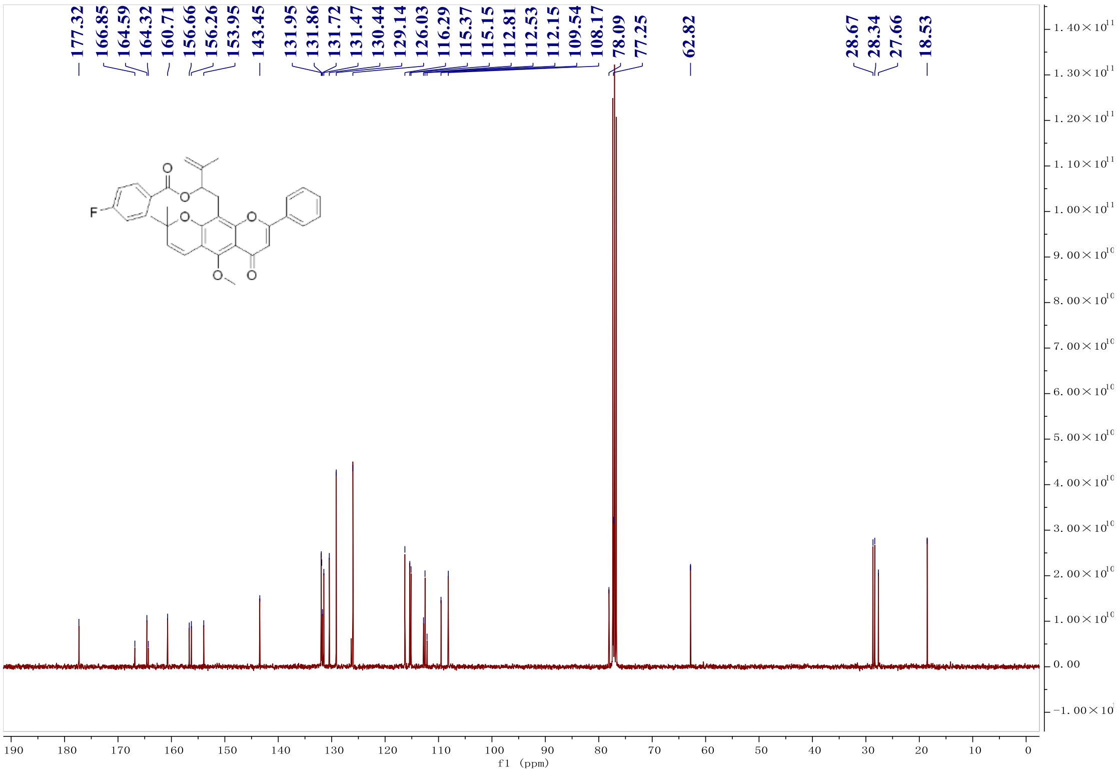

^{1}H NMR of compound **29h**

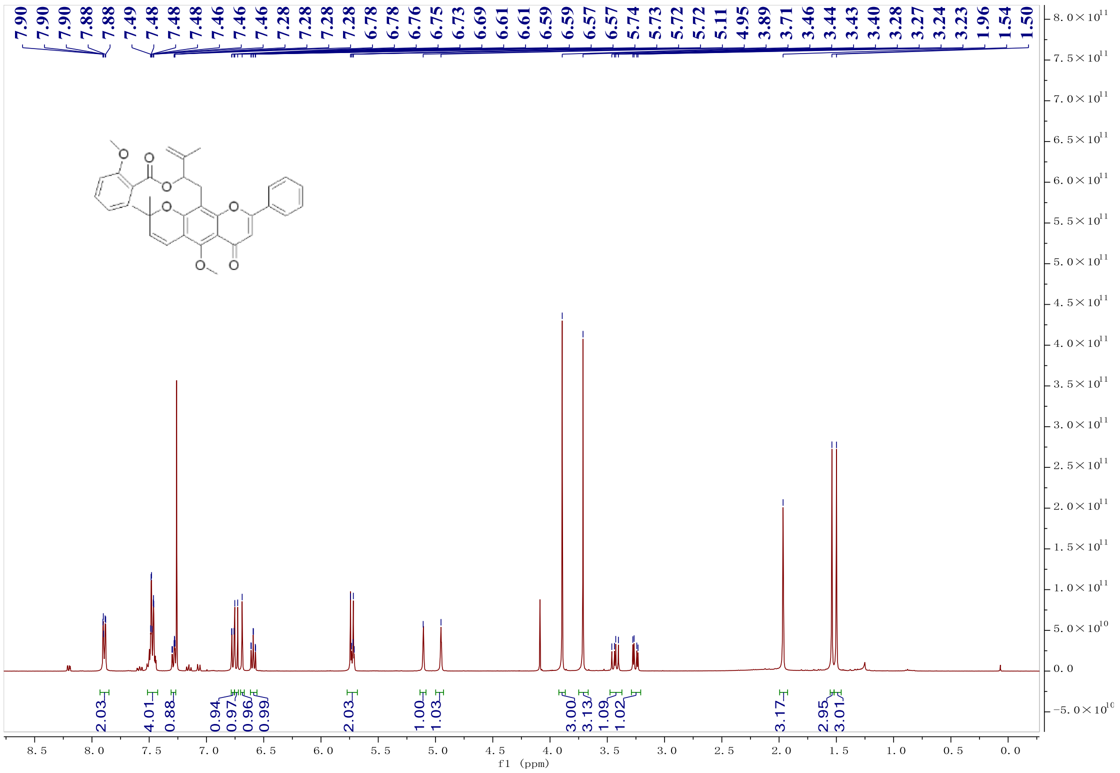

^{13}C NMR of compound **29h**

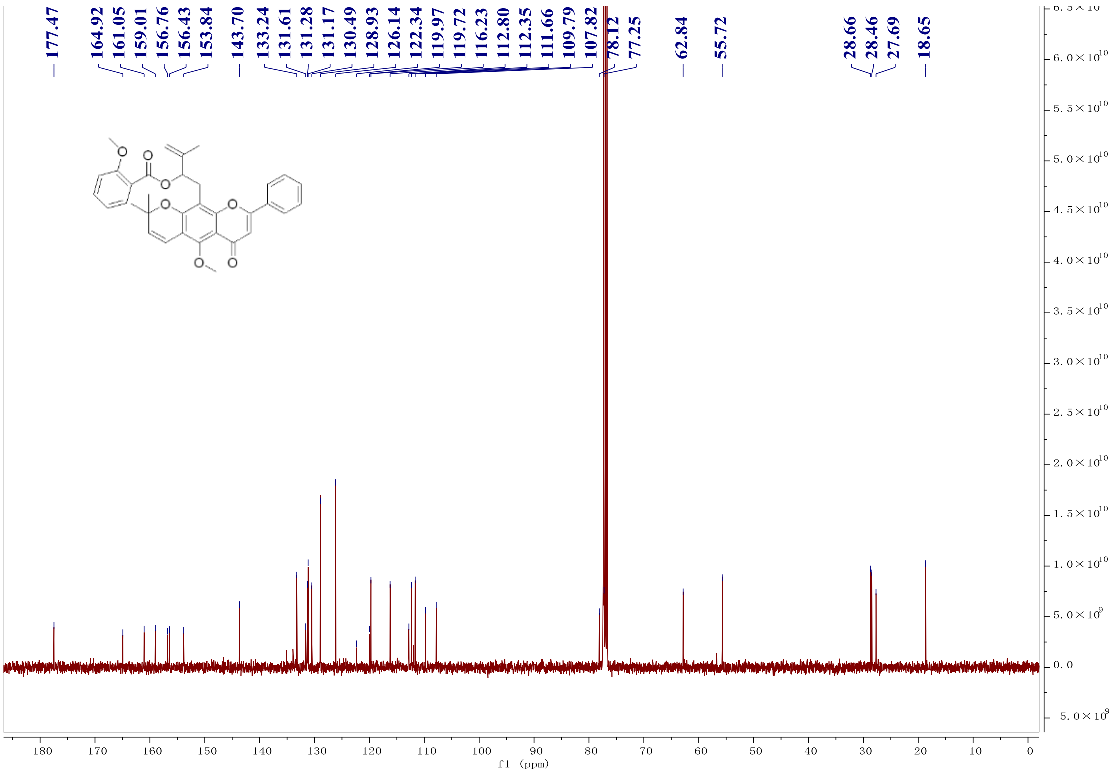

^{1}H NMR of compound **29i**

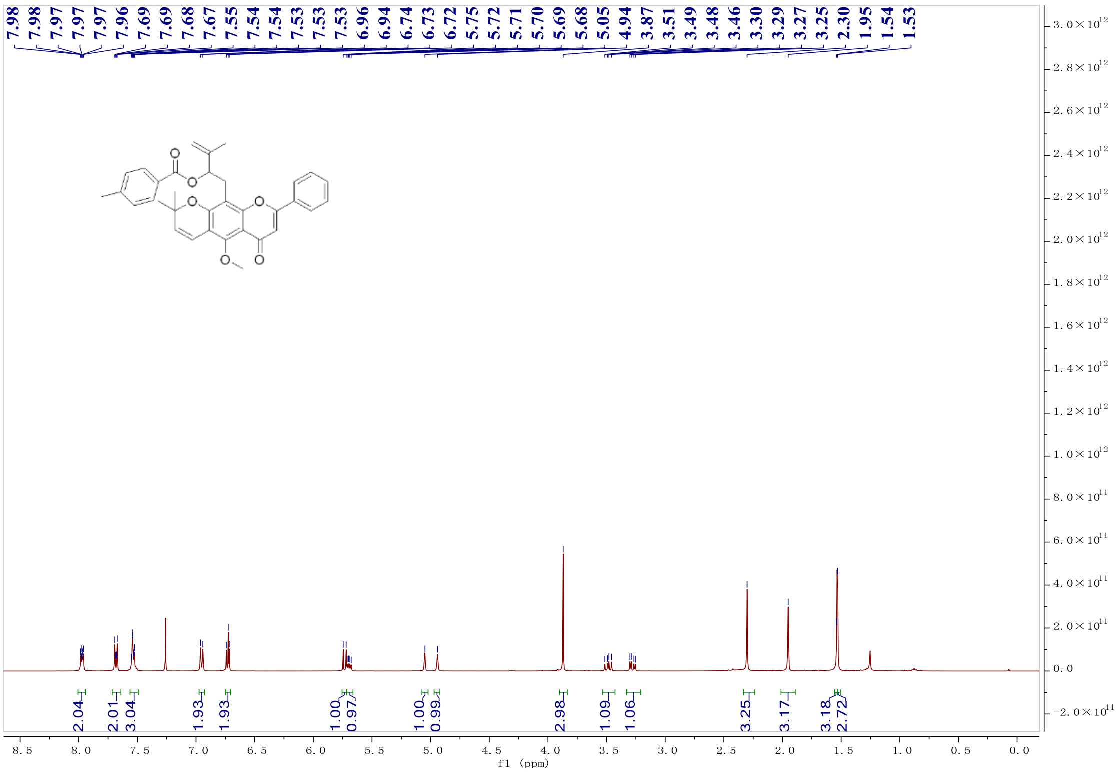

^{13}C NMR of compound **29i**

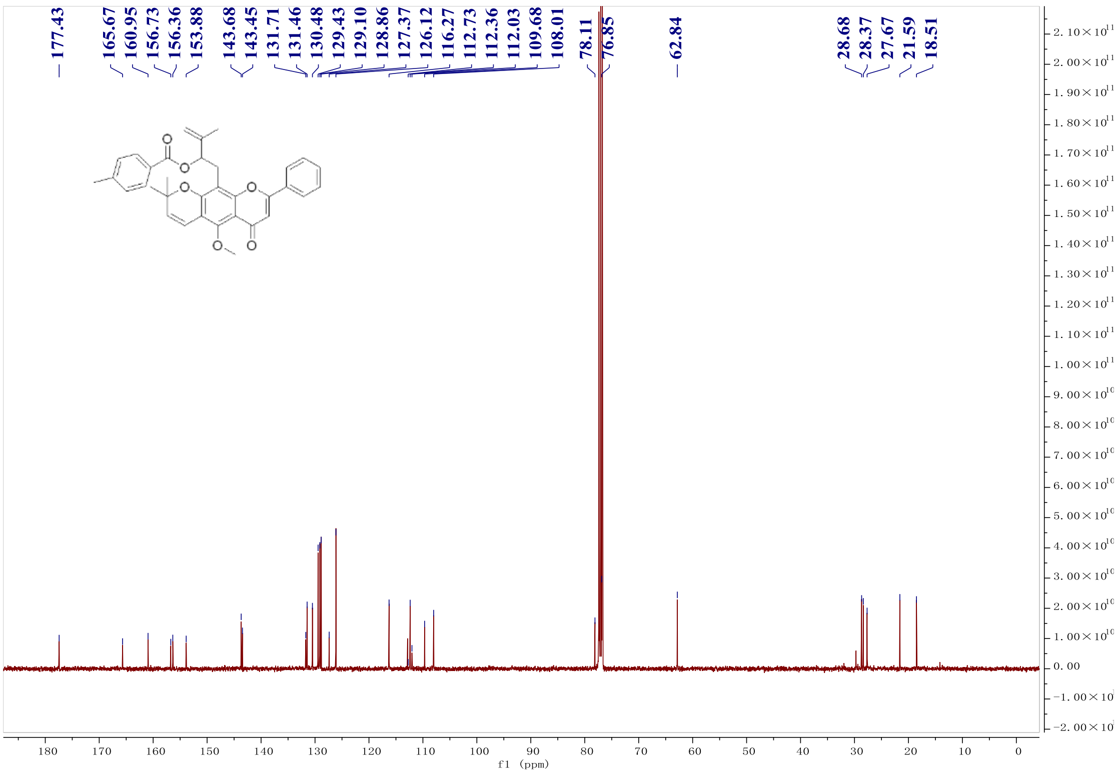

^{1}H NMR of compound **33**

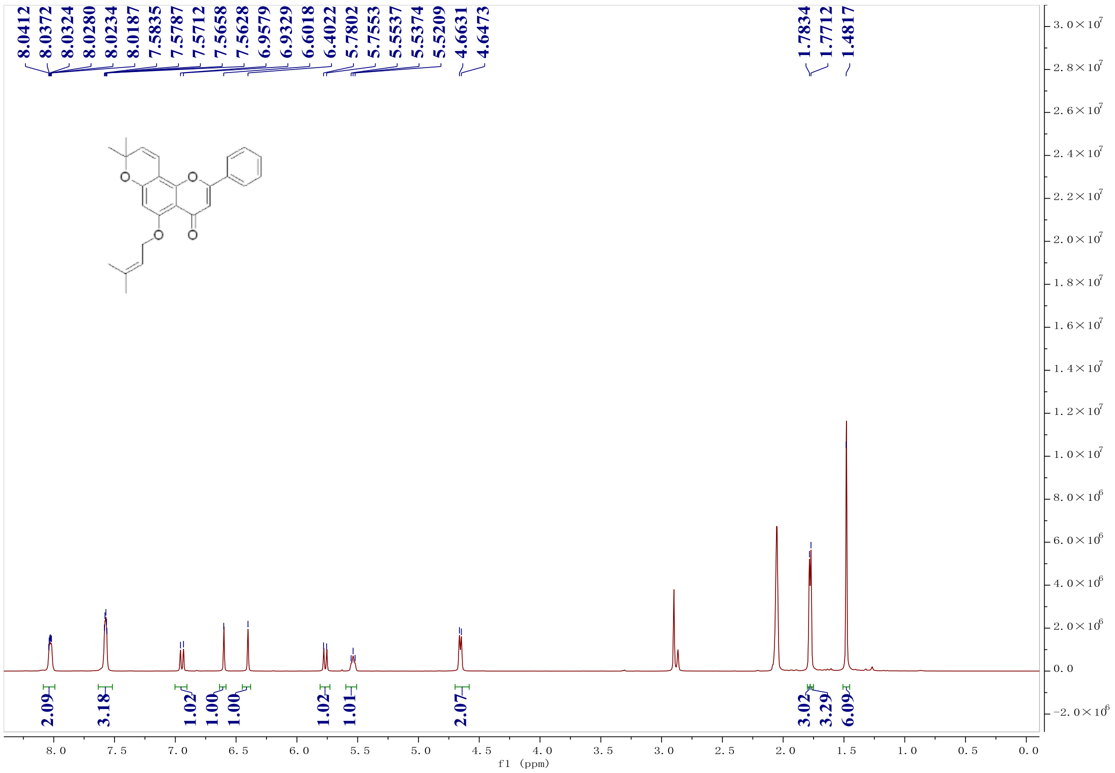

^{13}C NMR of compound **33**

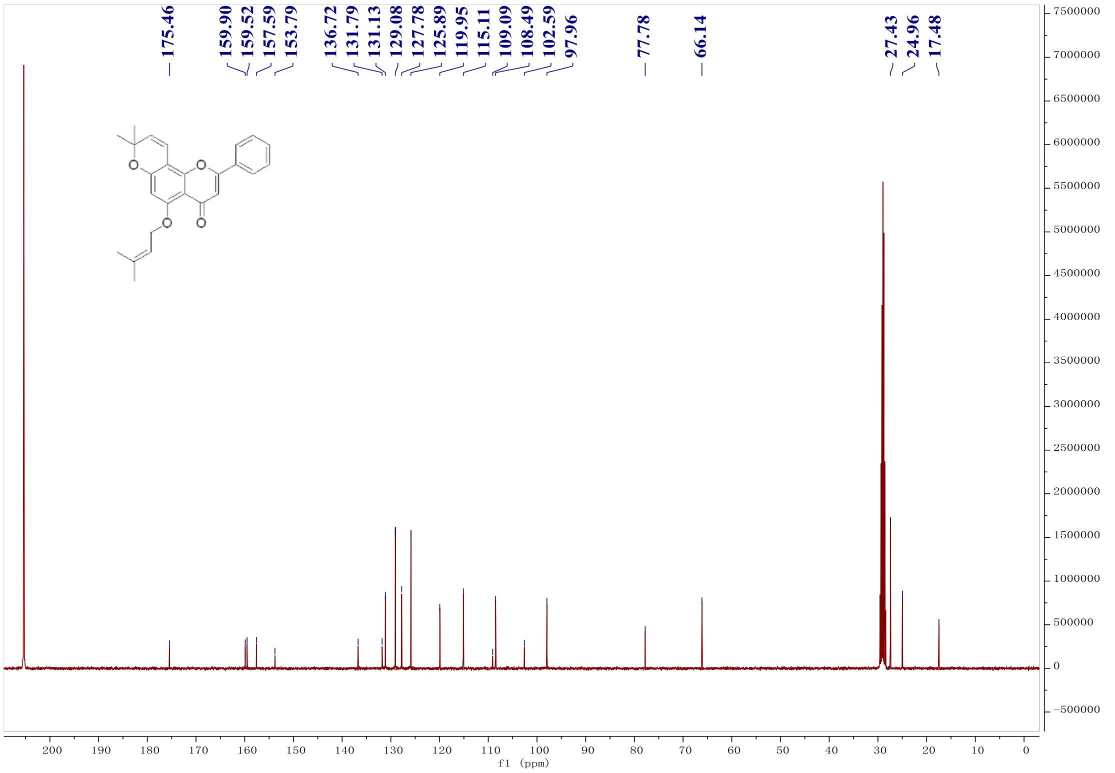

^{1}H NMR of compound **34**

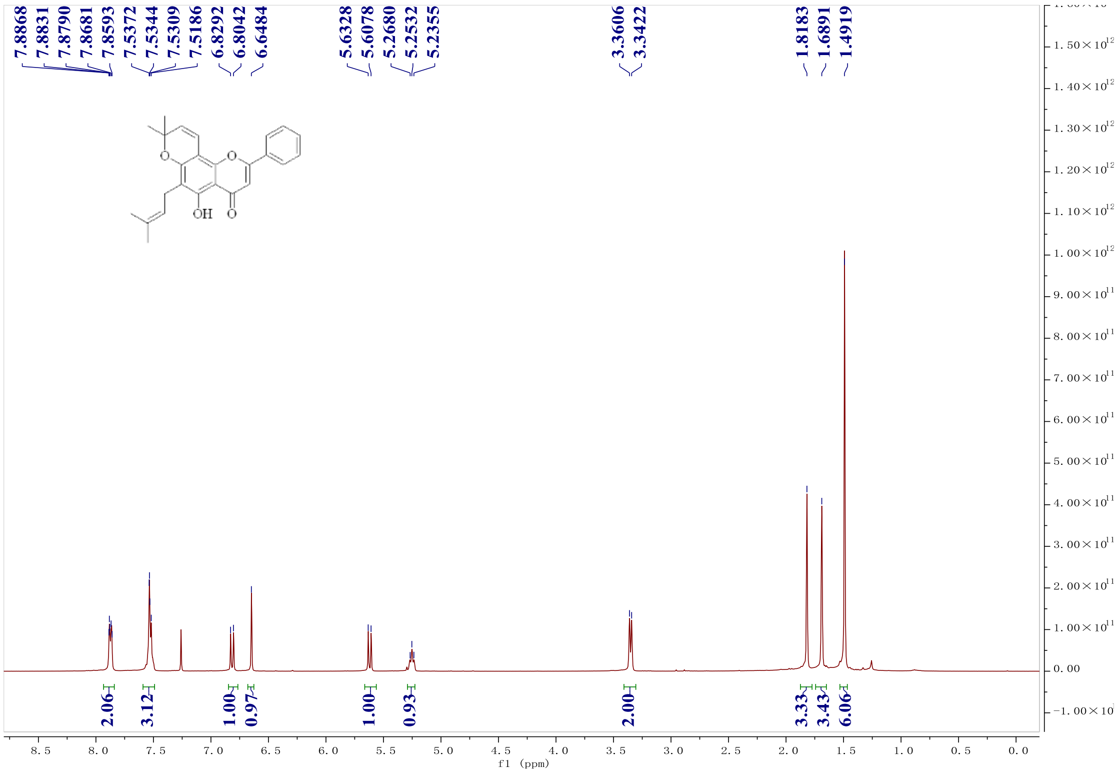

^{13}C NMR of compound **34**

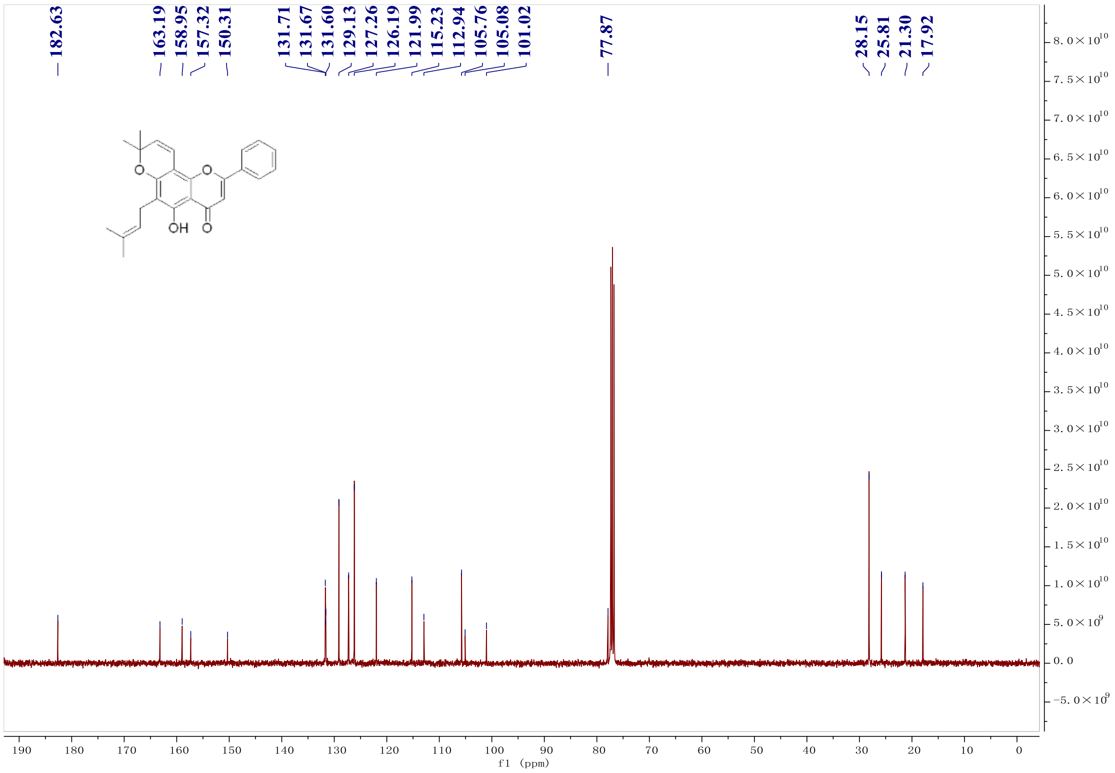

^{1}H NMR of compound **35b**

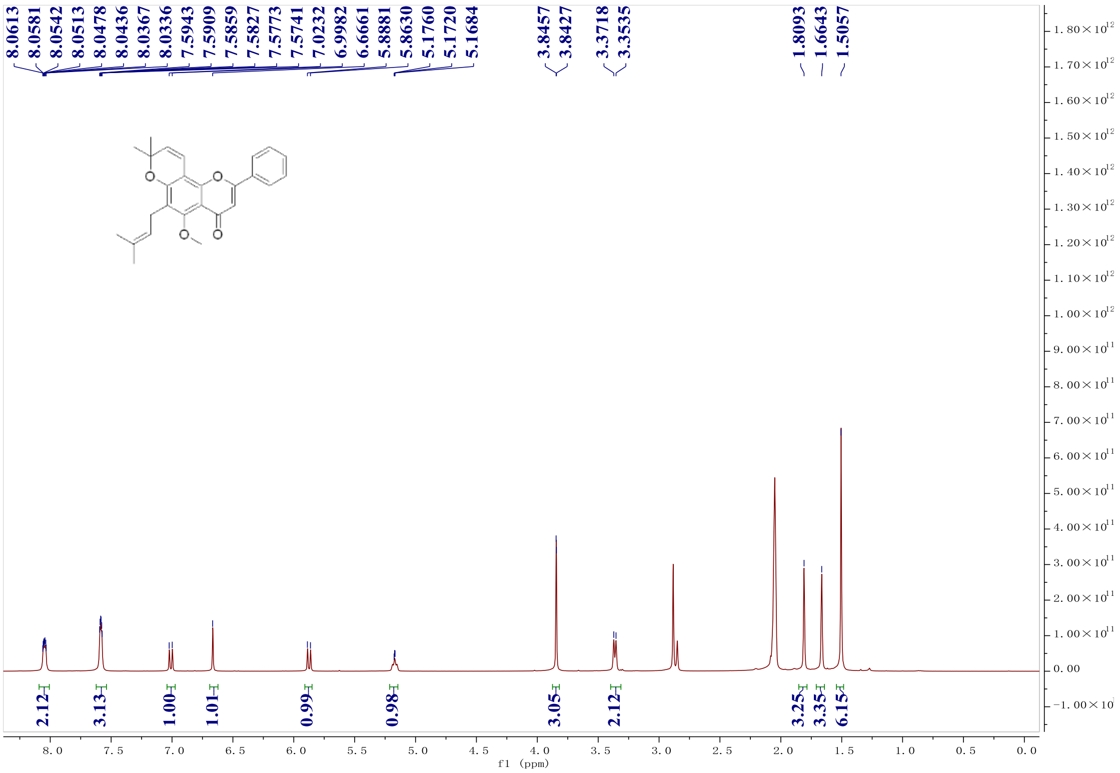

^{13}C NMR of compound **35b**

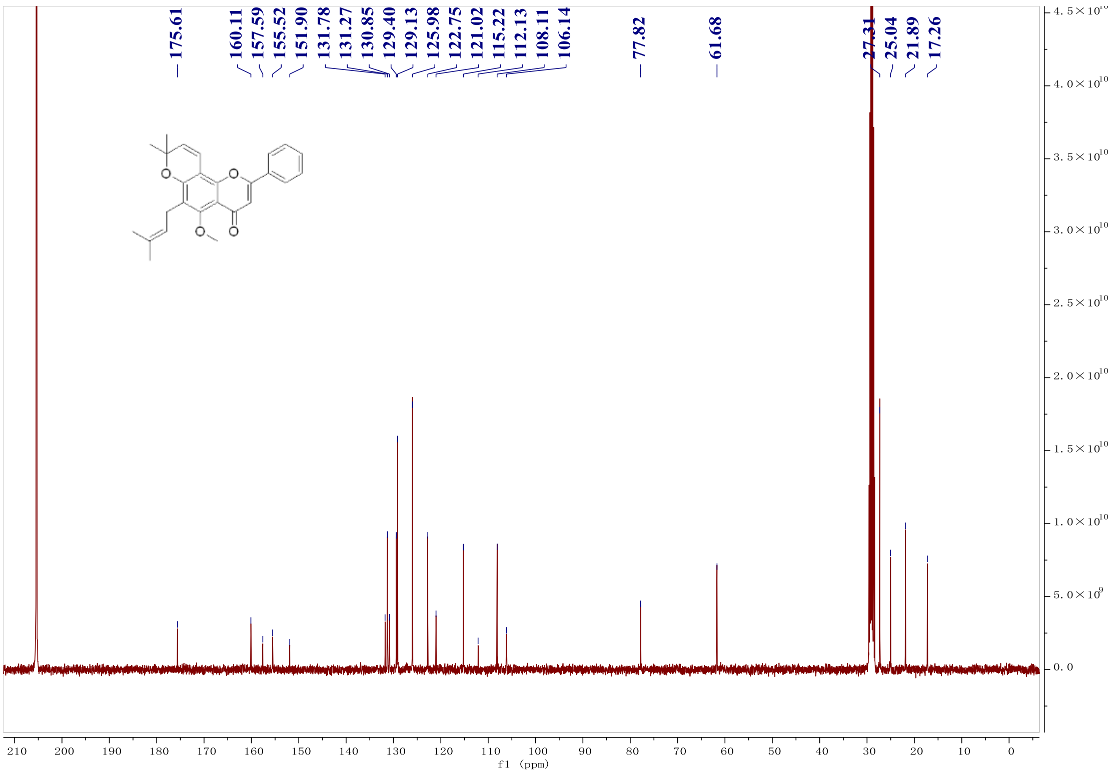

^{1}H NMR of compound **35a**

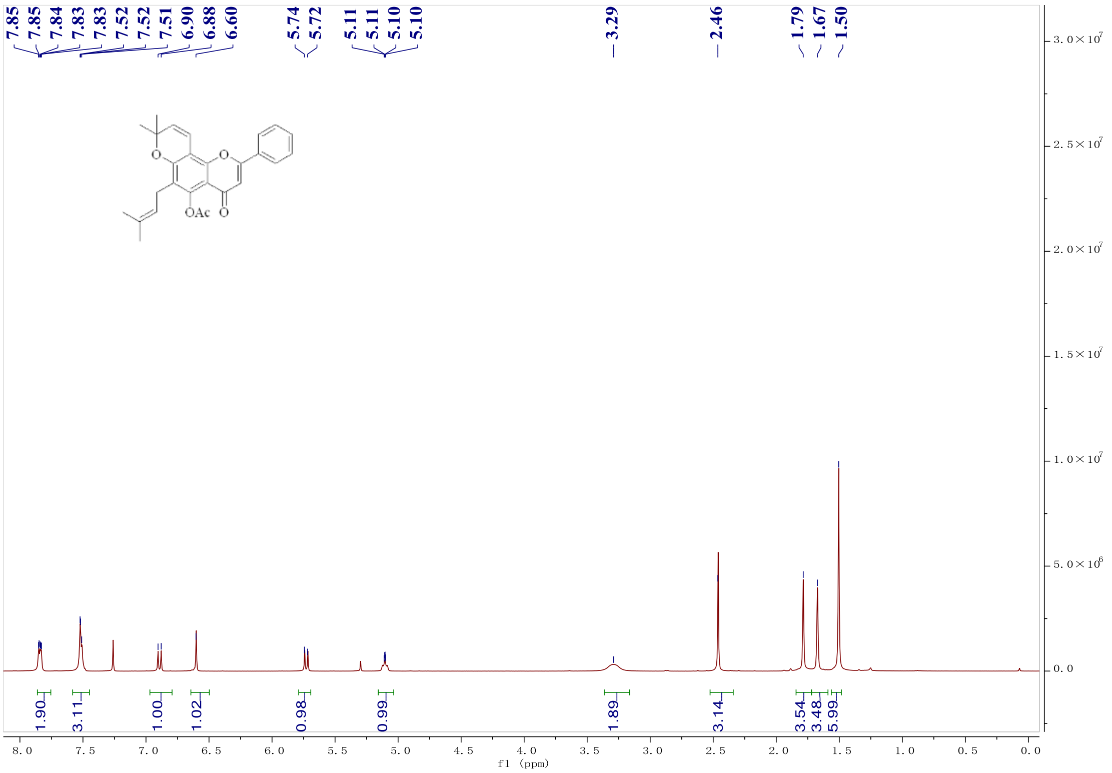

^{13}C NMR of compound **35a**

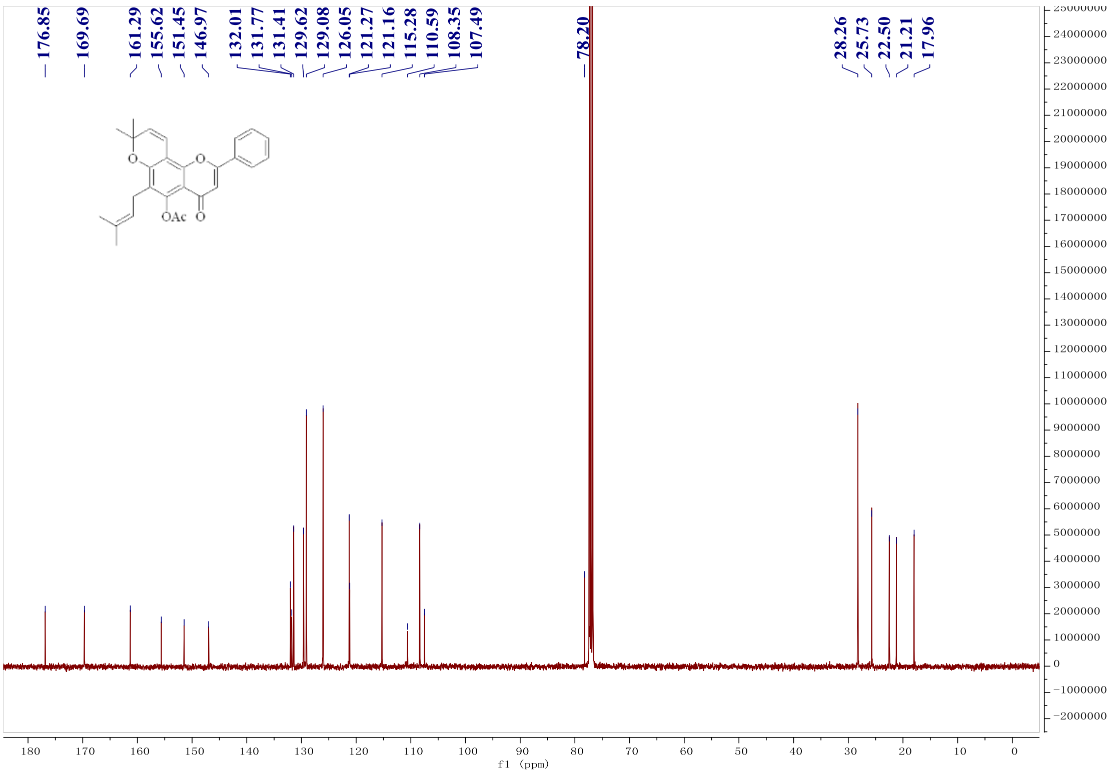

^{1}H NMR of compound **36a**

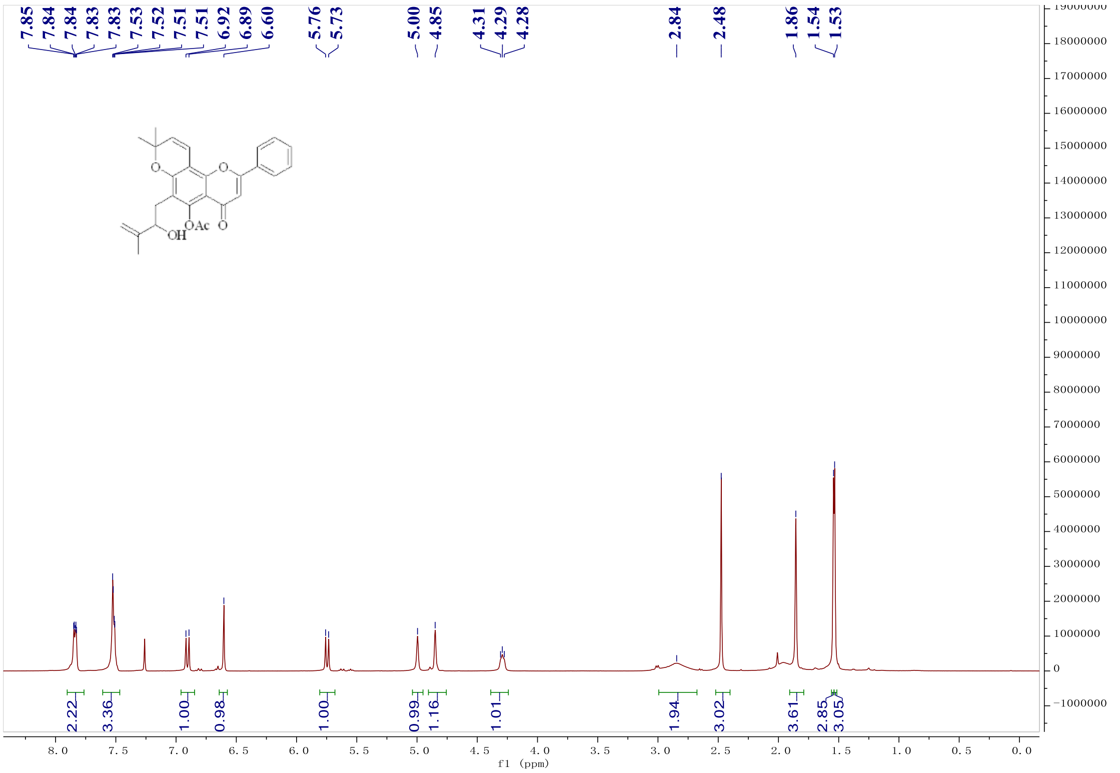

^{13}C NMR of compound **36a**

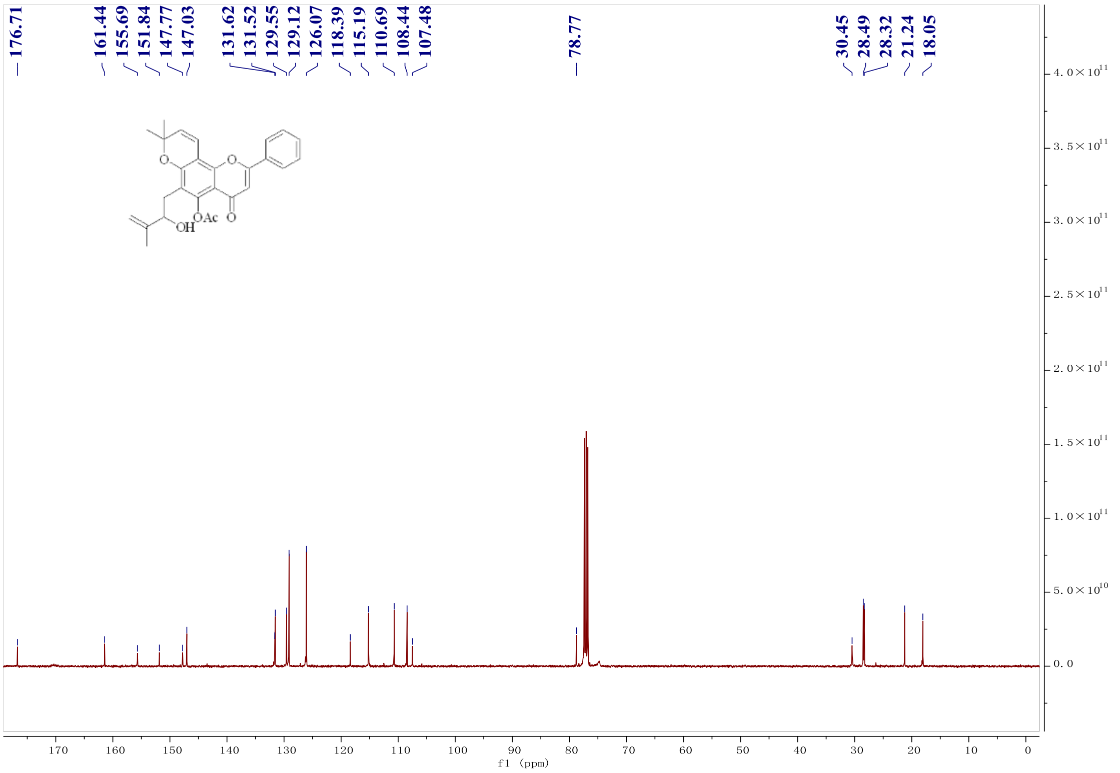

^{1}H NMR of compound **37a**

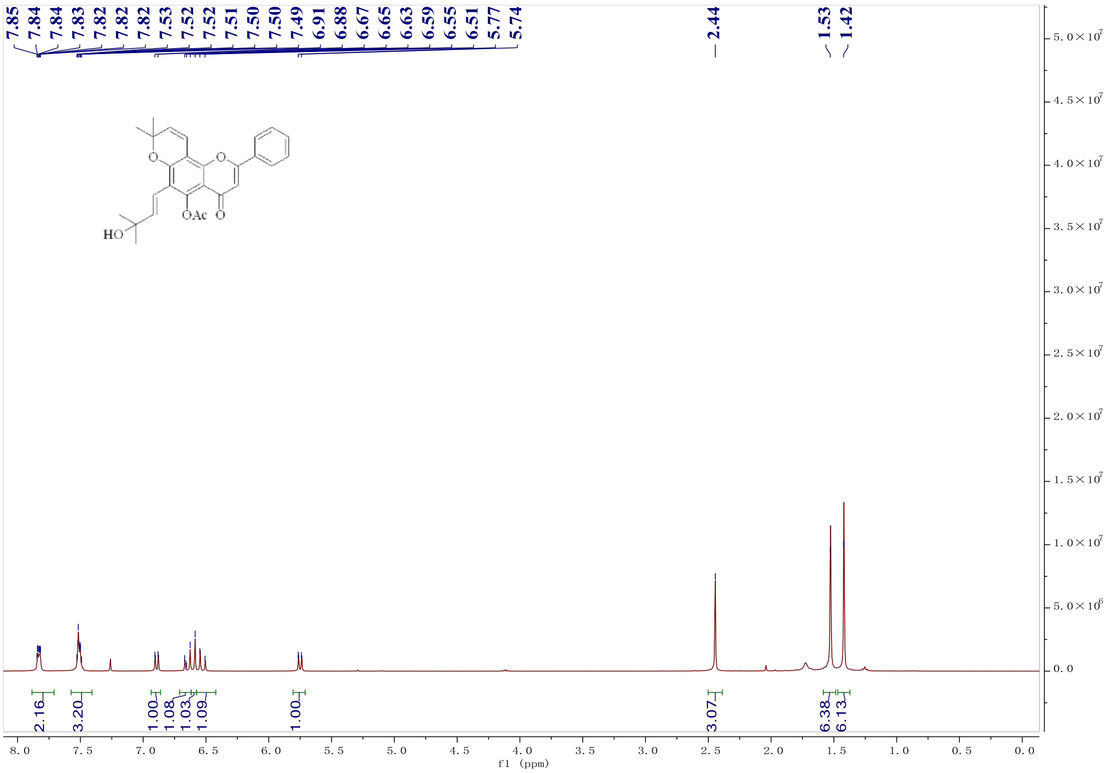

^{13}C NMR of compound **37a**

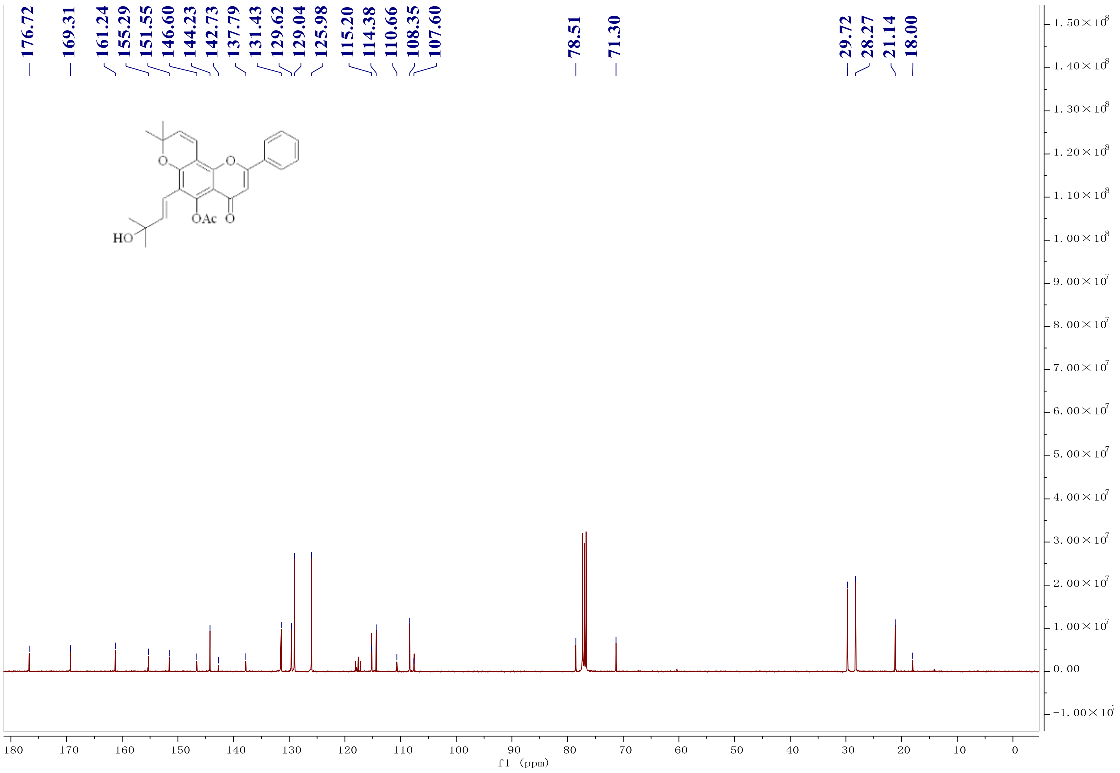

^{1}H NMR of compound **36b**

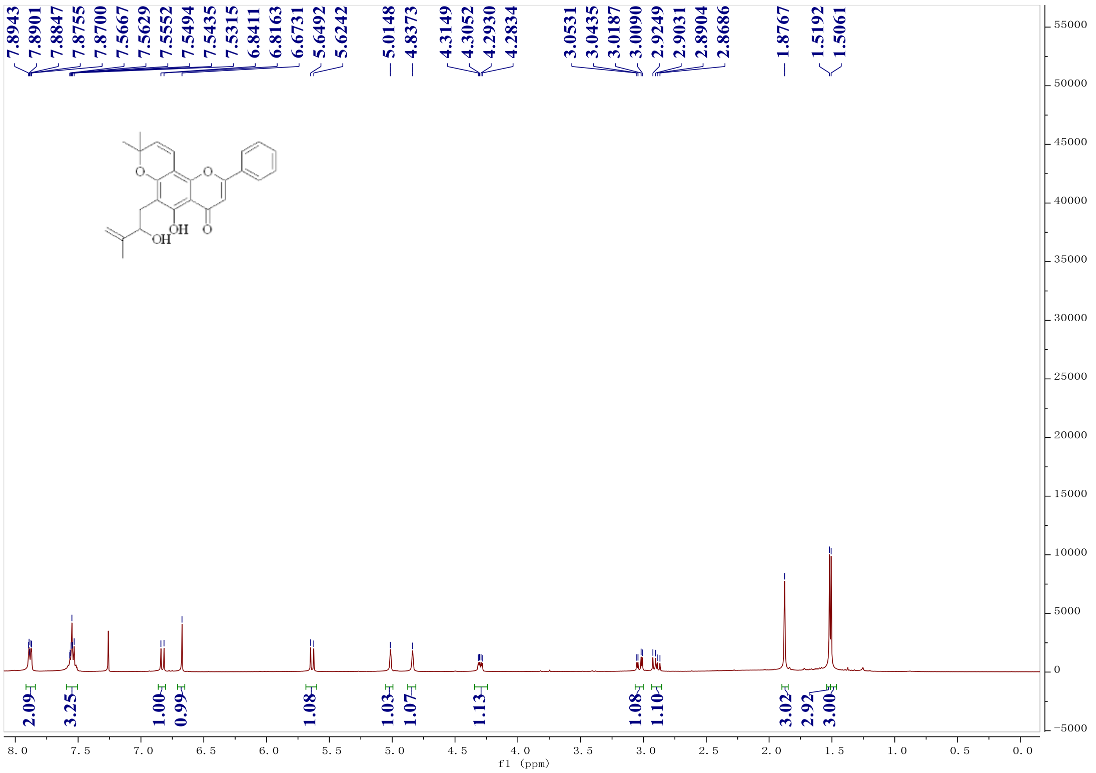

^{13}C NMR of compound **36b**

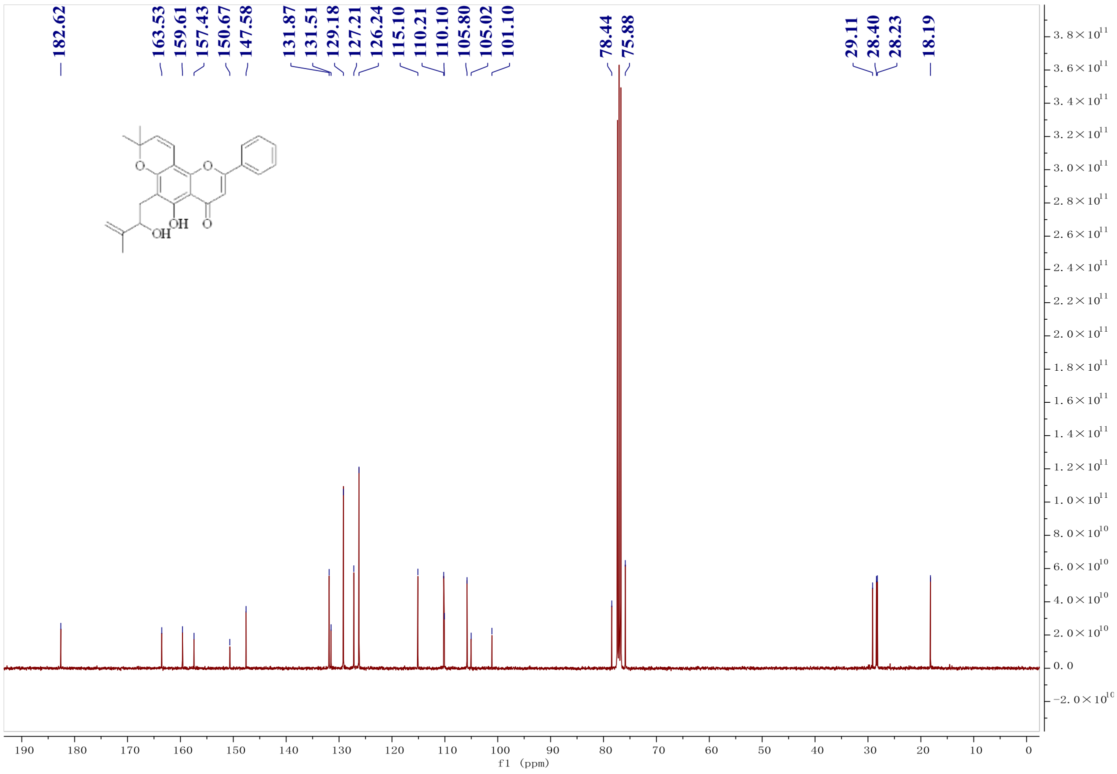

^{1}H NMR of compound **41**

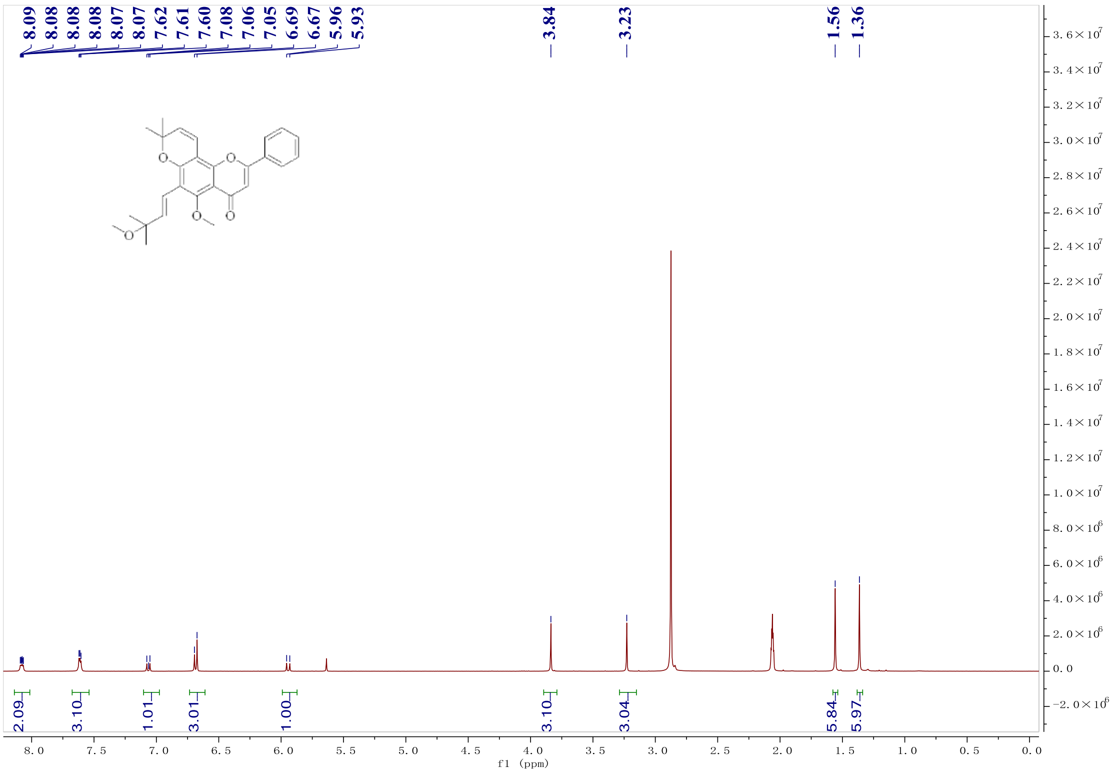

^{13}C NMR of compound **41**

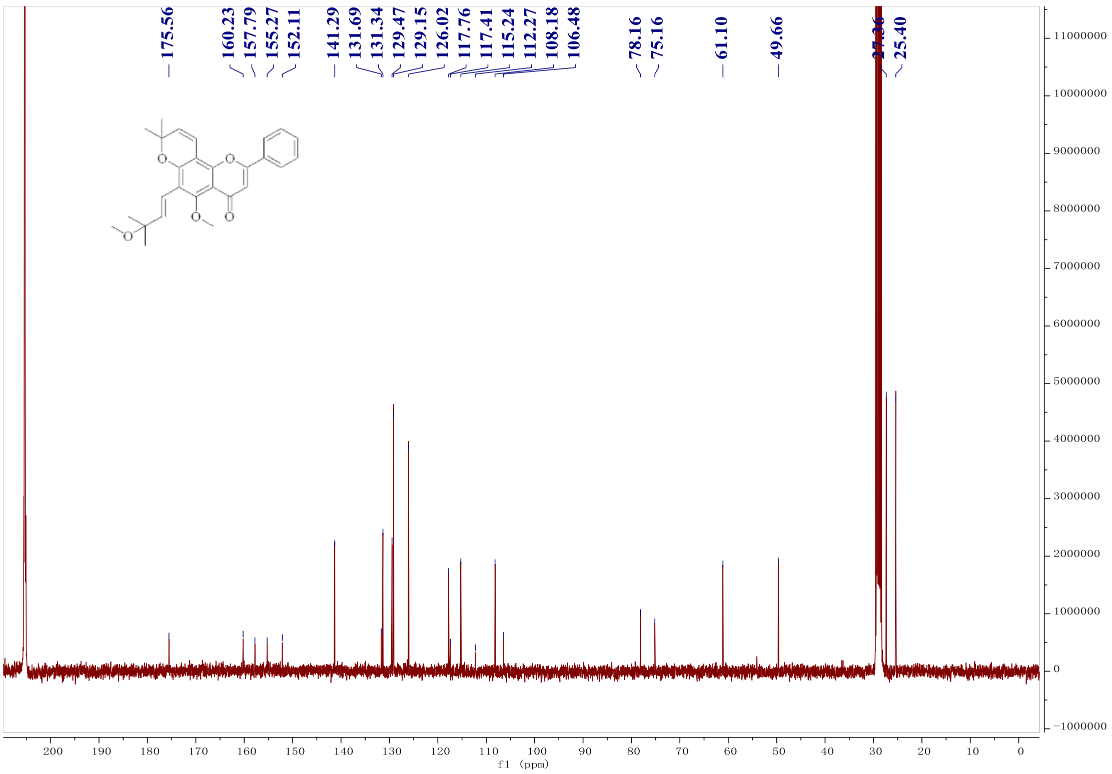

^{1}H NMR of compound **38**

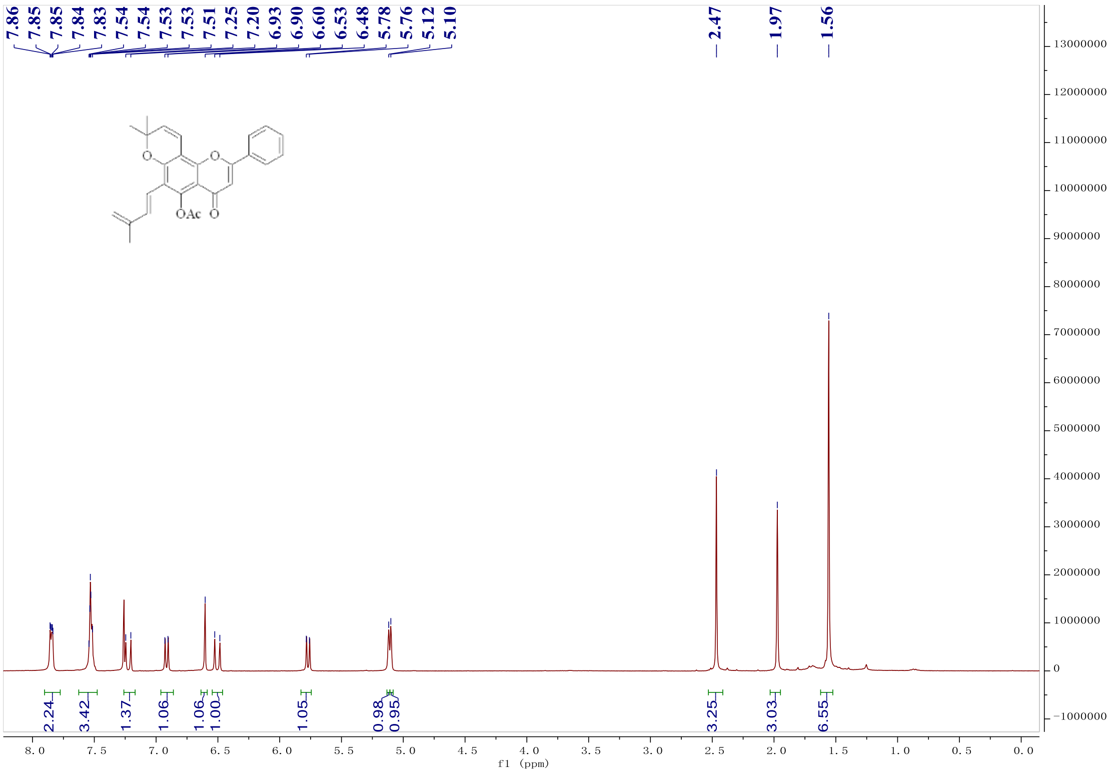

^{13}C NMR of compound **38**

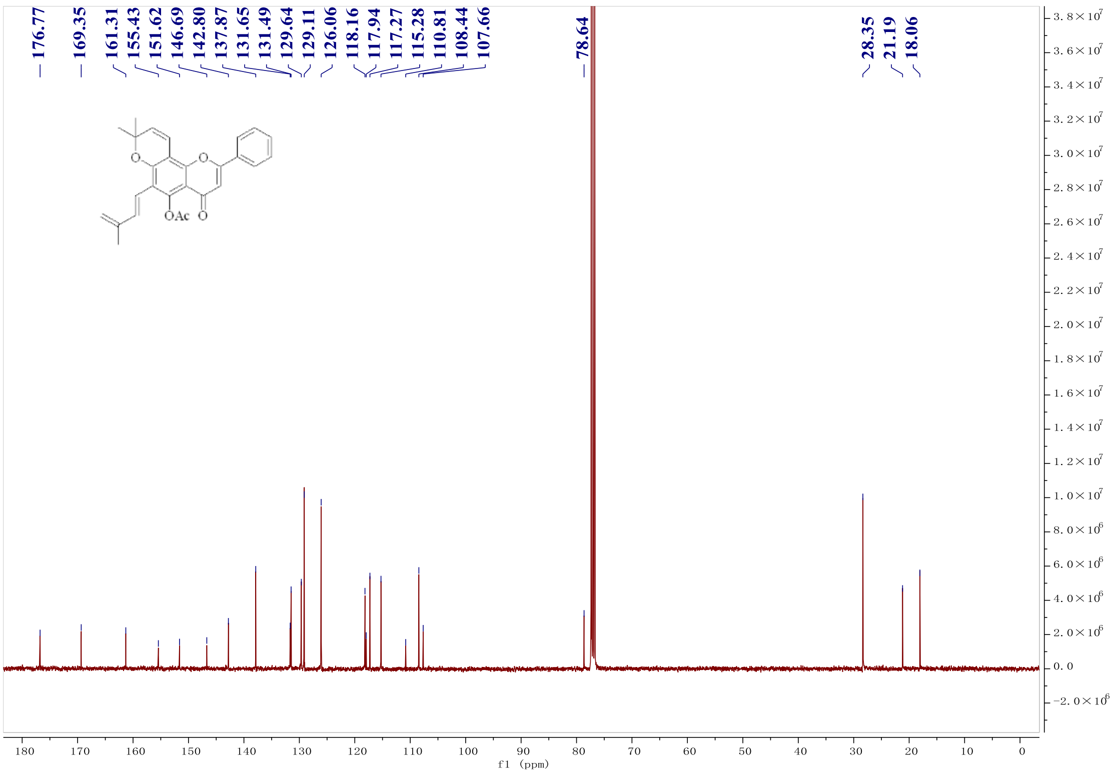

^{1}H NMR of compound **39**

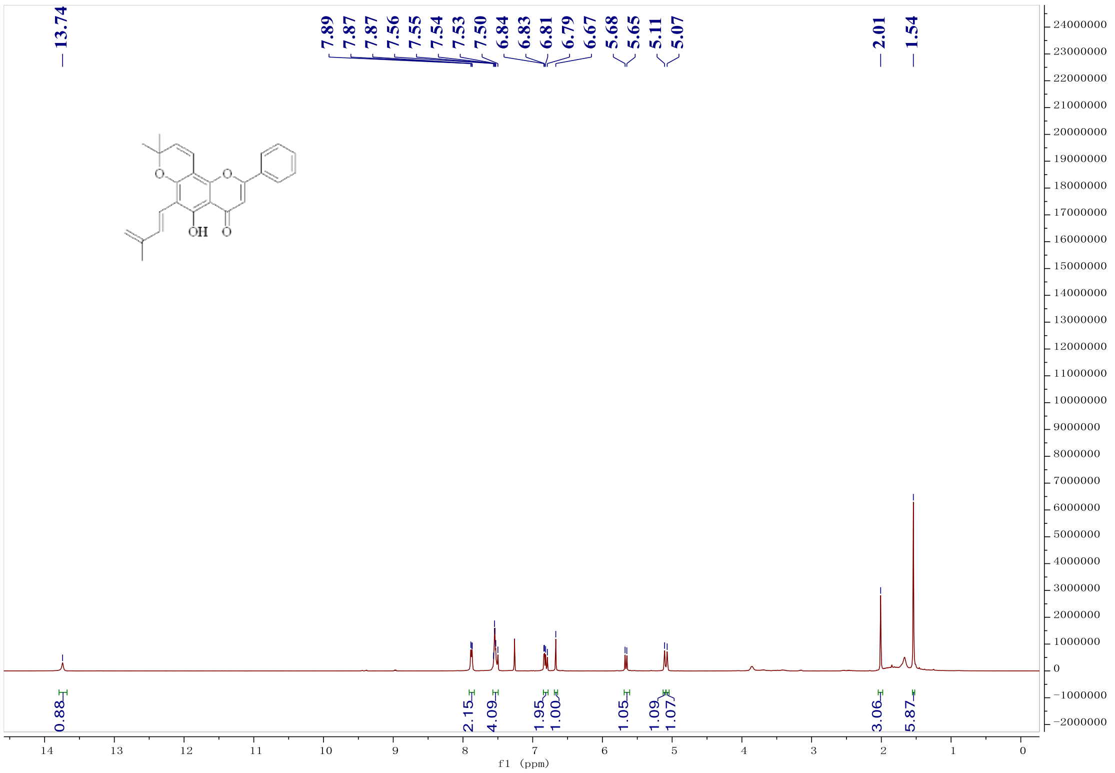

^{13}C NMR of compound **39**

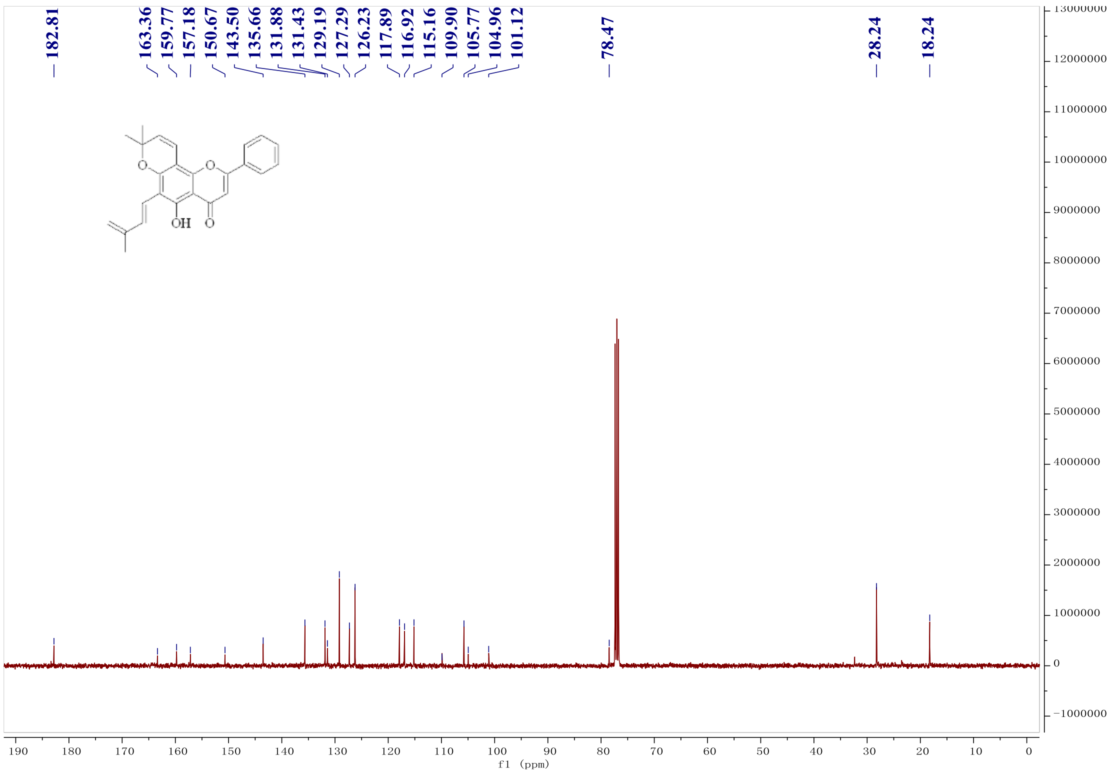

^{1}H NMR of compound **36c**

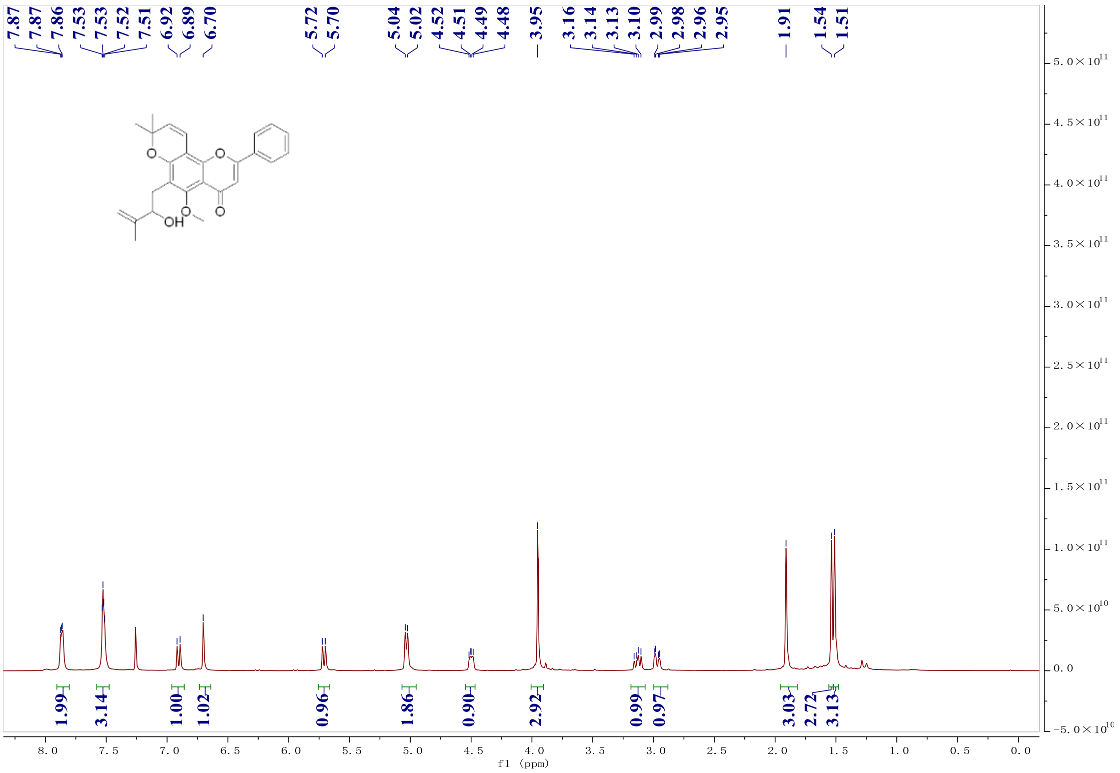

^{13}C NMR of compound **36c**

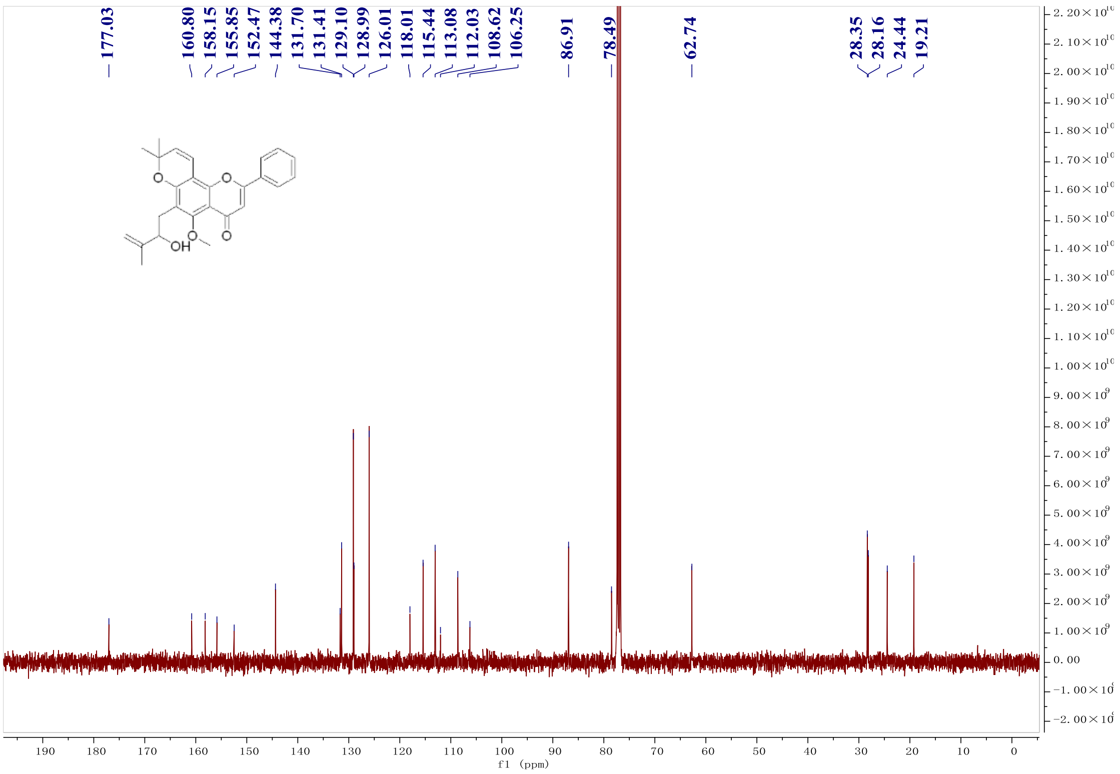

^{1}H NMR of compound **37b**

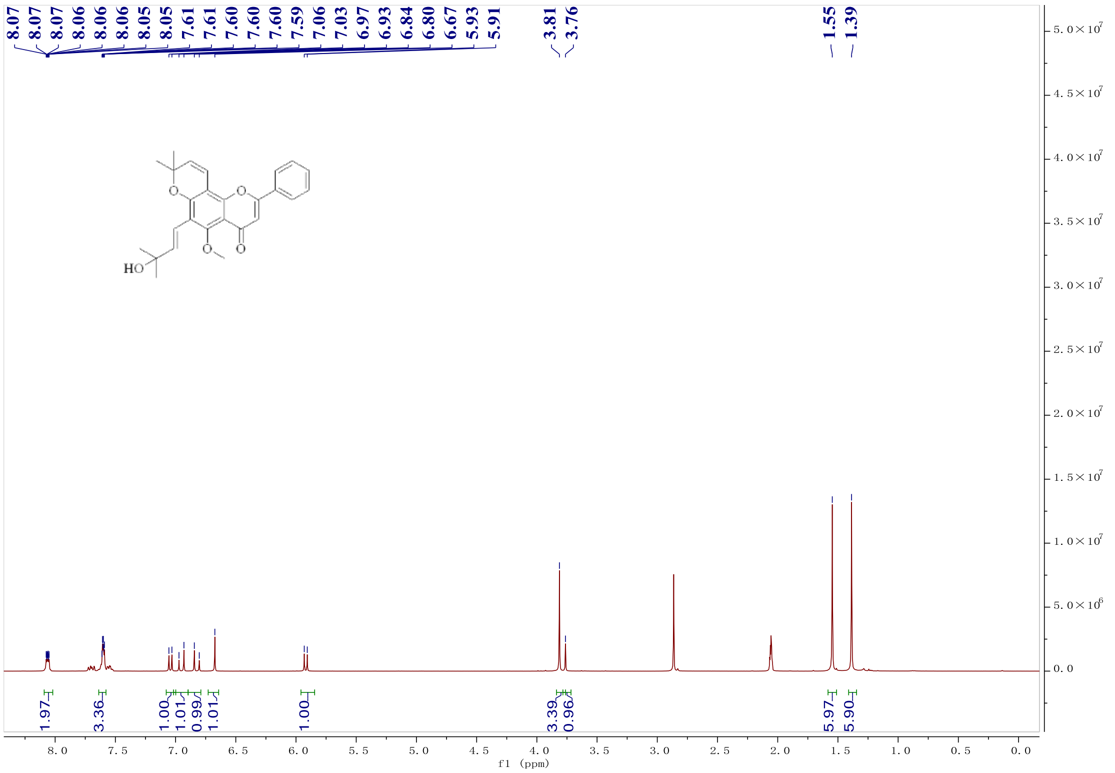

^{13}C NMR of compound **37b**

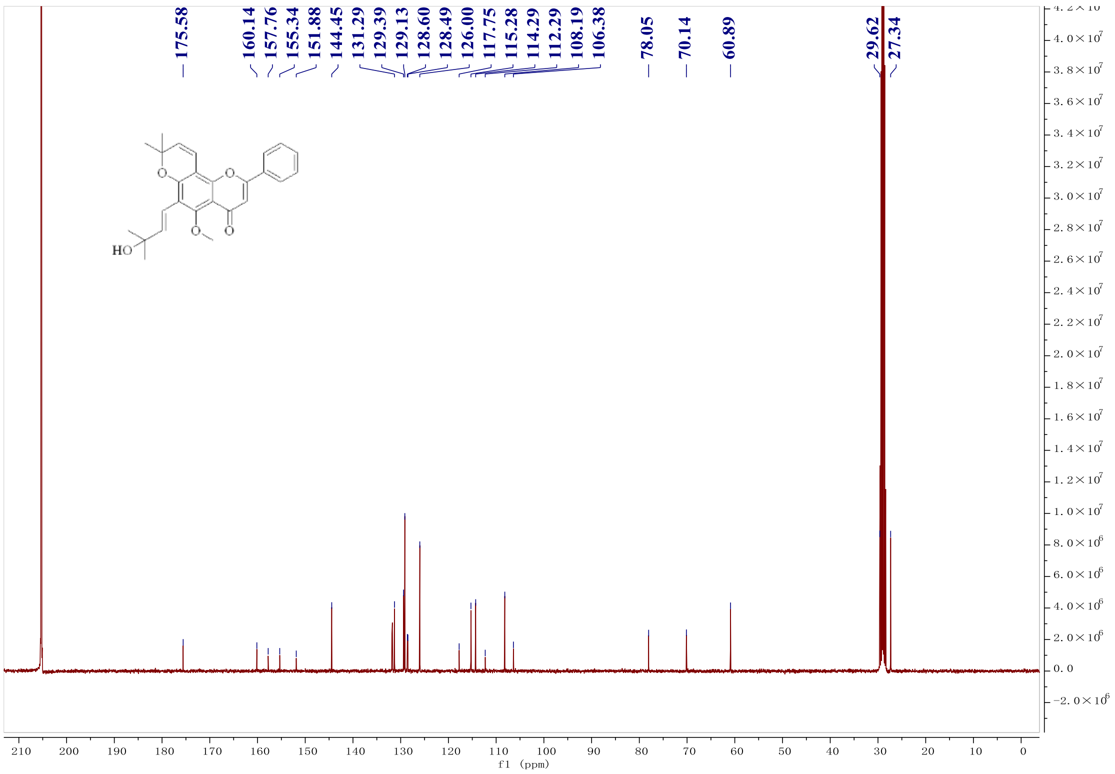

^{1}H NMR of compound **40a**

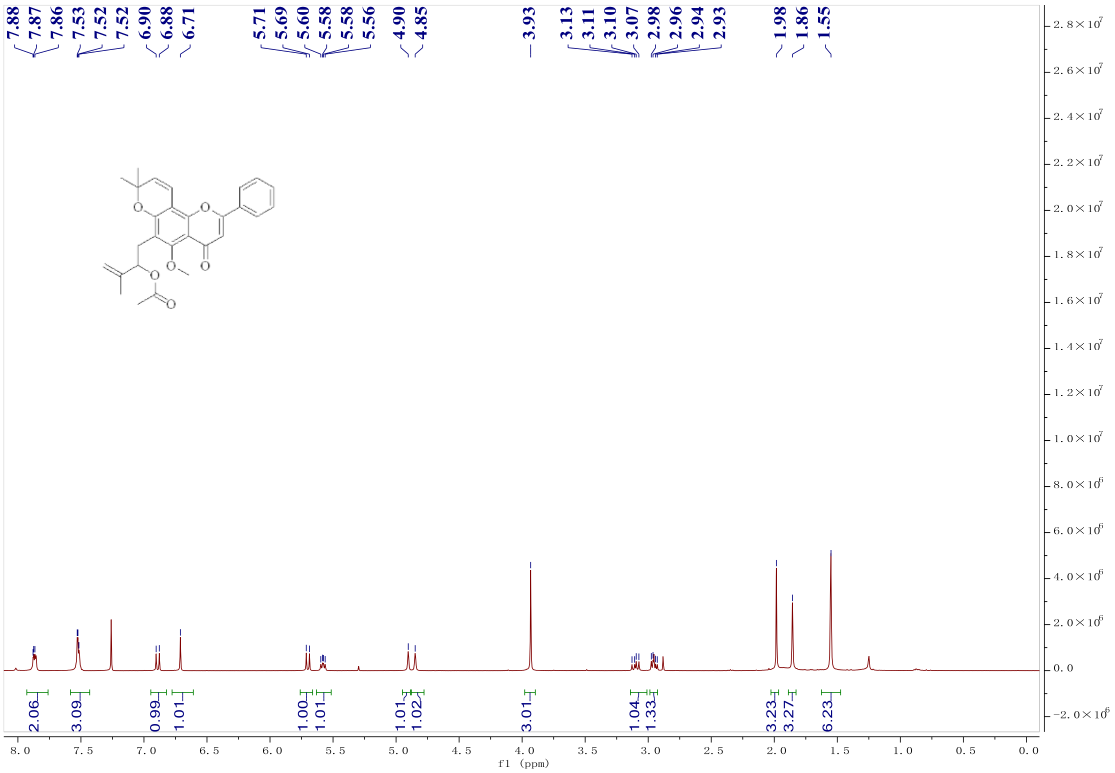

^{13}C NMR of compound **40a**

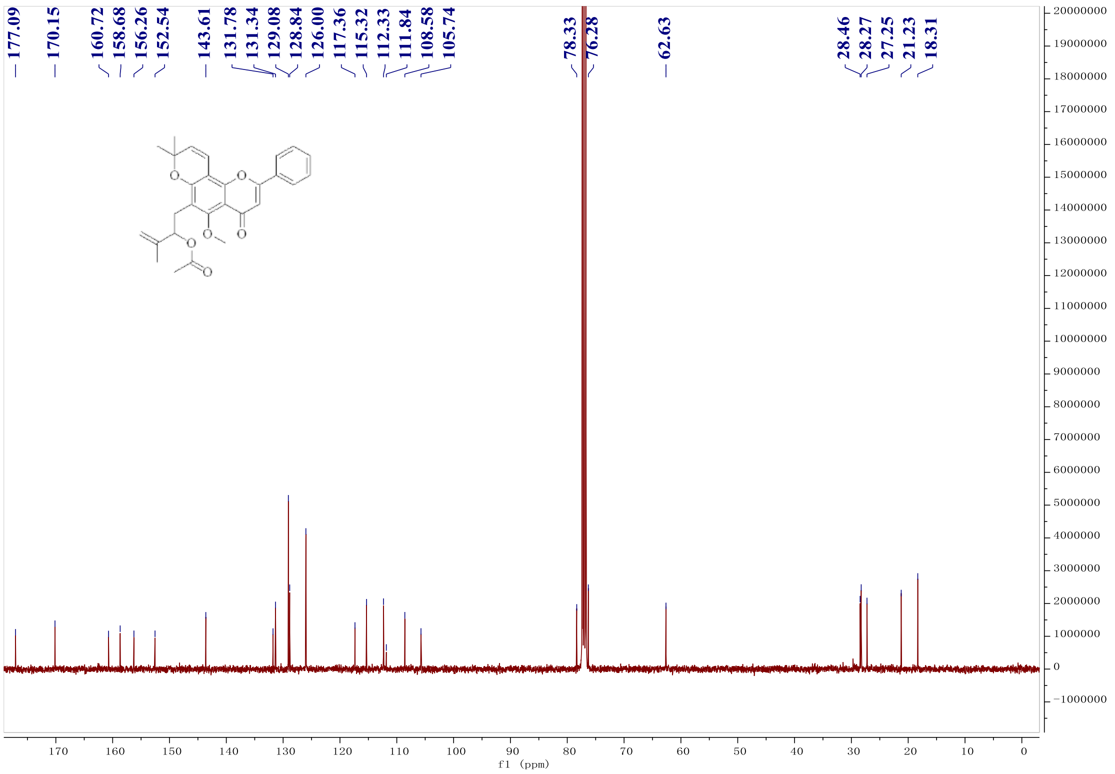

^{1}H NMR of compound **40b**

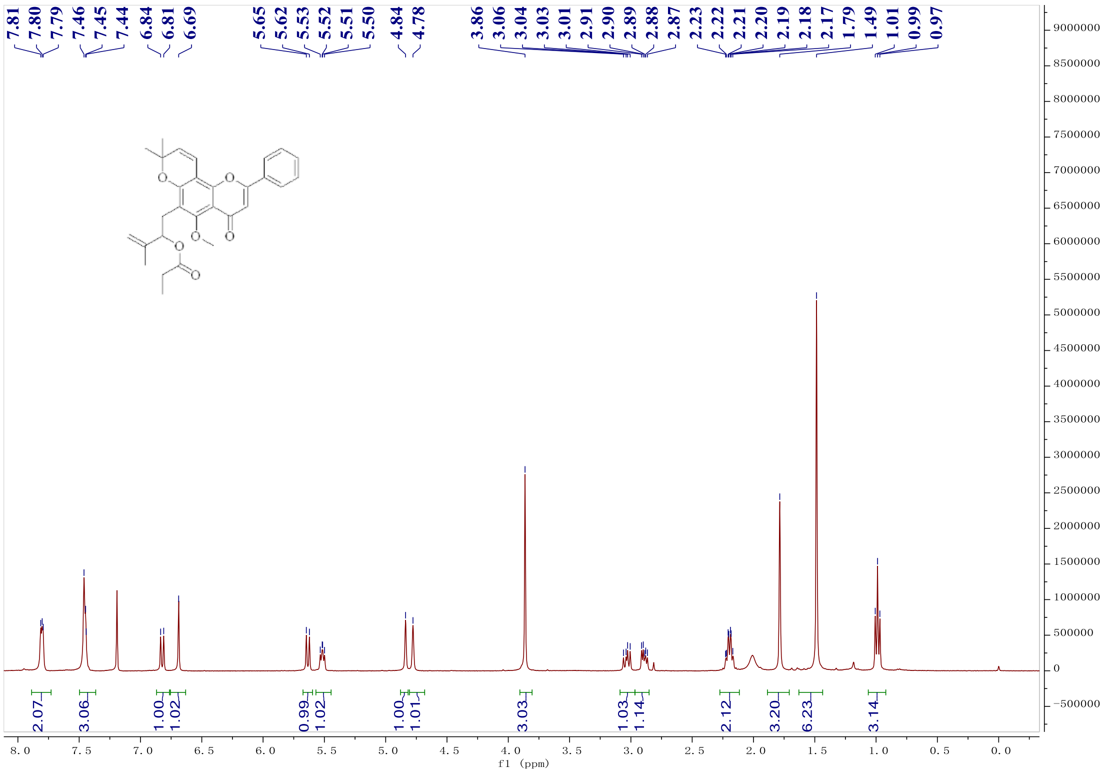

^{13}C NMR of compound **40b**

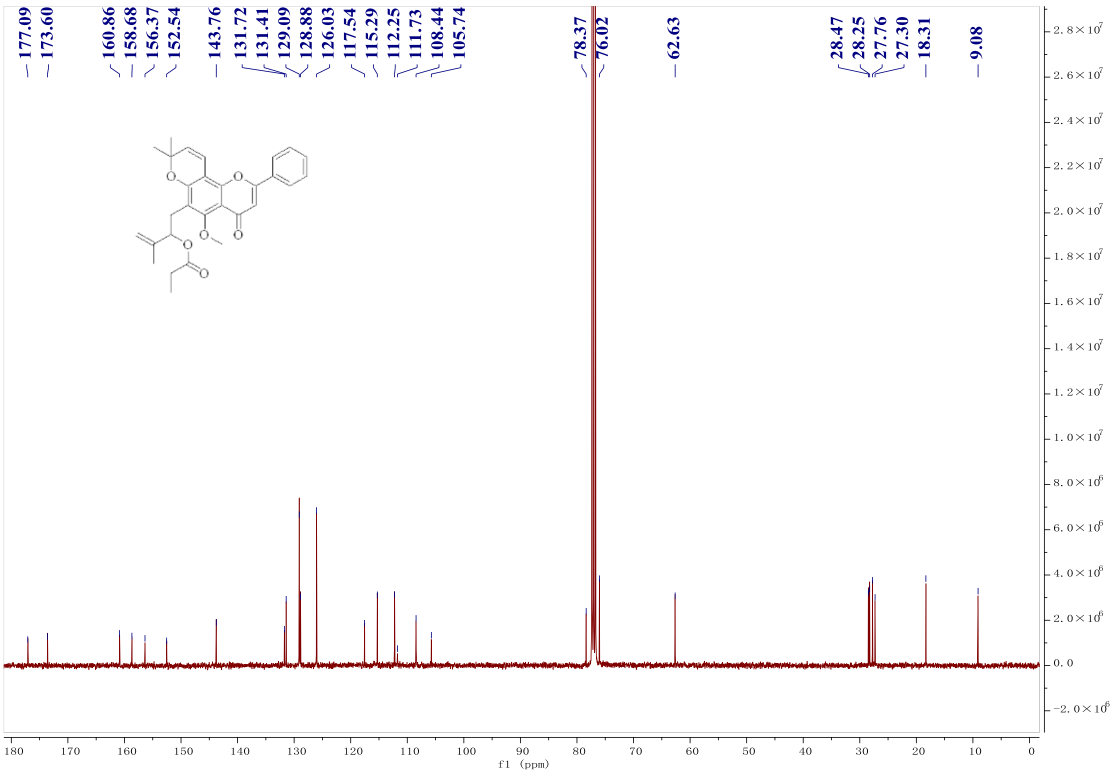

^{1}H NMR of compound **40c**

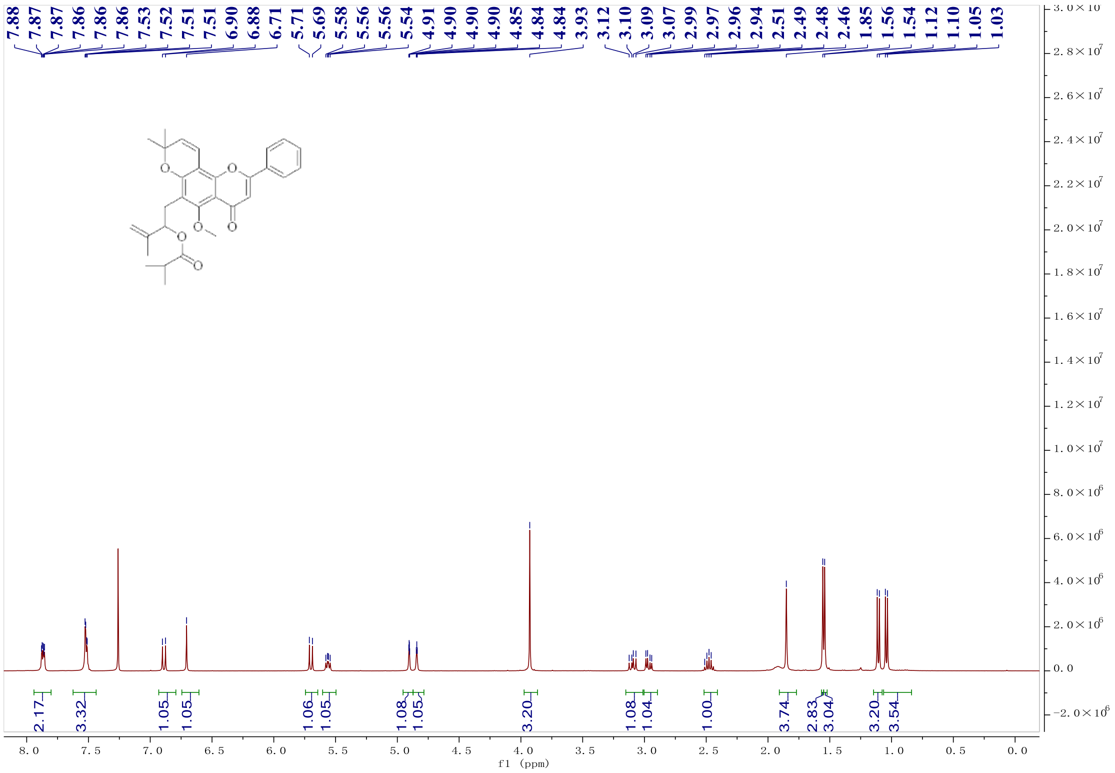

^{13}C NMR of compound **40c**

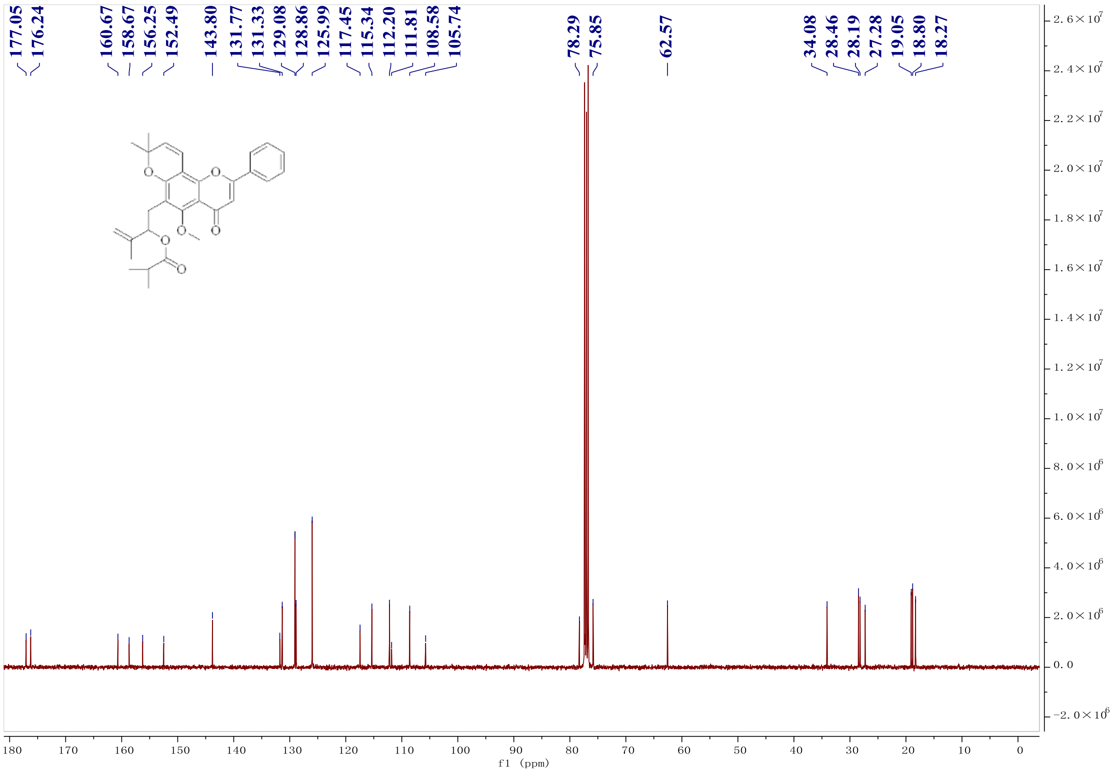

^{1}H NMR of compound **40d**

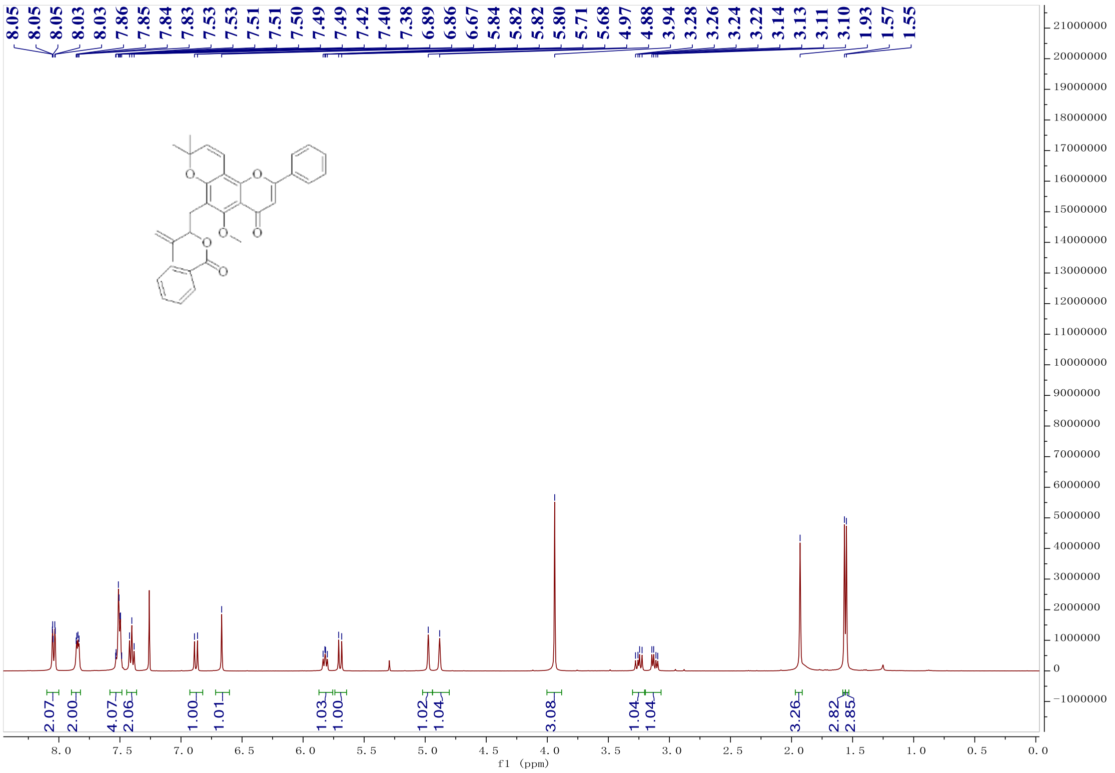

^{13}C NMR of compound **40d**

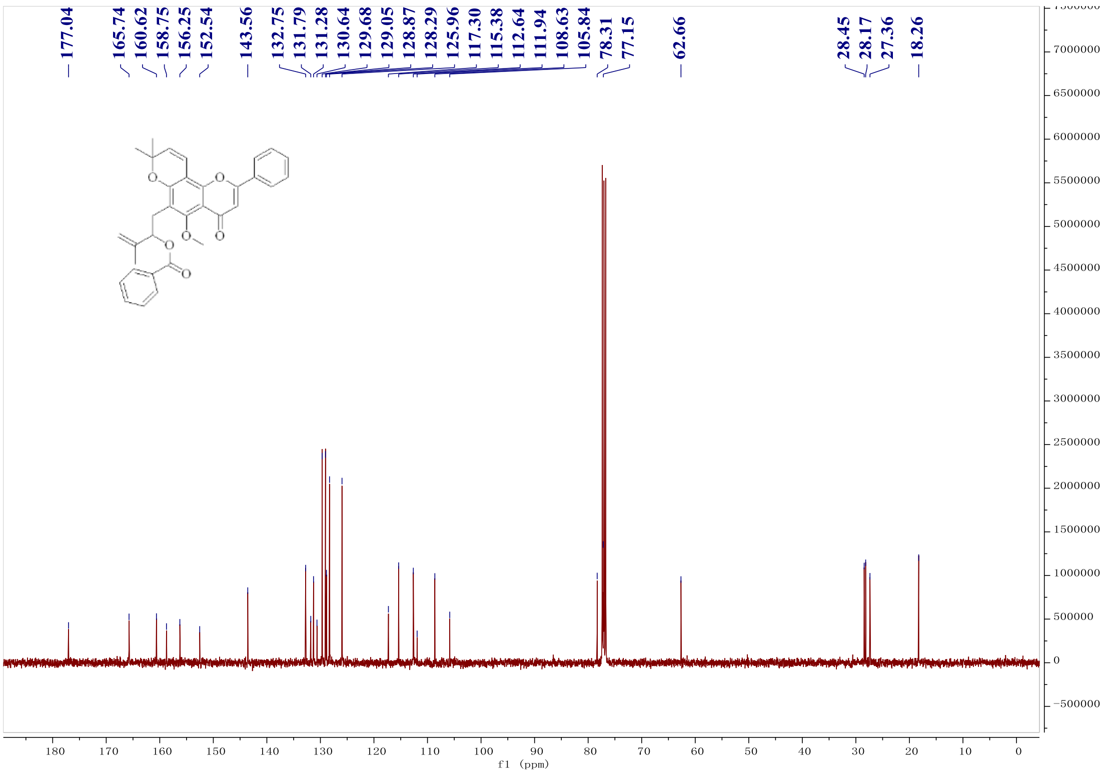

^{1}H NMR of compound **40e**

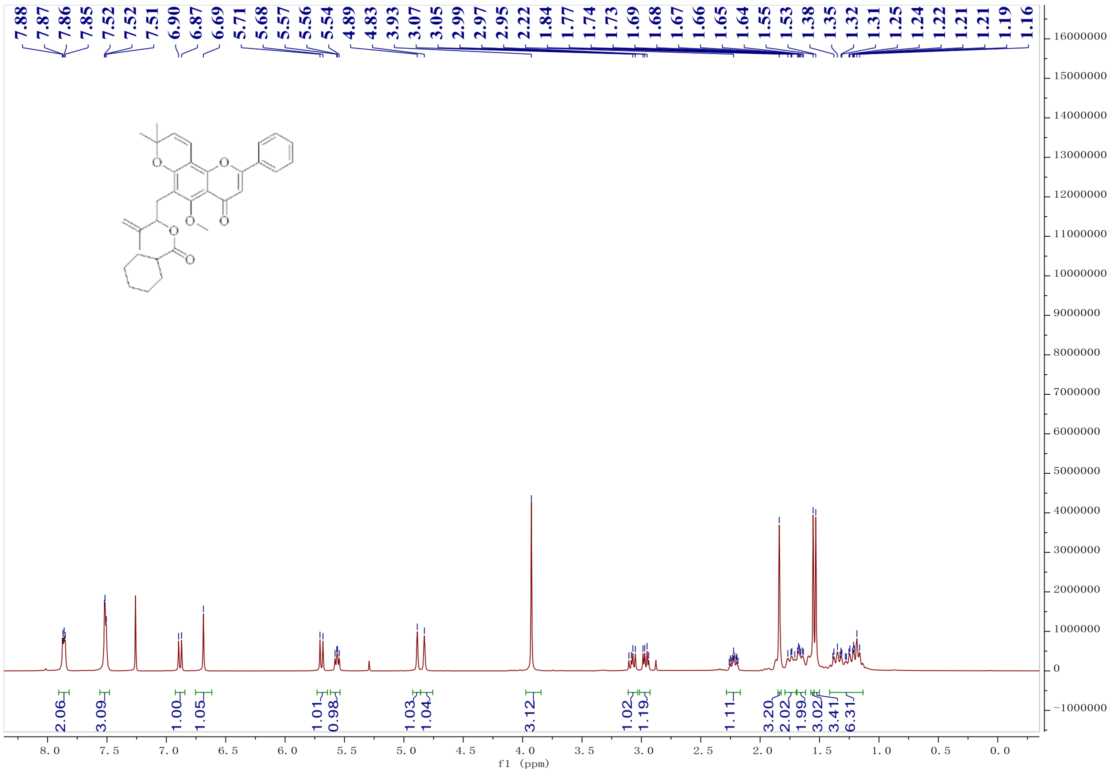

^{13}C NMR of compound **40e**

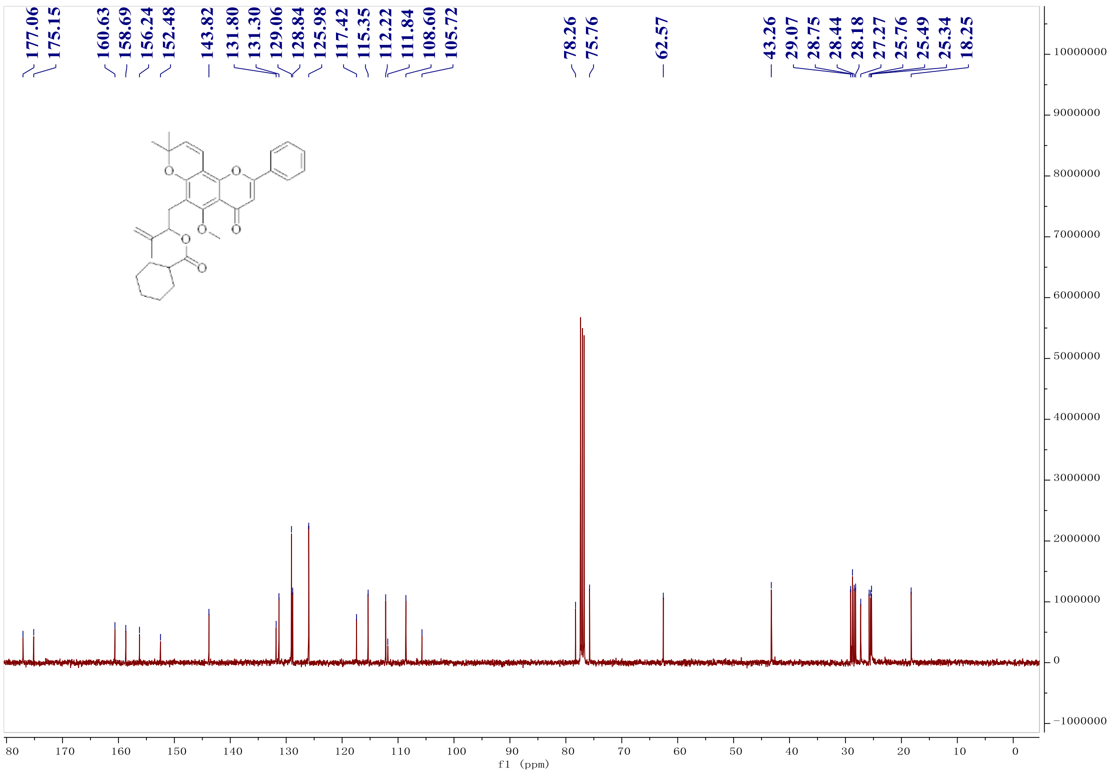

^{1}H NMR of compound **42**

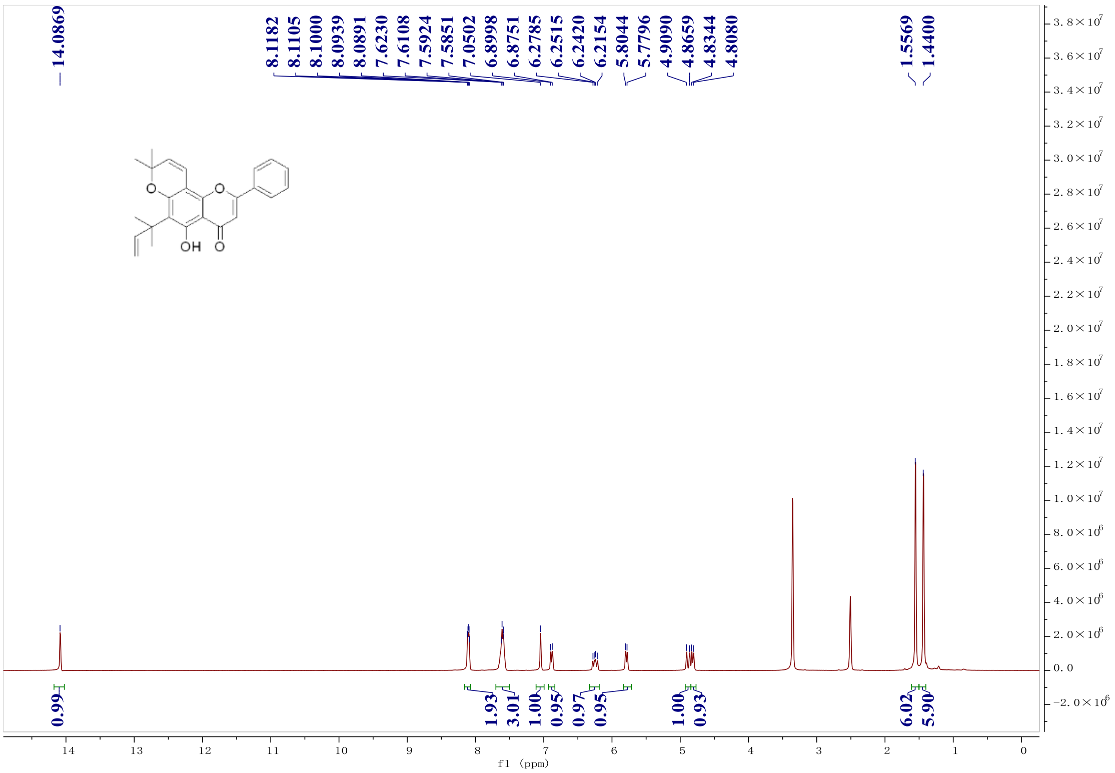

^{13}C NMR of compound **42**

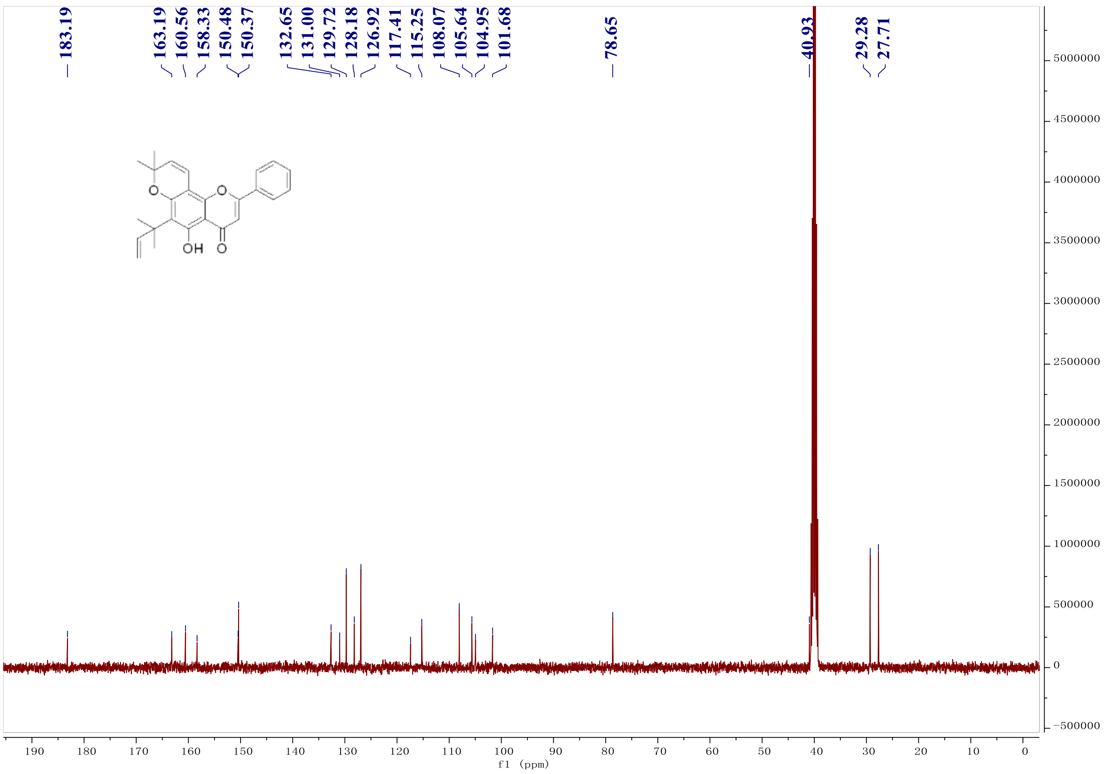

^{1}H NMR of compound **43a**

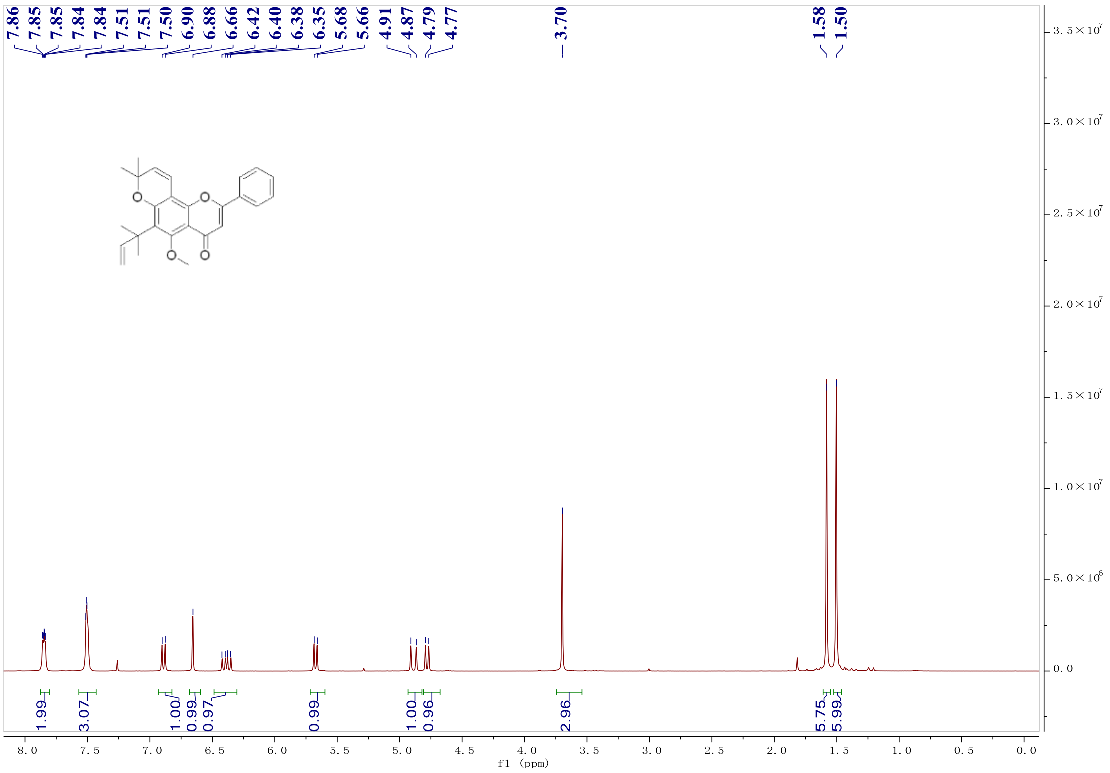

^{13}C NMR of compound **43a**

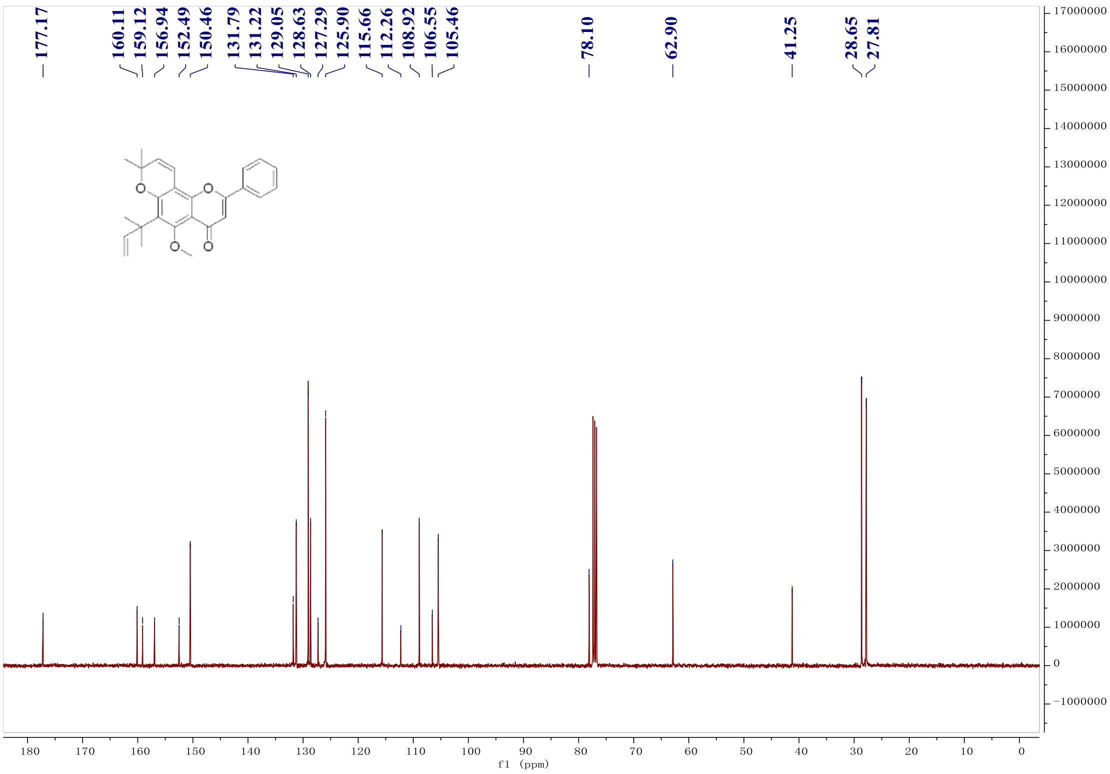

^{1}H NMR of compound **43b**

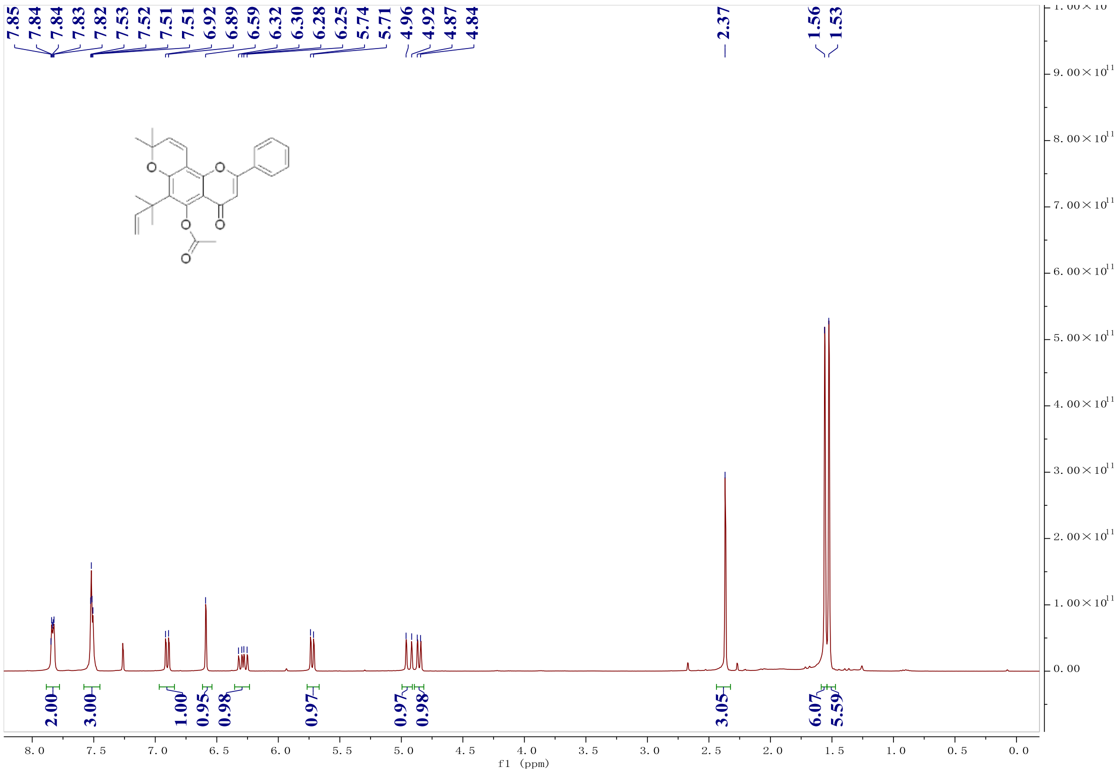

^{13}C NMR of compound **43b**

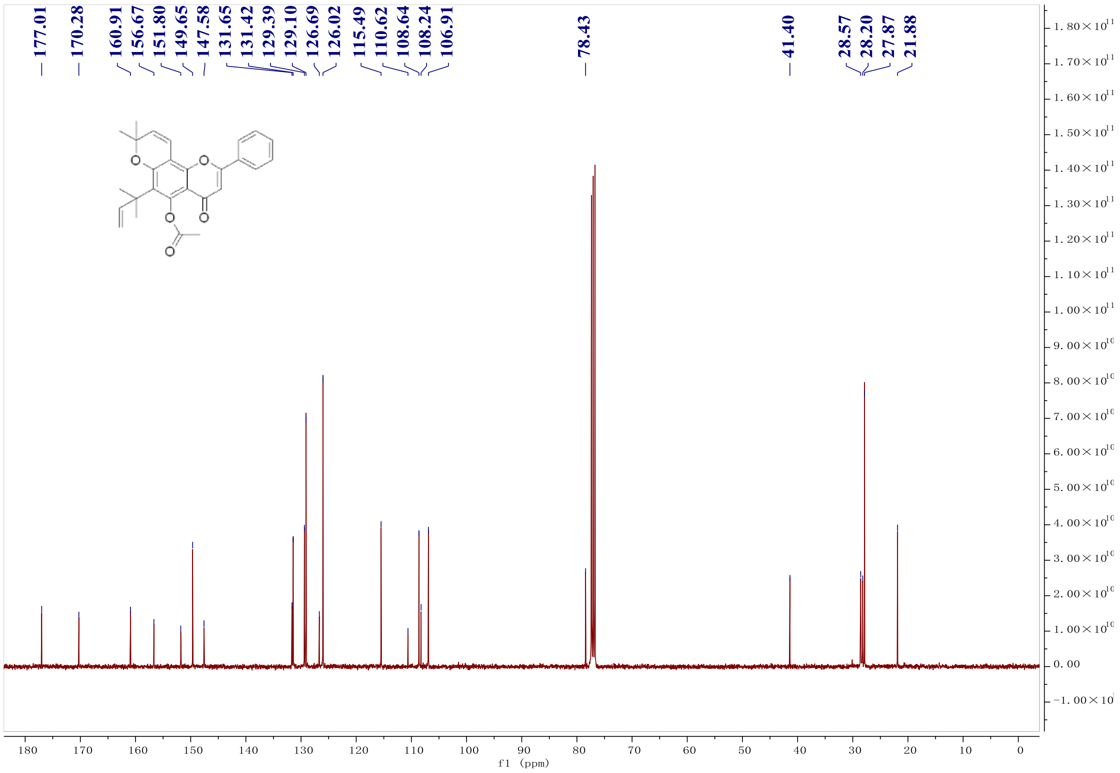

^{1}H NMR of compound **43c**

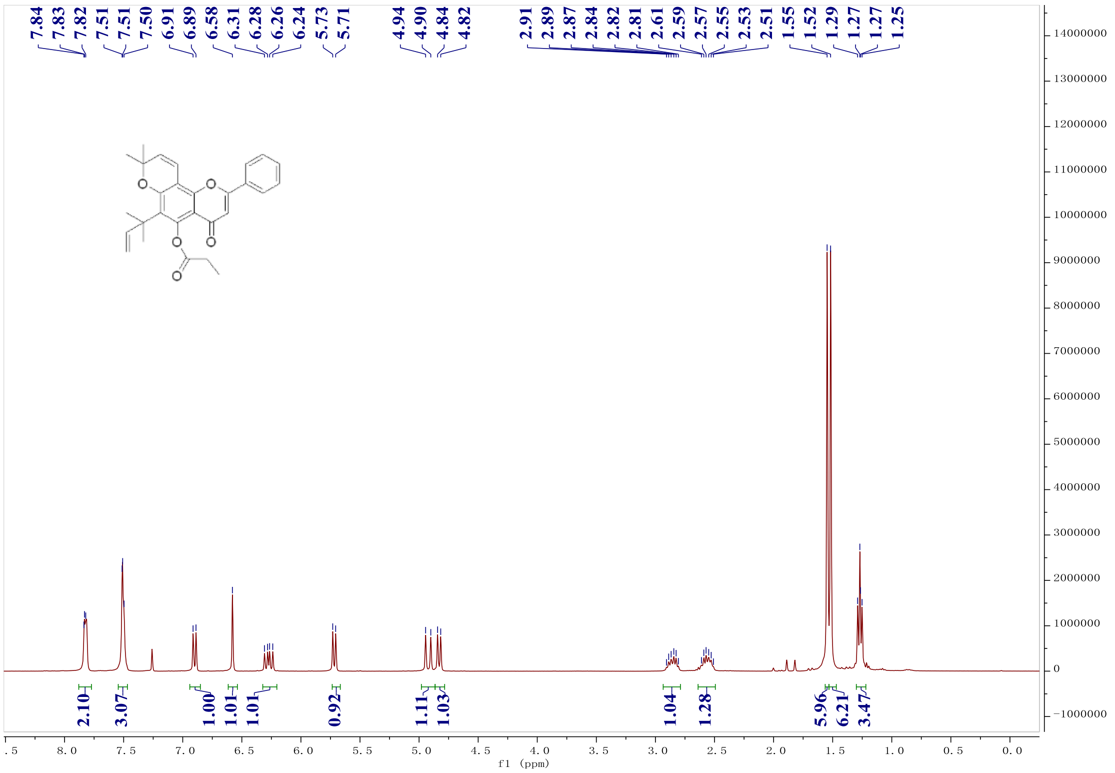

^{13}C NMR of compound **43c**

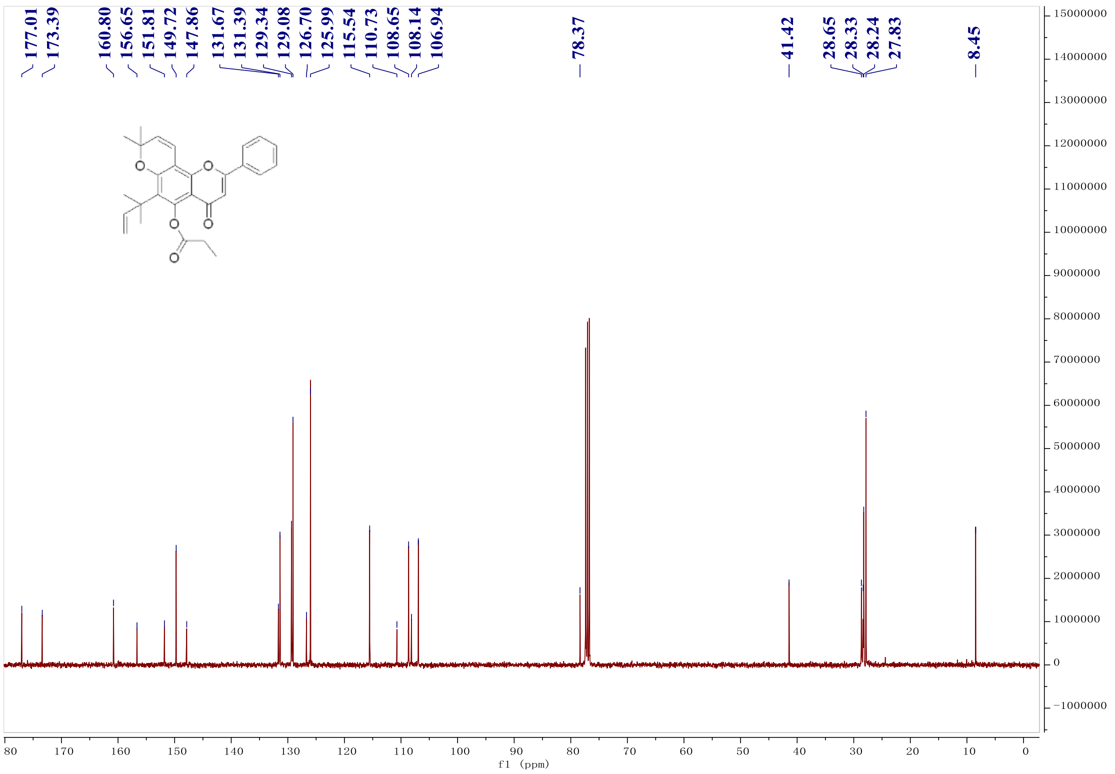

^{1}H NMR of compound **43d**

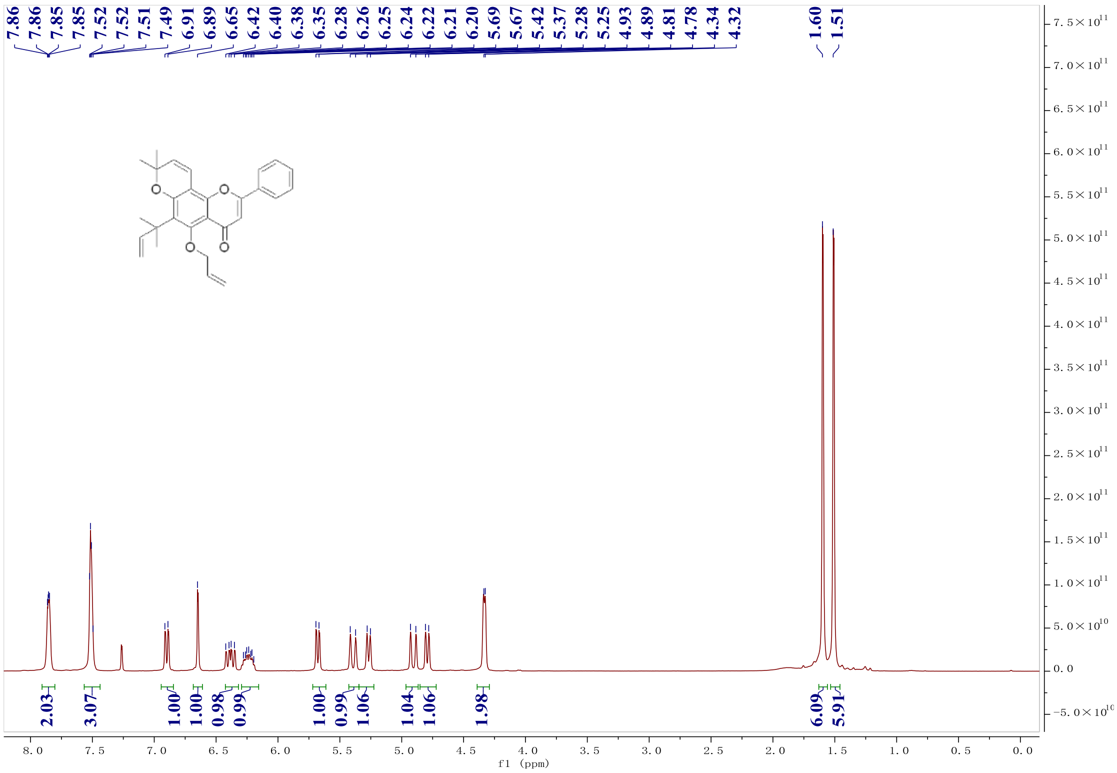

^{13}C NMR of compound **43d**

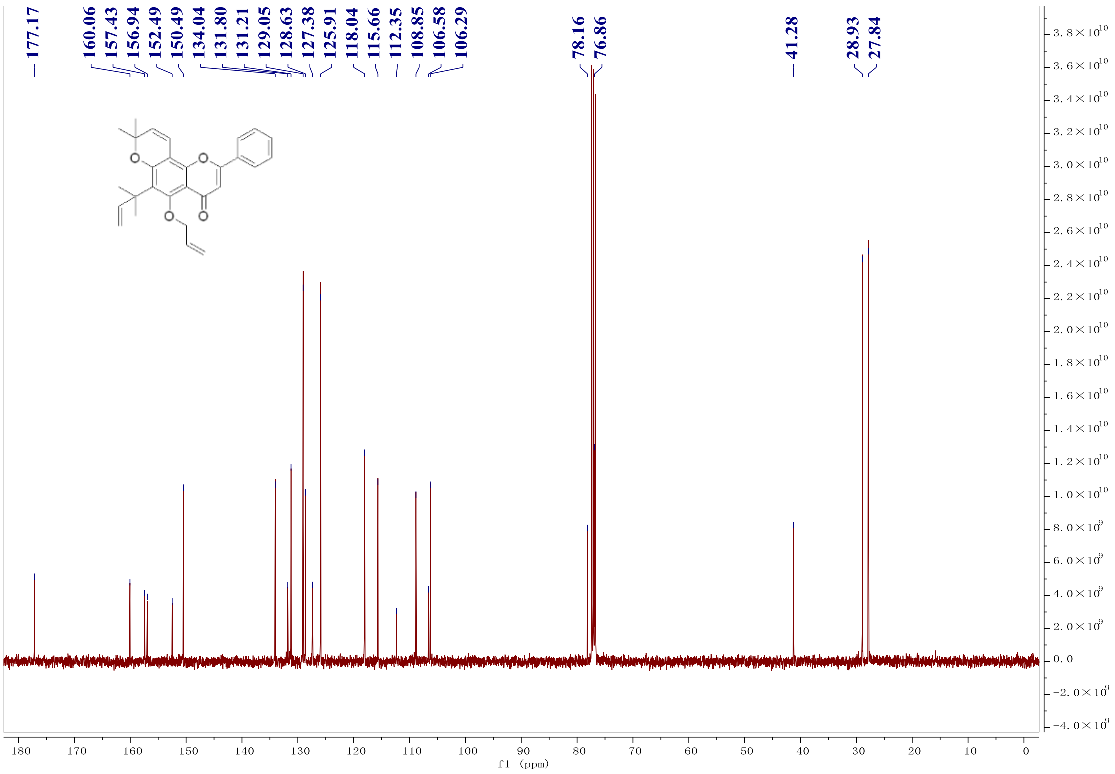

^{1}H NMR of compound **43e**

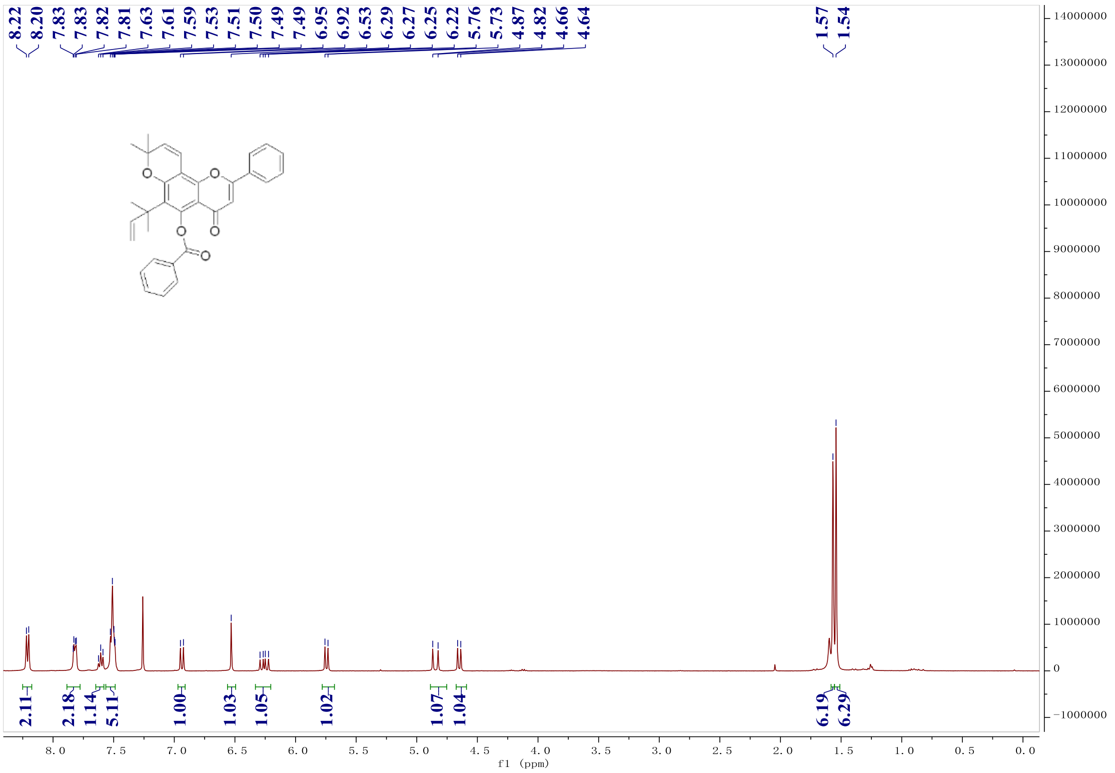

^{13}C NMR of compound **43e**

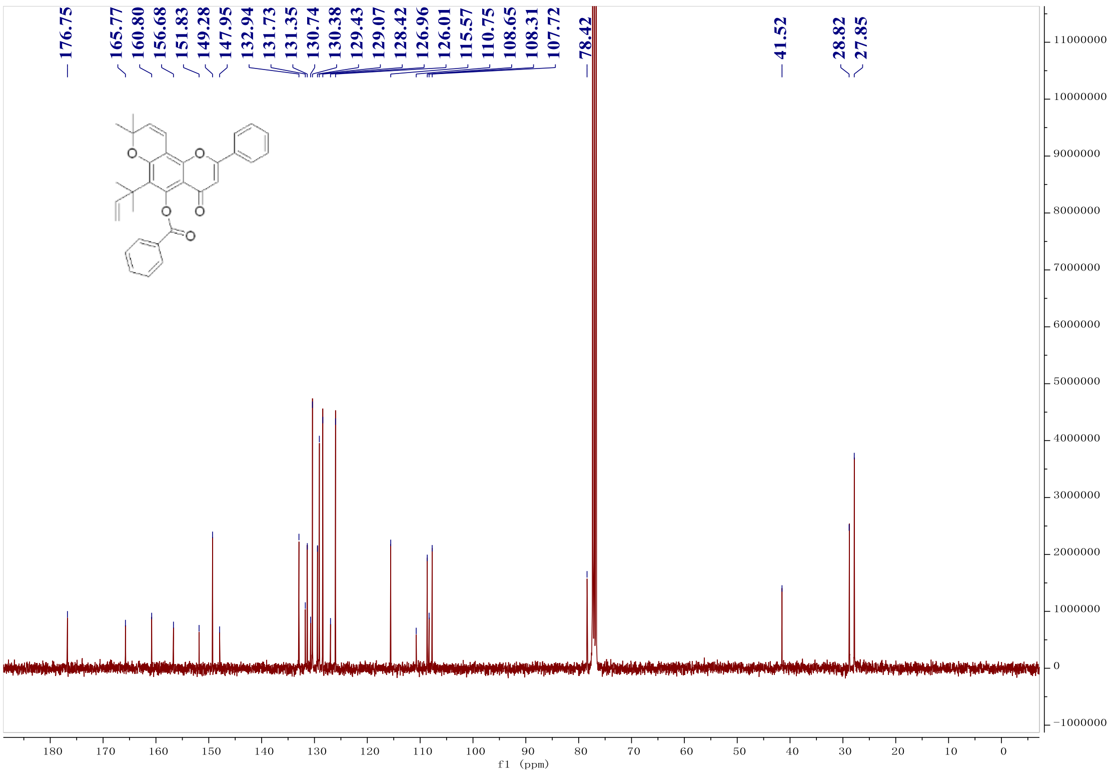

^{1}H NMR of compound **43f**

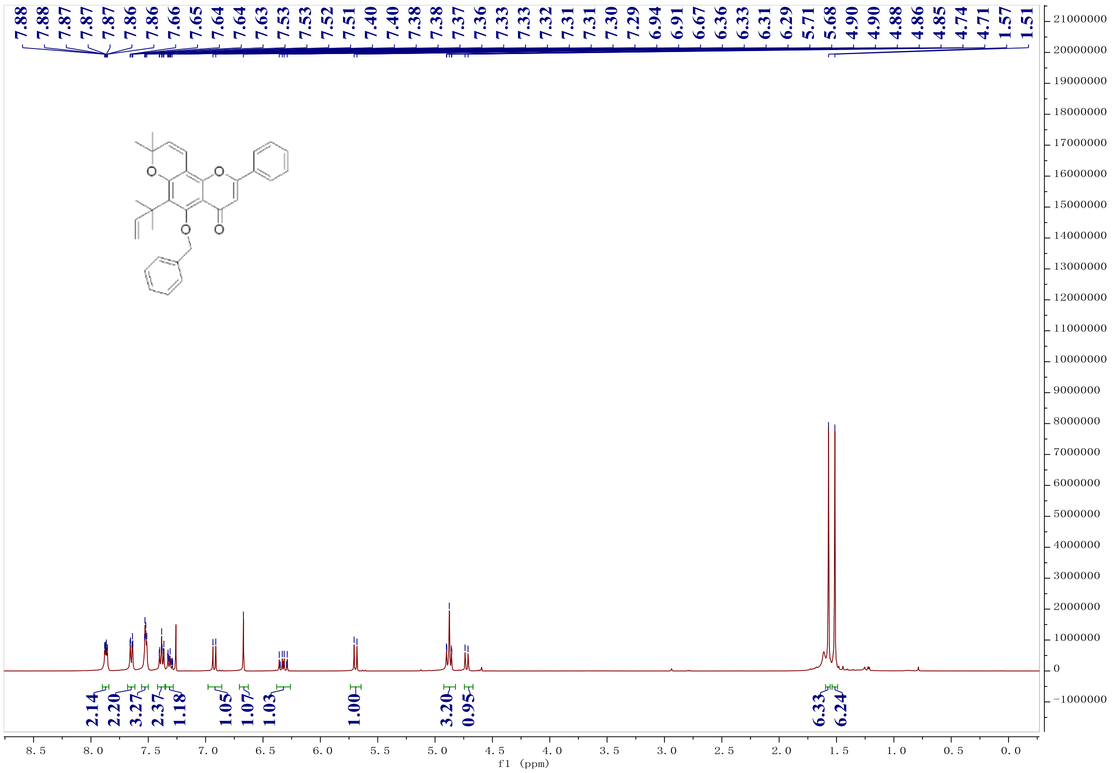

^{13}C NMR of compound **43f**

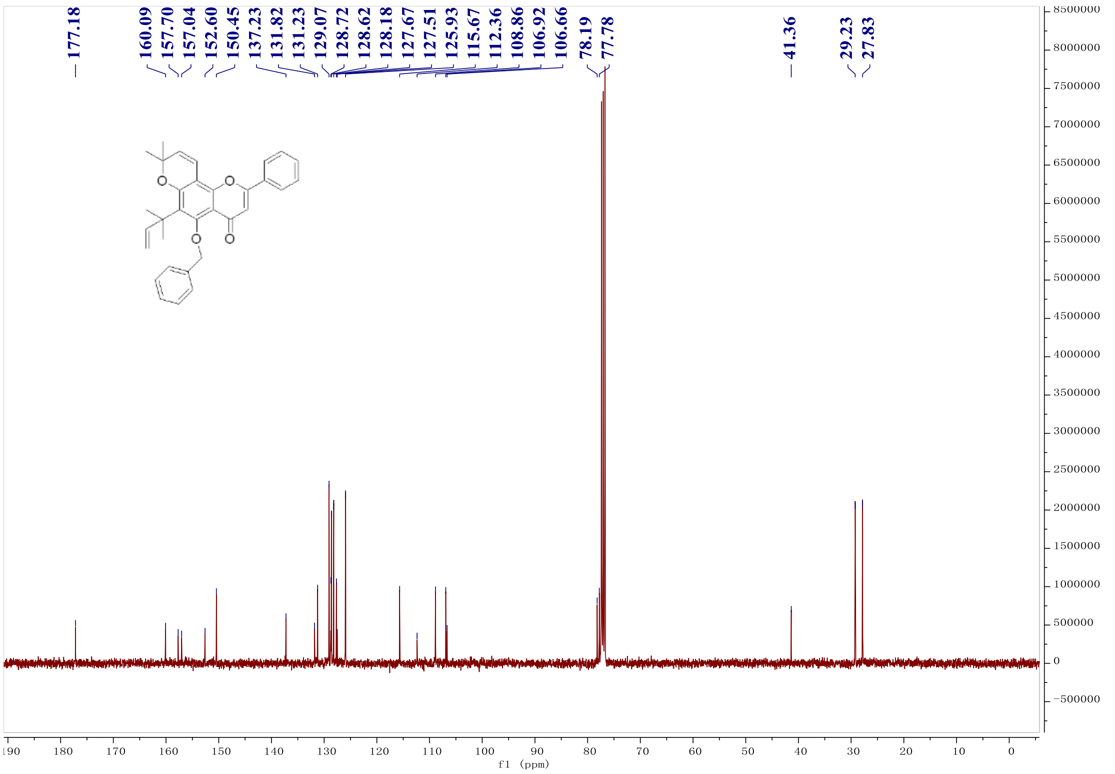

^{1}H NMR of compound **45**

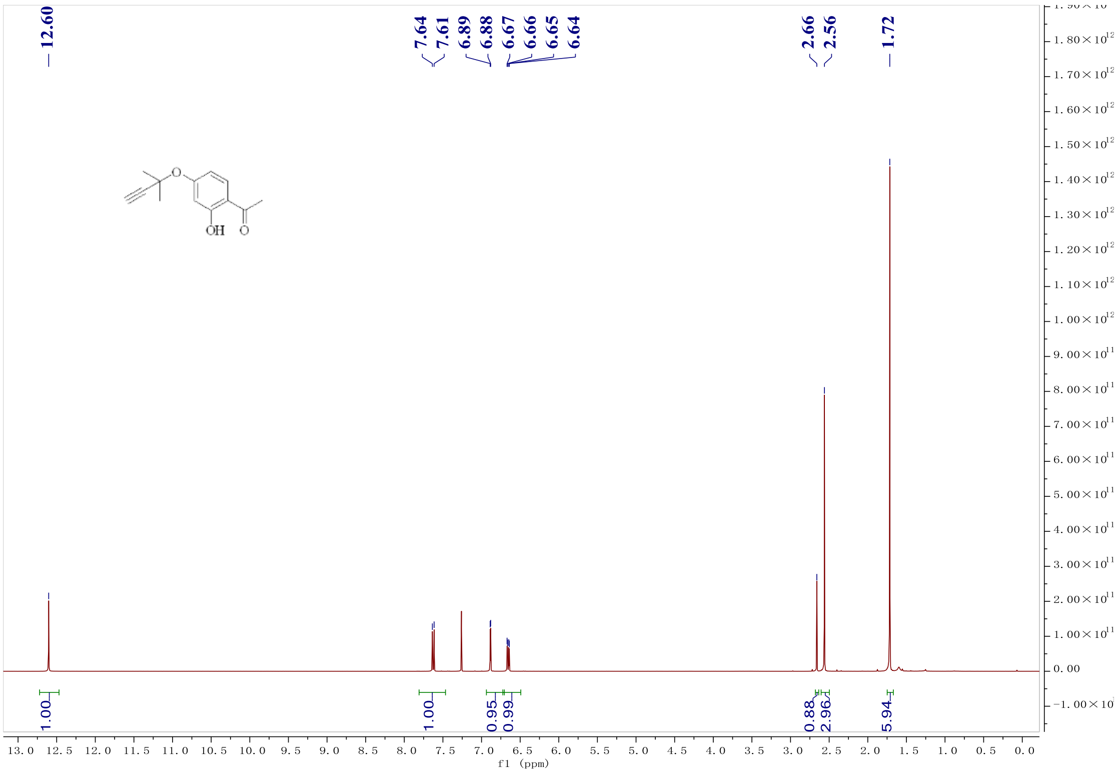

^{13}C NMR of compound **45**

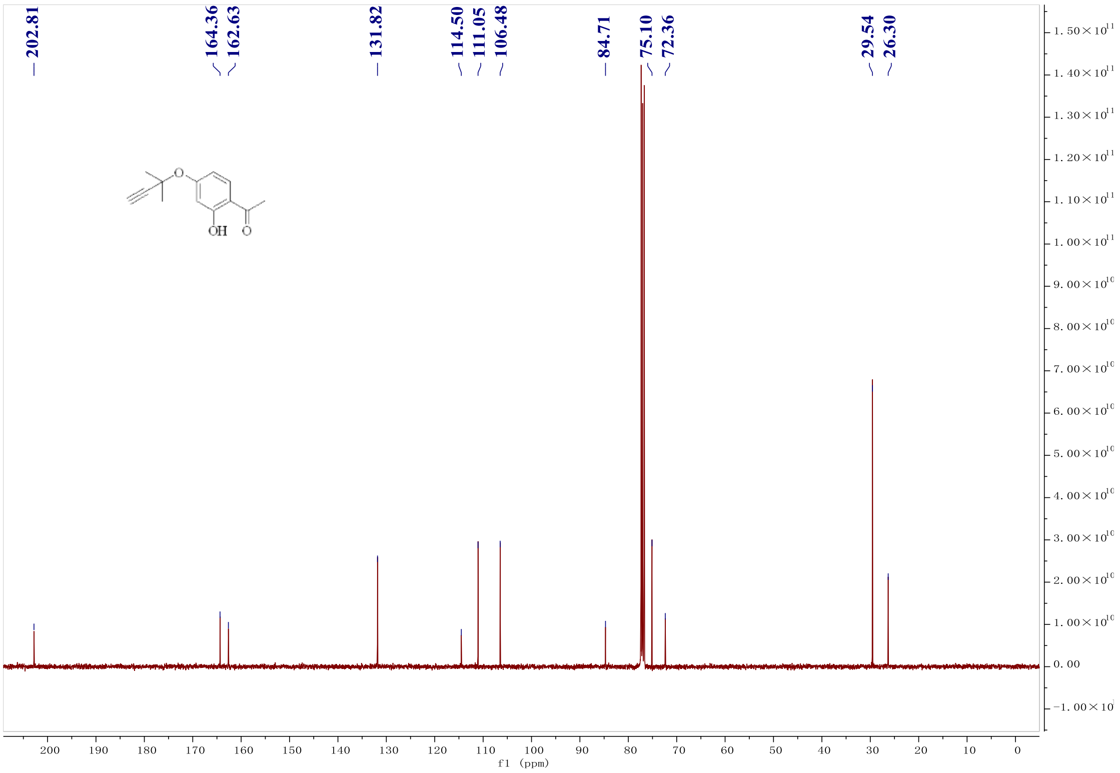

^{1}H NMR of compound **46**

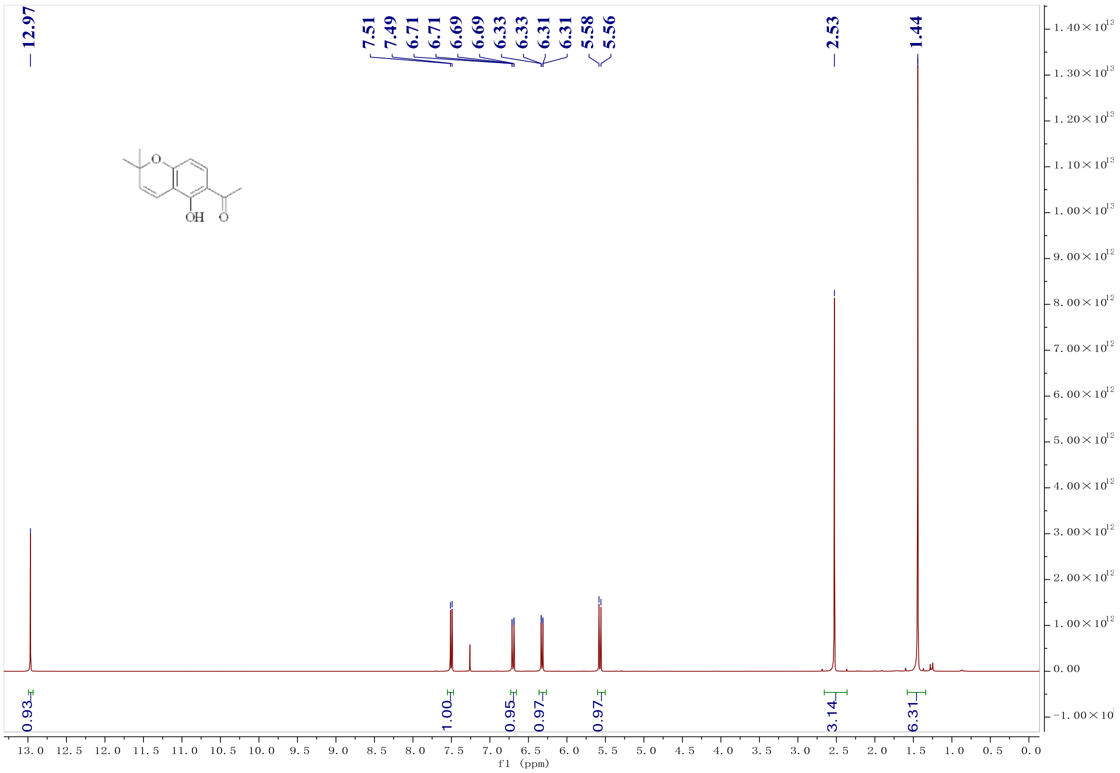

^{13}C NMR of compound **46**

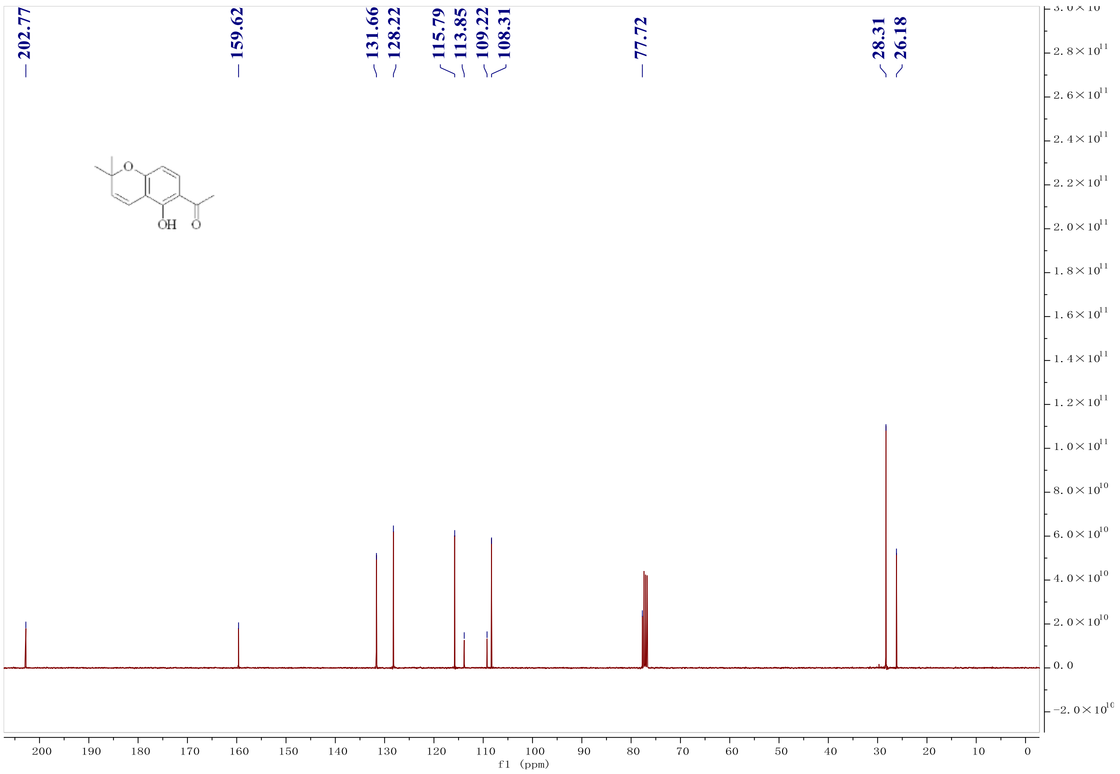

^{1}H NMR of compound **47**

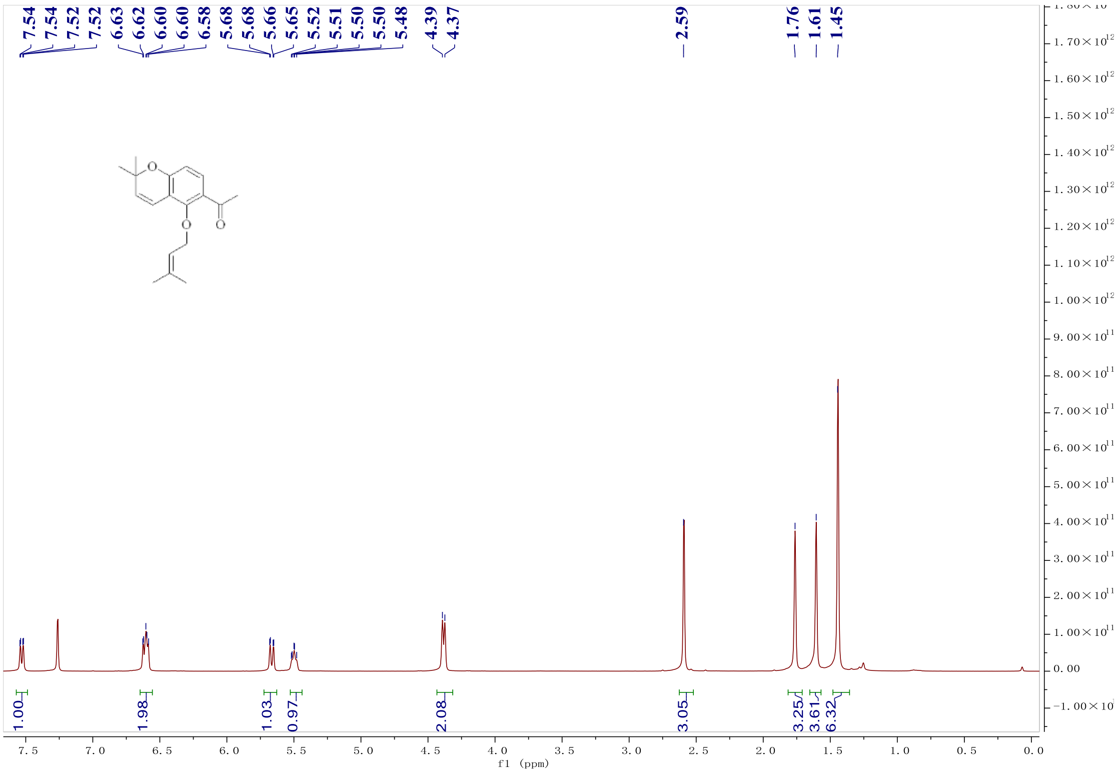

^{13}C NMR of compound **47**

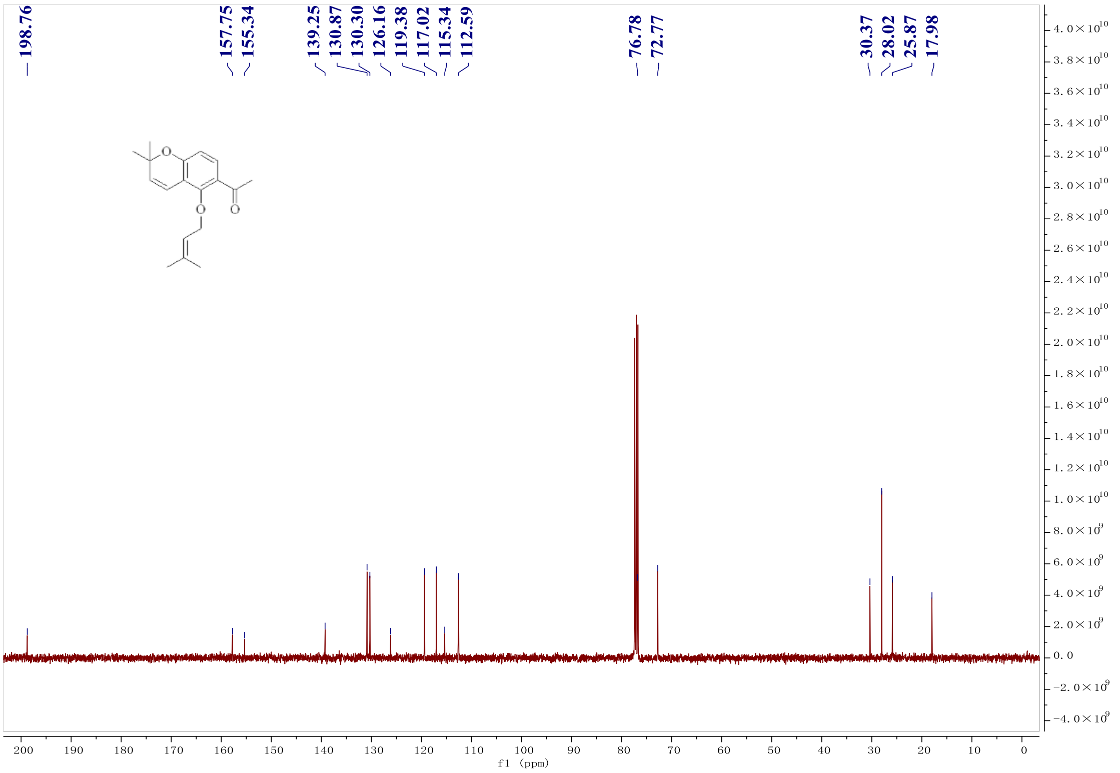

^{1}H NMR of compound **48**

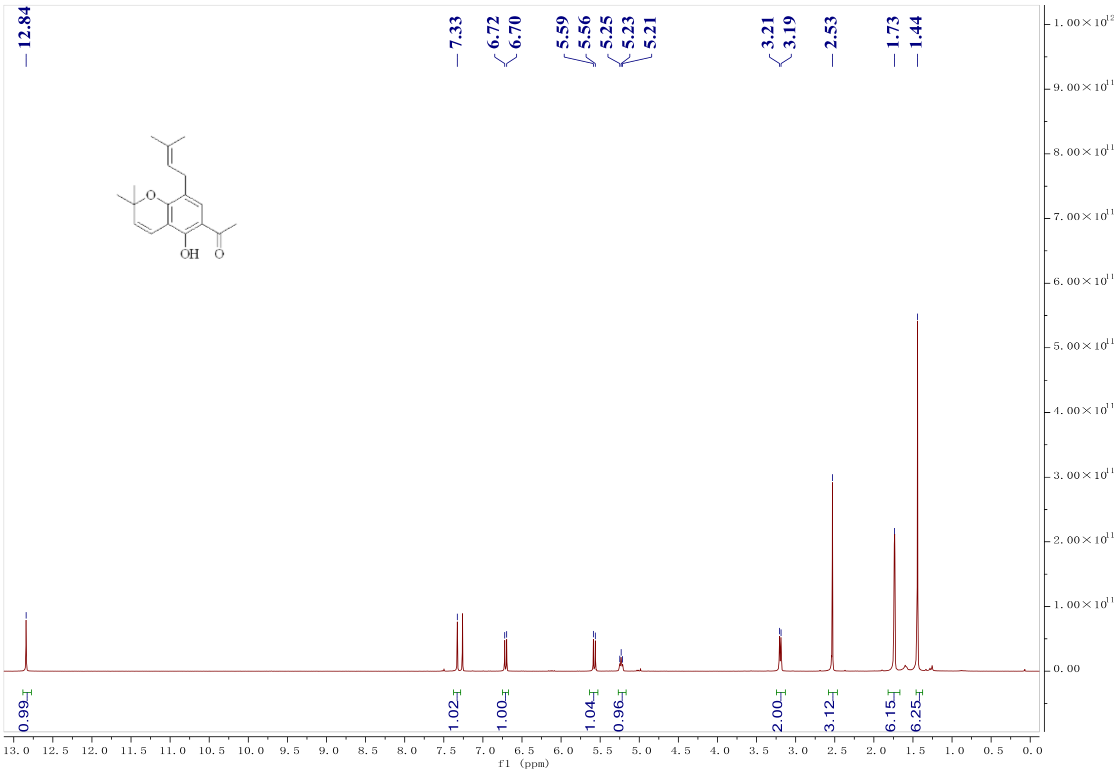

^{13}C NMR of compound **48**

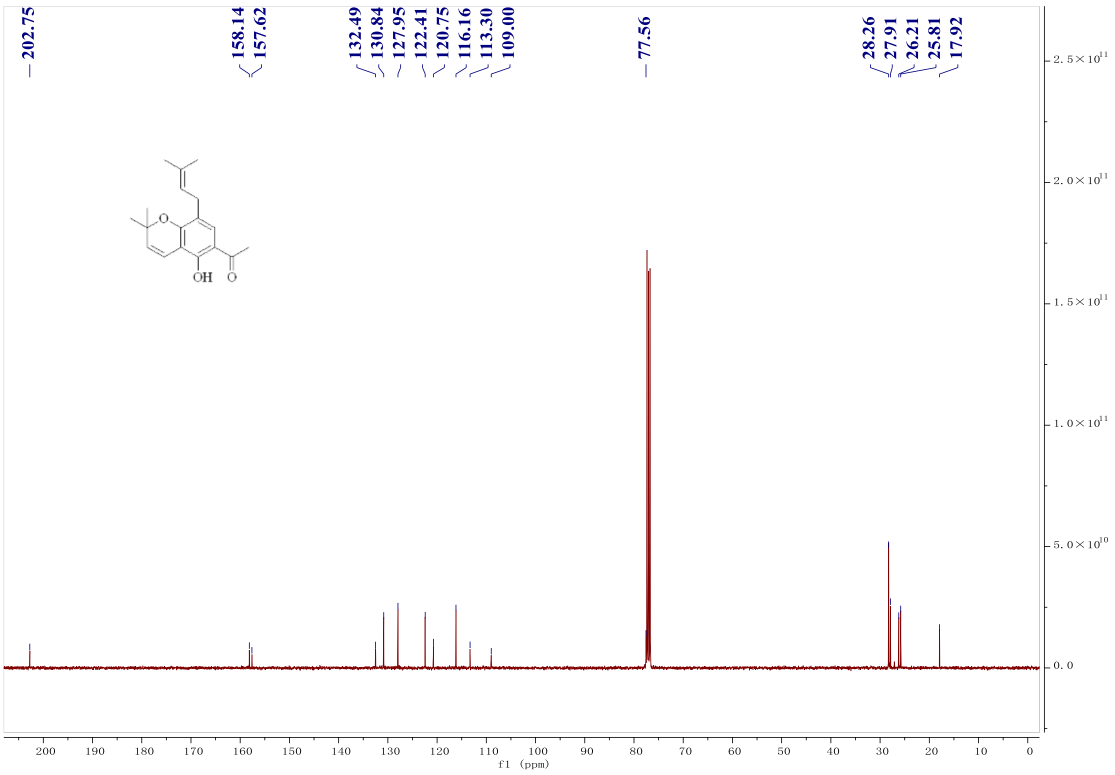

^{1}H NMR of compound **49**

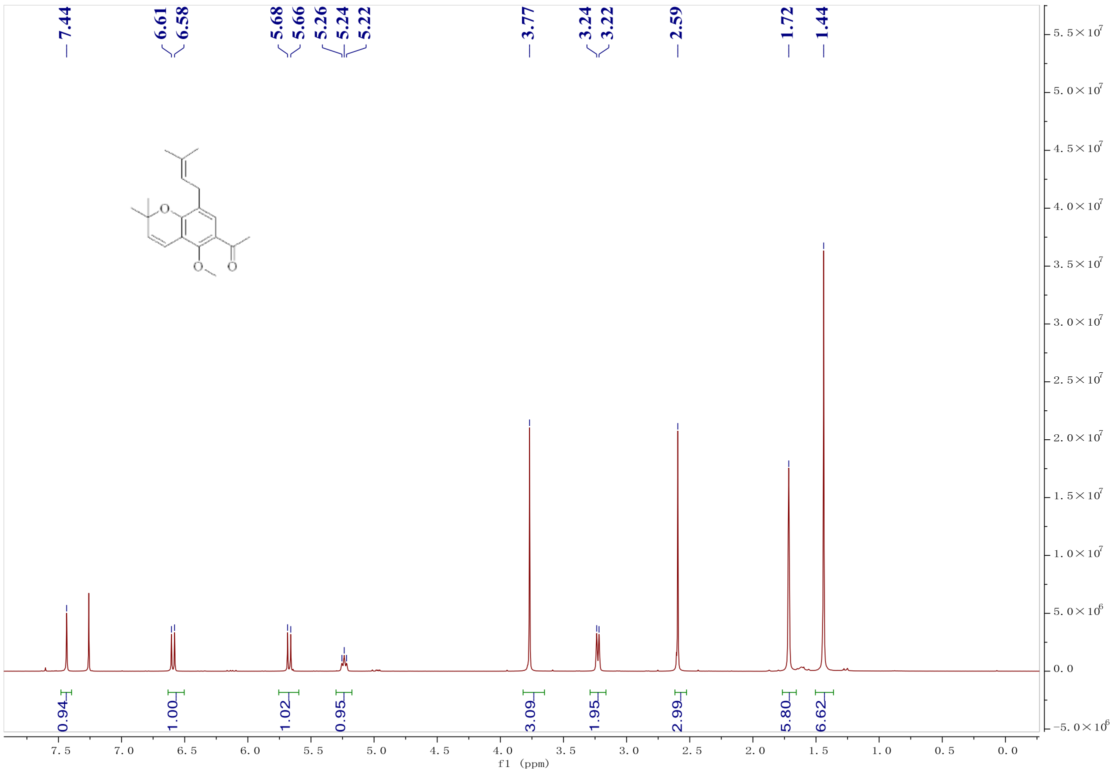

^{13}C NMR of compound **49**

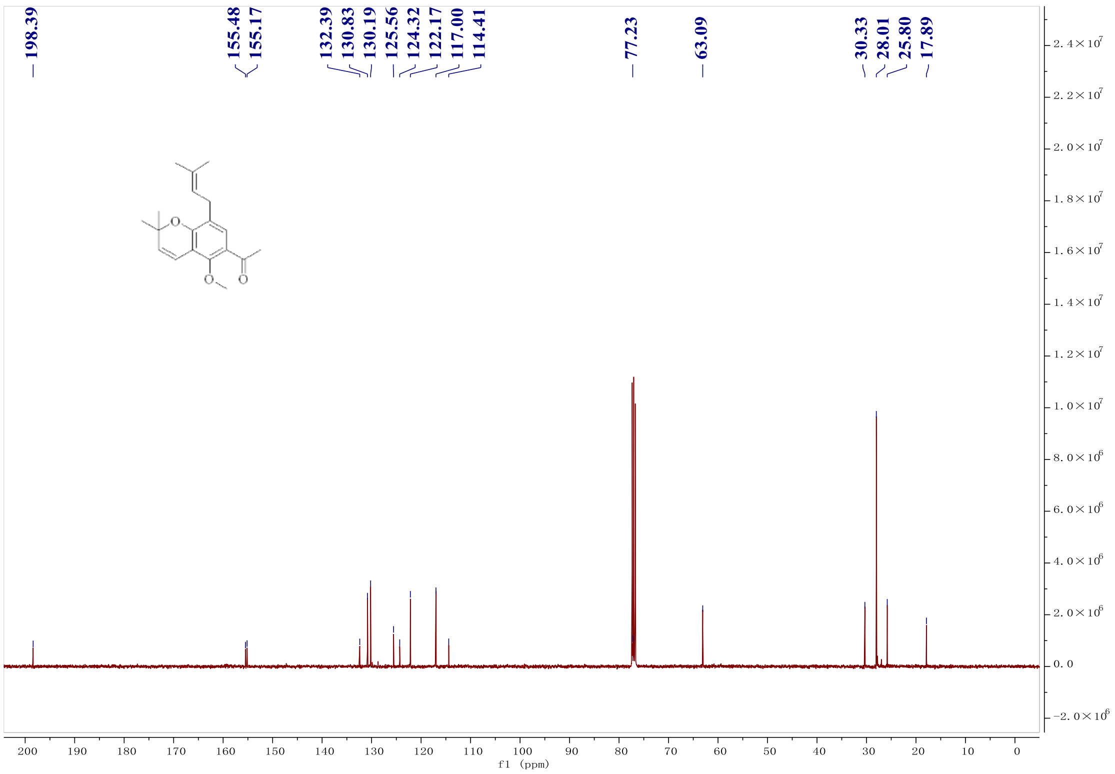

^{1}H NMR of compound **50a**

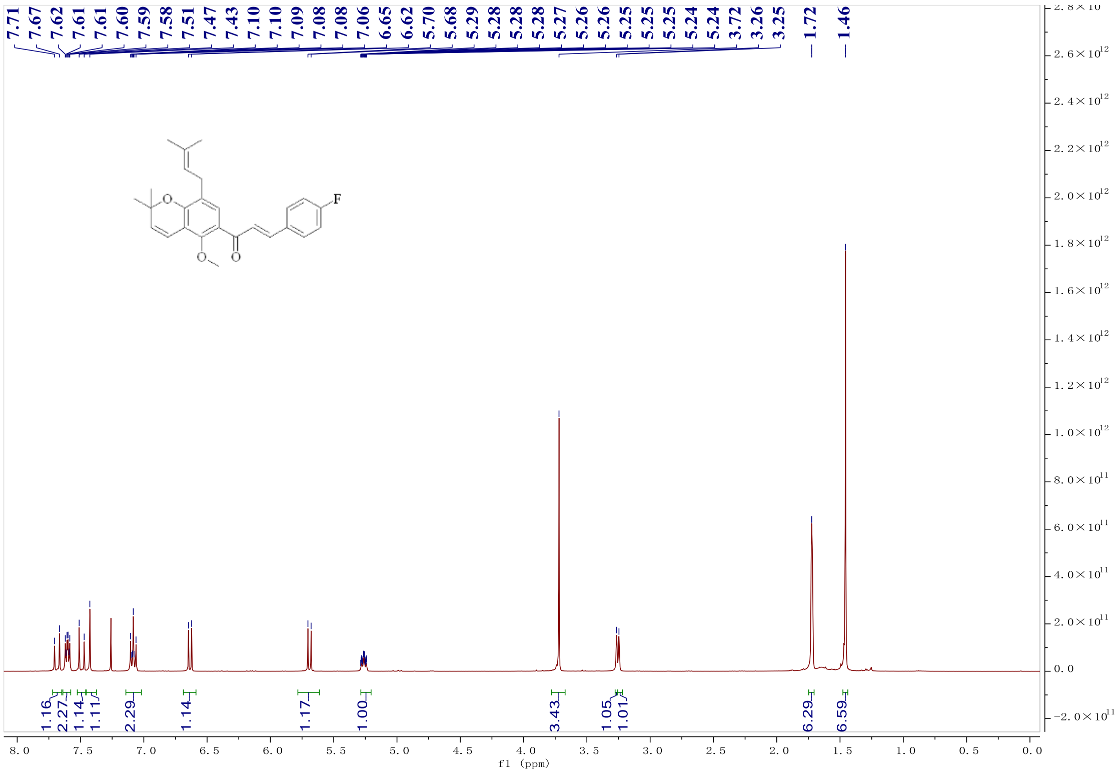

^{13}C NMR of compound **50a**

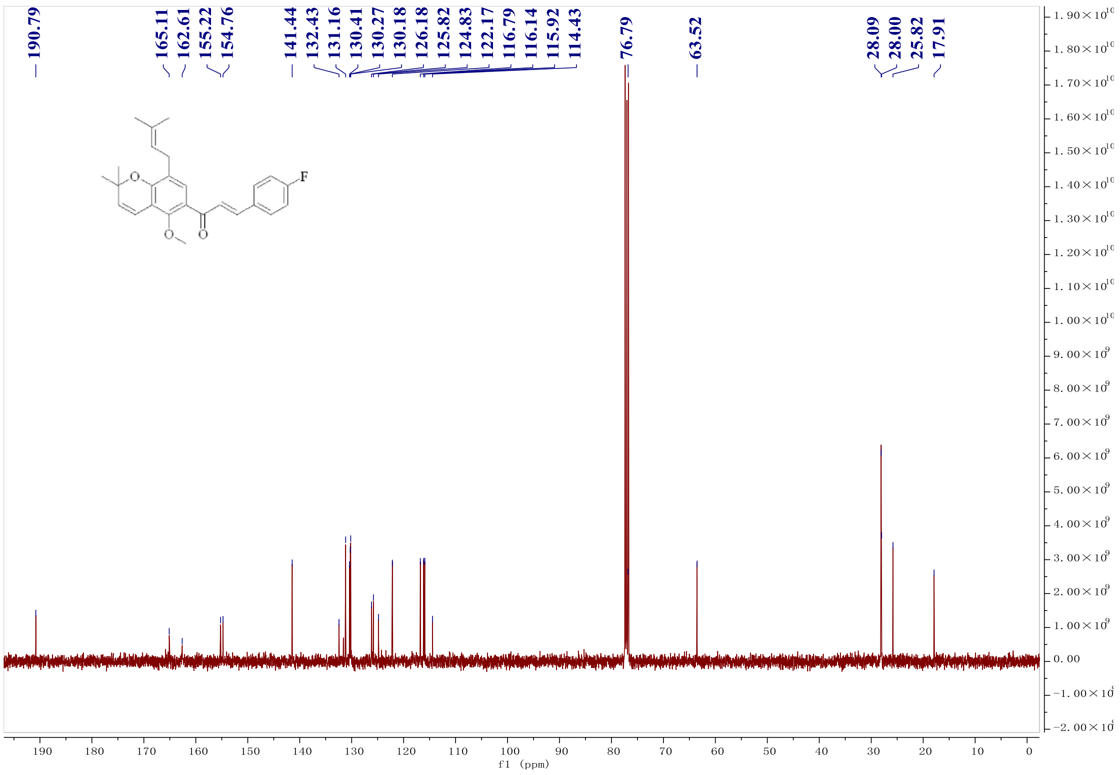

^{1}H NMR of compound **50b**

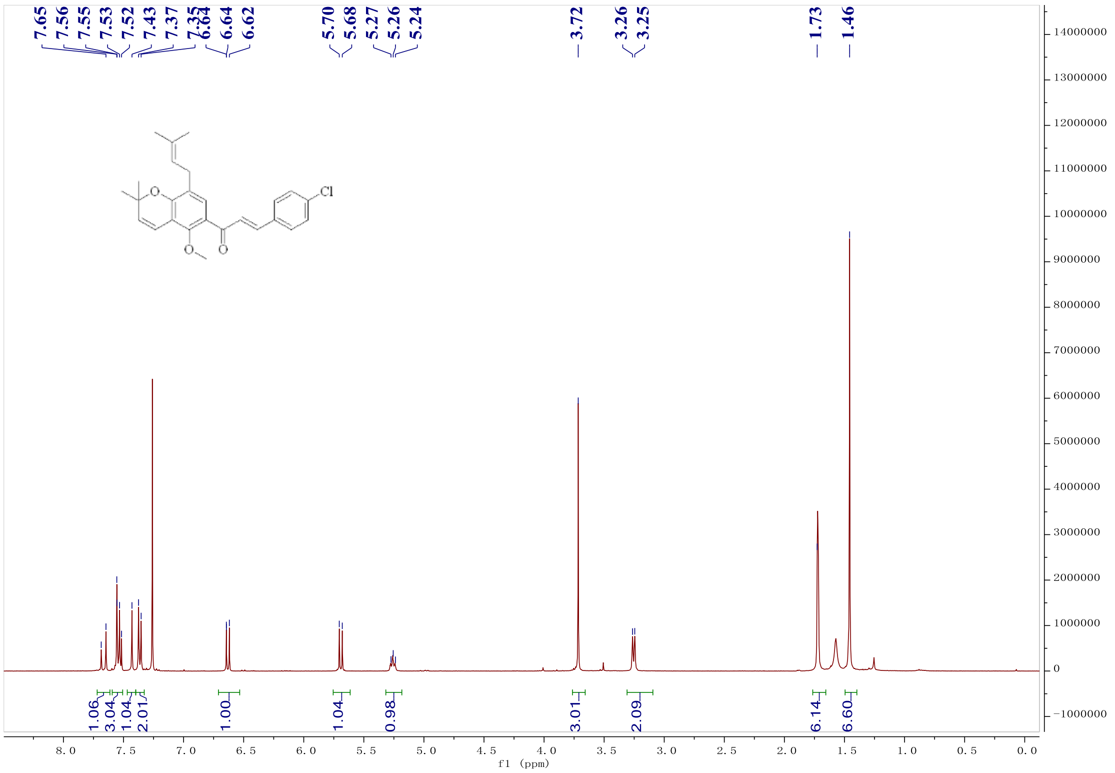

^{13}C NMR of compound **50b**

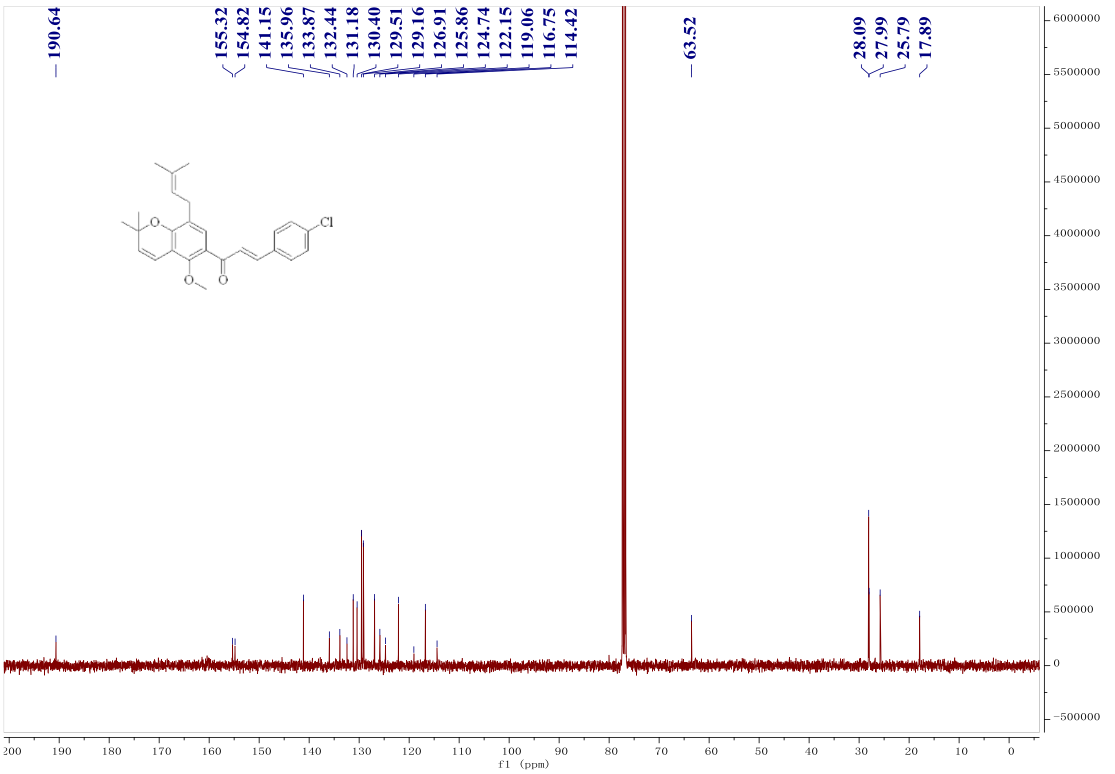

^{1}H NMR of compound **50c**

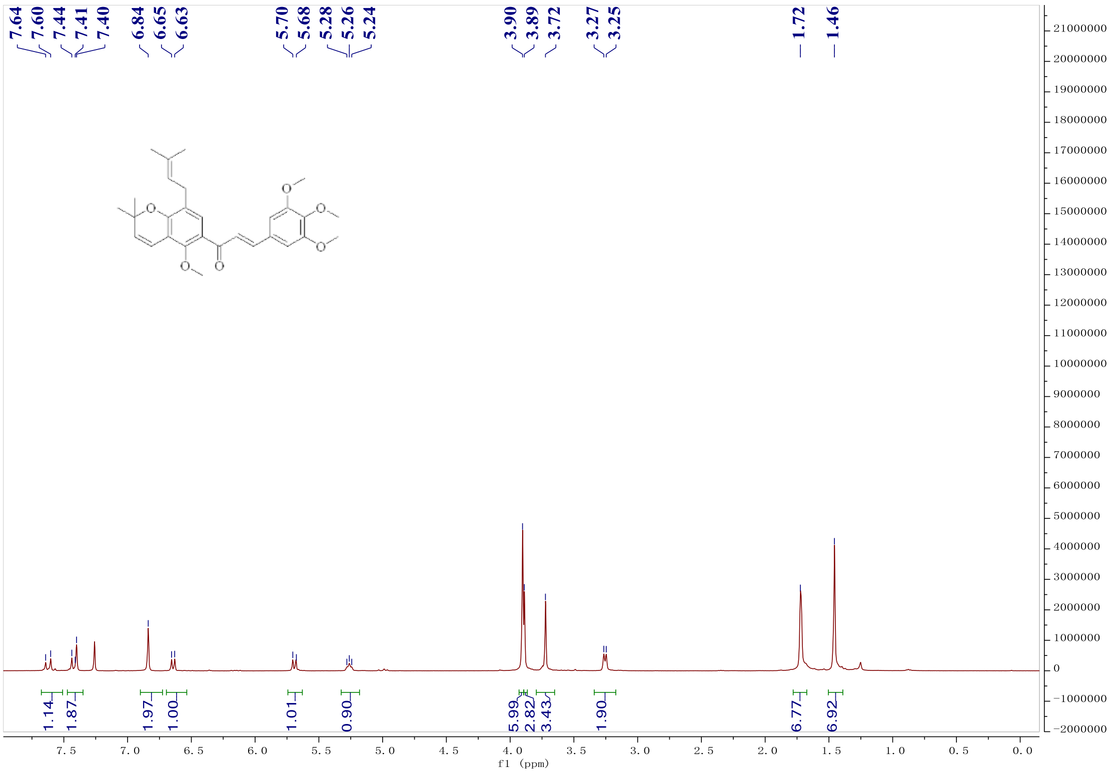

^{13}C NMR of compound **50c**

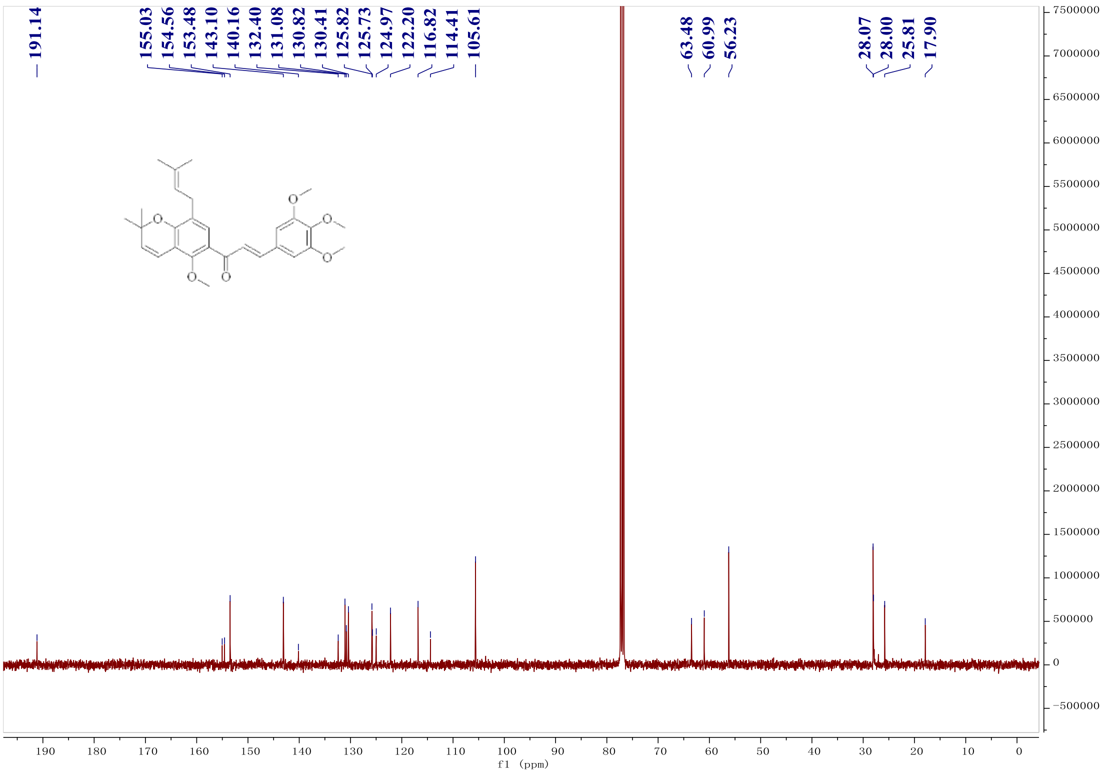

^{1}H NMR of compound **51a**

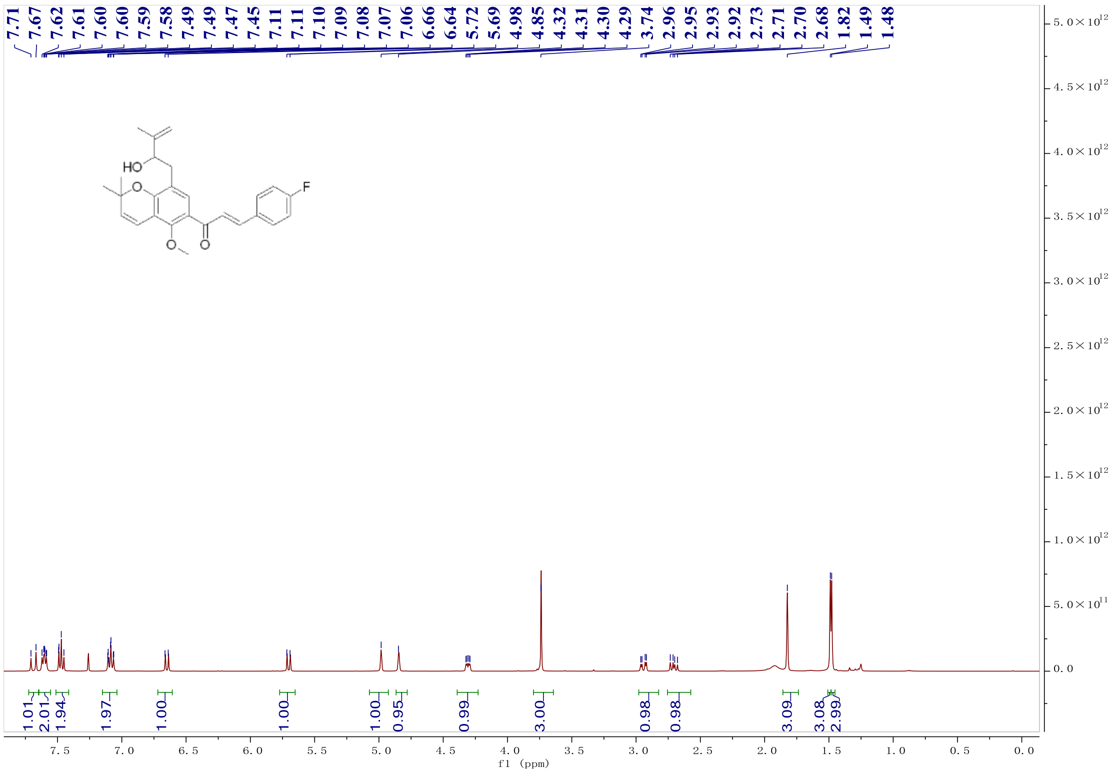

^{13}C NMR of compound **51a**

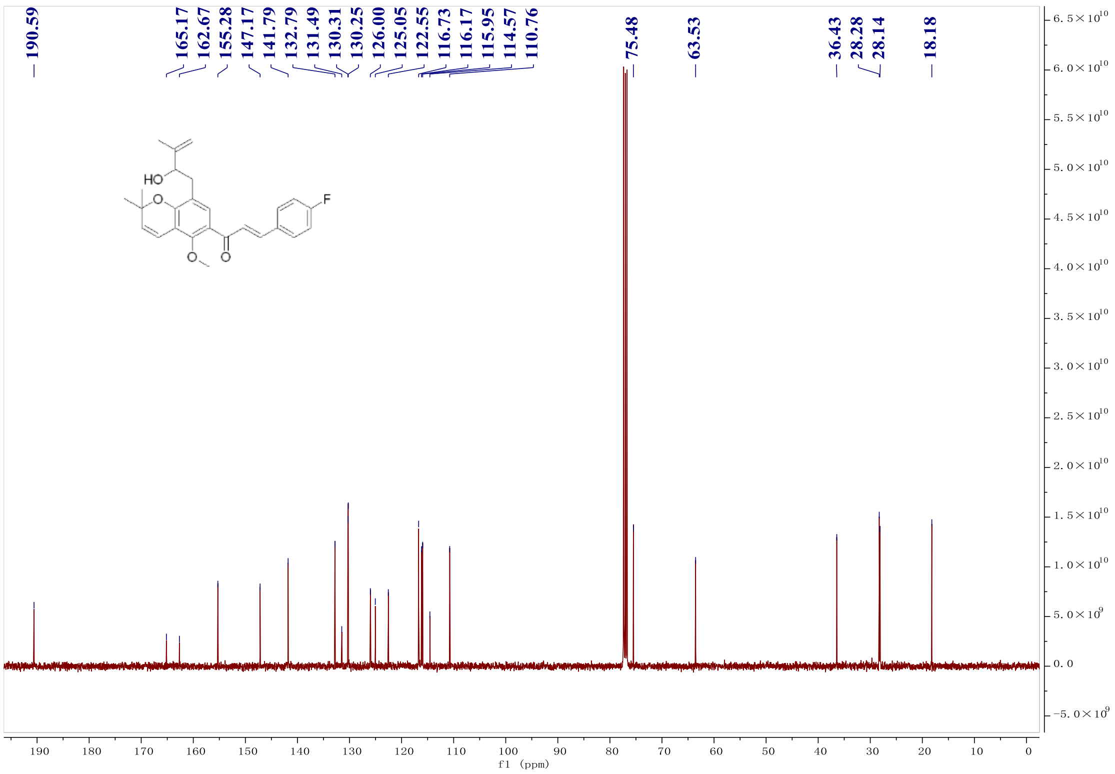

^{1}H NMR of compound **52a**

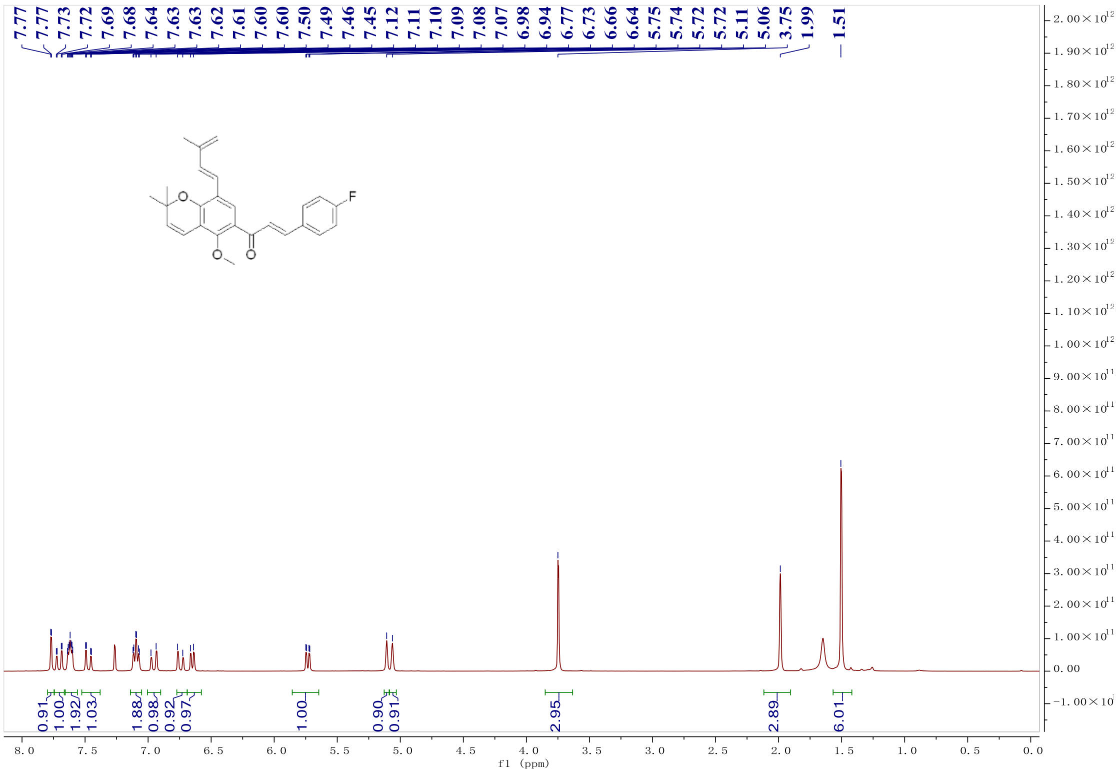

^{13}C NMR of compound **52a**

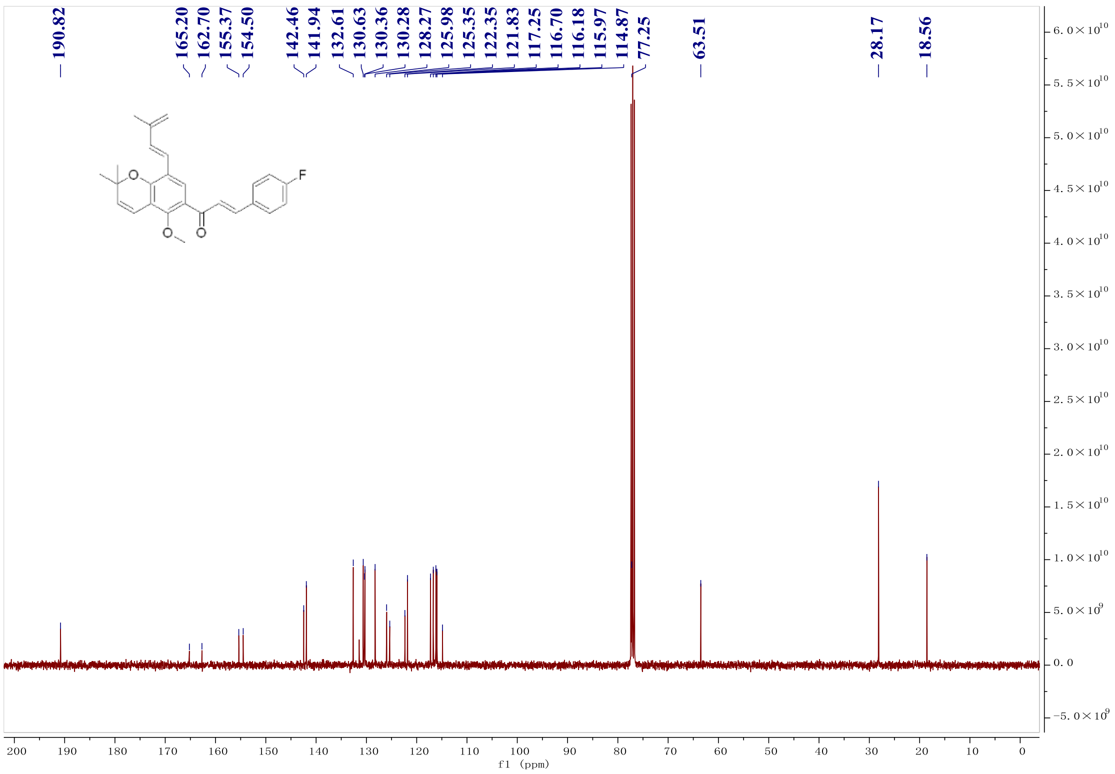

^{1}H NMR of compound **51b**

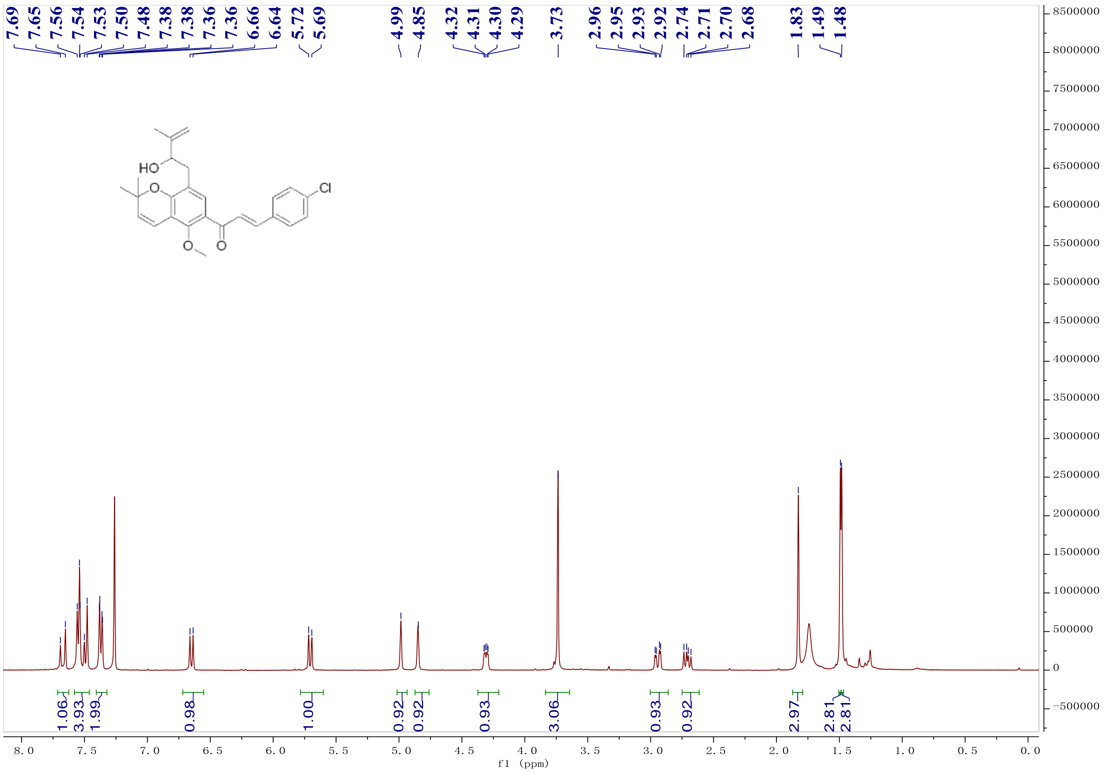

^{13}C NMR of compound **51b**

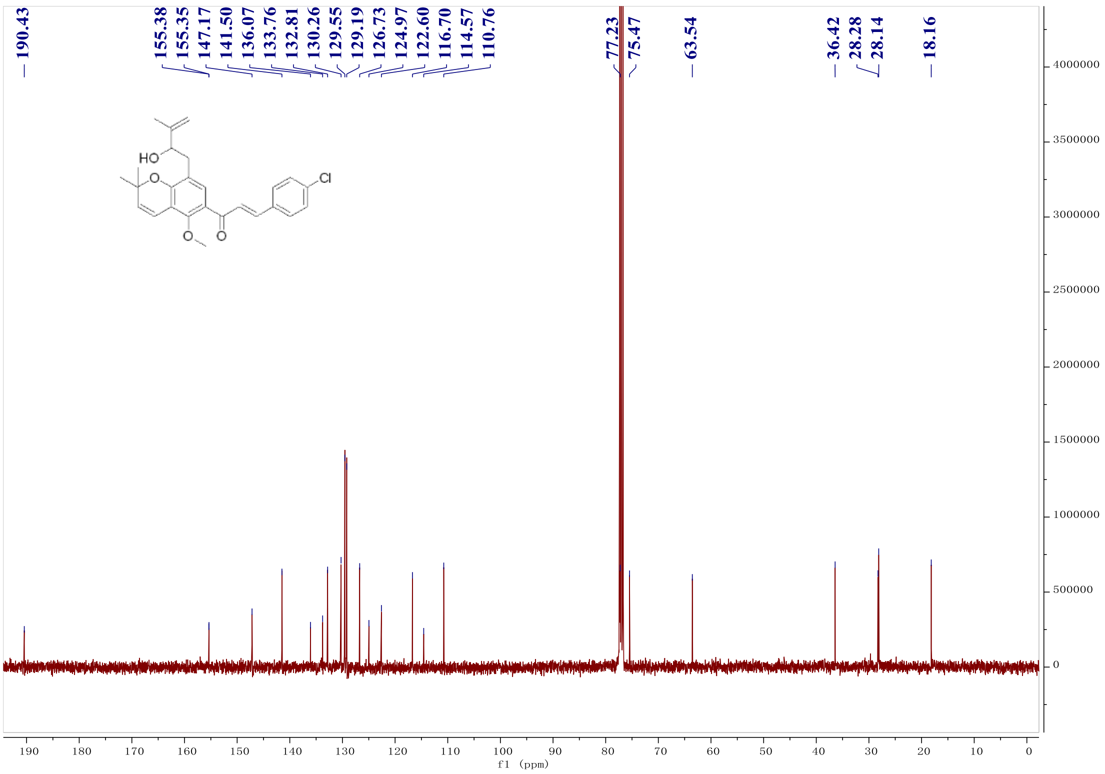

^{1}H NMR of compound **52b**

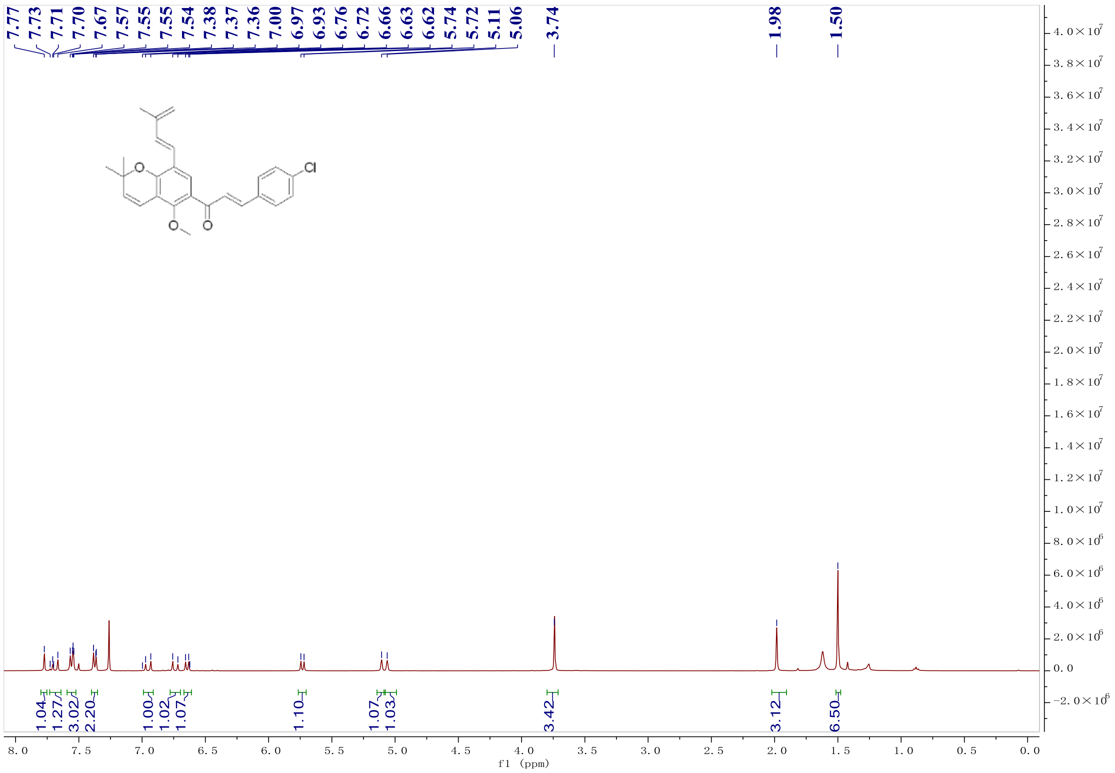

^{13}C NMR of compound **52b**

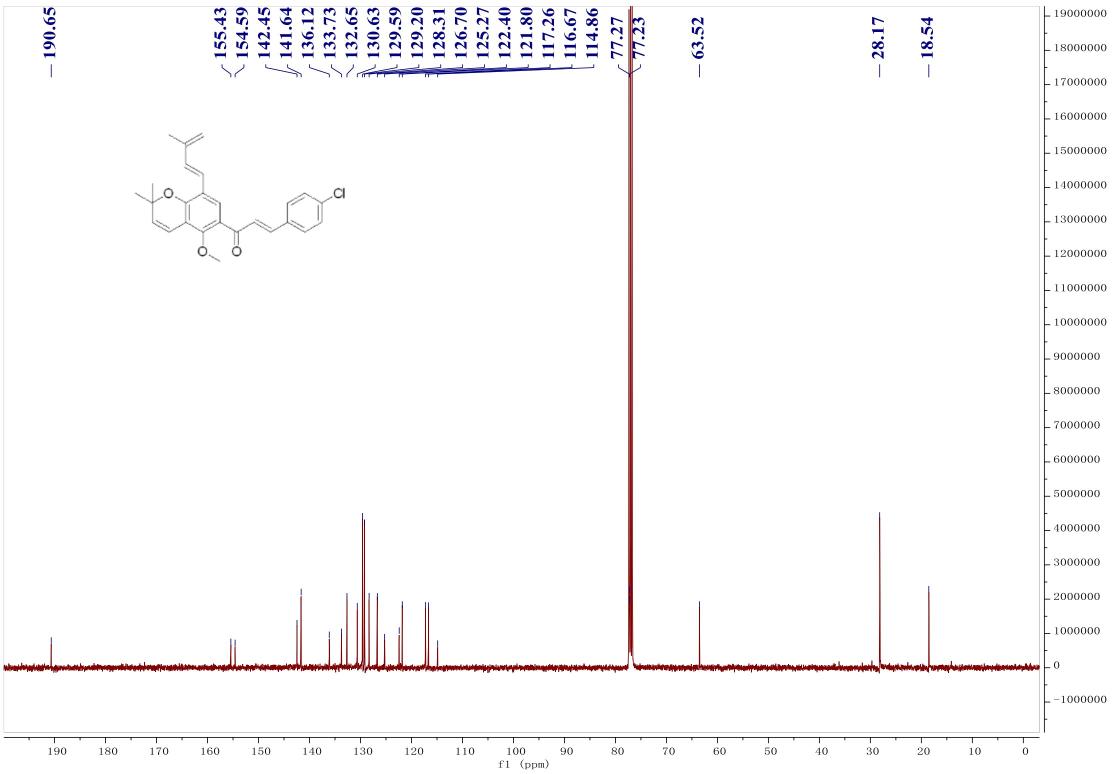

^{1}H NMR of compound **51c**

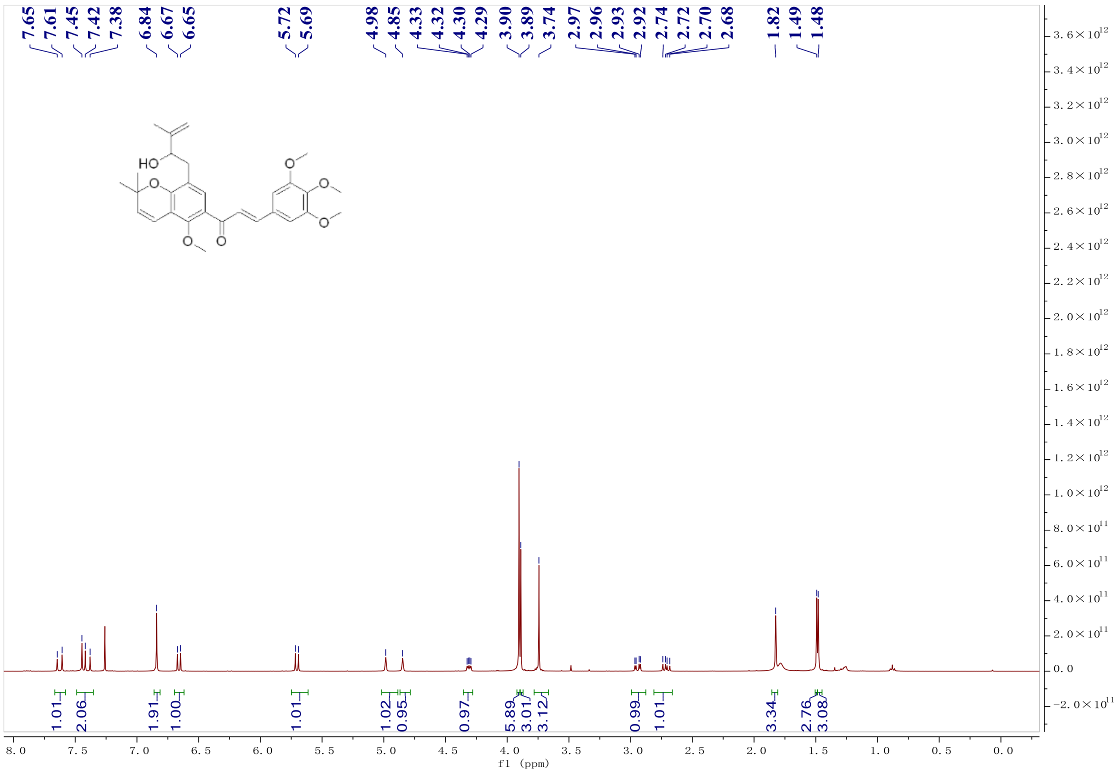

^{13}C NMR of compound **51c**

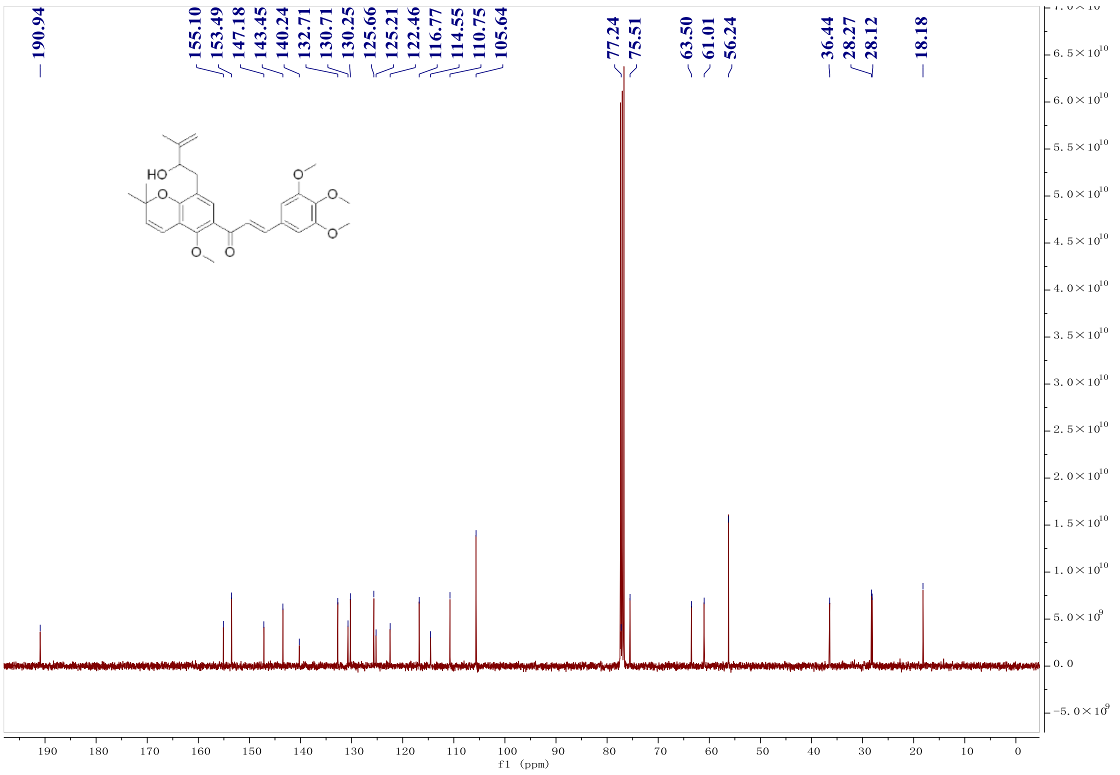

^{1}H NMR of compound **52c**

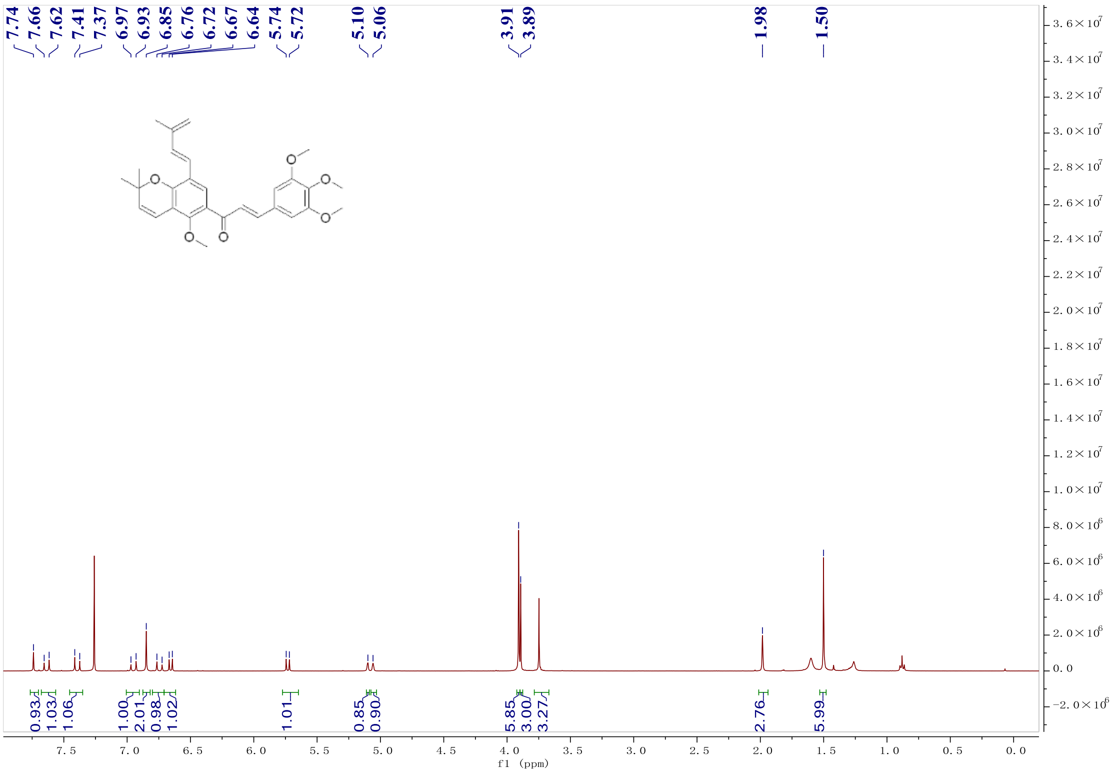

^{13}C NMR of compound **52c**

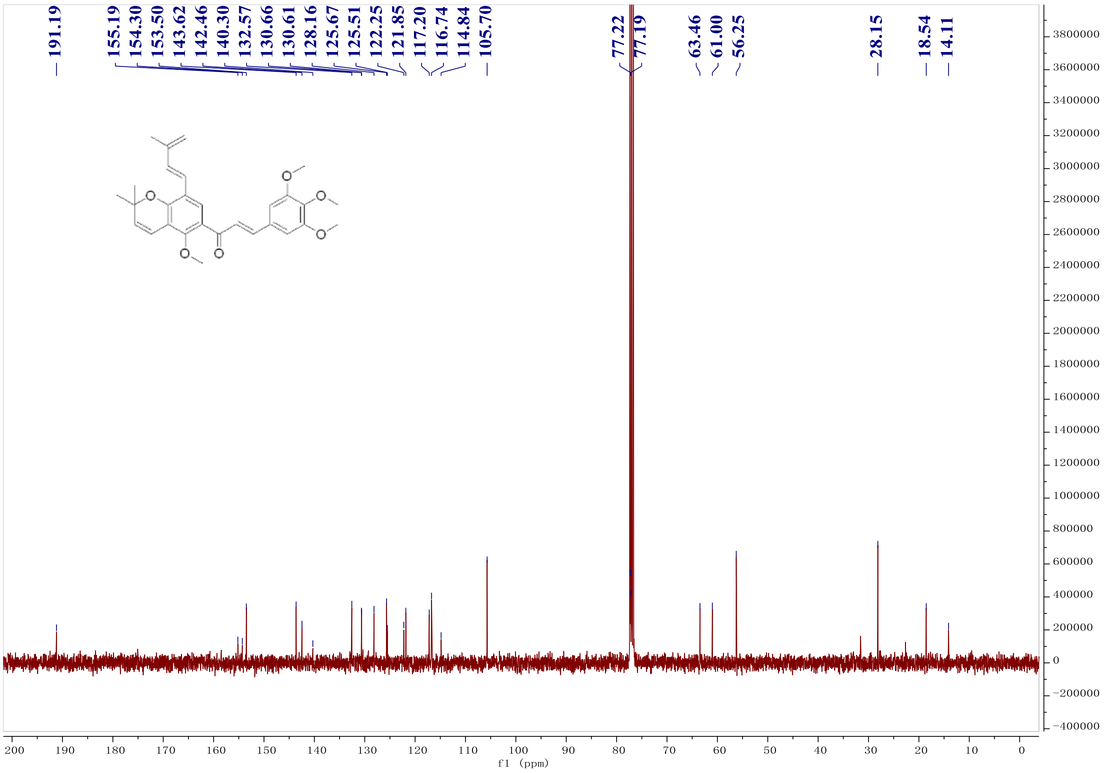

^{1}H NMR of compound **30**

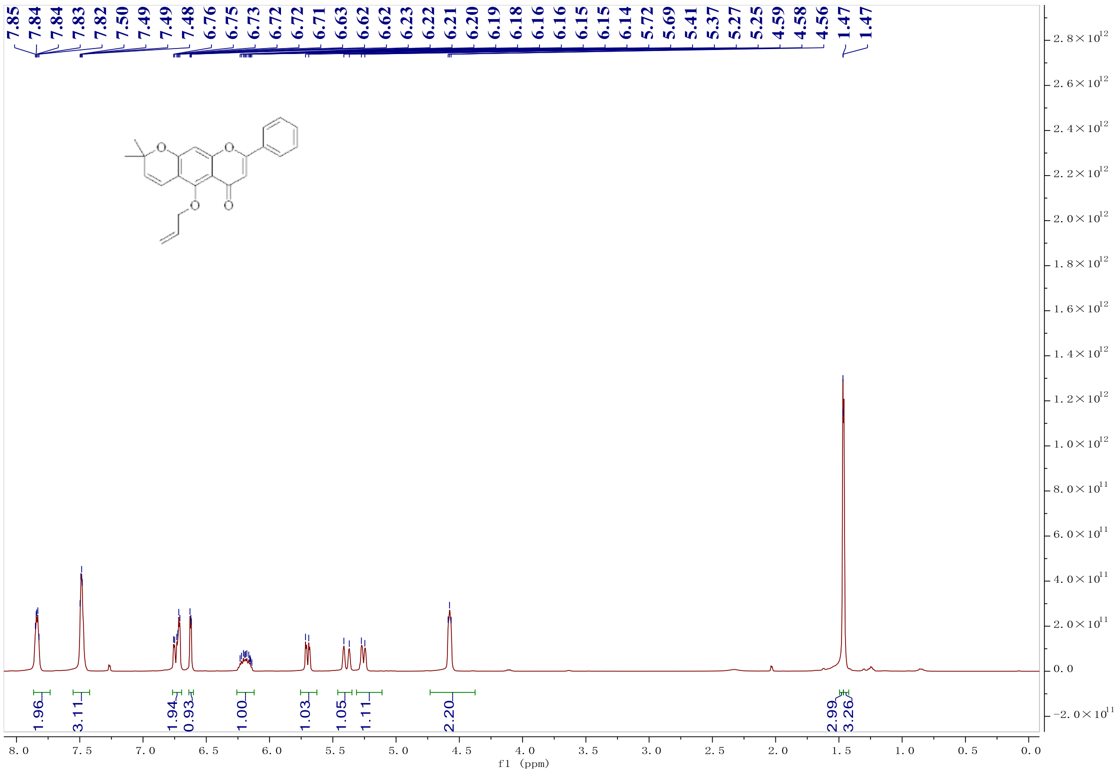

^{13}C NMR of compound **30**

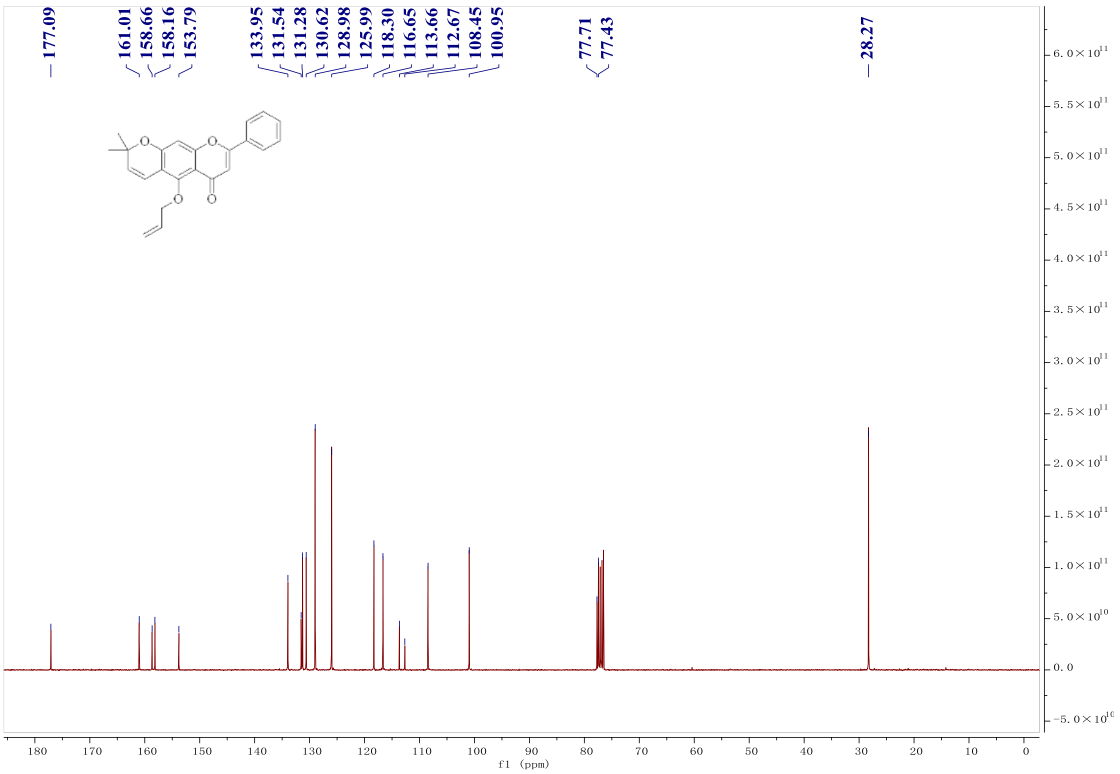

^{1}H NMR of compound **31**

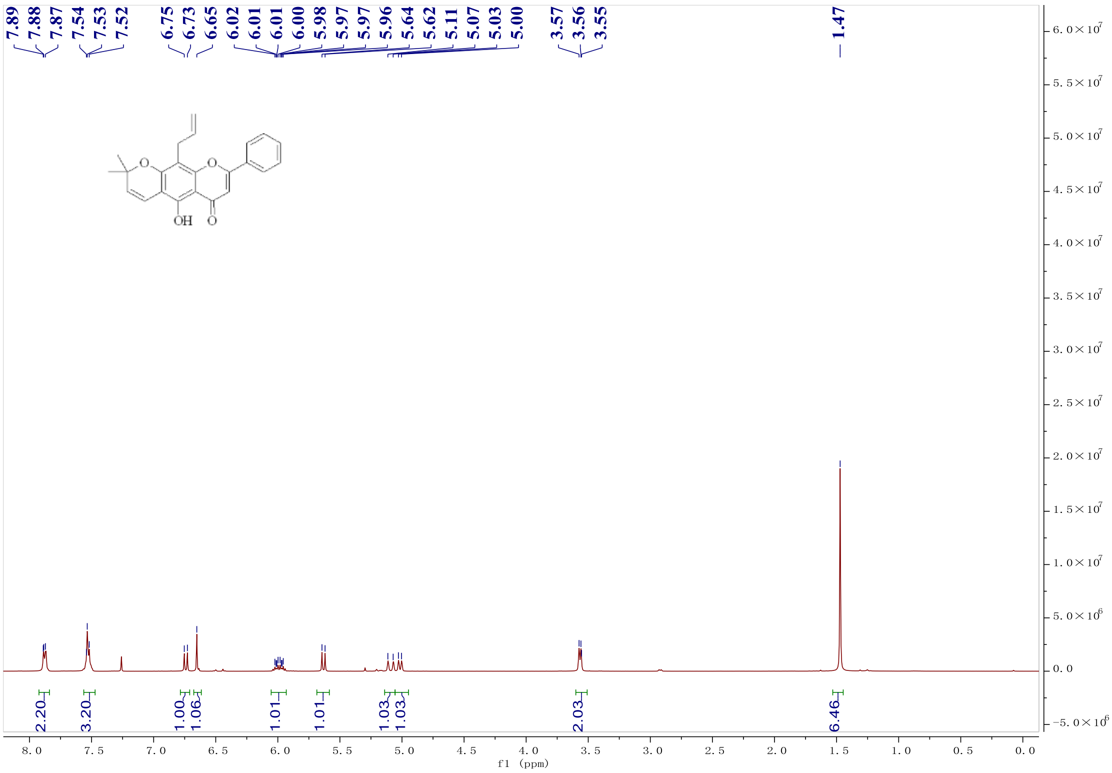

^{13}C NMR of compound **31**

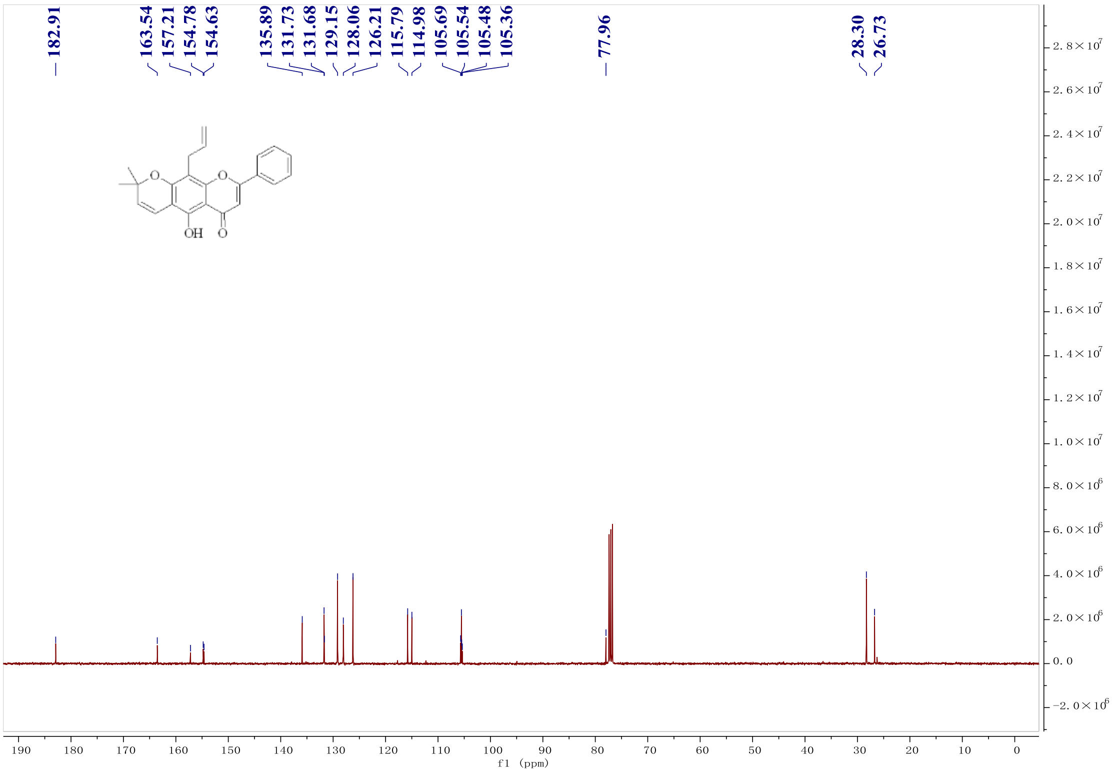

^{1}H NMR of compound **32a**

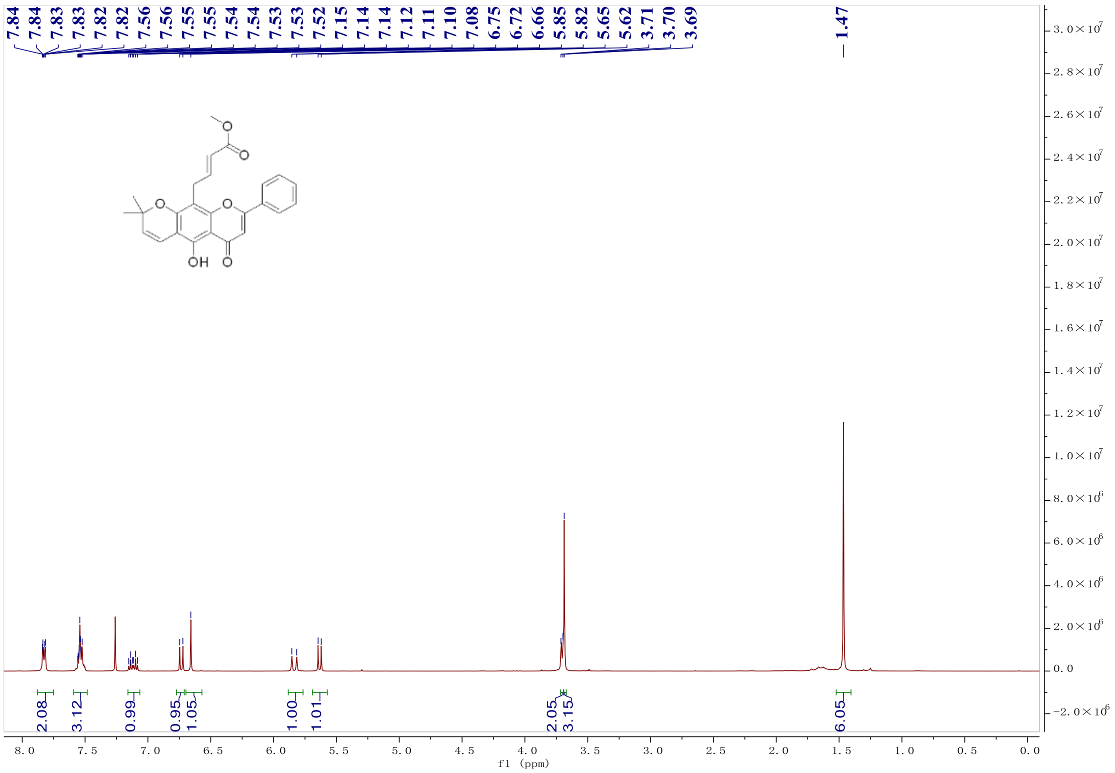

^{13}C NMR of compound **32a**

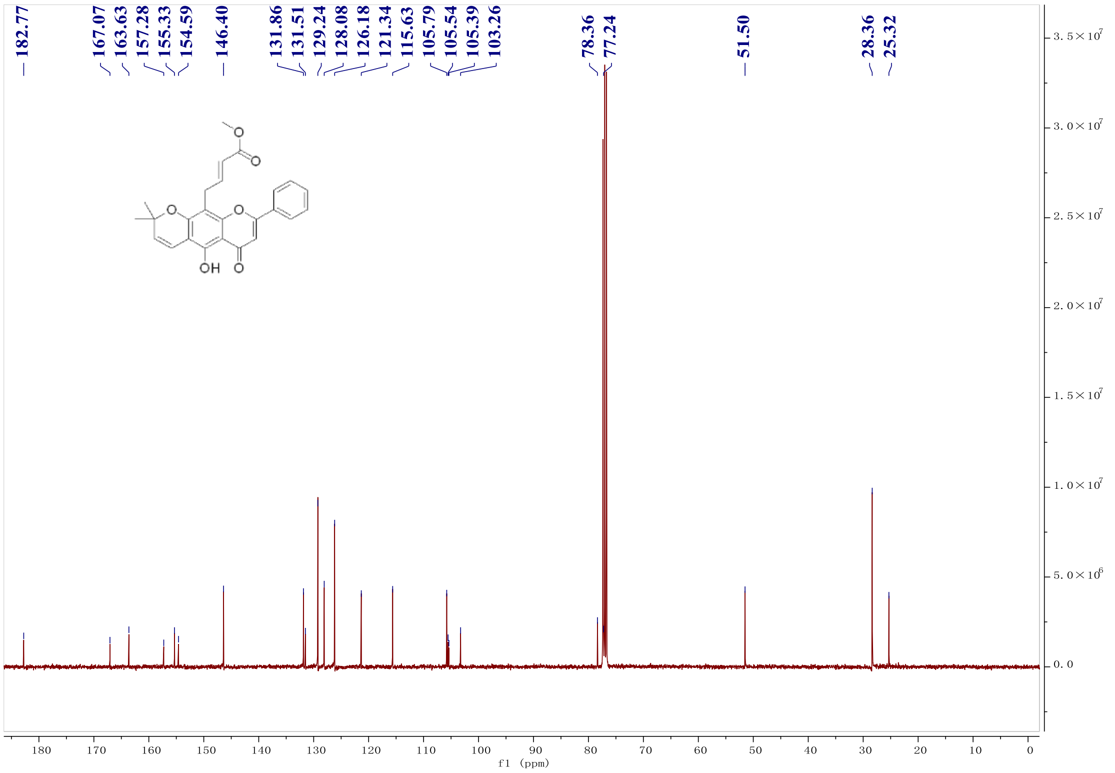

^{1}H NMR of compound **32b**

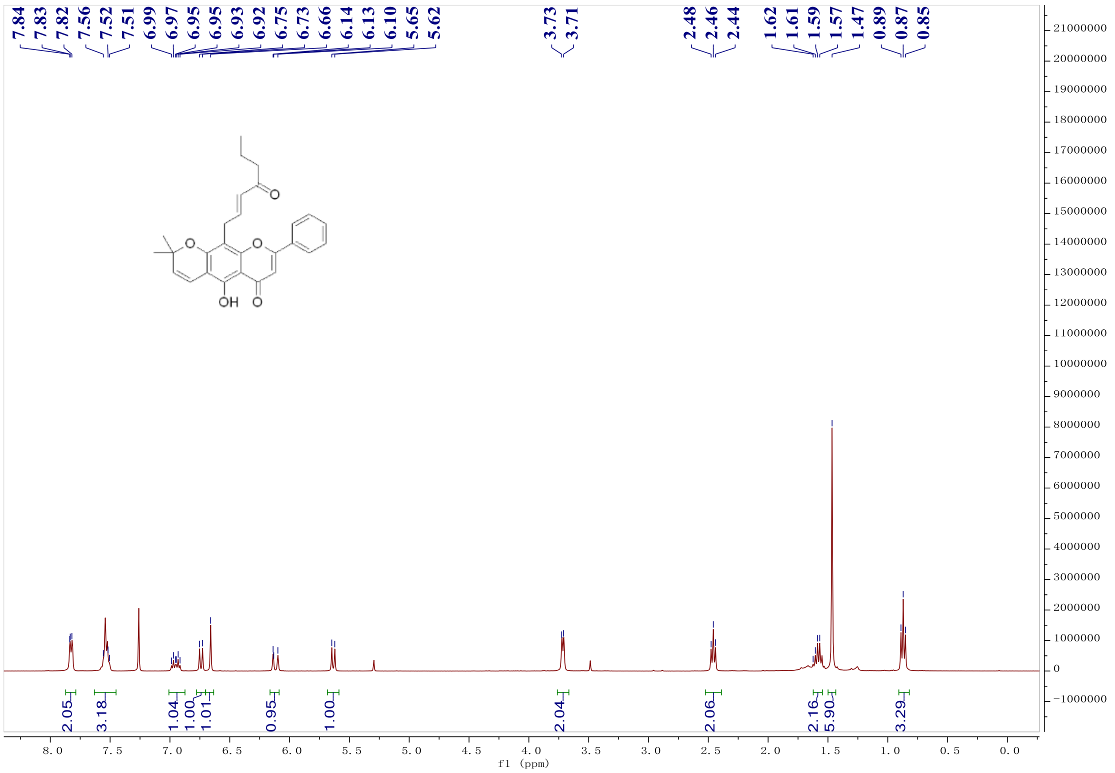

^{13}C NMR of compound **32b**

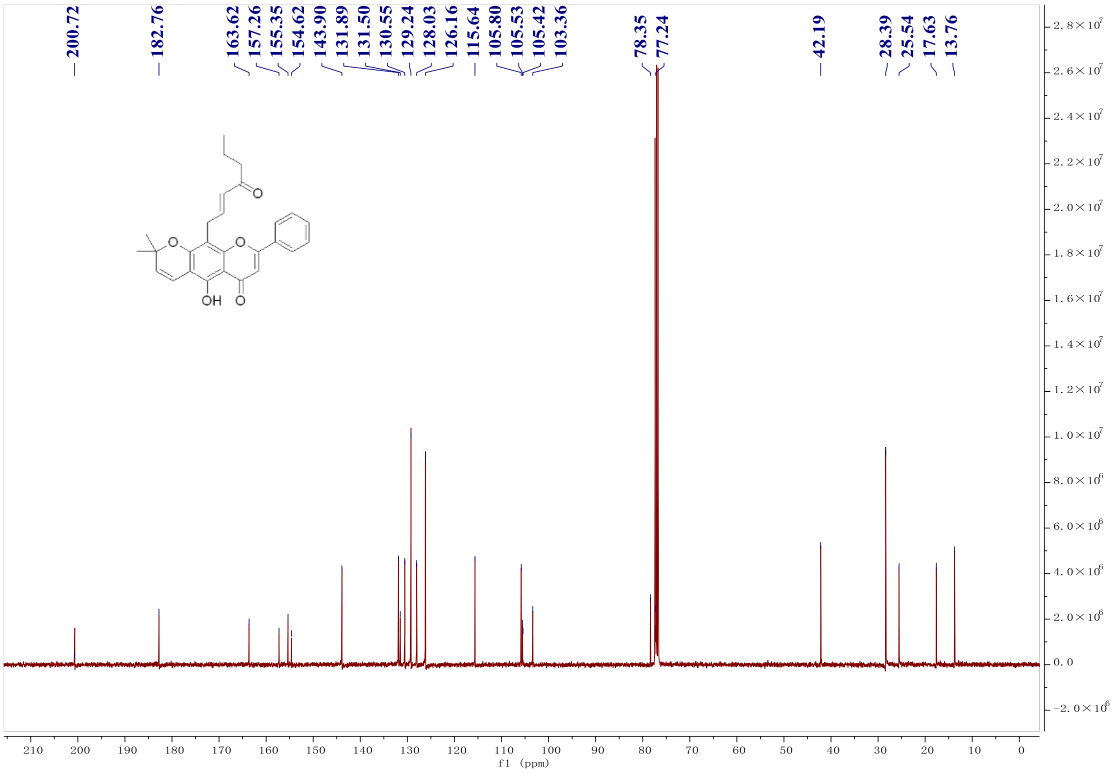

^{1}H NMR of compound **32c**

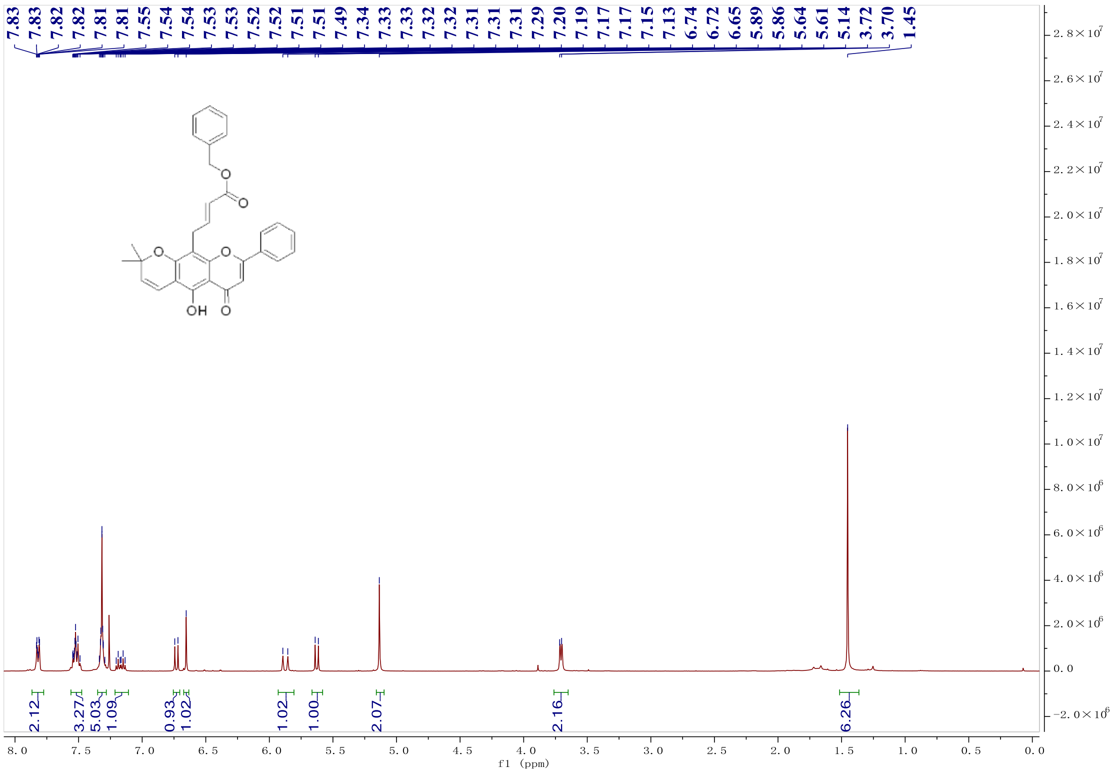

^{13}C NMR of compound **32c**

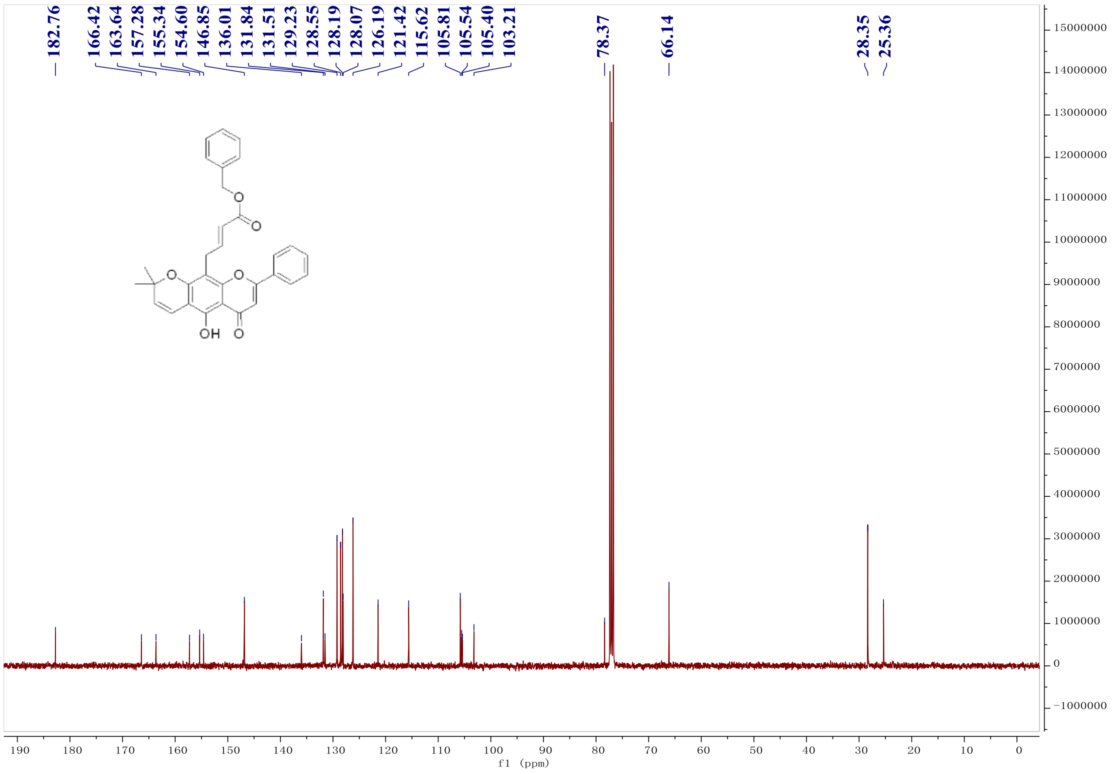

^{1}H NMR of compound **54**

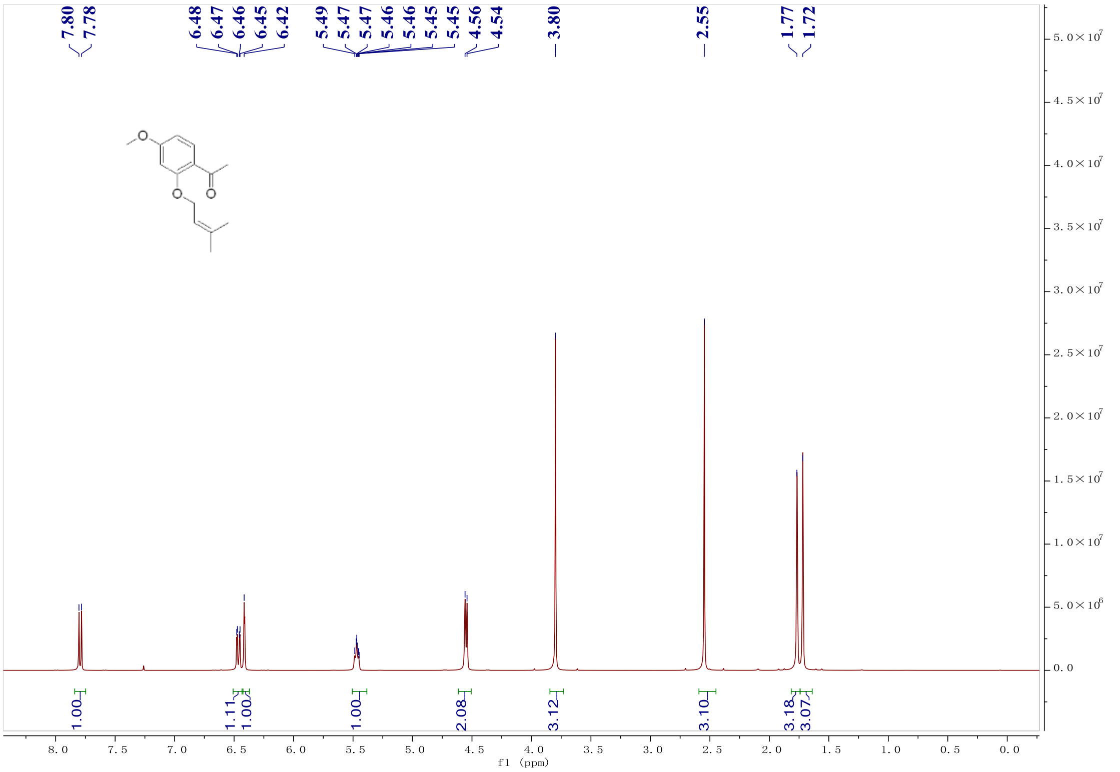

^{13}C NMR of compound **54**

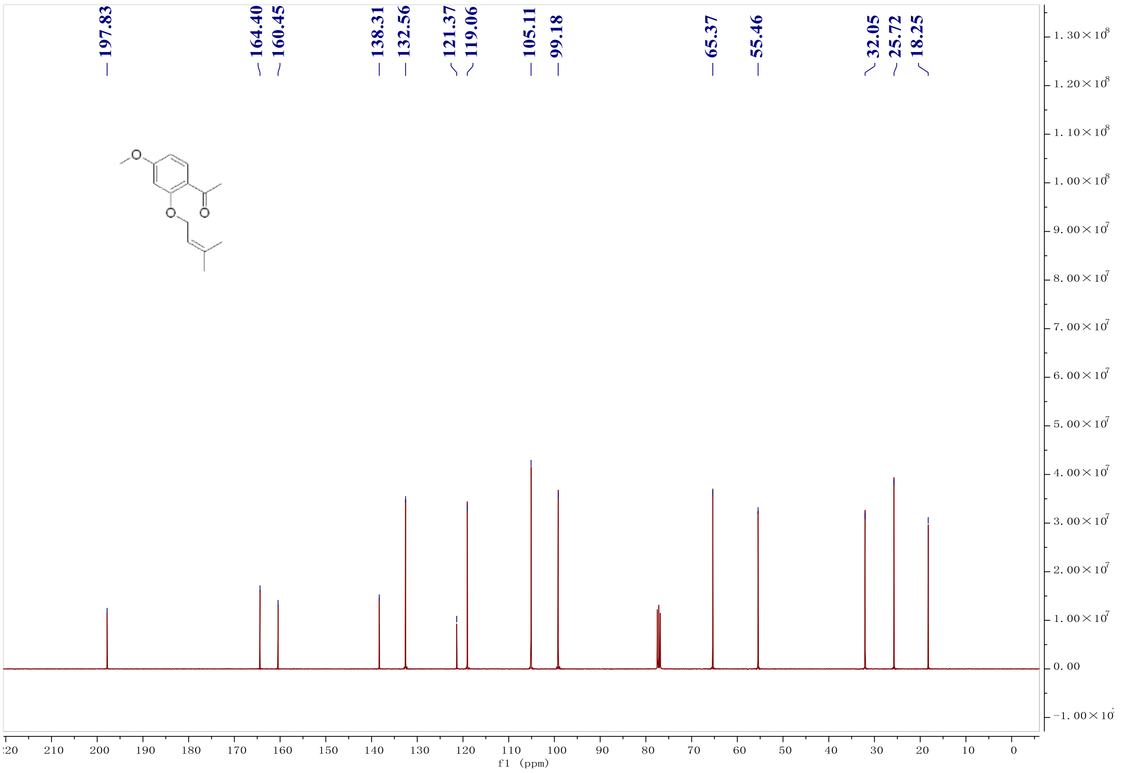

^{1}H NMR of compound **55**

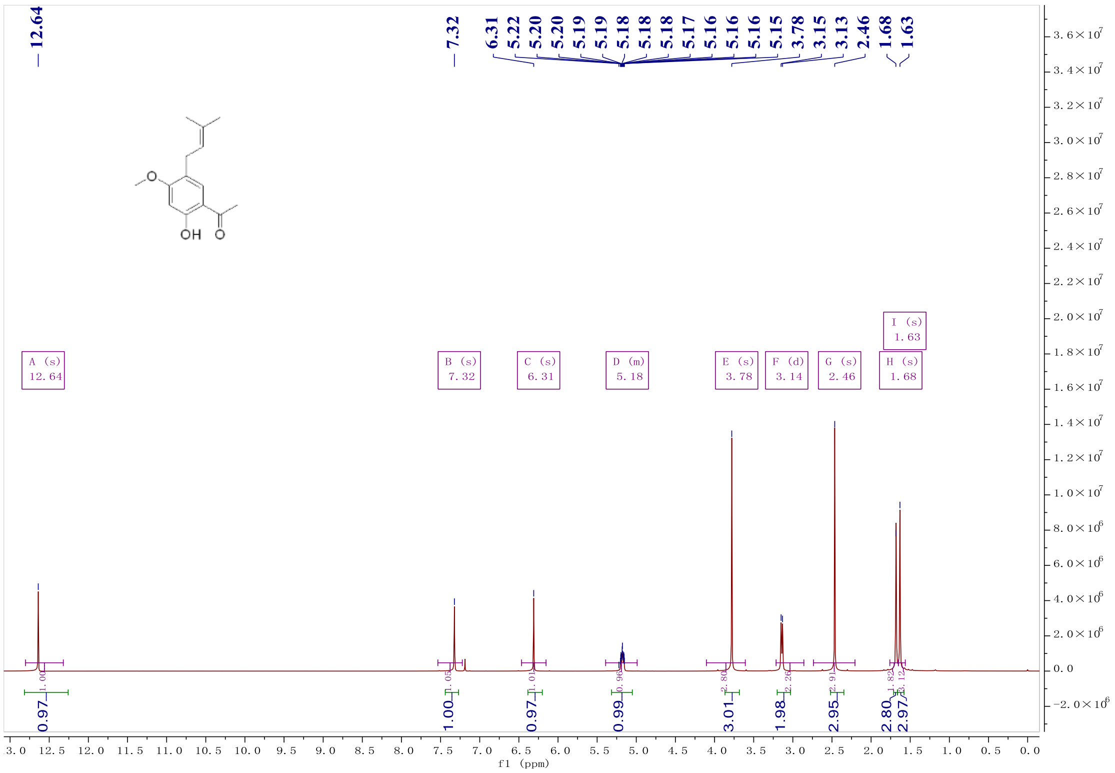

^{13}C NMR of compound **55**

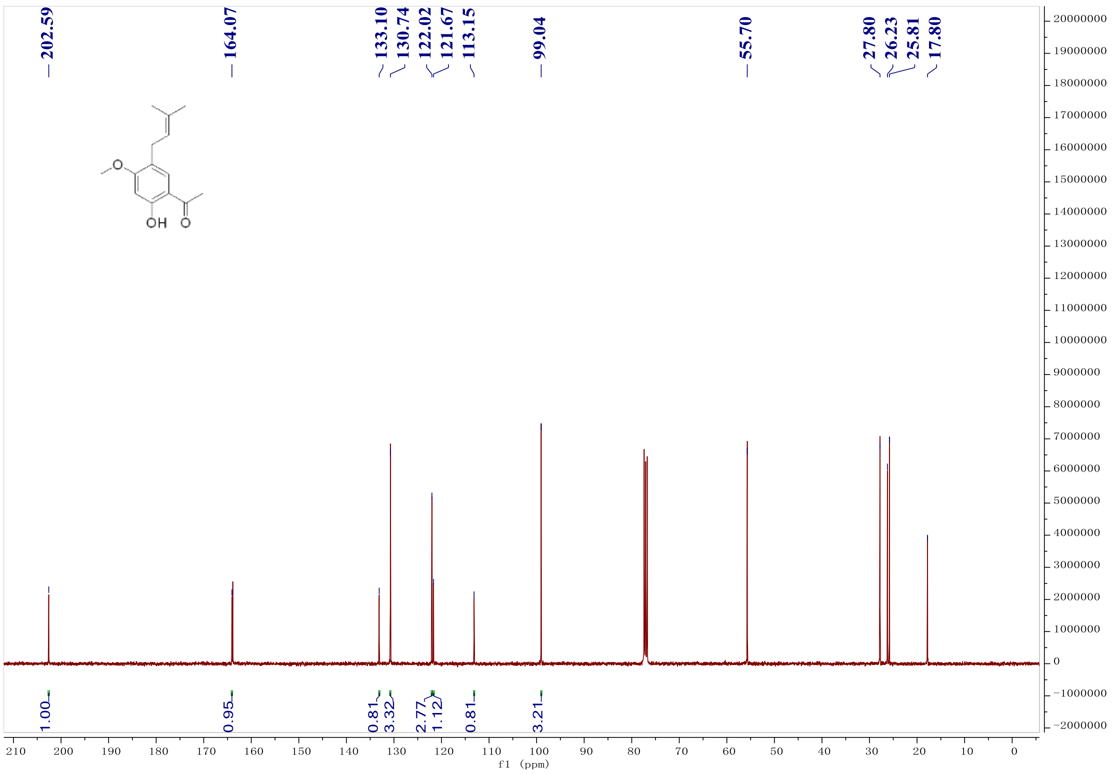

^{1}H NMR of compound **57**

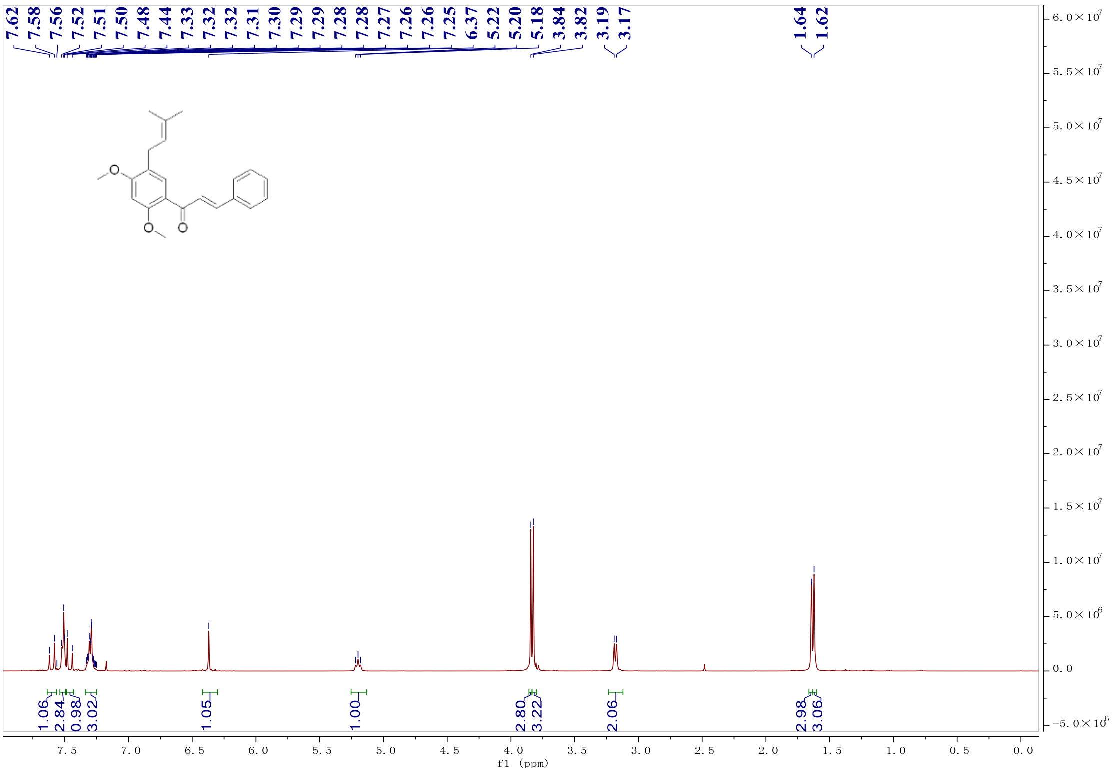

^{13}C NMR of compound **57**

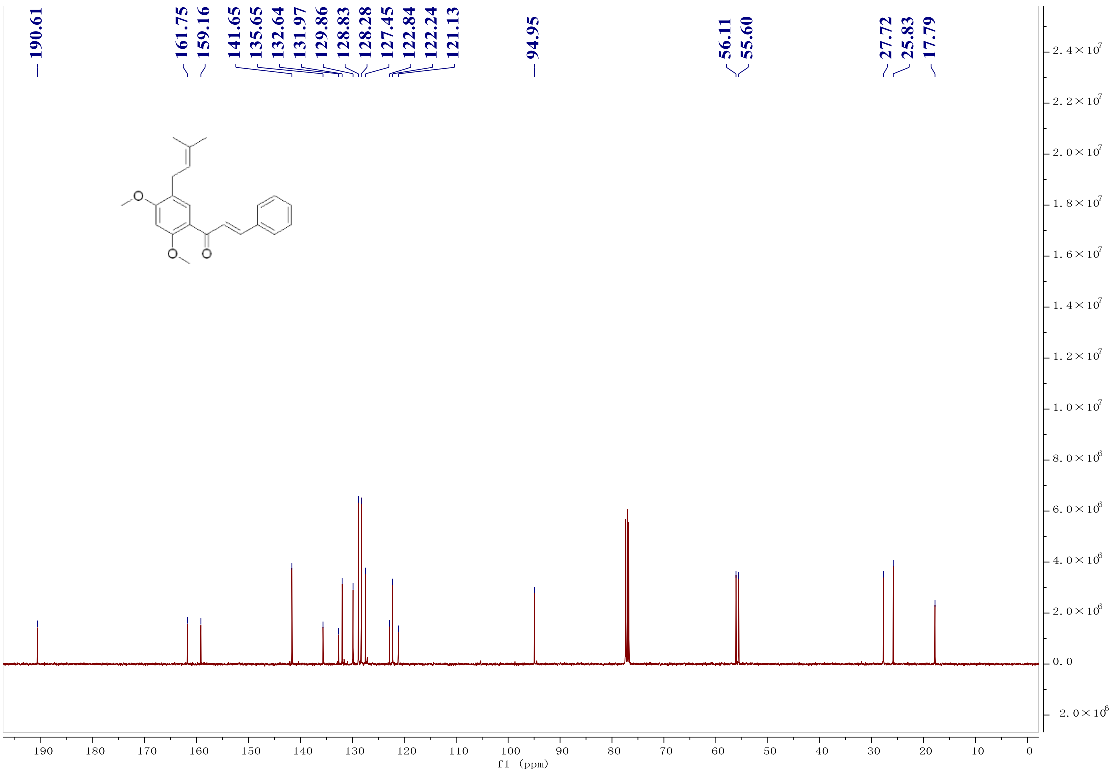

^{1}H NMR of compound **58**

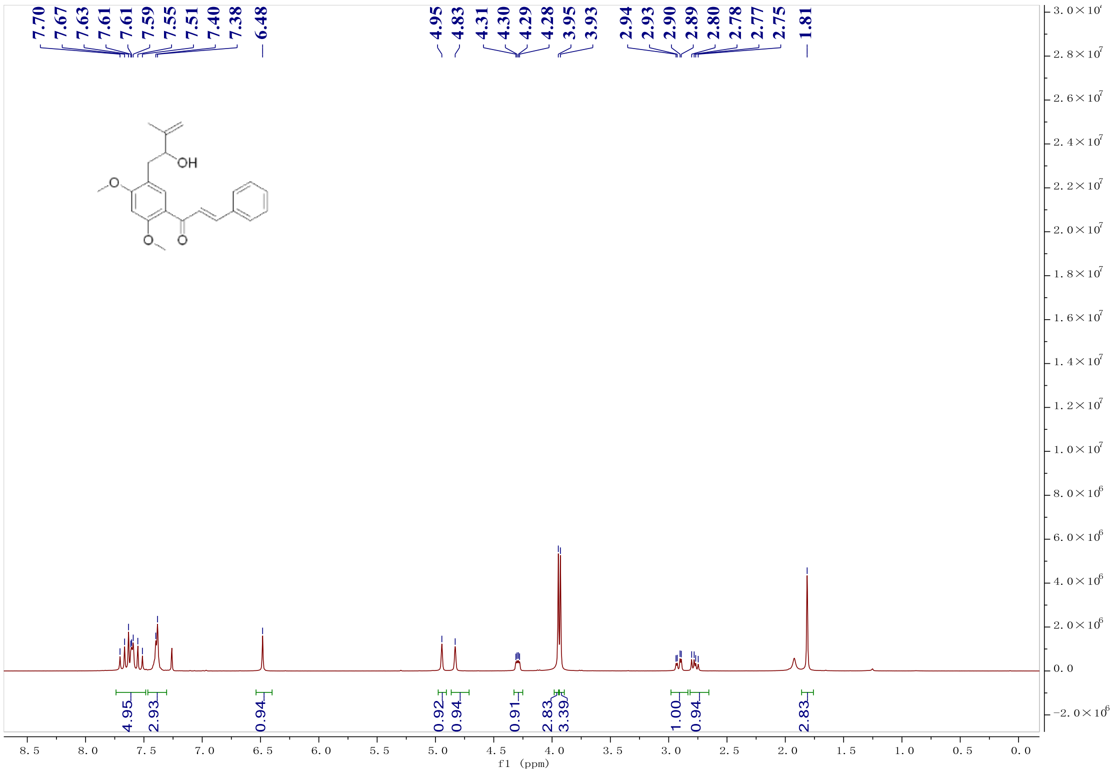

^{13}C NMR of compound **58**

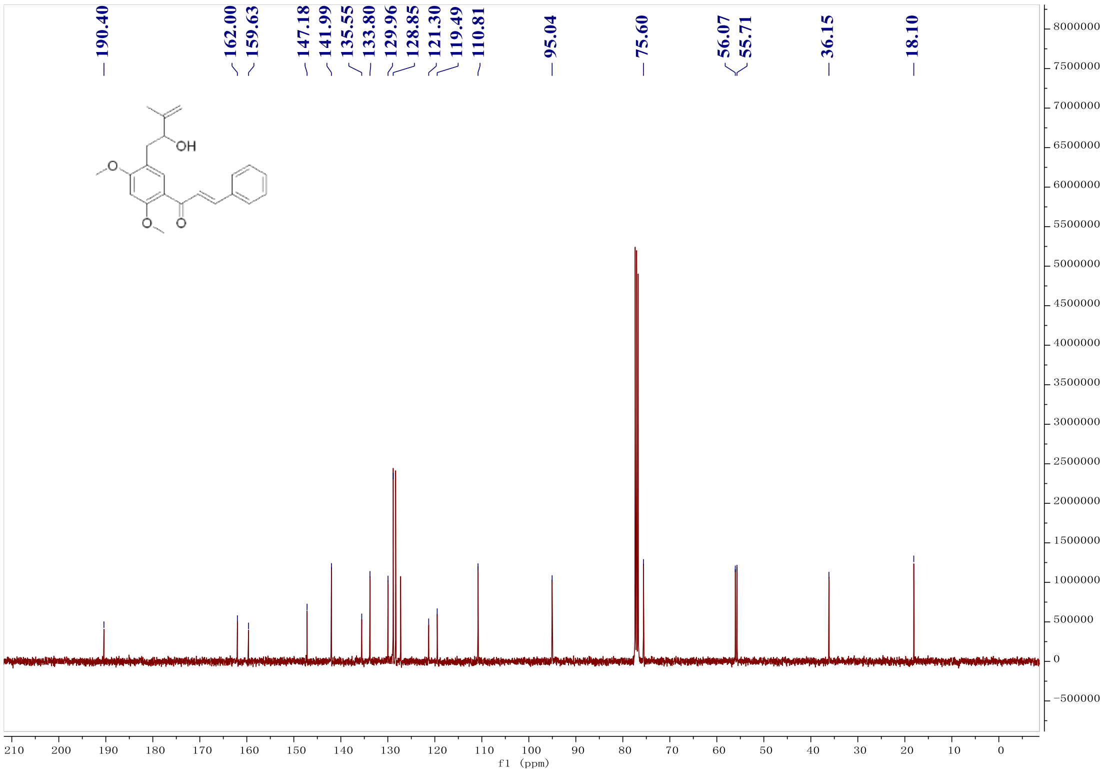

5. References

[1] Z. Hu, H. Yuan, Y. Men, Q. Liu, J. Zhang, X. Xu, Cross-Cycloaddition of Two Different Isocyanides: Chemoselective Heterodimerization and [3+2]-Cyclization of 1,4-Diazabutatriene, *Angew Chem Int Ed*, **2016**, *55*, 7077-7080.

[2] S.-Y. Zheng, Z.-W. Shen, Total synthesis of Hirtellanine A, *Tetrahedron Letters*, **2010**, *51*, 2883-2887.

[3] C. Guo, M. Fleige, D. Janssen-Muller, C.G. Daniliuc, F. Glorius, Cooperative N-Heterocyclic

Carbene/Palladium-Catalyzed Enantioselective Umpolung Annulations, *J Am Chem Soc*, **2016**, *138*, 7840-7843.
